# Synthesis and Structure of Group 13 POCOP Complexes

**DOI:** 10.1021/acs.inorgchem.5c05278

**Published:** 2026-04-03

**Authors:** Sanjukta Pahar, Taylor Wilde, Kushagra Agrawal, Nathan T. Coles, Benson M. Kariuki, Deborah L. Kays, Andrew J. Logsdail, Thomas Wirth, Emma Richards, Rebecca L. Melen

**Affiliations:** † Cardiff Catalysis Institute, School of Chemistry, 2112 Cardiff University, Translational Research Hub, Maindy Road, Cathays, Cardiff, Cymru/Wales CF24 4HQ, U.K.; ‡ School of Chemistry, Cardiff University, Main Building, Park Place, Cardiff, Cymru/Wales CF10 3AT, U.K.

## Abstract

In this study, we explore the coordination complexes of the resorcinol bis­(diphenylphosphinite) (POCOP) pincer ligand with Group 13 (aluminum, gallium and indium) metal centers. We report the synthesis of six new Group 13 POCOP complexes that have all been structurally characterized. The aluminum and indium complexes all crystallize in a five-coordinate “closed” geometry; however, for gallium, an “open” four-coordinate complex is additionally observed in the solid state, similar to an intramolecular frustrated Lewis pair.

## Graphical abstract

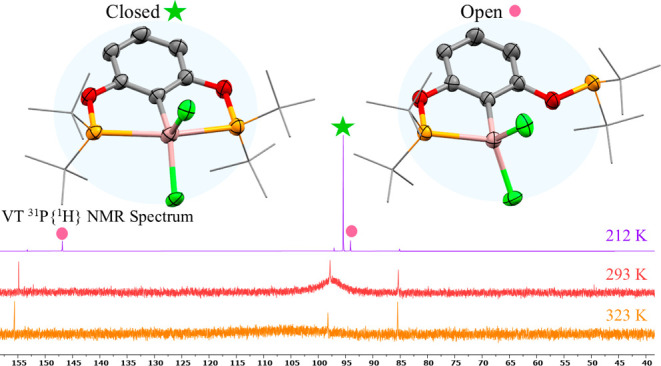

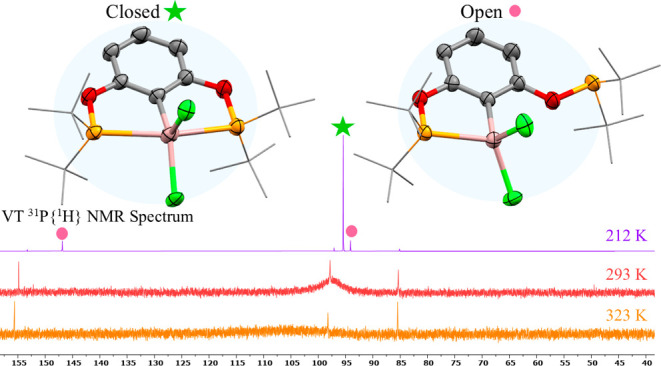

## Introduction

Over the past decade, there has been an increasing interest in the synthesis of novel Group 13 complexes for applications in polymerization,^1–4^ small molecule activation,^5–7^ and catalysis.^8–10^ Aluminum complexes attract attention due to the high abundance, low cost, and relatively low toxicity of aluminum.^10^ In our previous studies, we have focused on the preparation of homoleptic aluminum compounds containing electron-withdrawing monodentate ligands.^11^ In contrast, polydentate ligands can offer advantages over monodentate ligands, including enhanced stability and/or increased reactivity of the resulting metal complexes for catalytic applications.^12–18^ By varying the steric and electronic properties of the ligand donor atoms, as well as the number of coordinating groups, the Lewis acidity and stability of the metal center can be finely tuned to modulate the desired properties. Bidentate, tridentate and tetradentate ligands comprising nitrogen or oxygen-based donor atoms (e.g., NacNac, Salen) have been widely explored as ligands for aluminum.^19–21^ Monoanionic tridentate ligands are less studied within main group chemistry,^22–30^ especially when considering softer donors.^31–34^ Both PNP and NCN pincer ligands in combination with Al have shown that dynamic coordination is possible in solution, highlighting that hemilability is a design factor that could be leveraged to access more effective catalysts.^23,31^ This is further highlighted in other systems bearing chelating ligands where ligand hemilability has facilitated the development of tunable ring-opening polymerization catalysts for both Al and In,^35,36^ as well as increased stability of Al dehydrocoupling catalysts.^37^ In transition metal complexes, tridentate pincer ligands, containing a central donor atom flanked by two other donating centers, have played an important role in organometallic chemistry and homogeneous catalysis.^12–18^ In particular, the tridentate monoanionic resorcinol bis­(diphenylphosphinite) (POCOP) pincer ligand (^R^POCOP = 1,3-(OPR_2_)­C_6_H_3_) has been widely used,^38–41^ especially with late transition metals,^15,42–45^ since its first synthesis in 2000 (Figure 1).^46^ POCOP ligands have a central arene, which contains an anionic C-donor atom and is flanked by two phosphinite moieties. The ease of synthesis and derivatization of this ligand (at phosphorus or on the aromatic backbone), to regulate the ligand’s steric and electronic properties, has led to widespread use in coordination chemistry and organometallic catalysis.^41^ Despite extensive application in transition metal chemistry, the ^R^POCOP ligand has not previously been coordinated to Group 13 elements. In this study, we were interested in the synthesis of ^*t*Bu^POCOP complexes with heavier Group 13 elements, such as aluminum, gallium and indium, to explore how a softer, potentially hemilabile pincer framework interacts with these metals. In particular, we sought to determine whether the POCOP ligand could access alternative coordination modes and dynamic behavior. This may enable distinct reactivity patterns in small-molecule activation and catalysis relative to more rigid ligand systems.

**Figure 1:**
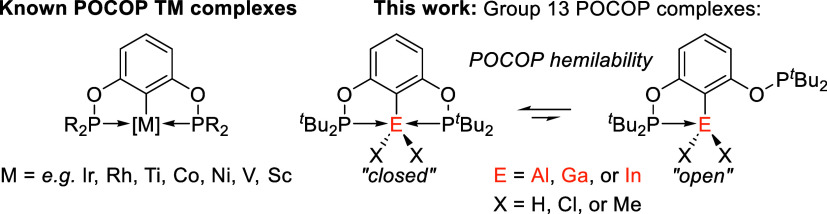
^R^POCOP pincer metal complexes, and ^*t*Bu^POCOP Group 13 complexes presented in this work. R = ^*i*^Pr, ^*t*^Bu.

## Results and Discussion

Initially, the ligand precursor [^*t*Bu^POCOP]Br (2,6-(^*t*^Bu_2_PO)_2_C_6_H_3_Br, **1**) was synthesized following literature procedures from the reaction of 2-bromoresorcinol with 2.5 equiv of di-*tert*-butylchlorophosphine (^*t*^Bu_2_PCl) in the presence of excess triethylamine (NEt_3_, 2.7 equiv).^39^ The reaction of the lithiated ligand with various Group 13 metal precursors was then explored (Scheme 1). In situ lithiation of [^*t*Bu^POCOP]­Br, using one equivalent of ^*n*^BuLi, forming [^*t*Bu^POCOP]­Li, followed by reaction with 1.1 or 1.2 equiv of AlCl_3_, GaCl_3_ or InCl_3_ led to the new Group 13 POCOP complexes [^*t*Bu^POCOP]­AlCl_2_ (**2**), [^*t*Bu^POCOP]­GaCl_2_ (**3**), and [^*t*Bu^POCOP]­InCl_2_ (**4**) in 40%, 59%, and 31% yield, respectively. Alternatively, reaction of the in situ generated [^*t*Bu^POCOP]Li with AlMe_2_Cl (1.2 equiv) led to the dimethyl aluminum POCOP complex [^*t*Bu^POCOP]­AlMe_2_ (**5**) in 27% yield. The POCOP aluminum dihydride complex [^*t*Bu^POCOP]­AlH_2_ (**6**) could also be prepared by two separate routes: the reaction of the in situ generated [^*t*Bu^POCOP]Li with AlH_3_·NMe_2_Et (1.2 equiv), and by the reduction of **2** with AlH_3_·NMe_2_Et (2.1 equiv). This gave **6** in 33% and 20% yields, respectively. Finally, if [^*t*Bu^POCOP]­AlCl_2_ (**2**) was reacted with just one equivalent of AlH_3_·NMe_2_Et, a mixture of both complex **6** and the unsymmetrically substituted [^*t*Bu^POCOP]­Al­(H)Cl complex (**7**) were formed. For this mixture, an isolated yield was not possible as the two compounds crystallized simultaneously and could not be separated without decomposition. In all cases, an upfield shift is observed in the ^31^P­{^1^H} NMR spectrum of complexes **2**–**7** (δ = 146.88–93.21 ppm) relative to **1** (δ = 155.23 ppm). Note that the isolated yields given for all Al complexes are relatively low owing to the similar solubility of the target complexes with protonated ligand and minor impurities, which makes clean isolation challenging and often requires several rounds of purification. Complexes **2**–**7** are also highly reactive to traces of moisture, leading to protonation of the ligand, as can be discerned by observation of a diagnostic signal in the ^31^P­{^1^H} spectrum at δ = 152–150 ppm for [^*t*Bu^POCOP]­H.

**Scheme 1:**
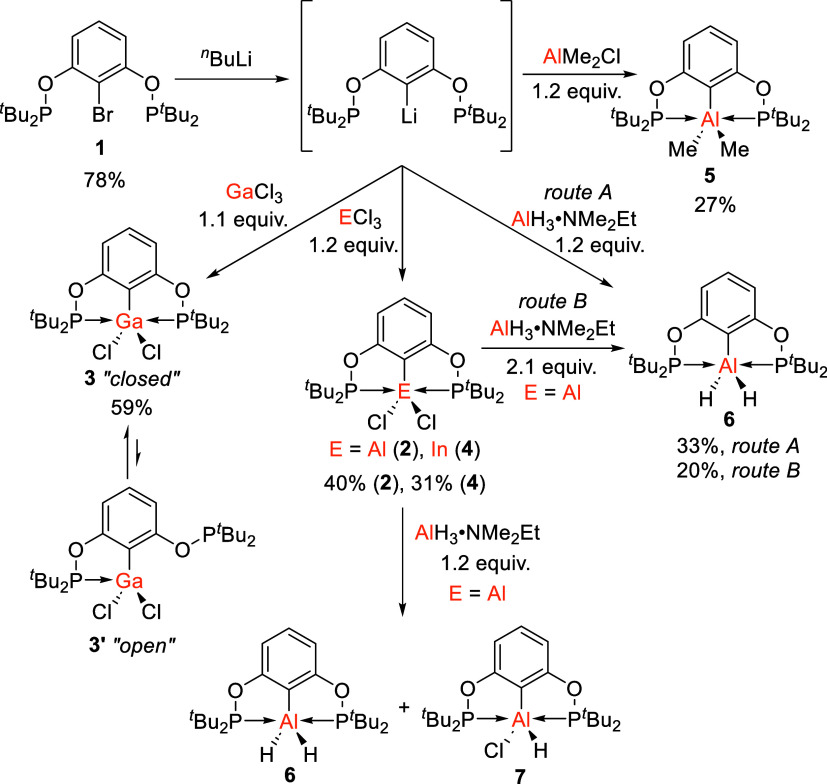
Synthesis of Group 13 ^*t*Bu^POCOP Complexes **2**–**7** ^a^

a Isolated yields are reported with the exception of the mixture of **6** and **7**. All reactions performed at room temperature. Lithiation of **1** was performed in pentane, and was added to the Group 13 reagent in THF.

All of the compounds could be recrystallized by slow evaporation from highly concentrated solutions of aromatic solvents (toluene or benzene), under an inert atmosphere, to yield crystals suitable for structure determination by single crystal X-ray diffraction analysis (Figure 2). For the aluminum complexes (**2**, **5**–**7**), all are five-coordinate with both phosphorus atoms coordinating to the aluminum center. The P–Al bond lengths range between 2.571(2)–2.762(1) Å, with the longest bond P–Al lengths observed for **5**. The Al–C­(arene) bond lengths range from 2.005(2) Å in complex **2** to 2.035(2) Å in complex **5**. To determine the geometry at each Al center in the solid state, the structural parameter τ_5_ were calculated for all complexes to assess the extent of a square pyramidal geometry relative to a trigonal bipyramidal geometry. Values of 0.37 and 0.46 for **2**, 0.38 for **5**, approximate values of 0.51 for **6**, and 0.17 and 0.28 for **7** were obtained. It should be noted that **2** and **7** have two structural parameters due to disorder of the AlX_2_ group within the binding site and that accurate τ_5_ values for **6** and **7** cannot be obtained as the exact location of the hydrides have not been accurately determined, having been only located within the electron density maps from the X-ray diffraction measurements. With the exception of **6**, all of the Al containing structures have structural parameter values less than 0.5, indicating that they are closer to square pyramidal in geometry.

**Figure 2:**
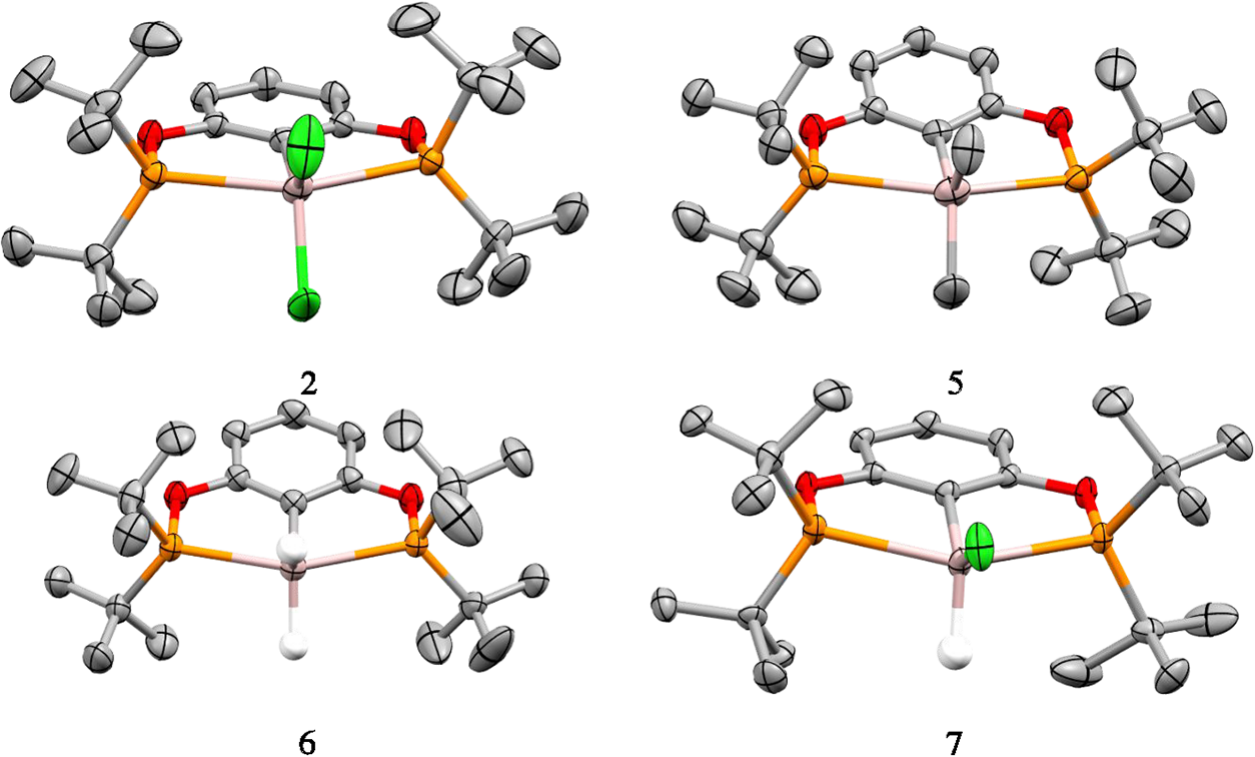
Solid state structures of aluminum ^*t*Bu^POCOP complexes **2**, **5**–**7**. C is shown in gray, H in white, O in red, P in orange, Cl in green, and Al in pink. Hydrogen atoms, except those attached to aluminum, are omitted for clarity. Disorder is omitted for clarity (**2** and **7**). Thermal ellipsoids are shown at 50% probability.

Structural comparisons to similar complexes in the literature are relatively difficult due to the large differences in bond lengths of related NCN and PNP pincer systems (Figure 3). Regardless, AlCl_2_ and AlH_2_ complexes bearing aryl-diamine ligands synthesized by Cowley et al.,^22^ Hey-Hawkins et al.,^23^ and aryl-dimine complexes by Vidović et al.^47^ show shortened Al–C­(arene) bonds (1.912(7)–1.9446(18) Å) compared to complexes **2**, **5**–**7** (2.005(2)–2.035(2) Å). As expected, these complexes also show notably shorter N–Al bonds (2.233(5)–2.321(3) Å) than the corresponding P–Al bonds (2.571(2)–2.762(1) Å). The P–Al bonds are marginally elongated for **2**, **5**–**7** when compared to the PNP ligand system used by Fryzuk (2.509(7)–2.542(8) Å).^31^ One marked difference is that complex **5** bearing an AlMe_2_ group is a 5-coordinate structure in the solid state, which differs to NCN systems where there is a tendency toward tetrahedral coordination as shown by Mu et al.^48^ Comparison to the AlEt_2_ complex by Hey-Hawkins et al. is not discussed due to potential unresolved disorder of the central Al atom in the deposited structure. In terms of τ_5_ parameters, the flexible PNP ligand displays coordination much closer to trigonal bipyramidal. This is attributed to the presence of two large Si atoms within the backbone allowing for a N–Al–N angle of 171.8(3), which is closer to linearity than those in complexes **2**, **5**–**7** (149.12(7)–156.1(4)°). NCN complexes by Cowley et al. and Hey-Hawkins et al. display τ_5_ values that deviate toward square pyramidal geometry (0.54–0.58) but retain a marginal preference toward trigonal bipyramidal geometry, which is also seen for complex **6**. However, this contrasts with complexes **2**, **5** and **7** which are perturbed toward square pyramidal geometry (0.17–0.46). This is attributed to both the larger Al–C­(arene) and P–Al bonds (compared to N–Al), leading to more acute P–Al–P angles.

**Figure 3:**
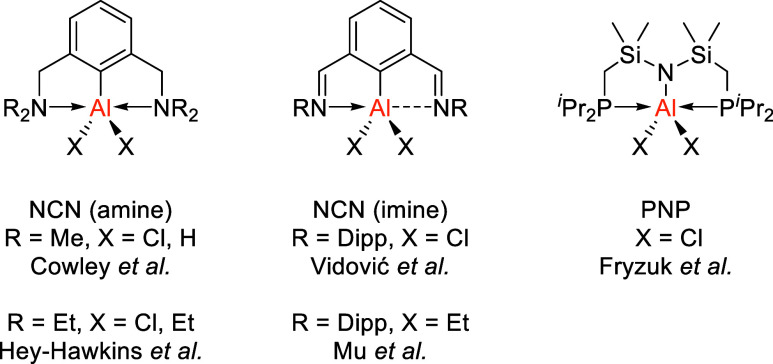
Structurally related NCN and PNP pincer complexes. The dotted line for the NCN (imine) complexes represents ambiguity in the bonding nature in the solid state due to vastly differing N–Al distances. Dipp = 2,6-diisopropylphenyl.

While the aluminum ^*t*Bu^POCOP complexes had similar structural characteristics in the solid state, their behavior in solution varied. Complexes **2**, **6** and **7** displayed single sharp signals as evidenced by small full width at half-maximum peak height (FWHM = ν_1/2_) in the ^31^P­{^1^H} NMR spectra at δ = 96.9 ppm (ν_1/2_ = 13.5 Hz), 99.3 ppm (ν_1/2_ = 7.5 Hz) and 98.5 ppm (ν_1/2_ = 4.2 Hz), respectively. The ^1^H and ^13^C­{^1^H} NMR spectra of **2**, **6** and **7** also indicate a symmetrical molecule is observed, indicating retention of a *C*_2*v*_ 5-coordinate geometry in solution. For the methylated congener **5** however, broad peaks at δ ∼ 110 ppm (ν_1/2_ = ∼700 Hz) and δ ∼ 102 ppm (ν_1/2_ = 73.3 Hz) were observed in the ^31^P­{^1^H} NMR spectrum, which suggested some fluxionality of the AlMe_2_ moiety within the ligand. To probe this further, variable temperature NMR spectroscopy was conducted. As the temperature was reduced, the broad peaks sharpened, yielding a more complex NMR spectrum (see Figure 4). The major peak is observed at δ = 107.6 ppm (ν_1/2_ = 17.6 Hz) and is attributed to **5** where both phosphorus atoms are bound to the central metal, which we refer to as the “closed” conformer. The chemical shift of this peak is consistent with chemical shifts observed for complexes **2**, **4**, **6** and **7**, with the shift being most downfield due to the electron donating nature of the Me groups making the Al less Lewis acidic. At 232 K, two peaks in a ratio of 1:0.98 appear at δ = 141.2 ppm (ν_1/2_ = 28.3 Hz) and 109.6 ppm (ν_1/2_ = 27.5 Hz), which strongly suggests that these signals derive from a single species. The chemical shift at δ = 109.6 ppm (ν_1/2_ = 27.5 Hz) is consistent with an Al–phosphinite bond due to the large upfield shift (Δδ = −41.3 ppm) from the uncoordinated ligand. The upfield shift is consistent with borane-phosphinite^49–51^ and aluminum-phosphite complexes^52^ as well as complexes **2**, **4** and **6** presented herein which do not exhibit broadening in the ^31^P­{^1^H} NMR spectra. The signal at δ = 141.2 ppm (ν_1/2_ = 28.3 Hz) is much closer to that of starting material **1** (δ = 155.2 ppm), indicating dissociation of one of the Al–P bonds. We attribute this feature to an “open” conformer **5′**, which is depicted in Figure 4. The splitting of this signal at low temperatures, indicating an “open” conformer, is in agreement with observations made by Fryzuk for a PNP-Al­(benzyl)_2_ complex.^31^ Additional evidence for an “open” conformer can be found vide infra in discussions on the [^*t*Bu^POCOP]­GaCl_2_ complex **3**/**3′**. The peak at δ = 101 ppm (ν_1/2_ = 4.6 Hz) is believed to be from traces of AlMeCl_2_ within the AlMe_2_Cl, leading to the formation of [^*t*Bu^POCOP]­Al­(Me)­Cl, which makes up ∼14% of the sample and integrates consistently at both 211 and 284 K in the ^1^H and ^31^P­{^1^H} spectra. The integrals in the ^1^H NMR spectra for this minor component agree with this assignment, as well as the upfield shift of the ^31^P NMR signal due to the loss of an electron donating Me group; however, it has not been possible to isolate this compound independently. Other smaller peaks also became visible while cooling the sample, which may relate to subtle changes between the “open” and “closed” conformations in solution, but these are minor components of the solution (<5%) and so cannot be definitively characterized.

**Figure 4:**
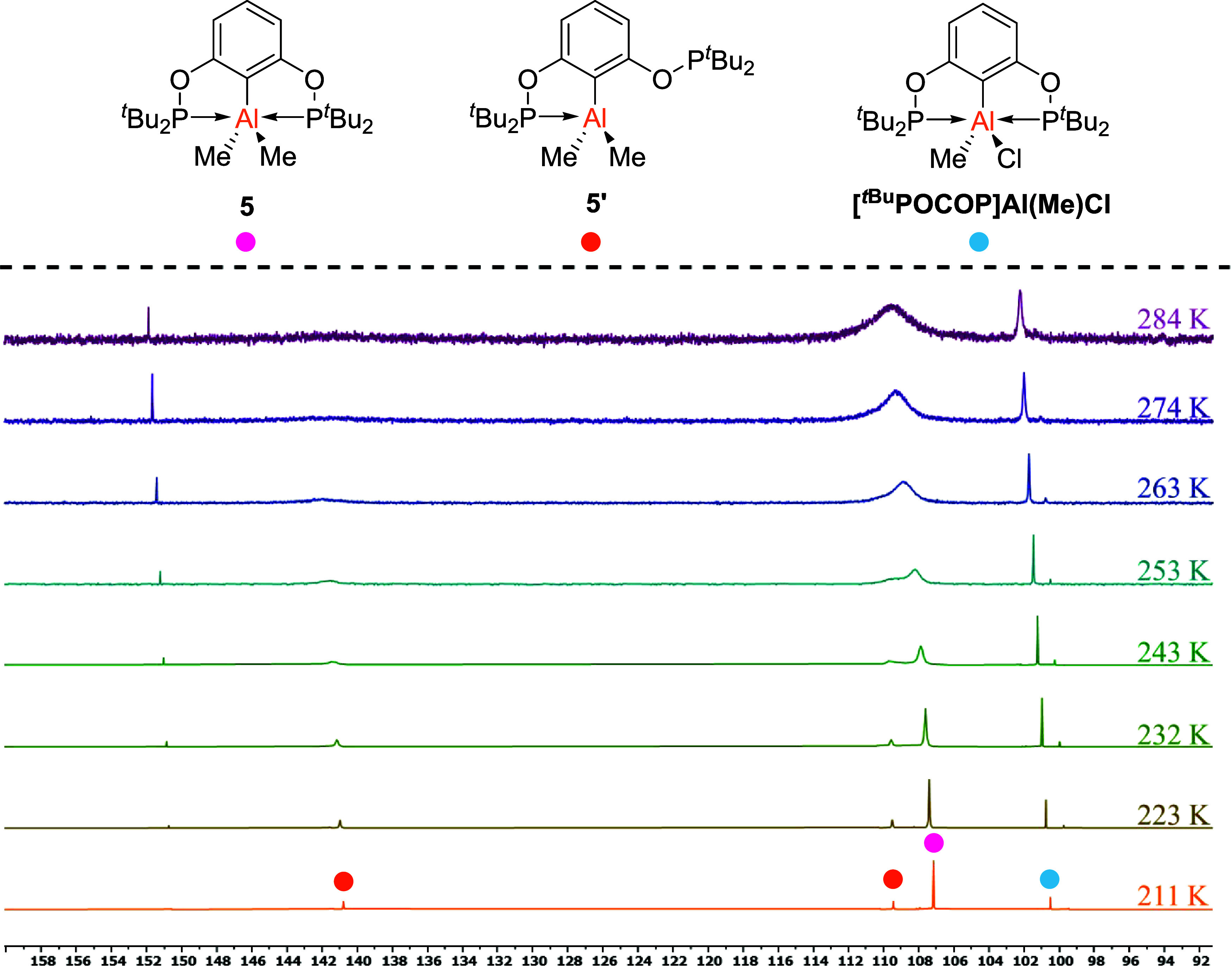
Top, structures observed in the variable temperature inverse gated ^31^P­{^1^H} NMR spectra (*d*_8_-toluene) of **5**. Bottom, variable temperature inverse gated ^31^P­{^1^H} NMR spectra (*d*_8_-toluene) of **5**.

The indium derivative **4** was initially synthesized with the intention of exploring how the POCOP ligand would bind to this slightly larger Group 13 center. From a structural perspective, complex **4** is similar to its aluminum analogue, **2**, containing a 5-coordinate metal center (Figure 5). There is an elongation of both the In–C­(arene) and In–P bonds relative to the Al–C­(arene) and Al–P bonds, as is expected due to the larger size of the indium atom. The structural parameter τ_5_ for both components of disordered **4** were calculated to be 0.29 and 0.33, which agrees with observations for the Al complexes, highlighting a distortion toward a square pyramidal geometry. Both the In–C­(arene) (2.03(3)–2.127(14) Å) and τ_5_ values (0.1–0.4) are in line with diamine-aryl complexes (NCN)­InCl_2_ and (NCN)­InI_2_.^53,54^ The similarities between **2** and **4** were also retained in solution, with **4** exhibiting a single signal in the ^31^P­{^1^H} NMR spectrum δ = 93.2 ppm (ν_1/2_ = 3.4 Hz), that is shifted slightly upfield relative to **2** but within the expected region. It is noted that **4** consistently contains a small contaminant at δ = 92.2 ppm in the ^31^P­{^1^H} NMR spectrum. This has been confirmed to be a small amount of a mixed Cl/Br species from halide exchange via mass spectrometry and NMR spectroscopy. Further details on this species can be found in the SI (Figure S33–35, S37).

**Figure 5:**
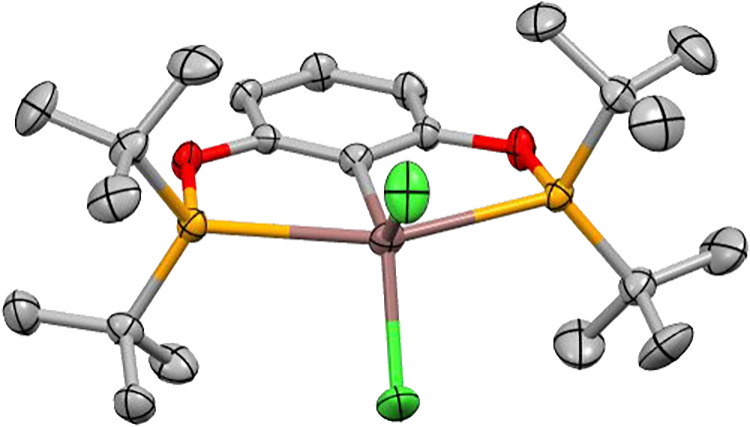
Solid state structure of indium POCOP complex **4**. C is shown in gray, O in red, P in orange, Cl in green, and In in dark pink. Hydrogen atoms are omitted for clarity. Disorder is omitted for clarity. Thermal ellipsoids are shown at 50% probability.

In contrast to the other complexes discussed thus far, the Ga complex exhibits more complex structural properties in the solid state. Two polymorphs have been isolated: one crystallizing in the space group *P*1̅ containing independent molecules with two differing conformations, **3** (“closed”) and **3′** (“open”), within the asymmetric unit (Figure 6). The second polymorph crystallized in the space group *P*2_1_/*c* containing a disordered mixture of the two differing conformations, with **3** being the major component (**3′** only constitutes ∼5% of the model). For the sample crystallizing in *P*1̅, one molecule of **3** (the “closed” conformer) bears similarities to those described above for aluminum and indium, where the gallium center is five-coordinate, with the ^*t*Bu^POCOP ligand behaving as a monoanionic tridentate ligand. For the disordered units of **3** in the *P*1̅ structure, the Ga–P bond lengths range from 2.624(10)–2.657(7) Å, which show marginal elongation compared to **2**, while the Ga–C­(arene) bond lengths of 1.998(8) and 1.978(10) Å are within the error of those observed for the aluminum derivatives. The Ga–C­(arene) bonds are marginally elongated relative to a NCN (amine) pincer bearing a GaCl_2_ moiety, synthesized by Cowley et al. (1.924(4) Å) matching the trend observed for the Al complexes.^55^ The five-coordinate structural parameters τ_5_ for **3**, which has two values due to disorder of the GaCl_2_, were 0.27 and 0.38, again implying a bias toward square pyramidal geometry, which is consistent with both **2** and **4**. The second species within the asymmetric unit was identified as the “open” conformation **3′**. **3′** features a four-coordinate gallium complex where one phosphinite arm has rotated away from the metal center meaning the ^*t*Bu^POCOP ligand is now coordinated as a monoanionic, bidentate ligand, demonstrating the hemilability of the ^*t*Bu^POCOP ligand. In **3′**, a shortening of the Ga–P bonds to 2.389(12) Å /2.4386(11) Å (**3′** disordered) is observed while the Ga–C­(arene) distances 1.96(3) Å/1.960(3) Å are within error to those of **3**. The “open” complex (**3′**) could resemble an intramolecular frustrated Lewis pair (FLP), where the molecule contains unquenched Lewis acidic (Ga) and Lewis basic (P) sites within the same molecule.^56–61^

**Figure 6:**
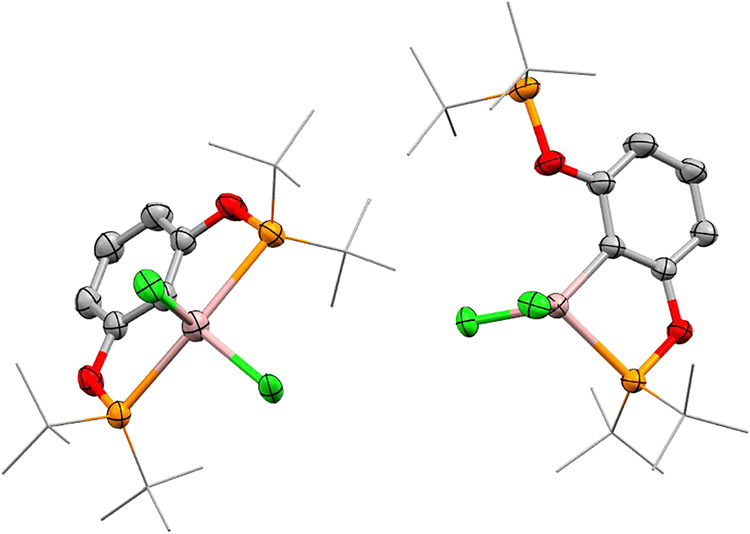
Solid state structure of the *P*1̅ polymorph showing both the “closed” (**3**, left) and “open” (**3′**, right) conformations of the [^*t*Bu^POCOP]­GaCl_2_ complex in the asymmetric unit. C is shown in gray, O in red, P in orange, Cl in green, and Ga in pink. Hydrogen atoms are omitted, and ^*t*^Bu groups are depicted as wire frames for clarity. Disorder is omitted for clarity. Thermal ellipsoids are shown at 50% probability.

As observed for **5**/**5′** at 294 K, the ^31^P­{^1^H} NMR spectrum of **3**/**3′** at 298 K also showed a particularly broad signal that could not be assigned to a single species. Further investigation of **3**/**3′** by variable temperature ^31^P­{^1^H} NMR spectroscopy in *d* ^8^-toluene was performed to better resolve the signals (Figure 7). Heating the sample of **3/3′** to 323 K led to further broadening of the signal at δ ∼ 100 ppm. However, upon cooling the sample to 211 K, the spectrum showed substantially improved resolution, with the appearance of three new sharp signals. The major signal (δ = 95.4 ppm) is in the region expected for the “closed” conformer, as observed for **2** and **4**, and so is attributed to complex **3**. The two new minor signals at δ = 94.1 ppm, consistent with an E–P bond, and δ = 146.9 ppm, consistent with an unbound P, integrate in a ratio of 1:1, which would be expected for the “open” conformation (**3′**). This agrees with the structural data where both the “open” and “closed” conformers were shown to be possible, further supporting the observations made for **5**/**5′**.

**Figure 7:**
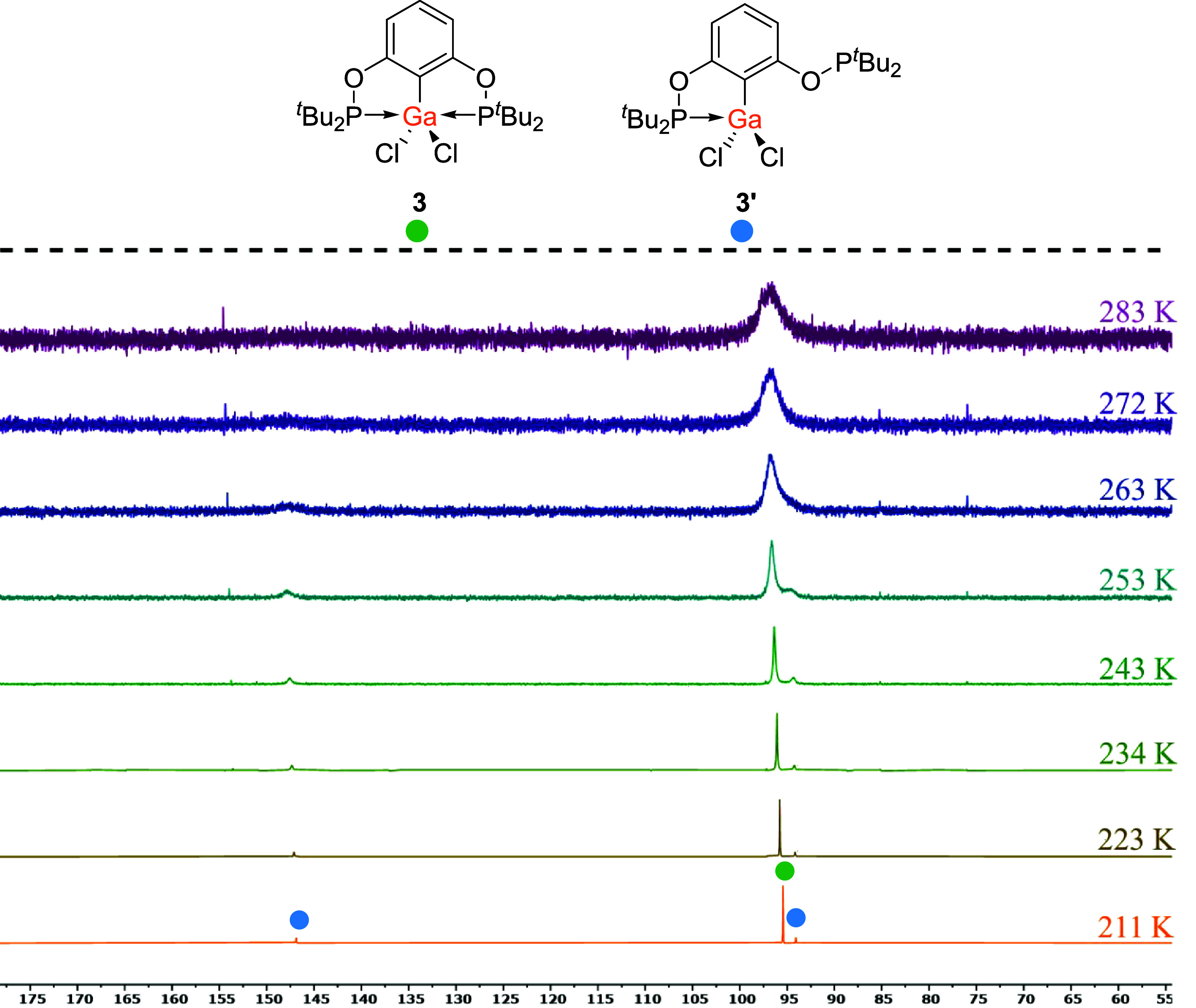
Variable temperature inverse gated ^31^P­{^1^H} NMR spectra (*d*_8_-toluene) of **3** and **3′** from 211–283 K.

To understand the difference in stability of the four- and five-coordinate complexes **2**, **3** and **4**, respectively, and to rationalize why hemilabile behavior is observed experimentally for some complexes but not others, we calculated the relative energies of the molecules using density functional theory (DFT). The DFT total energy calculations were performed with FHI-aims^62^ with the light (2020) basis set and the M06 exchange-correlation density functional.^63^ Further details of these method choices are provided in the Supporting Information. For these Group 13 structures, the geometries of the closed (**2**, **3** and **4**) and open (**2′**, **3′** and **4′**) complexes were optimized, and the energy differences between the two conformations were calculated (Figure 8). In all cases, the closed conformations are most stable, but the significantly smaller energy difference between the closed and open conformations for the gallium complex (14.13 kcal/mol), compared to the aluminum (17.20 kcal/mol) and indium (20.62 kcal/mol) analogues, is consistent with the experimentally observed increased propensity for hemilability in the gallium system. Indeed, the transition state energies for dissociation of one phosphine arm also show that the closed gallium complex (**3**) is the easiest to open to form **3′**, with a barrier of 17.83 kcal/mol. Opening the aluminum complex (**2**, **2′**) has a higher barrier of 19.50 kcal/mol, and the indium complex (**4**, **4′**) the highest barrier of 22.70 kcal/mol. The dissociation of the second phosphinite arm is calculated to be thermodynamically and kinetically unfavorable, with a barrier of >31 kcal/mol for Al, and Ga complexes (see SI, Figure S89). The In complex is unstable to dissociation of both phosphinite arms from the metal center. These calculated trends directly correlate with the variable temperature NMR and solid state observations, providing a computational rationale for the differing dynamic behavior across the Group 13 series. Orbital analysis was performed for the aluminum and gallium complexes (see SI, Figure S91), which shows that the lowest unoccupied molecular orbital (LUMO) for the closed (**2**, **3**) and open (**2′**, **3′**) complexes are localized on the aluminum or gallium metal center. This low-lying metal-centered LUMO is consistent with the Lewis acidic character of these complexes and supports their potential relevance for bond activation and catalysis. The LUMO + 1 orbital is located at the P–O bond of the ligand (P–O σ*-orbital) for the open aluminum (**2′**) and the gallium (**3**, **3′**) complexes, indicating an accessible antibonding interaction that rationalizes the experimentally observed susceptibility of these systems toward hydrolysis via P–O bond cleavage. The highest occupied molecular orbital (HOMO) and HOMO–1 for all cases (**2**/**2′** and **3**/**3′**) are localized on the aromatic ring of the ligand (see SI, Figure S92).

**Figure 8:**
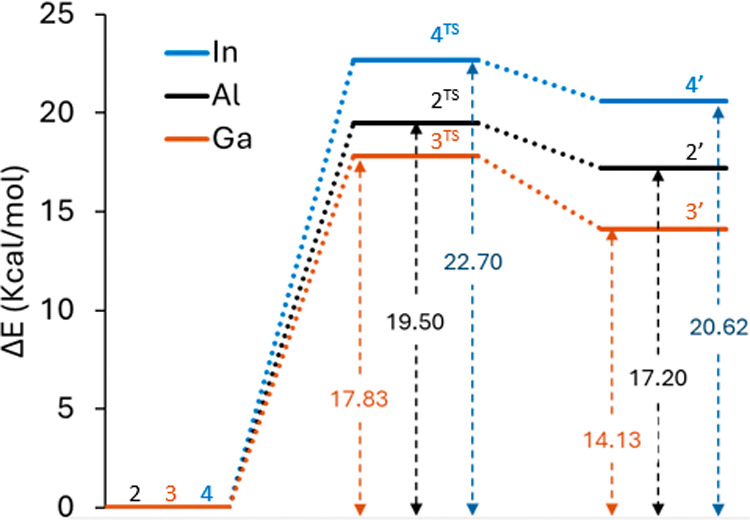
DFT calculated energy profile of POCOP complexes going from their closed (**2**, **3**, **4**) to open conformations (**2′**, **3′**, **4′**) via respective transition states (**2** ^**TS**^, **3** ^**TS**^, **4** ^**TS**^).

Finally, we investigated the use of the hydride complex in the activation of small molecules. Aluminum hydrides have been shown to undergo insertion into a range of unsaturated molecules including CO_2_,^64–67^ ketones,^37,68^ carbodiimides^37,69^ and isocyanates.^64,69^ To assess how the hemilability of ^*t*Bu^POCOP influences reactivity toward these substrates, hydride complex **6** was treated with 4-fluorophenyl isocyanate, DCC (*N*,*N*′-dicyclohexylcarbodiimide), benzophenone and CO_2_. Reactivity of the above molecules was monitored in situ by **^1^** H and ^31^P­{^1^H} NMR spectroscopy using a C_6_D_6_ solution of complex **6** (0.01 mmol).

Addition of **6** to 1 equiv of isocyanate did not result in any substantial reactivity, even after 16 h. Reactivity with 2 or 4 equiv immediately led to a highly complex mixture of products. It was not possible to extract the identity of any of the species formed.

Reactions with benzophenone and **6** have proved more fruitful. When reacting 1, 2 and 3 equiv of benzophenone with **6**, a peak at δ = 6.45 ppm in the ^1^H NMR spectrum is observed indicating reduction of the benzophenone, with an accompanying carbon peak at δ = 77.8 ppm in the ^13^C­{^1^H} spectrum. These shifts are consistent with other aluminum-alkoxide complexes of this type.^37,68,70–72^ When 1 equiv of benzophenone was used, residual hydride complex **6** was detected in solution. This suggested that the product from reactivity with one equivalent of benzophenone led to favorable reactivity with a second equivalent, likely due to dissociation of the phosphinite arms from the Al center. This was further confirmed by reactivity with 2 and 3 equiv of benzophenone which yielded ^1^H NMR spectroscopic signals identical to the product obtained when 1 equiv was used (Figure S75, SI). The ^31^P­{^1^H} spectrum of these reactions is more complicated, with 3 major peaks at δ = 149.8 ppm, δ = 148.2 ppm and δ = 145.3 ppm, indicating dissociation of the phosphinite arms in solution (Figure S76, SI). A fourth signal is observed at δ = 152.5 ppm which is believed to be protonated ligand. This is further complicated by the absence of a correlation peak for one of the ^*t*^Bu signals in the ^1^H–^31^P HMBC spectrum at δ = 0.98 ppm (see Figure S68). Crystals suitable for X-ray diffraction were obtained by placing a concentrated pentane solution of the crude reaction mix of **6** and 2 equiv of benzophenone at −32 °C. As the reactions with varying quantities of benzophenone had suggested, we obtained structural evidence of a compound that has undergone double substitution, yielding a dialkoxide complex (Figure S88, SI). The complex shows one bound and one unbound phosphinite at the aluminum center. This may indicate why the solution data for this species is relatively complex, as there is a large amount of steric bulk imparted from the alkoxides, hindering free rotation. We also can not be certain that there are not other species that are formed as we were only able to obtain one crystal that was suitable for diffraction as all crystals were coated in a viscous oil and readily degraded at room temperature.

We then turned our attention to the reactivity of a carbodiimide. When 1.5 equiv of DCC were reacted with **6** at 50 °C, one major species was yielded. Completion of the reaction was marked by a ^31^P­{^1^H} NMR spectroscopic shift to δ = 140.7 ppm, consistent with dissociation of the phosphinite arms. In the ^1^H NMR spectrum, the *m*-Ar protons of the ^*tBu*^POCOP ligand shift upfield by Δδ = 1.04 ppm, overlapping with a sharp singlet at δ = 7.84 ppm. This singlet is assigned to hydride insertion into the carbodiimide, forming an aluminum-bound amidate. The ^13^C­{^1^H} NMR spectrum further supports this assignment, showing a new resonance at δ = 162.0 ppm. Although the alkyl region is challenging to fully deconvolute, broad signals at δ = 56.0 and 56.7 ppm lie in the expected region for N–C bonds. The broadening of these resonances is also consistent with amine/imine tautomerism of an amidate group. Crystals of the complex were obtained by dissolving the crude reaction mixture in pentane and cooling to −30 °C. It was found that rather than the monoinsertion product expected, the complex had undergone reactivity with 2 equiv of DCC yielding complex **8** (Figure 9). The Al–N bond lengths (1.9434(13)–2.0281(13) Å), C–N bond lengths (1.3100(19)–1.3273(19) Å) and N–C–N angles (112.24(13)–112.97(13)°) are consistent with those observed for other Al-amidinate complexes.^69,73–75^

**Figure 9:**
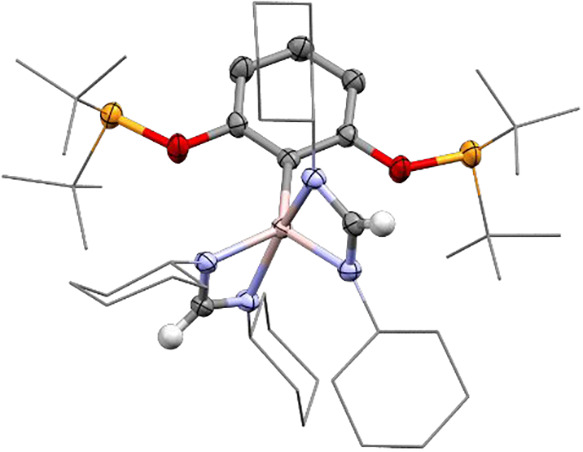
Solid state structure of complex **8**. C is shown in gray, H in white, N in blue, O in red, P in orange and Al in pink. Hydrogen atoms, except those derived from AlH_2_, Cy and ^*t*^Bu groups are depicted as wire frames for clarity. Disorder is omitted for clarity. Thermal ellipsoids are shown at 50% probability.

The reactivity of **6** toward CO_2_ has proven to be highly challenging. The addition of CO_2_ gives somewhat unpredictable mixtures of products and is highly dependent upon factors such as quantity of material reacted, headspace of the flask and the temperature of addition (see SI, Section 4 for further details on product distributions). Performing reactions with 5 mg of **6** it was possible to observe a sole species in the ^31^P­{^1^H} NMR spectrum at δ = 100.2 ppm (Figure S71). It was found that, during the course of characterizing this species, it degrades at room temperature and is completely consumed within 24 h. While trying to scale up the reaction (50 mg) we reliably obtained very complex spectra where two major complexes formed, one at δ = 100.2 ppm and a second more stable species at δ = 145.3 ppm in the ^31^P­{^1^H} NMR spectrum. From both of these scales we have managed to postulate the identity of two possible products from the reactivity of **6** with CO_2_.

For the signal at δ = 100.2 ppm, evidence of CO_2_ reduction was observed in the ^1^H NMR spectrum, with a triplet in the formate region at δ = 8.62 ppm, integrating to 1H relative to the *meta*-Ar–H peak at δ = 6.74 ppm of the CO_2_ adduct. This was accompanied by a broad peak at δ = 5.45 ppm indicative of a Al–H bond suggesting this species resulted from monoaddition of CO_2_ to the dihydride complex (Figure 10, left). This was further evidenced in the ^13^C­{^1^H} spectrum where a triplet at δ = 166.8 ppm, further supporting the formation of a monoformate species. The *tert*-butyl groups display asymmetry as they split into two doublets, which is to be expected for an asymmetric molecule. Due to the instability of this species, we have been unable to structurally validate the compound. Mass spectrometry of this species was also unsuccessful. From the NMR spectra obtained, the chemical shifts for the formate is in line with other Al-formate complexes where the signals are observed at δ = 6.98–8.52 ppm and δ = 161.6–163.8 in the ^1^H and ^13^C­{^1^H} spectra, respectively.^64,66,76,77^ The signals obtained for the POCOP ligand across all spectra are also consistent with closed complexes **2**, **6** and **7** herein.

**Figure 10:**
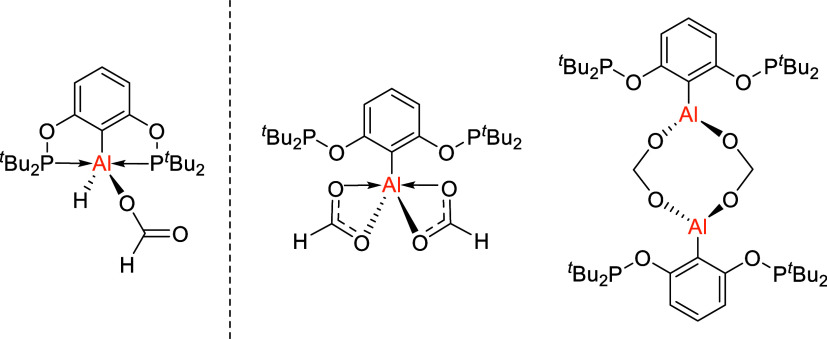
Left, proposed structure for the major species initially formed upon reaction of **6** with CO_2_. Right, proposed structures for the major product remaining 24 h after addition of CO_2_.

The second species identified on increasing the scale to 50 mg at δ = 145.3 ppm in the ^31^P­{^1^H} NMR spectrum had similar spectroscopic signals to the postulated monoformate above. A broad singlet at δ = 8.14 ppm was detected in the^1^H NMR spectrum that integrated to 2H relative to the *meta*-Ar–H of the POCOP ligand which had now shifted to δ = 7.61 ppm, indicative of an open conformation of the ligand (see discussion of **3**/**3′**, **5**/**5′** vide infra). The ^13^C­{^1^H} spectrum showed a correlation peak between the proton signal at δ = 8.14 ppm and the carbon signal at δ = 166.6 ppm. Aluminum formate (Al­(O_2_CH)_3_(H_2_O)) is reported to have chemical shifts in the ^1^H and ^13^C­{^1^H} NMR spectra at δ = 8.2 ppm and δ = 166 ppm, respectively.^78^ This may suggest that a diformate species has formed, where the phosphinite arms are no longer bound to the Al, akin to complex **8**. Alternatively, bridging complexes where the formate has undergone a second reduction of the CO_2_ to obtain a bridging acetal are known. Driess et al. have shown a NacNac derived acetal dimer to have chemical shifts of δ = 4.74 ppm (^1^H) and δ = 85.7 ppm (^13^C).^79^ This substantially contrasts the dimeric acetal complex formed with a bis­(phosphoranyl)­methanido ligand by So et al. which displays ^1^H and ^13^C­{^1^H} NMR spectroscopic signals at δ = 8.24 ppm and δ = 161.3 ppm, respectively.^66^ Attempts to obtain mass spectrometry data to identify a monomeric or dimeric structure proved unsuccessful. We also sought to determine the diffusion coefficients of the molecule through DOSY measurements, but as we were unable to separate the major species from the side products the ^1^H NMR spectrum was too cluttered to obtain meaningful conclusions as to the identity.

## Conclusion

In conclusion, we have prepared six new Group 13 complexes stabilized by the POCOP ligand and these have all been structurally characterized. The gallium complex, [^*t*Bu^POCOP]­GaCl_2_ (**3**/**3′**), shows the existence of both a “closed” and an “open” coordination complex in the solid state and in solution. The [^*t*Bu^POCOP]­AlMe_2_ also displays fluxional coordination in solution, with both the “closed” (**5**) and “open” (**5′**) conformers observed through VT NMR studies. DFT calculations support that the ligand is hemilabile when coordinated to gallium, allowing a four-coordinate complex to be accessed. This open conformation is reminiscent of an intramolecular FLP where the molecule contains unquenched Lewis acidic and basic sites. Initial investigations into the reactivity of hydride complex **6** shows that it can insert into unsaturated small molecules such as carbodiimides and CO_2_, however, these show varying stability which is likely to preclude their use in catalysis.

## Experimental Section

### General Experimental

All reactions, characterizations and manipulations were carried out utilizing standard Schlenk techniques under an inert N_2_ atmosphere and with an oil pump to supply vacuum. A glovebox (MBraun) with an atmosphere of dinitrogen was used for product isolation, crystallizations and preparations for reactions or analysis. Clean, oven-dried glassware would be cycled with N_2_ before use. Filtration mediums were oven-dried at 120 °C for 24 h prior to use. All solvents used were either dispensed from the solvent purification system (MB SPS-800) or manually distilled and/or dried and degassed. All dry solvents were stored under a nitrogen or argon atmosphere over dried 3 Å molecular sieves. AlCl_3_ was purified by sublimation before use. Benzene-*d*_6_ (C_6_D_6_) for NMR spectroscopy was dried over potassium metal and purified by distillation prior to use, and toluene-*d*_8_ was dried over 3 Å molecular sieves. Remaining chemicals and reagents not already mentioned were commercially purchased from suppliers with no further purification. ^1^H, ^13^C­{^1^H}, ^31^P and ^31^P­{^1^H} NMR spectra were recorded on Bruker Avance II 400 or Bruker Avance 500 spectrometers. Chemical shifts are expressed as parts per million (ppm, δ) downfield of tetramethyl silane (TMS) and are referenced to C_6_D_6_ (^1^H: 7.16 ppm/^13^C: 128.06 ppm) or toluene-*d*_8_ (^1^H: 2.08, 6.97, 7.01, 7.09 ppm/^13^C: 20.43, 125.13, 127.96, 128.87, 137.48 ppm) as internal standards. The description of signals includes s = singlet, d = doublet, t = triplet, q = quartet, m = multiplet, br. = broad and app. = apparent. All coupling constants are absolute values and are expressed in Hertz (Hz). Yields are given as isolated yields. Mass spectra were measured on a Thermo scientific Exactive GC-MS spectrometer and ions were generated by electron ionization (EI) or a Waters Xevo G2-XS QTOF spectrometer via electrospray ionization (ESI) or atmospheric pressure chemical ionization (ACPI). The molecular ion peak values are quoted for molecular ion (M^+^). Elemental analysis (EA) samples were weighed using Mettler Toledo high precision scale and analyzed using ThermoFlash 2000 at London Metropolitan University’s EA service. Acetanilide was used as standard for EA within ±0.23% for carbon, ±0.07% for hydrogen and ±0.09% for nitrogen of the calculated value. Full experimental procedures and characterization data can be found in the Supporting Information. ***Caution!*** Synthetic procedures listed below require the use of Schlenk apparatus that use cryogenic liquid nitrogen. Extreme care should be taken both in the handling of the cryogenic liquid nitrogen and its use in the Schlenk line trap to avoid the condensation of oxygen from air. ***Caution!*** *n*-Butyllithium is pyrophoric and must be handled using proper needle and syringe techniques. All manipulations used less than 5 mL of a 2.5 M solution in hexanes and were carefully quenched in a 1:1 isopropanol–toluene mixture after addition.

### Synthesis of **1** ([^tBu^POCOP]­Br)

This synthetic method was used following a procedure published in literature.^39^ Dry 2-methyltetrahydrofuran (20 mL) was added to 2-bromoresorcinol (1.00 g, 5.29 mmol), forming a clear solution in which ^*t*^Bu_2_PCl (2.52 mL, 13.23 mmol) and triethylamine (2.02 mL, 14.29 mmol) were added dropwise. The mixture was then stirred for 12 h at 90 °C. A white suspension was observed, and the solvent was then removed in vacuo. The remaining solid was extracted with hexane (2 × 20 mL) and the pale-yellow filtrate solution was cooled to −18 °C in a freezer for 3 days. The crystalline product was separated from the solution by canula filtration, and the solids were dried under vacuum to obtain pure compound **1** (1.84 g, 3.86 mmol, 73%) as colorless crystals. ^**1**^ **H NMR** (400 MHz, C_6_D_6_, 298 K): δ = 7.31–7.28 (m, 2H, *m-*Ar*H*), 6.96 (t, ^3^ *J*_H–H_ = 8.3 Hz, 1H, *p-*Ar*H*), 1.15 (d, ^3^ *J*_H–P_ = 11.8 Hz, 36H, P^*t*^ *Bu*_2_) ppm. ^**13**^ **C­{** ^**1**^ **H} NMR** (101 MHz, C_6_D_6_, 298 K): δ = 158.0 (d, *J* = 10 Hz, *o*-Ar*C*), 127.9 (m, *p*-Ar*C*), 110.7 (d, *J* = 24 Hz, *m*-Ar*C*), 105.5 (*i*-Ar*C*), 36.0 (d, *J* = 26 Hz, P^*t*^ *Bu*_2_(*C*)), 27.5 (d, *J* = 16 Hz, P^*t*^ *Bu*_2_(*C*H_3_)) ppm. ^**31**^ **P NMR** (162 MHz, C_6_D_6_, 298 K): δ = 155.5–155.0 (m) ppm. ^**31**^ **P­{** ^**1**^ **H} NMR** (162 MHz, C_6_D_6_, 298 K): δ = 155.2 (s) ppm. **HRMS (EI)** *m* **/** *z* **:** [M]^+^ calculated for [C_22_H_39_O_2_BrP_2_]^+^: 476.1603, found 476.1601.

### General Procedure for the Synthesis of Compounds **2**–**6**

Under an inert atmosphere, pentane (20 mL) was added to [^*t*Bu^POCOP]Br (1.00 g, 2.09 mmol) and ^*n*^BuLi (2.5 M in hexanes, 0.92 mL, 2.30 mmol) was added dropwise to the solution at room temperature. After 2 h of stirring, the [^*t*Bu^POCOP]­Li mixture was added dropwise to a solution (Et_2_O or THF, see SI) of the corresponding group 13 reagent (AlCl_3_, GaCl_3_, InCl_3_, AlMe_2_Cl, AlH_3_·NMe_2_Et) at room temperature and was stirred for 12 h. The volatiles were subsequently removed under reduced pressure. See SI for further details on individual workups. Crystals suitable for X-ray diffraction of complexes **2**–**6** were obtained by slow evaporation of concentrated benzene solutions in a glovebox. Further details on purification of each complex can be found in section 3 of the SI.

### Synthesis of Compound **7**

Using the general procedure above, an Et_2_O solution (10 mL) of sublimed AlCl_3_ (335 mg, 2.51 mmol) and AlH_3_·NMe_2_Et (0.5 M in toluene, 5.02 mL, 2.51 mmol) were used for the synthesis of **7**. After the addition of [^*t*Bu^POCOP]Li to the AlCl_3_ solution, the reaction was stirred for 1 h, followed by the addition of AlH_3_·NMe_2_Et. The reaction was stirred for a further 48 h. Precipitation was observed and was separated by canula filtration. Volatiles of the filtrate fraction were removed under reduced pressure. Solids were dissolved in C_6_H_6_ (3 mL) to slowly evaporate in the glovebox in a small 7 mL vial to give colorless crystals as compound **7** (350 mg, 0.76 mmol, *ca*. 36%). Note: Compounds **3** and **6** also appear in the NMR spectra of the product mixture. We propose that halogen/hydride exchange takes place in solution leading to complex mixtures. See SI for spectroscopic assignment.

### Synthesis of Compound **8**

[^*t*Bu^POCOP]­AlH_2_ (50 mg, 0.11 mmol) was dissolved in 0.6 mL of *d*_6_-benzene and added to a J Young’s NMR tube. DCC (34 mg, 0.165 mmol) was added and the sample, sealed and heated at 50 °C for 16 h. On completion, the solvent was allowed to evaporate in a glovebox resulting in a colorless oil. This oil was dissolved in ∼0.3 mL of pentane and placed in a freezer at −30 °C, resulting in large colorless crystals after 2 days. The supernatant was removed and the crystals dried under vacuum yielding pure **8**, 12 mg (0.014 mmol, 13%). ^**1**^ **H NMR** (500 MHz, C_6_D_6_, 298 K): δ = 7.90–7.83 (m, 3H, *m-*Ar*H*/NC­(*H*)­N), 7.16–7.12 (m, 1H, *p-*Ar*H*), 3.33 (br. s, 2H, Cy­(C*H*)), 3.07 (br. s, 2H, Cy­(C*H*)), 2.30 (br. s, 2H, Cy­(C*H*_2_)), 2.02 (br. s, 2H, Cy­(C*H*_2_)), 2.09–1.51 (m, 18H, Cy­(C*H*_2_)), 1.29–1.16 (m, 54H, Cy­(C*H*_2_)/ P^*t*^ *Bu*_2_) ppm. ^**13**^ **C­{** ^**1**^ **H} NMR** (126 MHz, C_6_D_6_, 298 K): δ = 167.6 (d, *J* = 11 Hz, *o*-Ar*C*), 162.0 (N*C*N), 129.69 (*i*-Ar*C*), 127.5 (t, J= 3 Hz, *p*-Ar*C*), 109.8 (d, *J* = 36 Hz, *m*-Ar*C*), 56.7 (br., N*C*H), 56.0 (br., Cy­(*C*H)), 37.8 (br., Cy­(*C*H_2_)), 36.5 (br., Cy­(*C*H_2_)), 35.2 (br., Cy­(*C*H_2_)/P^*t*^ *Bu*_2_(*C*)), 33.0 (br., Cy­(*C*H_2_)), 28.3 (d, *J* = 17 Hz, P^*t*^ *Bu*_2_(*C*H_3_)), 26.7–26.1 (br. m, Cy­(*C*H_2_)) ppm. ^**31**^ **P­{** ^**1**^ **H} NMR** (202 MHz, C_6_D_6_, 298 K): δ = 140.8 (s) ppm. **LRMS (ESI)** *m* **/** *z* **:** [M–AlCy_2_N_2_C]^+^ calculated for [C_35_H_63_N_2_O_2_P_2_]^+^: 605.44, found 605.44.

### CO_2_ Activation by **6**

[^*t*Bu^POCOP]­AlH_2_ (50 mg, 0.11 mmol) was dissolved in 0.7 mL of *d*_6_-benzene and added to a J Young’s NMR tube. The solution was freeze–pump–thawed three times. CO_2_ (1 bar) was added while the tube was agitated. The tube was then closed and inverted. This CO_2_ addition cycle was repeated six times. The sample was then analyzed by NMR spectroscopy immediately. Assignments can be found in the SI, Section 4.

## Supplementary Material



## Data Availability

Information about the data that underpins the results presented in this article can be found in the Cardiff University data catalogue at DOI: 10.17035/cardiff.30390859. This repository contains spectroscopic data in their raw (NMR, X-ray) and processed forms (IR, mass spectrometry, elemental analysis). The DFT calculation files that underpin the results presented in this article can be found on DOI: 10.6084/m9.figshare.28485857.

## Notes

Experimental details, materials and methods, HRMS spectra, IR spectra, NMR spectra, DFT calculations, and crystal data (PDF)

## Abbreviations

^R^POCOP: 2,6-(OPR_2_)­C_6_H_3_
DFT: density functional theory
HOMO: highest occupied molecular orbital
LUMO: lowest unoccupied molecular orbital
