# Supplementary material for: Synthesis and Structure of Group 13 POCOP Complexes
Source: Inorg Chem. 2026 Apr 3;65(15):8289–300. doi: 10.1021/acs.inorgchem.5c05278 (PMC13100951; doi:10.1021/acs.inorgchem.5c05278)
Supplement: Supplementary file 1 [file ic5c05278_si_001.pdf]

Supporting Information

for

Synthesis and Structure of Group 13 POCOP Complexes

Sanjukta Pahar,[†a] Taylor Wilde,[†a] Kushagra Agrawal,[a] Nathan T. Coles,*[b] Benson M. Kariuki,[b] Deborah L. Kays,[b] Andrew J. Logsdail,[a] Thomas Wirth,[b] Emma Richards,[a] and Rebecca L. Melen*[a]

[a]*Catalysis Institute, Cardiff University, Translational Research Hub, Maindy Road, Cathays, Cardiff, CF24 4HQ, Wales, United Kingdom*

[b]*School of Chemistry, Cardiff University, Main Building, Park Place, Cardiff, CF10 3AT, Cymru/Wales, United Kingdom.*

Authors to whom correspondence should be addressed:

E-mail: ColesN4@cardiff.ac.uk; MelenR@cardiff.ac.uk

Table of Contents

1. General experimental information 3
2. Synthesis and characterization of [^{tBu}POCOP]Li and [^{tBu}POCOP]H 4
3. Synthesis and characterization of compounds **1**–**8** 5
4. *In situ* reactivity of complex **6** 16
   4.1 Reactivity with benzophenone 16
   4.2 Reactivity with CO_2 16
5. NMR and mass spectroscopic data 17
6. NMR spectra from *in situ* reactions of **6** 92
   6.1 Reactivity with benzophenone 92
   6.2 Reactivity with CO_2 94
7. Crystallographic data for the structural analysis of compounds **1**–**8** and [^{tBu}POCOP]Al($OCHPh_2$)$_2$ 97
8. Computational methodology 111
9. References 116

1. General experimental information

All reactions, characterizations and manipulations were carried out utilizing standard Schlenk techniques under an inert N_2 atmosphere and with an oil pump to supply vacuum. A glovebox (MBraun) with an atmosphere of dinitrogen was used for product isolation, crystallizations and preparations for reactions or analysis. Clean, oven-dried glassware would be cycled with N_2 before use. Filtration mediums were oven-dried at 120 °C for 24 h prior to use. All solvents used were either dispensed from the solvent purification system (MB SPS-800) or manually distilled and/or dried and degassed. All dry solvents were stored under a nitrogen atmosphere over dried 3 Å molecular sieves. $AlCl_3$ was purified by sublimation before use. NMR spectroscopy solvents, benzene-d_6 (C_6D_6) was dried over sodium metal and purified by distillation prior to use, and toluene-d_8 was dried over 3 Å molecular sieves. Remaining chemicals and reagents not already mentioned were commercially purchased from suppliers with no further purification. ^{1}H, ^{13}C{^{1}H}, ^{31}P and ^{31}P{^{1}H} NMR spectra were recorded on Bruker Avance II 400 or Bruker Avance 500 spectrometers. Chemical shifts are expressed as parts per million (ppm, δ) downfield of tetramethyl silane (TMS) and are referenced to C_6D_6 (^{1}H: 7.16 ppm/^{13}C: 128.06 ppm) or toluene-d_8 (^{1}H: 2.08, 6.97, 7.01, 7.09 ppm/^{13}C: 20.43, 125.13, 127.96, 128.87, 137.48 ppm) as internal standards. The description of signals includes s = singlet, d = doublet, t = triplet, q = quartet, m = multiplet, br. = broad and app. = apparent. All coupling constants are absolute values and are expressed in Hertz (Hz). Mass spectra were measured on a Thermo Scientific Exactive GC-MS spectrometer and ions were generated by electron ionization (EI) or a Waters XEVO-G2XSQTOF with electrospray ionisation (ESI) or atomospheric pressure chemical ionisation (APCI). Elemental analysis (EA) samples were weighed using Mettler Toledo high precision scale and analyzed using ThermoFlash 2000 at London Metropolitan University's EA service. Acetanilide was used as standard for EA

within ±0.23% for carbon, ±0.07% for hydrogen and ±0.09% for nitrogen of the calculated value. Yields are given as isolated yields.

2. Synthesis and characterization of [^{tBu}POCOP]Li and [^{tBu}POCOP]H

Synthesis of [^{tBu}POCOP]Li

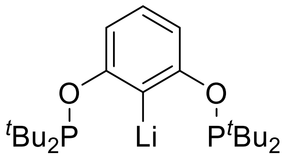

1 (1.98 g, 4.15 mmol) was dissolved in pentane (50 mL), and ^{n}BuLi (1.83 mL, 4.56 mmol, 2.5 M in hexanes) was added dropwise at room temperature; the clear solution turned pale yellow. The reaction was left to stir at room temperature for 2 h. The solvent was evaporated under reduced pressure, and the solids were further dried under high vacuum. The off-white solids were extracted into pentane (3 × 20 mL) and the volume was reduced before being put at –30 °C for 16 h. Several recrystallization attempts were made to obtain pure material and the batches were combined. [^{tBu}POCOP]Li was isolated as colorless crystals (420 mg, 25%, 1.04 mmol).

^{1}H NMR (400 MHz, C_6D_6, 298 K): δ = 7.25–7.17 (m, 3H, *m*-Ar*H*/*p*-Ar*H*), 6.96 (t, $^3J_{H–H}$ = 8.3 Hz, 1H, *p*-Ar*H*), 1.16 (d, $^3J_{H–P}$ = 11.6 Hz, 36H, P^t*Bu*$_2$) ppm.

^{13}C{^{1}H} NMR (151 MHz, C_6D_6, 298 K): δ = 168.93 (d, $^2J_{C–P}$ = 7 Hz, *o*-Ar*C*), 146.8 (*i*-Ar*C*), 128.9 (*p*-Ar*C*), 111.22 (d, $^2J_{C–P}$ = 14 Hz, *m*-Ar*C*), 35.63 (d, $^1J_{C–P}$ = 24 Hz, P^t*Bu*$_2$(*C*)), 28.3 (d, $^2J_{C–P}$ = 14 Hz, P^t*Bu*$_2$(*C*H$_3$)) ppm.

^{31}P NMR (162 MHz, C_6D_6, 243 K): δ = 142.9 (br. s) ppm.

^{31}P{^{1}H} NMR (162 MHz, C_6D_6, 298 K): δ = 142.8 (br. s) ppm.

Synthesis of [^{tBu}POCOP]H

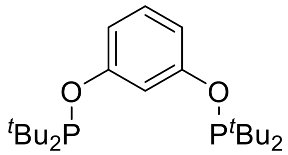

To an NMR tube containing 10 mg of [^{tBu}POCOP]Li in 0.6 mL of C_6D_6, 10 µL of wet propan-2-ol was added, resulting in a green solution. A precipitate formed and so the sample was filtered through a pipette fitted with glass fiber paper. The sample was then analyzed by NMR spectroscopy without further purification.

^{1}H NMR (400 MHz, C_6D_6, 298 K): δ = 7.50 (app. p, $^4J_{H–H}$ = 2.3 Hz, $^4J_{H–P}$ = 2.3 Hz, 1H, *i*-Ar*H*), 7.08–7.02 (m, 1H, *p*-Ar*H*), 6.98 (m, 2H, *m*-Ar*H*), 1.12 (d, $^3J_{H–P}$ = 11.6 Hz, 36H, P^t*Bu$_2$*) ppm.

^{13}C{^{1}H} NMR (101 MHz, C_6D_6, 298 K): δ = 161.52 (d, $^2J_{C–P}$ = 10 Hz, *o*-Ar*C*), 130.13 (*p*-Ar*C*), 111.90 (d, $^3J_{C–P}$ = 11 Hz, *m*-Ar*C*), 109.11 (t, $^4J_{C–P}$ = 12 Hz, *i*-Ar*C*), 35.68 (d, $^1J_{C–P}$ = 27Hz, P^t*Bu$_2$*(*C*)), 27.52 (d, $^2J_{C–P}$ = 16 Hz, P^t*Bu$_2$*(*C*H$_3$)) ppm.

^{31}P NMR (162 MHz, C_6D_6, 298 K): δ = 152.8–151.9 (m) ppm.

^{31}P{^{1}H} NMR (162 MHz, C_6D_6, 298 K): δ = 152.4 (s) ppm.

3. Synthesis and characterization of compounds 1–8

Synthesis of 1 ([^{tBu}POCOP]Br)[1]

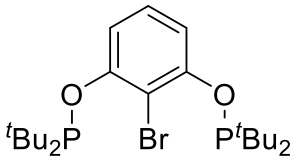

This synthetic method was used following a procedure published in literature.[1] Dry 2-methyltetrahydrofuran (20 mL) was added to 2-bromoresorcinol (1.00 g, 5.29 mmol), forming a clear solution in which ^{t}Bu$_2$PCl (2.52 mL, 13.23 mmol) and triethylamine (2.02 mL, 14.29 mmol) were added dropwise. The mixture was then stirred for 12 h at 90 °C. A white suspension was observed, and the solvent was then removed *in vacuo*. The remaining solid was extracted with

hexane (2 × 20 mL) and the pale-yellow filtrate solution was cooled to –18 °C in a freezer for 3 days. The crystalline product was separated from the solution by canula filtration, and the solids were dried under vacuum to obtain pure compound **1** (1.84 g, 3.86 mmol, 73%) as colorless crystals.

^{1}H NMR (400 MHz, C_6D_6, 298 K): δ = 7.31–7.28 (m, 2H, *m*-Ar*H*), 6.96 (t, $^{3}J_{H–H}$ = 8.3 Hz, 1H, *p*-Ar*H*), 1.15 (d, $^{3}J_{H–P}$ = 11.8 Hz, 36H, P^{t}*Bu*$_2$) ppm.

^{13}C{^{1}H} NMR (101 MHz, C_6D_6, 298 K): δ = 158.0 (d, $^{2}J_{C–P}$ = 10 Hz, *o*-Ar*C*), 127.9 (m, *p*-Ar*C*), 110.7 (d, $^{3}J_{C–P}$ = 24 Hz, *m*-Ar*C*), 105.5 (*i*-Ar*C*), 36.0 (d, $^{1}J_{C–P}$ = 26 Hz, P^{t}*Bu*$_2$(*C*)), 27.5 (d, $^{2}J_{C–P}$ = 16 Hz, P^{t}*Bu*$_2$(*C*H$_3$)) ppm.

^{31}P NMR (162 MHz, C_6D_6, 298 K): δ = 155.5–155.0 (m) ppm.

^{31}P{^{1}H} NMR (162 MHz, C_6D_6, 298 K): δ **=** 155.2 (s) ppm.

HRMS (EI) m/z: $[M]^+$ calculated for $[C_{22}H_{39}O_2BrP_2]^+$: 476.1603, found 476.1601.

The above values are in accordance with literature.[1]

General procedure: Under an inert atmosphere, pentane (20 mL) was added to [^{tBu}POCOP]Br (1.00 g, 2.09 mmol) and ^{n}BuLi (2.5 M in hexanes, 0.92 mL, 2.30 mmol) was added dropwise to the solution at room temperature. After 2 h of stirring, the [^{tBu}POCOP]Li mixture was added dropwise to a solution of the corresponding group 13 reagent ($AlCl_3$, $GaCl_3$, $InCl_3$, $AlMe_2Cl$, $AlH_3{\cdot}NMe_2Et$) at room temperature and was stirred for 12 h. The volatiles were subsequently removed under reduced pressure. Workup for individual species given below.

Synthesis of 2 ([^{tBu}POCOP]AlCl$_2$)

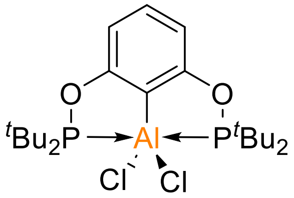

Using the *general procedure*, a THF solution (10 mL) of sublimed $AlCl_3$ (335 mg, 2.51 mmol) was used for the synthesis of compound **2**. On completion of the reaction, solids were extracted with toluene (2 × 15 mL), filtered, and the filtrate was reduced under vacuum to ~5 mL. Pentane (5 mL) was added to the concentrated solution and placed in the freezer at –30 °C for 16 h. The supernatant was removed and the solids were dried under vacuum (140 mg). The supernatant was concentrated and separated from the precipitate a further 3 times. All fractions containing the desired product (observed by $^{31}P\{^1H\}$ NMR spectroscopy) were combined and recrystallized by slow evaporation from concentrated C_6H_6 solutions in 7 mL vials in the glovebox, yielding large colorless crystals of compound **2** (410 mg, 0.83 mmol, 40%).

^{1}H NMR (400 MHz, C$_6$D$_6$, 298 K): δ = 7.04 (t, $^3J_{H-H}$ = 8.0 Hz, 1H, *p*-Ar*H*), 6.69 (d, $^3J_{H-H}$ = 8.6 Hz, 2H, *m*-Ar*H*), 1.29 (d,$^3J_{H-P}$ = 13.3 Hz, 36H, P^t*Bu$_2$*) ppm.

^{13}C{^{1}H} NMR (101 MHz, C$_6$D$_6$, 298 K): δ = 166.1 (t, *J* = 5 Hz, *o*-Ar*C*), 133.56 (*p*-Ar*C*), 117.8 (**i*-Ar*C*), 111.0 (app. t, *J* = 3 Hz, *m*-Ar*C*), 36.5 (P^t*Bu$_2$* (*C*)), 27.5–27.4 (app. m, P^t*Bu$_2$* (*C*H$_3$)) ppm.

**ipso carbon located via HMBC.*

^{31}P NMR (162 MHz, C$_6$D$_6$, 298 K): δ = 96.9 (m) ppm.

^{31}P{^{1}H} NMR (162 MHz, C$_6$D$_6$, 298 K): δ = 96.9 (s) ppm.

EA: Calcd (%) C: 53.34, H: 7.94, N: 0.00. Found (%), C: 53.32, H: 8.05, N: 0.00.

Synthesis of 3 & 3'([^{tBu}POCOP]GaCl$_2$)

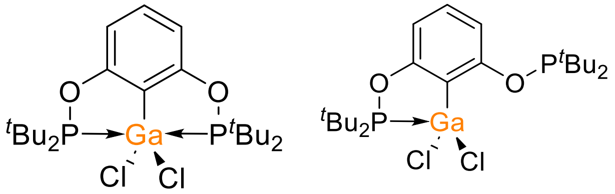

Using the *general procedure*, an Et_2O solution (5 mL) of $GaCl_3$ (405 mg, 2.30 mmol) was used for the synthesis of compound **3/3'**. On completion of the reaction, the crude product was extracted *via* a filter cannula, and the residual solids extracted with toluene (2 × 15 mL). The filtrate was placed in the freezer (–30 °C) yielding a white precipitate after 16 h. The solid was collected by filtration, and the filtrate was concentrated *in vacuo* before placing back in the freezer (–30 °C). This process was repeated until the precipitate did not contain any desired product by $^{31}P\{^1H\}$ NMR spectroscopy. The fractions were combined and recrystallized by slow evaporation from concentrated C_6H_6 solutions in 7 mL vials in the glovebox, yielding colorless crystals of **3/3'** (667 mg, 1.24 mmol, 59%).

3

^{1}H NMR (400 MHz, Tol-*d$_8$*, 212 K): δ = 6.94 (t, $^3J_{H–H}$ = 7.9 Hz, 1H, *p*-Ar*H*), 6.71 (d, $^3J_{H–H}$ = 7.9Hz, 2H, *m*-Ar*H*), 1.25 (d, $^3J_{H–P}$ = 12.6 Hz, 36H, P^t*Bu*$_2$) ppm.

^{13}C{^{1}H} NMR (101 MHz, C$_6$D$_6$, 298 K): δ = 163.9 (*o*-Ar*C*), 133.3 (t, *J* = 2 Hz, *p*-Ar*C*), 111.2 (*m*-Ar*C*), 36.9 (d, $^1J_{C–P}$ = 8 Hz, P^t*Bu*$_2$(*C*)), 27.3 (d, $^2J_{C–P}$ = 9 Hz, P^t*Bu*$_2$(*C*H$_3$)) ppm. *Unable to locate ipso carbon environment via HMBC.*

^{31}P{^{1}H} NMR (162 MHz, Tol-*d$_8$*, 212 K): δ = 95.4 (s) ppm.

HRMS (APCI) m/z: $[M+H]^+$ calculated for $[C_{22}H_{40}Cl_2GaO_2P_2]^+$: 537.1136, found 537.1147.

EA: Calcd (%) C: 49.10, H: 7.31, N: 0.00. Found (%), C: 49.21, H: 6.74, N: 0.00.

3'

^{1}H NMR (400 MHz, Tol-d_8, 213 K): δ = 7.60 (m, 1H, Ar*H*), 7.09 (m, Ar*H*), 1.20 (d, $^3J_{H–P}$ = 11.8 Hz, 18H, P^t*Bu*$_2$ unbound), 0.93 (d, $^3J_{H–P}$ = 15.7 Hz, 18H, P^t*Bu*$_2$ coordinated) ppm. Accurate determination of Ar*H* integrals not possible due to substantial overlap with solvent peaks.

^{31}P{^{1}H} NMR (162 MHz, Tol-d_8, 212 K): δ = 146.9 (s, unbound P), 94.1 (s, coordinated P) ppm.

Synthesis of 4 ([^{tBu}POCOP]InCl$_2$)

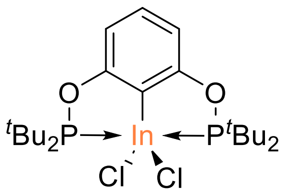

Using the *general procedure*, an Et_2O/THF mix (1:1, 20 mL) of $InCl_3$ (553 mg, 2.51 mmol) was used for the synthesis of compound **4**. On completion, volatiles were removed under reduced pressure. The crude solid was recrystallized from a concentrated C_6H_6 (3 mL) solution by slow evaporation in the glovebox in a 7 mL vial to yield colorless crystals of compound **4** (381 mg, 0.65 mmol, 31%). Note: We consistently obtained a 7–15% impurity in the batches of **4** that were found to be [^{tBu}POCOP]InClBr.

^{1}H NMR (400 MHz, C_6D_6, 298 K): δ = 7.04 (t, $^3J_{H–H}$ = 7.9 Hz, 1H, *p*-Ar*H*), 6.72 (d, $^3J_{H–H}$ = 8.0 Hz, 2H, *m*-Ar*H*), 1.21 (d, $^3J_{H–P}$ = 14.2 Hz, 36H, P^t*Bu*$_2$) ppm.

^{1}H NMR (400 MHz, Tol-d_8, 293 K): δ = 7.04 (tt, $^3J_{H–H}$ = 8.0 Hz, $^3J_{H–P}$ = 0.9 Hz, 1H, *p*-Ar*H*), 6.67 (dt, $^3J_{H–H}$ = 8.0 Hz, $^3J_{H–H}$ = 1.5 Hz, 2H, *m*-Ar*H*), 1.21 (d, $^3J_{H–P}$ = 14.2 Hz, 36H, P^t*Bu*$_2$) ppm.

^{13}C{^{1}H} NMR (101 MHz, C_6D_6, 298 K): δ = 163.3 (*o*-Ar*C*), 132.9 (*p*-Ar*C*), 117.7 (**i*-Ar*C*), 112.5 (app. t, *J* = 3 Hz, *m*-Ar*C*), 37.4–37.3 (app. m, P^t*Bu*$_2$(*C*)), 27.2–27.0 (app. m, P^t*Bu*$_2$(*C*H$_3$)) ppm. **ipso carbon located via HMBC.*

^{31}P NMR (162 MHz, C_6D_6, 298 K): δ = 93.2 (m) ppm.

^{31}P{^{1}H} NMR (162 MHz, C_6D_6, 298 K): δ = 93.2 (s) ppm.

$^{31}P\{^{1}H\}$ NMR (162 MHz, Tol-d_8, 293 K): δ = 93.1(s) ppm.

HRMS (ESI) m/z: $[M+H]^+$ calculated for $[C_{22}H_{40}Cl_2InO_2P_2]^+$: 583.0914, found 583.0928.

EA: Calcd (%) C: 45.31, H: 6.74, N: 0.00. Found (%), C: 44.68, H: 6.08, N: 0.00.

[^{tBu}POCOP]InClBr

^{1}H NMR (400 MHz, Tol-d_8, 293 K): δ = 7.02–6.99 (m, 1H, *p*-Ar*H*), 6.67–6.64 (m, 2H, *m*-Ar*H*), 1.22 (d, $^{3}J_{H-P}$ = 14.1 Hz, 18H, P^{t}*Bu*$_2$), 1.21 (d, $^{3}J_{H-P}$ = 14.2 Hz, 18H, P^{t}*Bu*$_2$) ppm.

$^{31}P\{^{1}H\}$ NMR (162 MHz, Tol-d_8, 293 K): δ = 92.1(s) ppm.

LRMS (ESI) m/z: $[M]^+$ calculated for $[C_{22}H_{40}BrClInO_2P_2]^+$: 629.04, found 629.04.

Synthesis of 5, 5' ([^{tBu}POCOP]AlMe$_2$) & [^{tBu}POCOP]Al(Me)Cl

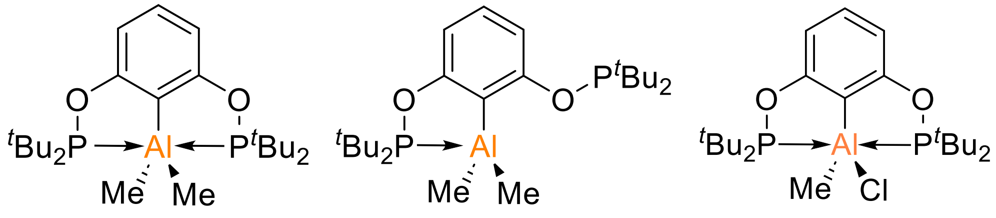

5 **5'** [^{tBu}POCOP]Al(Me)Cl

Using the *general procedure*, $AlMe_2Cl$ (0.9 M in heptanes, 2.8 mL, 2.51 mmol) was used for the synthesis of compound **5/5'**. On completion of the reaction, the crude product was extracted *via* a filter cannula, and the residual solids extracted with toluene (2 × 15 mL). The volatiles were removed and the crude product was dissolved in benzene (20 mL) in a Schlenk flask. A vigorous flow of argon gas was passed over the solution to concentrate the solution until precipitation occurred. Benzene was added until the precipitate redissolved and was transferred into the glovebox to recrystallize by slow evaporation from C_6H_6 in 7 mL vials in the glovebox, yielding colorless crystals of compound **5/5'** (253 mg, 0.56 mmol, 27%). *Note: For the synthesis of*

*compound **5/5'**, the $AlMe_2Cl$ used contains small quantities $AlMeCl_2$, which results in the formation of inseparable amounts (~14%) of the asymmetric [^{t}BuPOCOP]Al(Me)Cl compound along with the desired disubstituted methylated complex. Even through subsequent recrystallizations we were unable to completely separate the two compounds.*

5

^{1}H NMR (400 MHz, C_6D_6, 298 K): δ = 7.12 (t, $^{3}J_{H\text{–}H}$ = 7.9 Hz, 1H, *p*-Ar*H*), 6.98 (br., 2H, *m*-Ar*H*), 1.14 (d, $^{3}J_{H\text{–}P}$ = 12.3 Hz, 36H, P^{t}*Bu*$_2$), 0.09 (t, $^{3}J_{H\text{–}P}$ = 3.3 Hz, 6H, C*H*$_3$) ppm.

^{1}H NMR (400 MHz, Tol-*d*$_8$, 212 K): δ = 7.06 (t, $^{3}J_{H\text{–}H}$ = 7.9 Hz, 1H, *p*-Ar*H*), 6.85 (d, $^{3}J_{H\text{–}H}$ = 7.8 Hz, 2H, *m*-Ar*H*), 1.15 (d, $^{3}J_{H\text{–}P}$ = 11.7 Hz, 36H, P^{t}*Bu*$_2$), 0.18 (t, $^{3}J_{H\text{–}P}$ = 4.7 Hz, 6H, C*H*$_3$) ppm.

^{13}C{^{1}H} NMR (101 MHz, C_6D_6, 298 K): δ = 166.8 (*o*-Ar*C*), 131.0 (app. t, *J* = 2 Hz, *p*-Ar*C*), 124.6 (**i*-Ar*C*), 110.0 (app. d, *J* = 9 Hz, *m*-Ar*C*), 36.0 (d, $^{1}J_{C\text{–}P}$ = 10 Hz, P^{t}*Bu*$_2$(*C*)), 27.5 (d, $^{2}J_{C\text{–}P}$ = 11 Hz, P^{t}*Bu*$_2$(*C*H$_3$)), −2.7 (br., Al(*C*H$_3$)$_2$) ppm. **ipso carbon located via HMBC.*

^{31}P{^{1}H} NMR (162 MHz, Tol-*d*$_8$, 212 K): δ = 107.4 (s) ppm.

HRMS (ESI) m/z: $[M+H]^+$ calculated for $[C_{24}H_{46}AlO_2P_2]^+$: 455.2783, found 455.2803.

EA: Calcd (%) C: 63.42, H: 9.98, N: 0.00. Found (%), C: 61.74, H: 9.00, N: 0.00. *Satisfactory EA could not be obtained for this sample due to contamination of the product with inseparable amounts of [^{tBu}POCOP]Al(Me)Cl.*

5'

^{1}H NMR (400 MHz, Tol-*d*$_8$, 212 K): δ = 7.60 (dd, $^{3}J_{H\text{–}H}$ = 8.1 Hz, $^{4}J_{H\text{–}H}$ = 5.0 Hz, 1H, *m*-Ar*H*), 1.25 (d, $^{3}J_{H\text{–}P}$ = 11.8 Hz, 3H, P^{t}*Bu*$_2$ unbound), 0.94 (d, $^{3}J_{H\text{–}P}$ = 13.9 Hz, 3H, P^{t}*Bu*$_2$ coordinated), 0.15 (m, 6H, C*H*$_3$) ppm. Not all signals could be located. Methyl signals overlap with **5**.

$^{31}P\{^{1}H\}$ NMR (162 MHz, Tol-d_8, 212 K): δ = 141.0 (s, unbound P), 109.5 (s, coordinated P) ppm.

[^{tBu}POCOP]Al(Me)Cl)

^{1}H NMR (400 MHz, C_6D_6, 298 K): δ = 7.07 (tt, $^{3}J_{H-H}$ = 7.9 Hz, $^{3}J_{H-P}$ = 0.9 Hz 1H, *p*-Ar*H*), 6.77 (br. d, $^{3}J_{H-H}$ = 7.8 Hz, 2H, *m*-Ar*H*), 1.30 (d, $^{3}J_{H-P}$ = 13.0 Hz, 36H, P$^{t}Bu_2$), 0.31 (t, $^{3}J_{H-P}$ = 4.3 Hz, 6H, CH_3) ppm.

^{1}H NMR (400 MHz, Tol-d_8, 212 K): δ = 7.02 (m, 1H, *p*-Ar*H*), 6.79 (d, $^{3}J_{H-H}$ = 7.9 Hz, 2H, *m*-Ar*H*), 1.30 (d, $^{3}J_{H-P}$ = 12.9 Hz, 3H, P$^{t}Bu_2$), 0.37 (t, $^{3}J_{H-P}$ = 4.5 Hz, 6H, CH_3) ppm. *p*-ArH signal is obscured by both **5** and deuterated solvent peak.

$^{31}P\{^{1}H\}$ NMR (162 MHz, C_6D_6, 298 K): δ = 102.5 (s) ppm.

$^{31}P\{^{1}H\}$ NMR (162 MHz, Tol-d_8, 212 K): δ = 100.8 (s) ppm.

Synthesis of 6 ([^{tBu}POCOP]AlH_2)

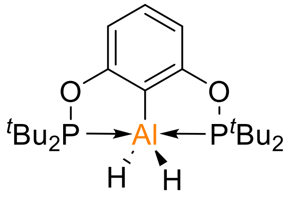

6

*Two routes were devised for the synthesis of compound **6**.*

Route A: Using the *general procedure*, $AlH_3{\cdot}NMe_2Et$ (0.5 M in toluene, 5.0 mL, 2.51 mmol) was used for the synthesis of compound **6**. After stirring for 12 hours, the crude product was extracted *via* a filter cannula. The volatiles were removed and the crude product was dissolved in benzene (15 mL) in a Schlenk flask. A vigorous flow of argon gas was passed over the solution to concentrate the solution until an oil had formed, to which pentane was added (2 mL). The solution was concentrated a second time, until crystals began to form. The sample was then transferred, washing the original crystals in with benzene, into a 7 mL vial in the glovebox and allowed to slowly evaporate, yielding colorless crystals of compound **6** (293 mg, 0.69 mmol, 33%). *Note: if too much C_6H_6 is added to*

the filtrate fraction for recrystallisation, the solution forms a gel from slow evaporation and not a crystalline material.

Route B: *The general procedure was not used for this reaction.* To a solution of [^{tBu}POCOP]$AlCl_2$ (575 mg, 1.16 mmol) in toluene (40 mL) at –78 °C, 2.05 equivalents of $AlH_3·NMe_2Et$ (0.5 M in toluene, 4.92 mL, 2.39 mmol) was added. The reaction was stirred cold for 30 min before being warmed to room temperature and stirred for a further 16 h. The volatiles were removed under reduced pressure with slight heating (30 °C). Toluene (30 mL) was added to the solids, and the reaction mixture was stirred for 10 min filtering through Celite. The volatiles were removed under vacuum with slight heating (30 °C), and the solids were extracted with hexane (2 × 20 mL). The remaining material was recrystallized from a highly concentrated toluene solution of **6** at –18 °C, affording colorless crystals of compound **6** (100 mg, 0.23 mmol, 20%).

^{1}H NMR (400 MHz, C_6D_6, 298 K): δ = 7.11 (t, $^3J_{H–H}$ = 7.9 Hz, 1H, *p*-Ar*H*), 6.80 (d, $^3J_{H–H}$ = 9.2 Hz, 2H, *m*-Ar*H*), 1.25–1.21 (m, 36H, P^t*Bu*$_2$) ppm.

^{1}H NMR (400 MHz, Tol-*d*$_8$, 203 K): δ = 7.11–7.08 (m, 1H, *p*-Ar*H*), 6.92 (d, $^3J_{H–H}$ = 7.9 Hz, 2H, *m*-Ar*H*), 5.20 (t, $^3J_{H–P}$ = 50.2 Hz, 2H, Al*H*$_2$), 1.20 (m, 36H, P^t*Bu*$_2$) ppm.

^{13}C{^{1}H} NMR (101 MHz, C_6D_6, 298 K): δ = 166.8 (t, *J* = 5 Hz, *o*-Ar*C*), 131.4 (*p*-Ar*C*), 120.8 (**i*-Ar*C*), 110.3 (app. t, *J* = 3 Hz, *m*-Ar*C*), 35.9 (app. t, *J* = 3 Hz, P^t*Bu*$_2$(*C*)), 27.2 (app. t, *J* = 4 Hz, P^t*Bu*$_2$(*C*H_3)) ppm. **ipso carbon located via HMBC.*

^{31}P NMR (162 MHz, C_6D_6, 298 K): δ = 99.3 (br. s) ppm.

^{31}P{^{1}H} NMR (162 MHz, C_6D_6, 298 K): δ = 99.4 (s) ppm.

EA: Calcd (%) C: 61.96, H: 9.69, N: 0.00. Found (%), C: 62.44, H: 9.63, N: 0.00.

Synthesis of 7 ([^{tBu}POCOP]Al(H)Cl)

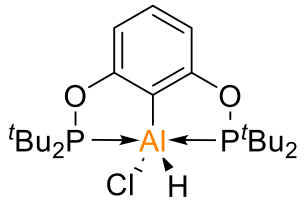

7

Using the *general procedure*, an Et_2O solution (10 mL) of sublimed $AlCl_3$ (335 mg, 2.51 mmol) and $AlH_3{\cdot}NMe_2Et$ (0.5 M in toluene, 5.02 mL, 2.51 mmol) were used for the synthesis of **7**. After the addition of [^{tBu}POCOP]Li to the $AlCl_3$ solution, the reaction was stirred for 1 h, followed by the addition of $AlH_3{\cdot}NMe_2Et$. The reaction was stirred for a further 48 h. The solution was separated from the precipitate *via* cannula filtration and the solvent was then removed under reduced pressure. The crude product was dissolved in C_6H_6 (3 mL) and recrystallized by slow evaporation in a glovebox in a 7 mL vial to give colorless crystals of compound **7** (350 mg, 0.76 mmol, *ca.* 36%). *Note: When dissolving a large quantity of crystalline material both **3** and **6** can be observed by NMR spectroscopy. We propose that halogen/hydride exchange takes place in solution leading to complex mixtures.*

^{1}H NMR (400 MHz, C_6D_6, 298 K): δ = 7.10–7.06 (m, 1H, *p*-Ar*H*), 6.76 (d, $^3J_{H–H}$ = 7.8 Hz, 2H, *m*-Ar*H*), 1.34–1.20 (m, P^t*Bu*$_2$) ppm.

^{13}C{^{1}H} NMR (101 MHz, C_6D_6, 298 K): δ = 166.6 (t, *J* = 5 Hz, *o*-Ar*C*, compound **7**) 132.6 (*p*-Ar*C*, compound **7**), 119.6 (**i*-Ar*C*), 110.6 (app. t, *J* = 3 Hz, *m*-Ar*C*, compound **7**), 36.2 (app. t, *J* = 2 Hz, P^t*Bu*$_2$ (*C*), compound **7**), 27.1 (app. t, *J* = 4 Hz, P^t*Bu*$_2$ (*C*H$_3$), compound **7**) ppm. **ipso carbon located via HMBC.*

^{31}P NMR (162 MHz, C_6D_6, 298 K): δ = 98.5 (m) ppm.

^{31}P{^{1}H} NMR (162 MHz, C_6D_6, 298 K): δ = 98.4 (s) ppm.

EA: Calcd (%) C: 57.33, H: 8.75, N: 0.00. Found (%), C: 58.06, H: 8.35, N: 0.00. *Note: Satisfactory EA could not be obtained for this sample due to contamination of the product with inseparable amounts of compound **3** and **6**.*

Synthesis of 8 ([^{tBu}POCOP]Al(CyNC(H)NCy)$_2$)

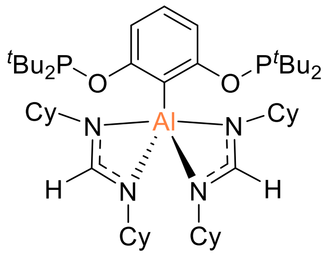

8

[^{tBu}POCOP]AlH_2 (50 mg, 0.11 mmol) was dissolved in 0.6 mL of d_6-benzene and added to a J Young's NMR tube. DCC (34 mg, 0.165 mmol) was added and the sample, sealed and heated at 50 °C for 16 h. On completion, the solvent was allowed to evaporate in a glovebox resulting in a colorless oil. This oil was dissolved in ~ 0.3 mL of pentane and placed in a freezer at −30 °C, resulting in large colorless crystals after 2 days. The supernatant was removed and the crystals dried under vacuum yielding pure **8**, 12 mg (0.014 mmol, 13 %).

^{1}H NMR (500 MHz, C_6D_6, 298 K): δ = 7.90–7.83 (m, 3H, *m*-Ar*H*/NC(*H*)N), 7.16–7.12 (m, 1H, *p*-Ar*H*), 3.33 (br. s, 2H, Cy(C*H*)), 3.07 (br. s, 2H, Cy(C*H*)), 2.30 (br. s, 2H, Cy(CH_2)), 2.02 (br. s, 2H, Cy(CH_2)), 2.09–1.51 (m, 18H, Cy(CH_2)), 1.29–1.16 (m, 54H, Cy(CH_2)/P^t*Bu*$_2$) ppm.

^{13}C{^{1}H} NMR (126 MHz, C_6D_6, 298 K): δ = 167.6 (d, $^2J_{C\text{–}P}$ = 11 Hz, *o*-Ar*C*), 162.0 (N*C*N), 129.69 (*i*-Ar*C*), 127.5 (t, $^4J_{C\text{–}P}$ = 3 Hz, *p*-Ar*C*), 109.8 (d, $^3J_{C\text{–}P}$ = 36 Hz, *m*-Ar*C*), 56.7 (br., N*C*H), 56.0 (br., Cy(*C*H)), 37.8 (br., Cy(*C*H$_2$)), 36.5 (br., Cy(*C*H$_2$)), 35.2 (br., Cy(*C*H$_2$)/P^t*Bu*$_2$(*C*)), 33.0 (br., Cy(*C*H$_2$)), 28.3 (d, $^2J_{C\text{–}P}$ = 17 Hz, P^t*Bu*$_2$(*C*H$_3$)), 26.7–26.1 (br. m, Cy(*C*H$_2$)) ppm.

^{31}P{^{1}H} NMR (202 MHz, C_6D_6, 298 K): δ = 140.8 (s) ppm.

LRMS (ESI) m/z: [M−$AlCy_2N_2C$]$^+$ calculated for [$C_{35}H_{63}N_2O_2P_2$]$^+$: 605.44, found 605.44.

4. *In situ* reactivity of complex 6

4.1 Reactivity with benzophenone

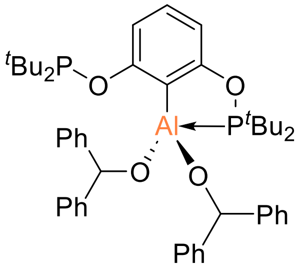

[^{tBu}POCOP]AlH_2 (50 mg, 0.11 mmol) was dissolved in 0.7 mL of d_6-benzene and added to a J Young's NMR tube. To this benzophenone was added (21 mg, 0.11 mmol; 42 mg, 0.22 mmol; 63 mg, 0.33 mmol). The sample was heated to 75 °C for 16 h and analyzed without further purification. Crystals from the reaction of 1 equivalent of benzophenone with **6** were obtained by allowing the solution to evaporate in the glovebox, dissolving in pentane (~0.2 mL) and placing in the freezer (−32 °C) for 4 days. Yield is not determined as characterization of the bulk solid after crystallization still showed complex **6**.

^{1}H NMR (600 MHz, C_6D_6, 298 K): δ = 7.52 (d, $^3J_{H-H}$ = 7.5 Hz, 8H, *o*-Ph*H*), 7.18–7.14 (m, 10H, *m*-Ph*H*/m-Ar*H*), 7.06–7.04 (m, 5H, *p*-Ph*H*/*p*-Ar*H*), 6.45 (s, 2H, OC*H*Ph$_2$), 0.98 (d,$^3J_{H-P}$ = 13.1 Hz, 36H, P^t*Bu$_2$*) ppm.

^{13}C{^{1}H} NMR (151 MHz, C_6D_6, 298 K): δ = 167.0 (t, $^3J_{C-P}$ = 4.2 Hz, *o*-Ar*C*), 148.3 (*i*-Ph*C*), 131.9 (t, $^4J_{C-P}$ = 2 Hz, *p*-Ar*C*) 128.10 (*m*-PhC), 127.7 (*o*-Ph*C*), 126.7 (*p*-Ph*C*), 110.5 (d, $^3J_{C-P}$ = 15 Hz, *m*-Ar*C*), 77.8 (O*C*HPh$_2$), 35.4 (d, $^1J_{C-P}$ = 9 Hz, P^t*Bu$_2$*(*C*)), 27.3 (d, $^2J_{C-P}$ = 11 Hz P^t*Bu$_2$*(*C*H$_3$)) ppm. Can't locate *ipso* carbon bound to Al for POCOP.

^{31}P{^{1}H} NMR (243 MHz, C_6D_6, 298 K): δ = 149.8 (s), 148.2 (s), 145.3 (s) ppm.

4.2 Reactivity with CO_2

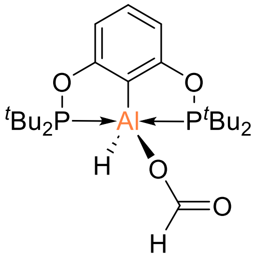

[^{tBu}POCOP]AlH_2 (5 mg, 0.01 mmol) was dissolved in 0.7 mL of d_6-benzene and added to a J Youngs NMR tube. The solution was freeze-pump-thawed three times. CO_2 (1 bar) was added while the tube was agitated. The tube was then closed and inverted. This CO_2 addition cycle was repeated six times. The sample was then analyzed by NMR spectroscopy immediately to give the suggested structure.

^{1}H NMR (400 MHz, C_6D_6, 298 K): δ = 8.62 (t, $^{4}J_{H–P}$ = 1.9 Hz, 1H, CO_2*H*), 7.07 (tt, $^{3}J_{H–H}$ = 7.9 Hz, $^{4}J_{H–P}$ = 0.9 Hz 1H, *p*-Ar*H*), 6.74 (d, $^{3}J_{H–H}$ = 7.9 Hz, 2H, *m*-Ar*H*), 1.21 (d, $^{3}J_{H–P}$ = 14.9 Hz, 18H, P^{t}*Bu*$_2$), 1.18 (d, $^{3}J_{H–P}$ = 14.9 Hz, 18H, P^{t}*Bu*$_2$) ppm.

^{13}C{^{1}H} NMR (101 MHz, C_6D_6, 298 K): δ = 166.8 (t, $^{3}J_{C–P}$ = 5 Hz, O_2*C*H), 163.2 (*o*-Ar*C*), 132.6 (*p*-Ar*C*), 110.6 (app. t, *J* = 3 Hz, *o*-Ar*C*), 36.1 (P^{t}*Bu*$_2$(*C*)), 27.1 (app t, *J* = 4 Hz, P^{t}*Bu*$_2$(*C*H_3)), 27.0 (app. t, *J* = 4 Hz, P^{t}*Bu*$_2$(*C*H_3)) ppm.

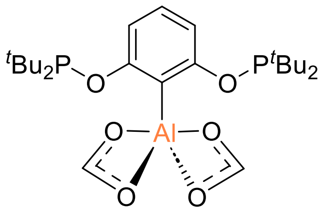

[^{tBu}POCOP]AlH_2 (50 mg, 0.11 mmol) was dissolved in 0.7 mL of d_6-benzene and added to a J Youngs NMR tube. The solution was freeze-pump-thawed three times. CO_2 (1 bar) was added while the tube was agitated. The tube was then closed and inverted. This CO_2 addition cycle was repeated six times. The sample was left to stand for 24 hours and the NMR spectra collected showed degradation of the signals assigned to the suggested mono-addition product assigned above.

^{1}H NMR (600 MHz, C_6D_6, 298 K): δ = 8.14 (br. s, 2H, Al–CO*H*), 7.61 (br. s, 2H, *m*-Ar*H*), 7.25 (br. m, 1H, *p*-Ar*H*), 1.20 (d, $^{3}J_{H–P}$ = 11.2 Hz, 18H, P^{t}*Bu*$_2$) ppm.

^{13}C{^{1}H} NMR (151 MHz, C_6D_6, 298 K): δ = 168.6 (d, $^{2}J_{C–P}$ = 10 Hz, *o*-Ar*C*), 166.2 (s, O_2*C*H), 124.4 (*p*-Ar*C*), 108.7 (d, $^{3}J_{C–P}$ = 29 Hz, *m*-Ar*C*), 35.3 (d, $^{1}J_{C–P}$ = 27 Hz, P^{t}*Bu*$_2$(*C*)), 27.5 (d, $^{2}J_{C–P}$ = 16 Hz, P^{t}*Bu*$_2$(*C*H_3)) ppm.

^{31}P{^{1}H} NMR (243 MHz, C_6D_6, 298 K): δ = 145.3 (s) ppm.

5. NMR and mass spectroscopic data

NMR SPECTRA KEY:

Undesired by-products =

Figure S1: ^{1}H NMR spectrum of [^{tBu}POCOP]Li (400 MHz, C_6D_6, 298 K).

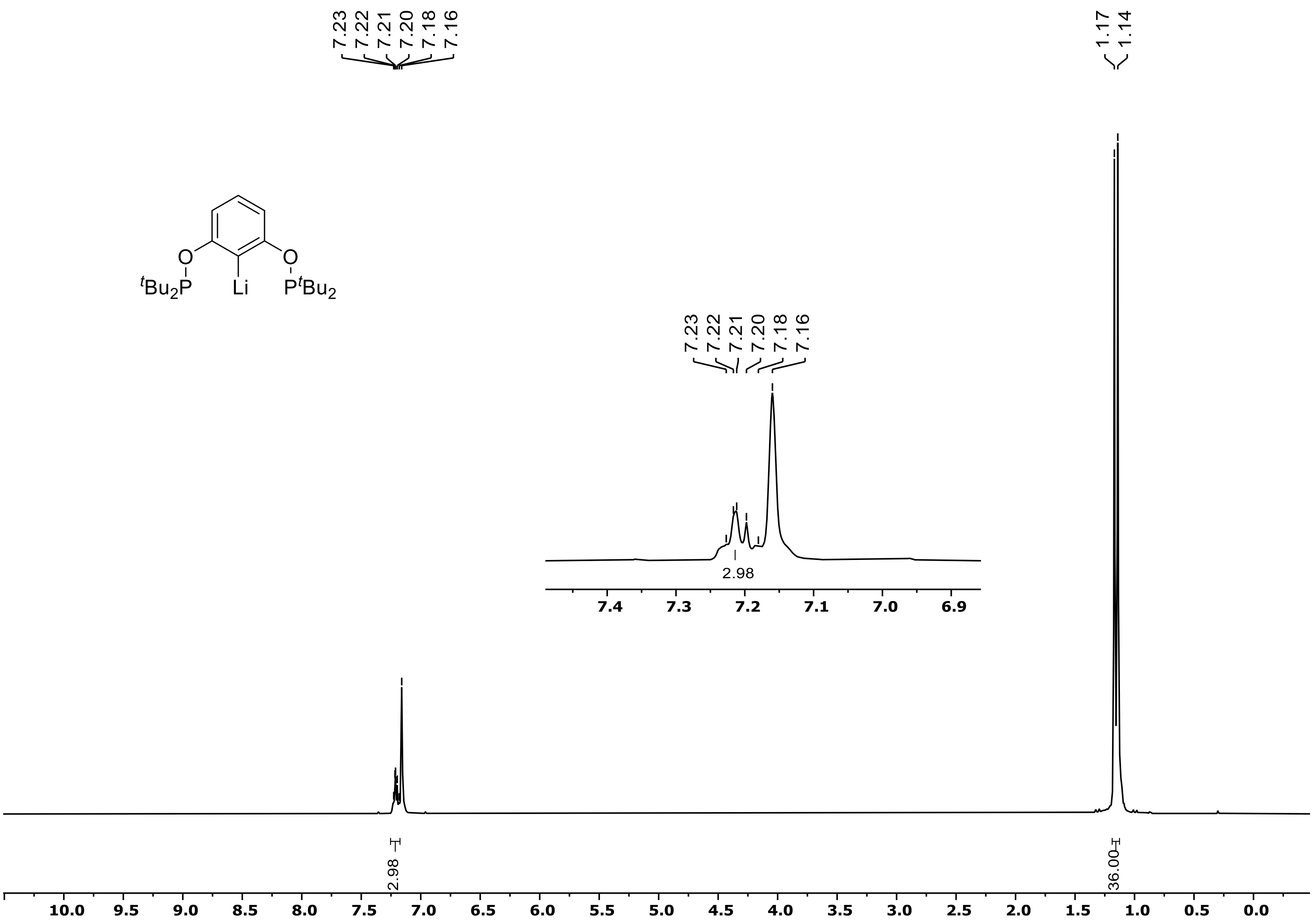

Figure S2: $^{13}C\{^{1}H\}$ NMR spectrum of [^{tBu}POCOP]Li (151 MHz, C_6D_6, 298 K).

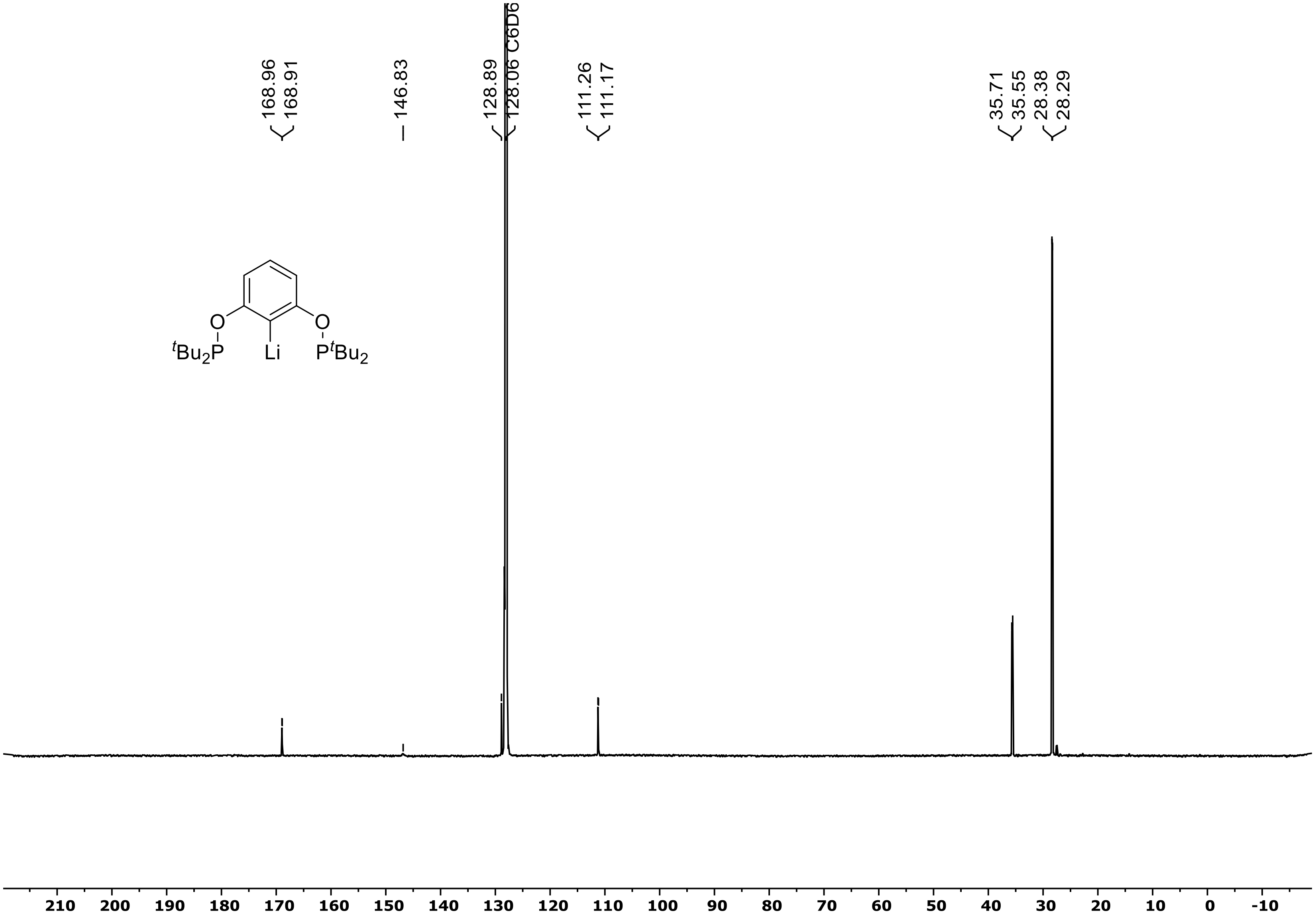

Figure S3: ^{31}P NMR spectrum of [^{tBu}POCOP]Li (243 MHz, C_6D_6, 298 K).

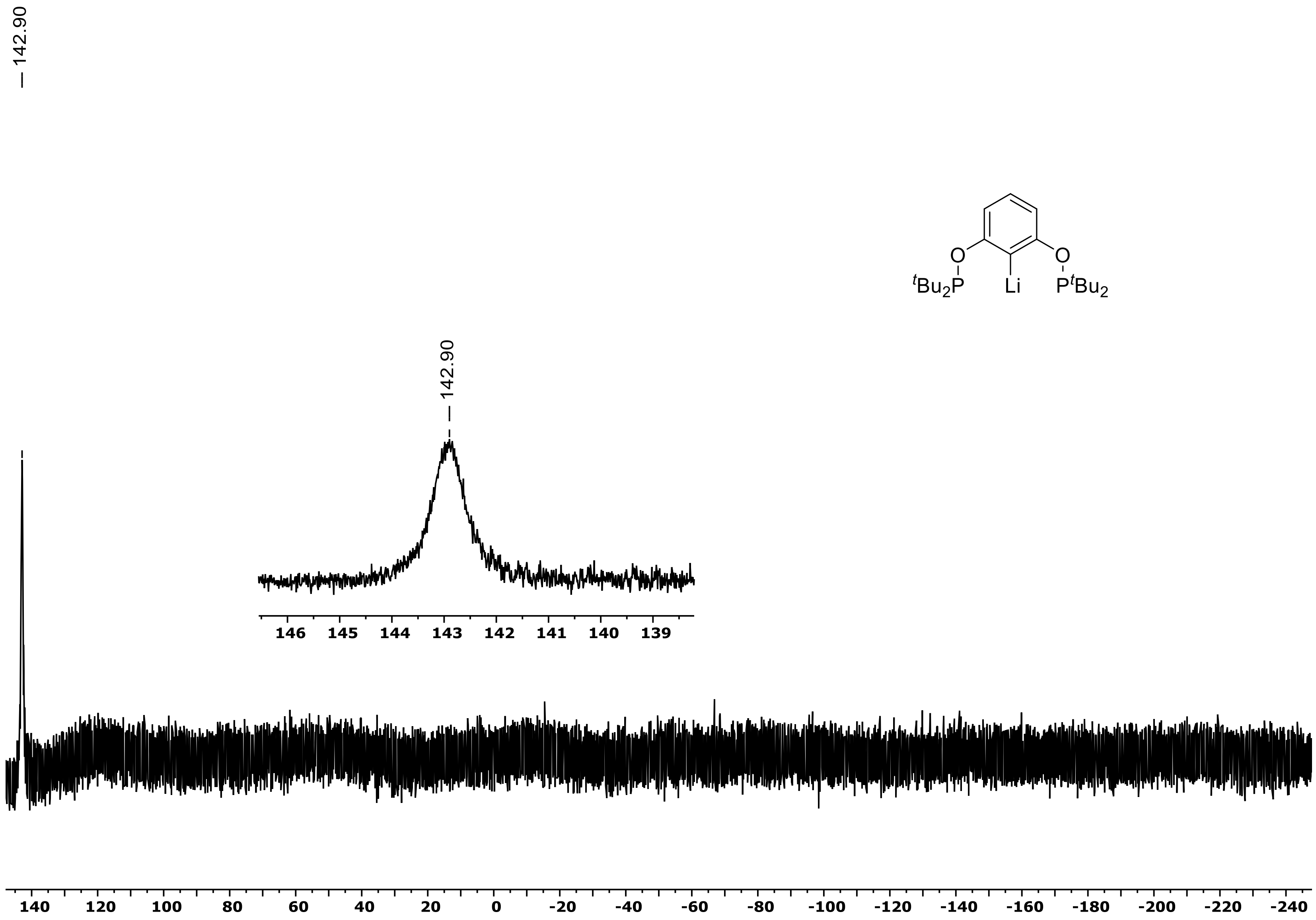

Figure S4: $^{31}P\{^{1}H\}$ NMR spectrum of [^{tBu}POCOP]Li (243 MHz, C_6D_6, 298 K).

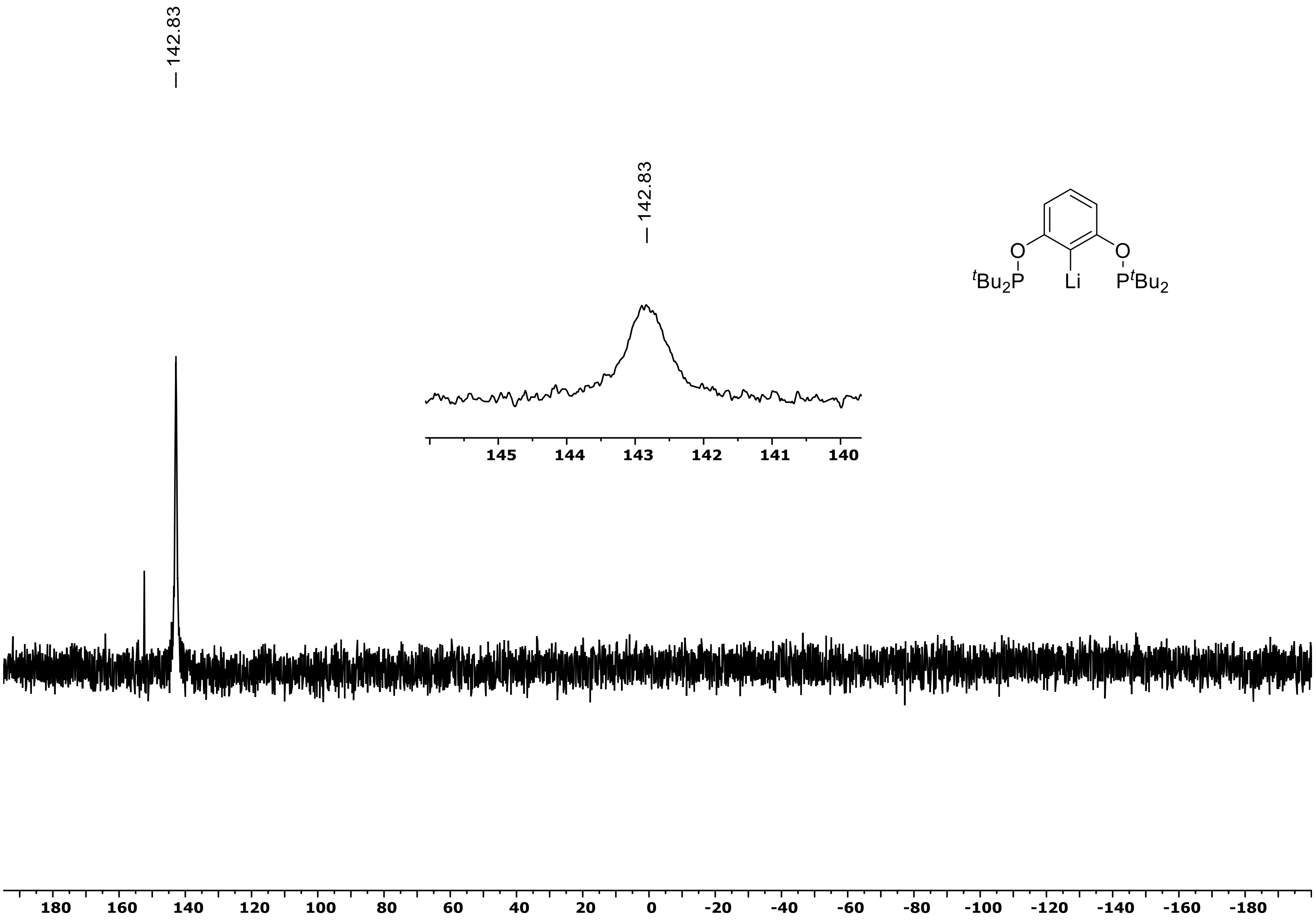

Figure S5: ^{1}H NMR spectrum of [^{tBu}POCOP]H (400 MHz, C_6D_6, 298 K).

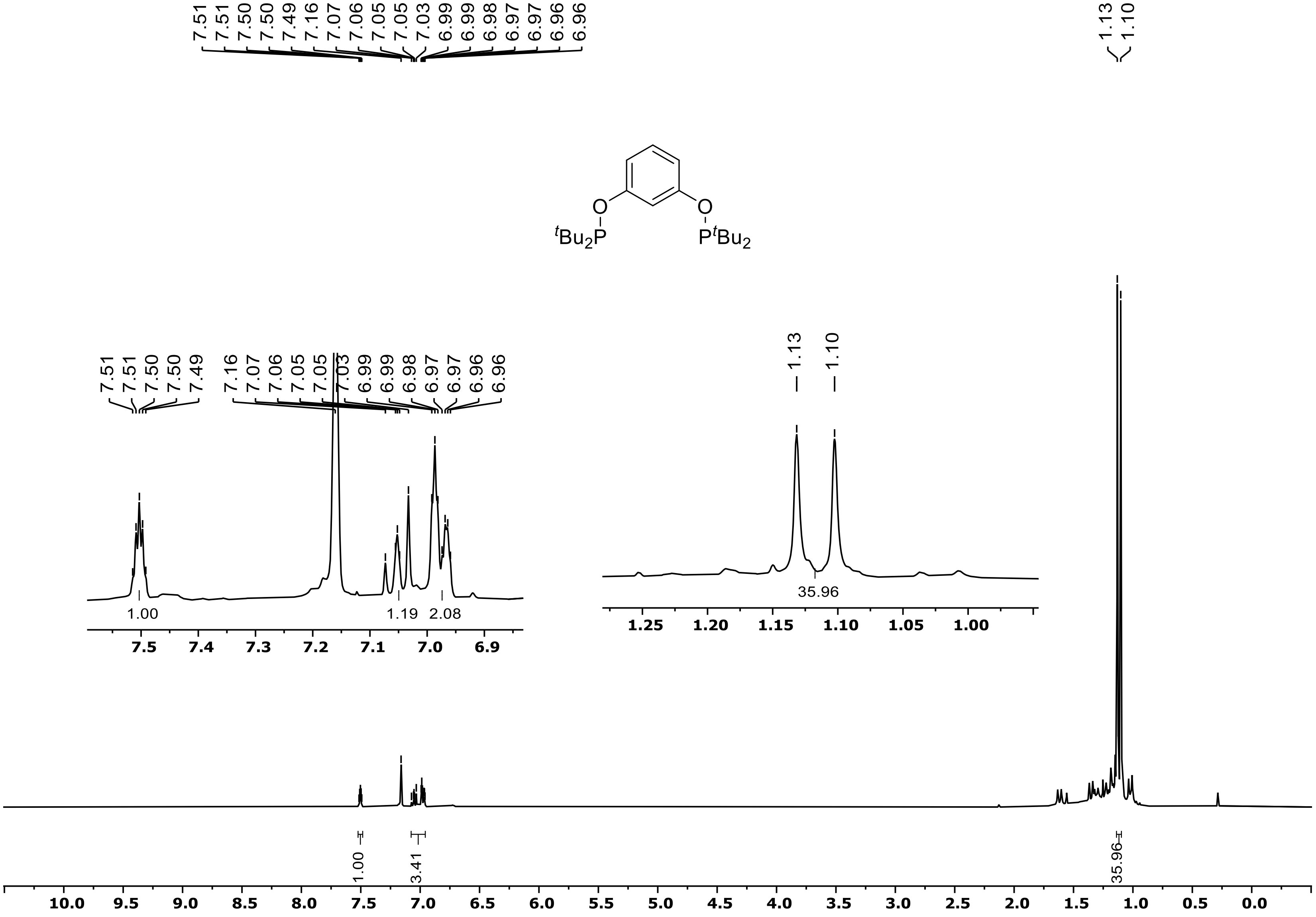

Figure S6: $^{13}C\{^1H\}$ NMR spectrum of [^{tBu}POCOP]H (101 MHz, C_6D_6, 298 K).

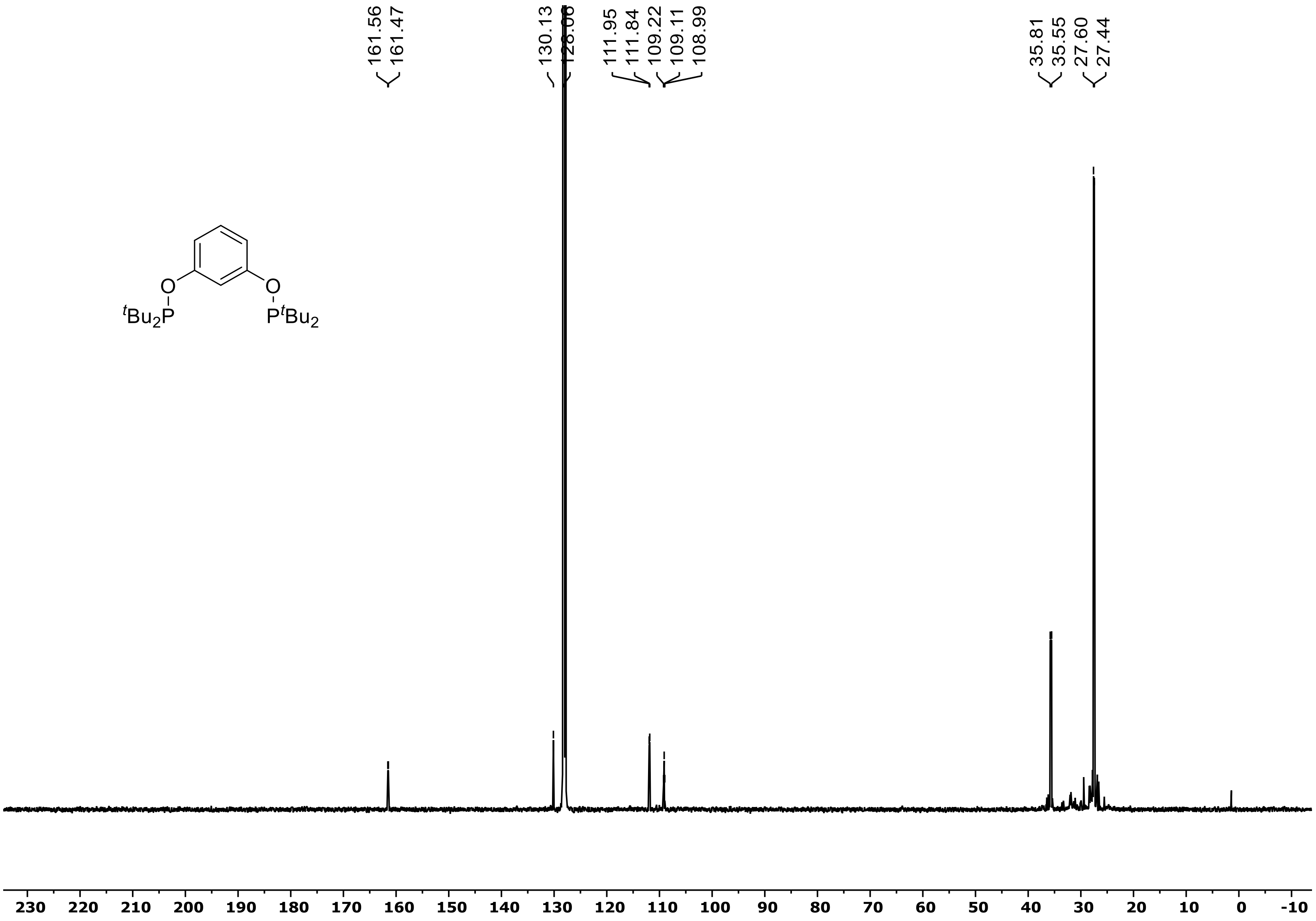

Figure S7: ^{31}P NMR spectrum of [^{tBu}POCOP]H (162 MHz, C_6D_6, 298 K).

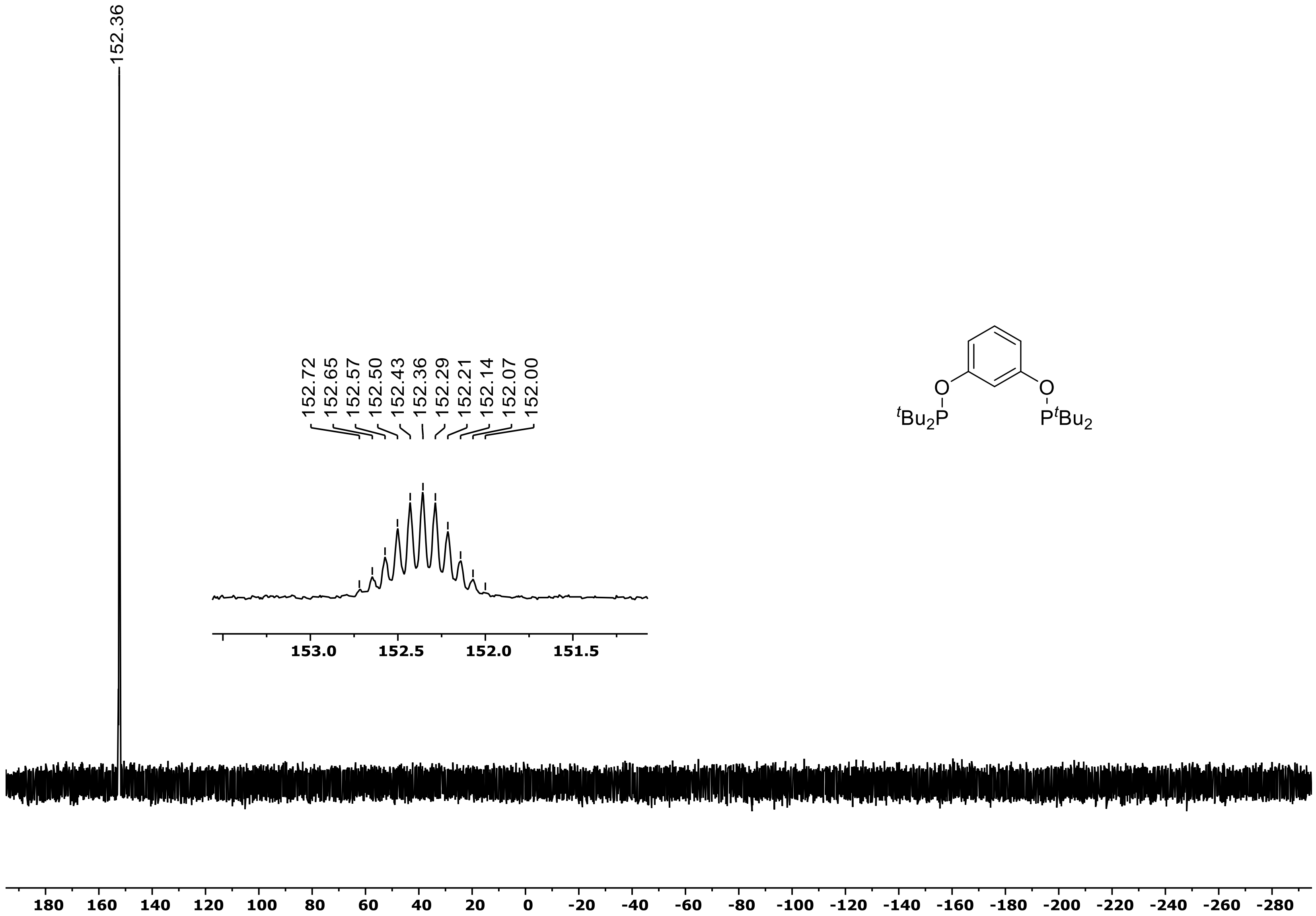

Figure S8: $^{31}P\{^{1}H\}$ NMR spectrum of [^{tBu}POCOP]H (162 MHz, C_6D_6, 298 K).

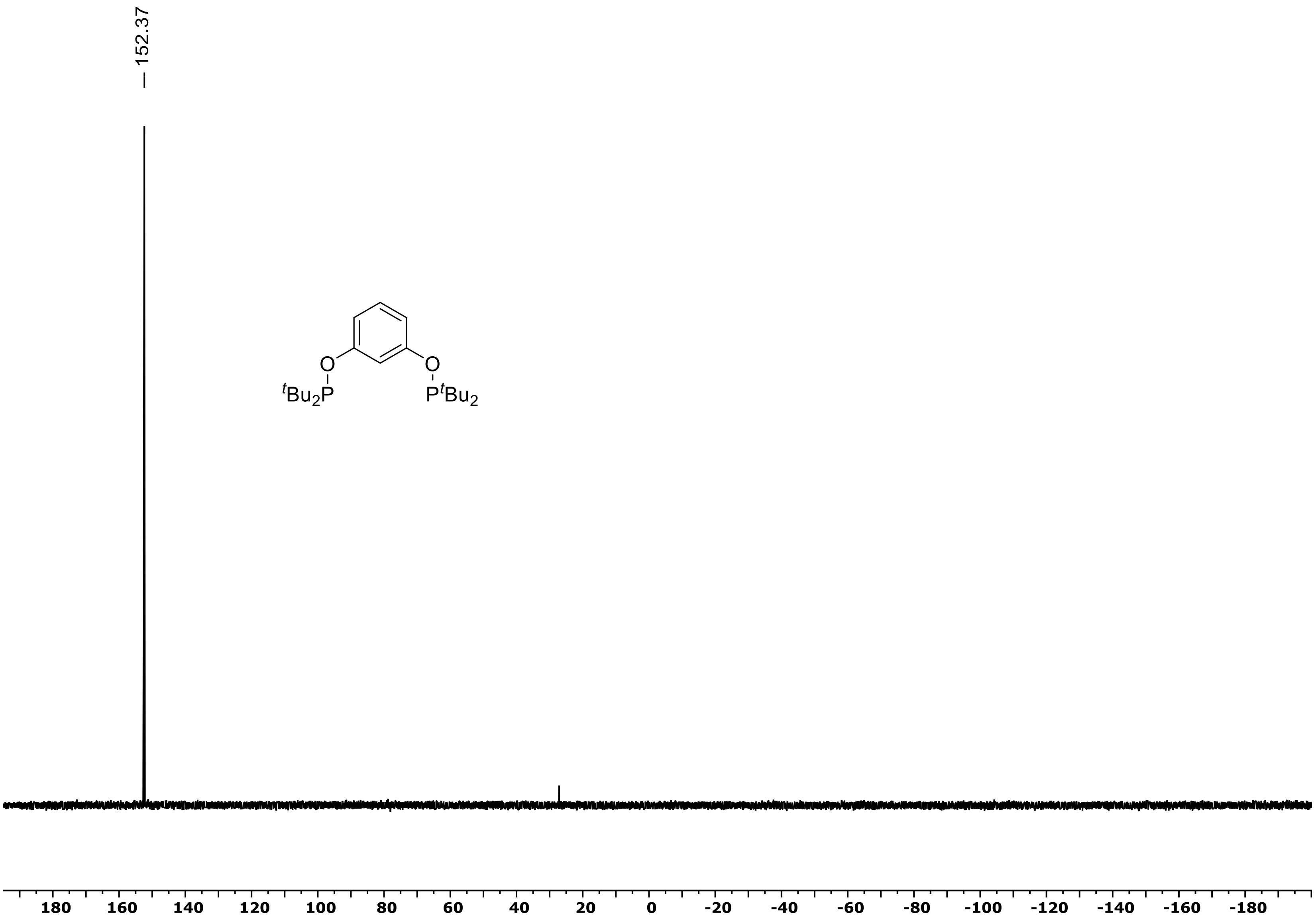

Figure S9: ^{1}H NMR spectrum of **1** (400 MHz, C_6D_6, 298 K).

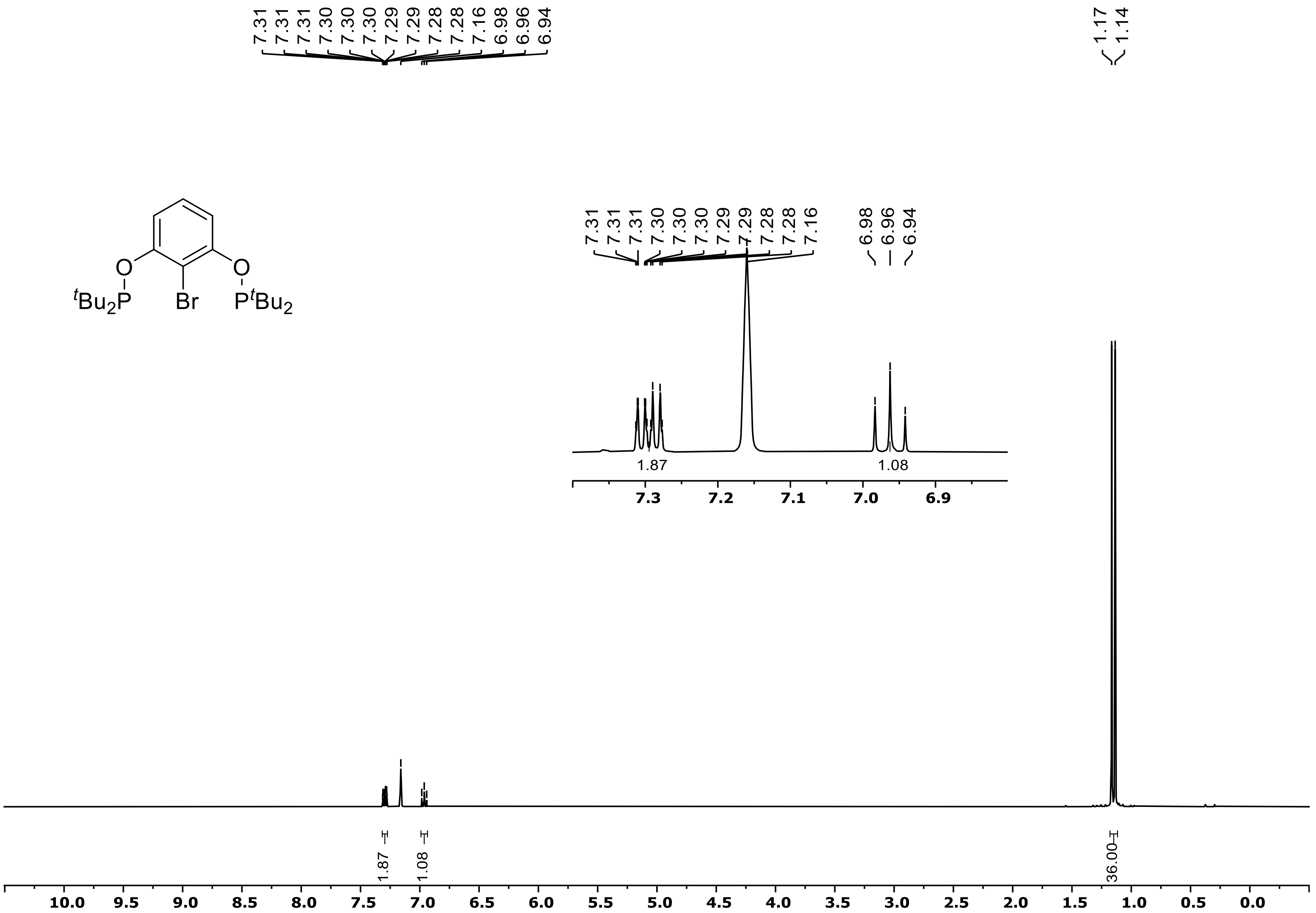

Figure S10: $^{13}C\{^1H\}$ NMR spectrum of **1** (101 MHz, C_6D_6, 298 K).

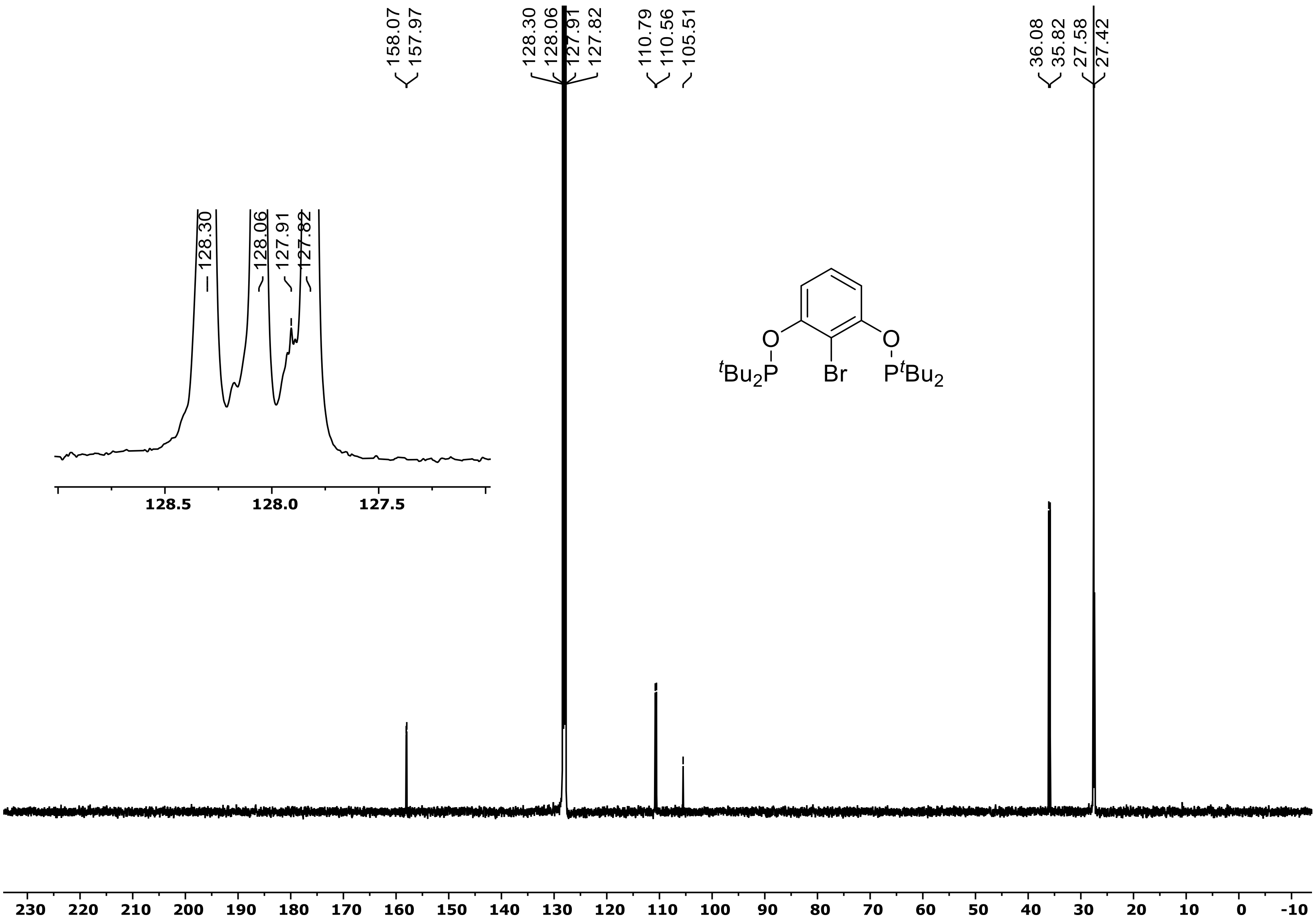

Figure S11: ^{31}P NMR spectrum of **1** (162 MHz, C_6D_6, 298 K).

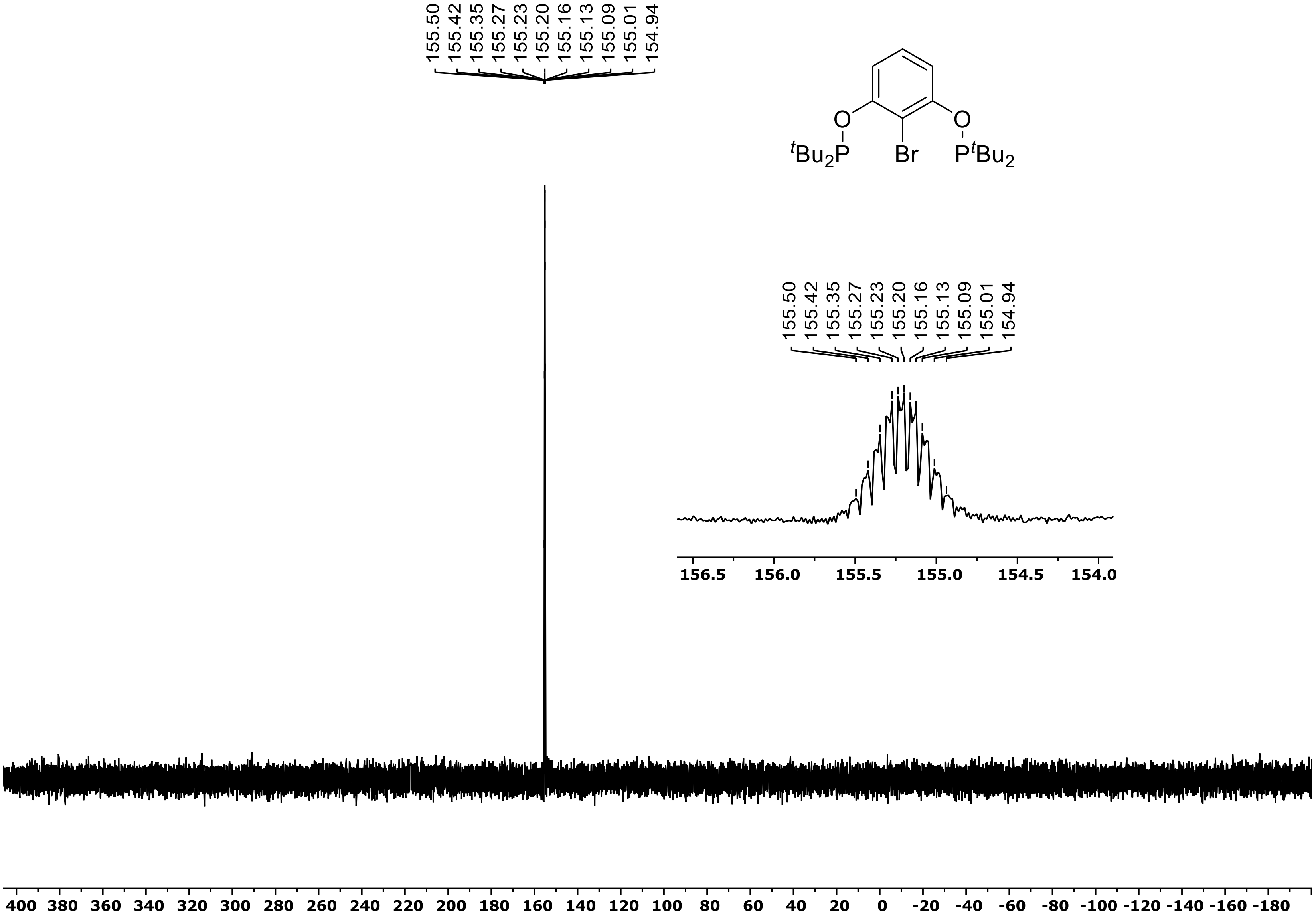

Figure S12: $^{31}P\{^{1}H\}$ NMR spectrum of **1** (162 MHz, C_6D_6, 298 K).

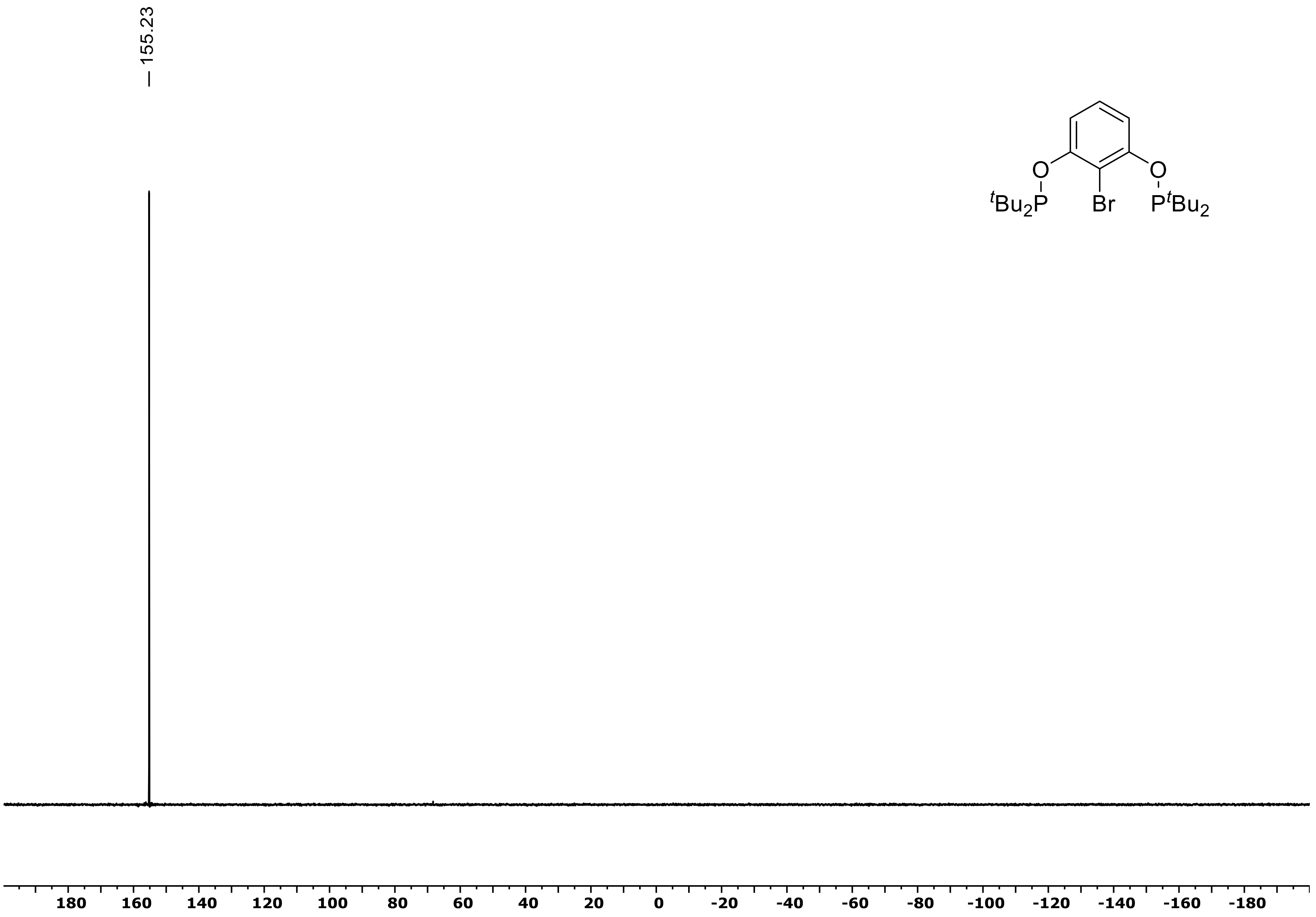

Figure S13: ^{1}H NMR spectrum of **2** (400 MHz, C_6D_6, 298 K).

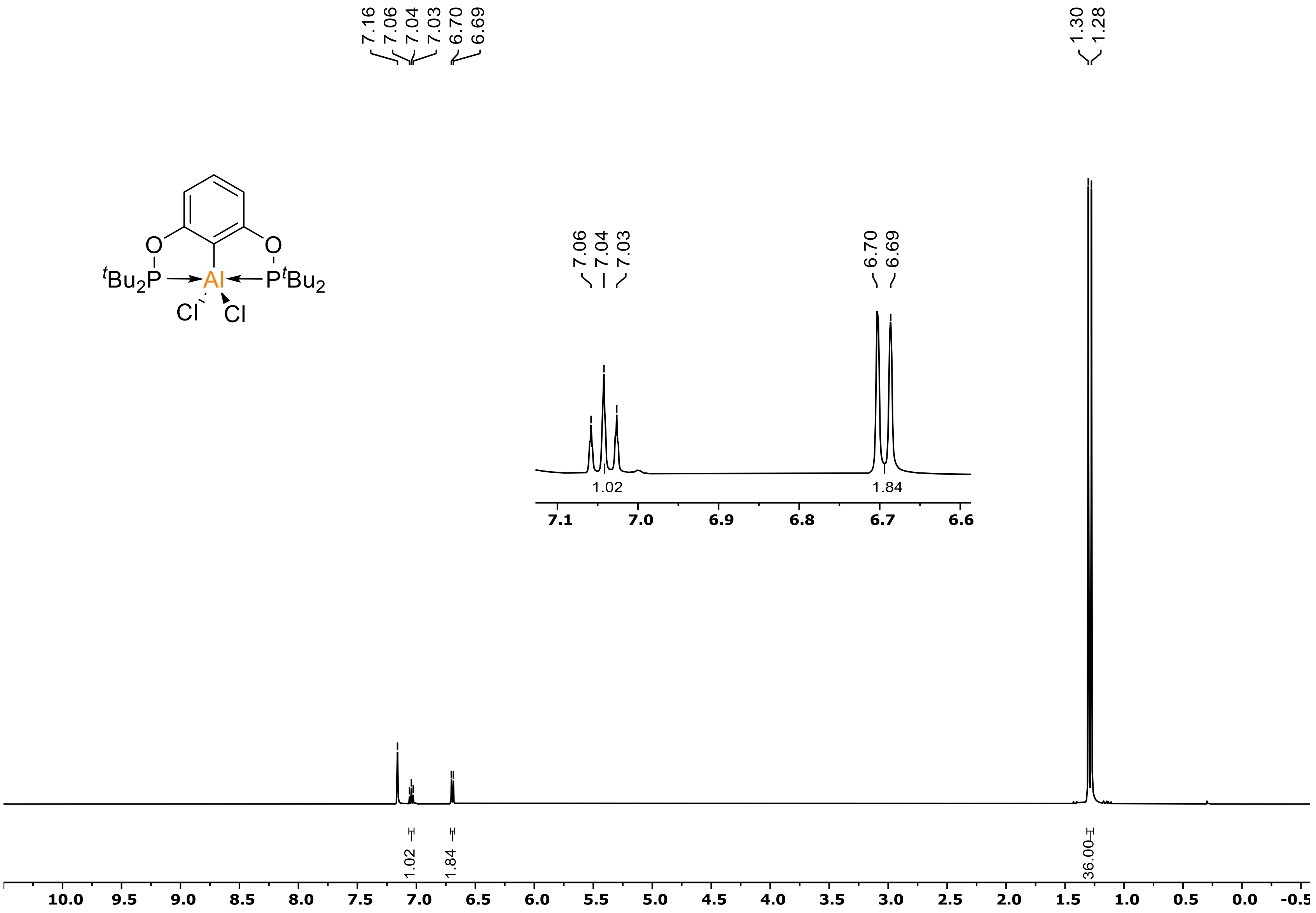

Figure S14: $^{13}C\{^{1}H\}$ NMR spectrum of **2** (101 MHz, C_6D_6, 298 K).

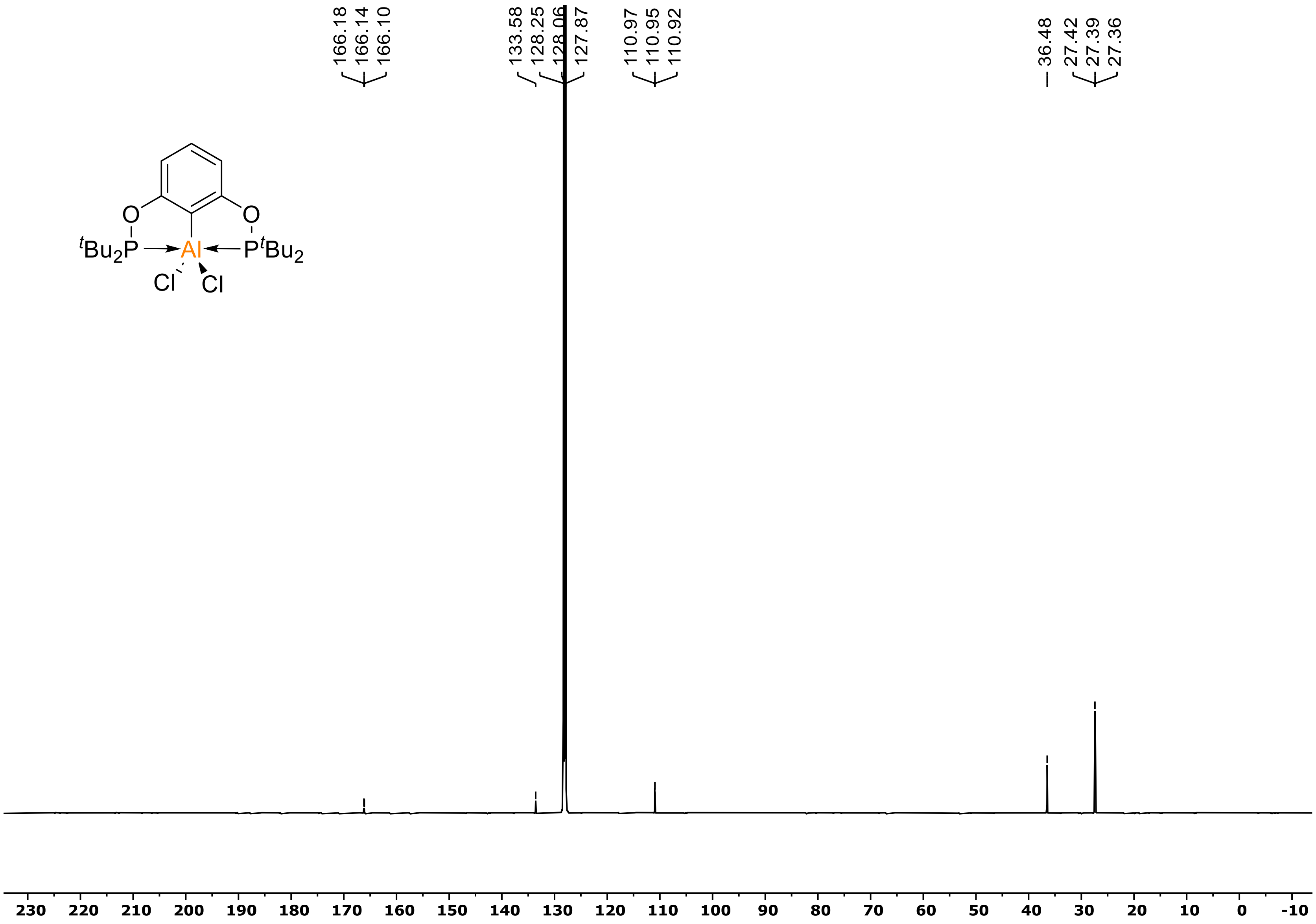

Figure S15: ^{1}H-^{13}C HMBC NMR spectrum of **2** (C_6D_6, 298 K).

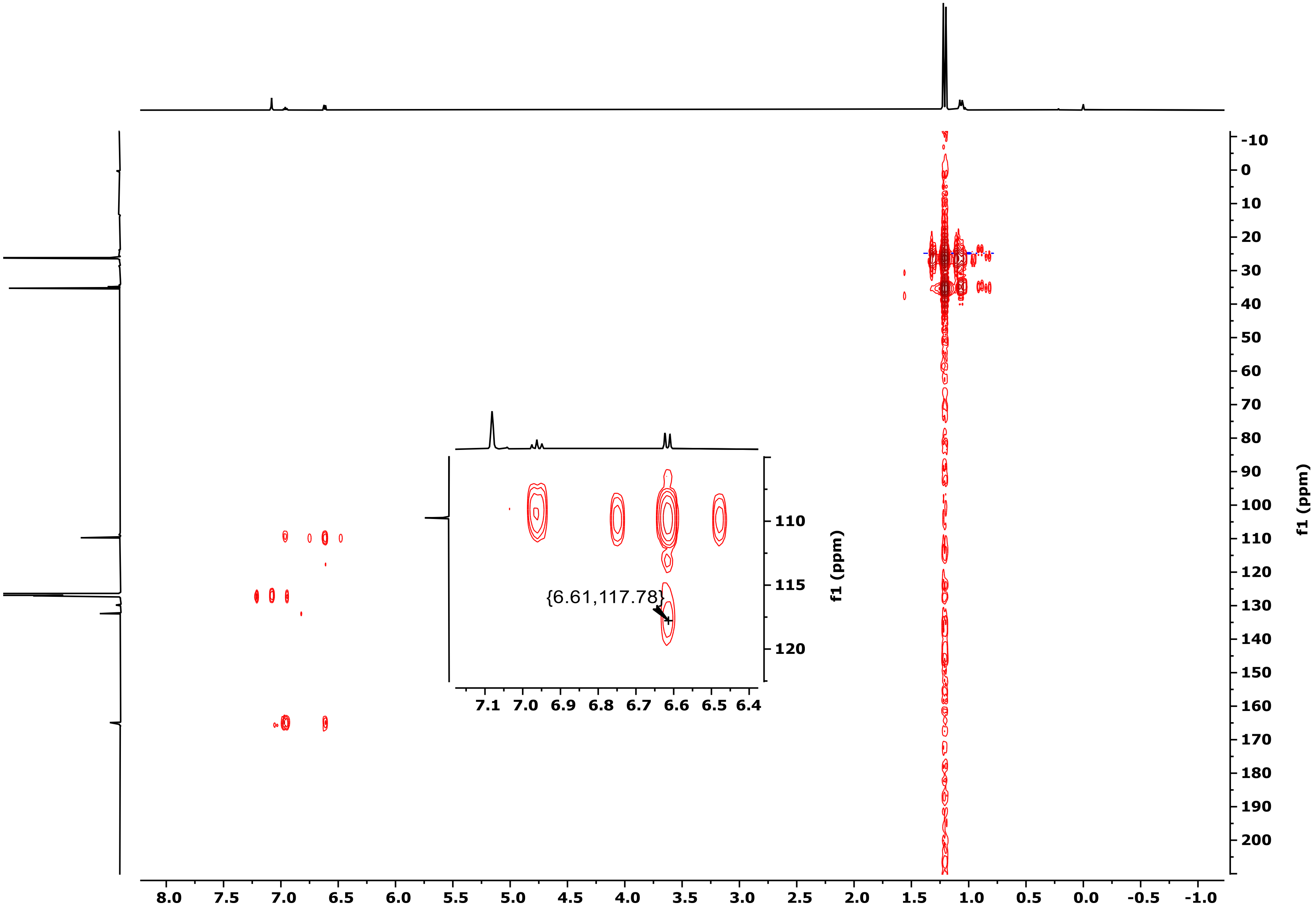

Figure S16: ^{31}P NMR spectrum of **2** (162 MHz, C_6D_6, 298 K).

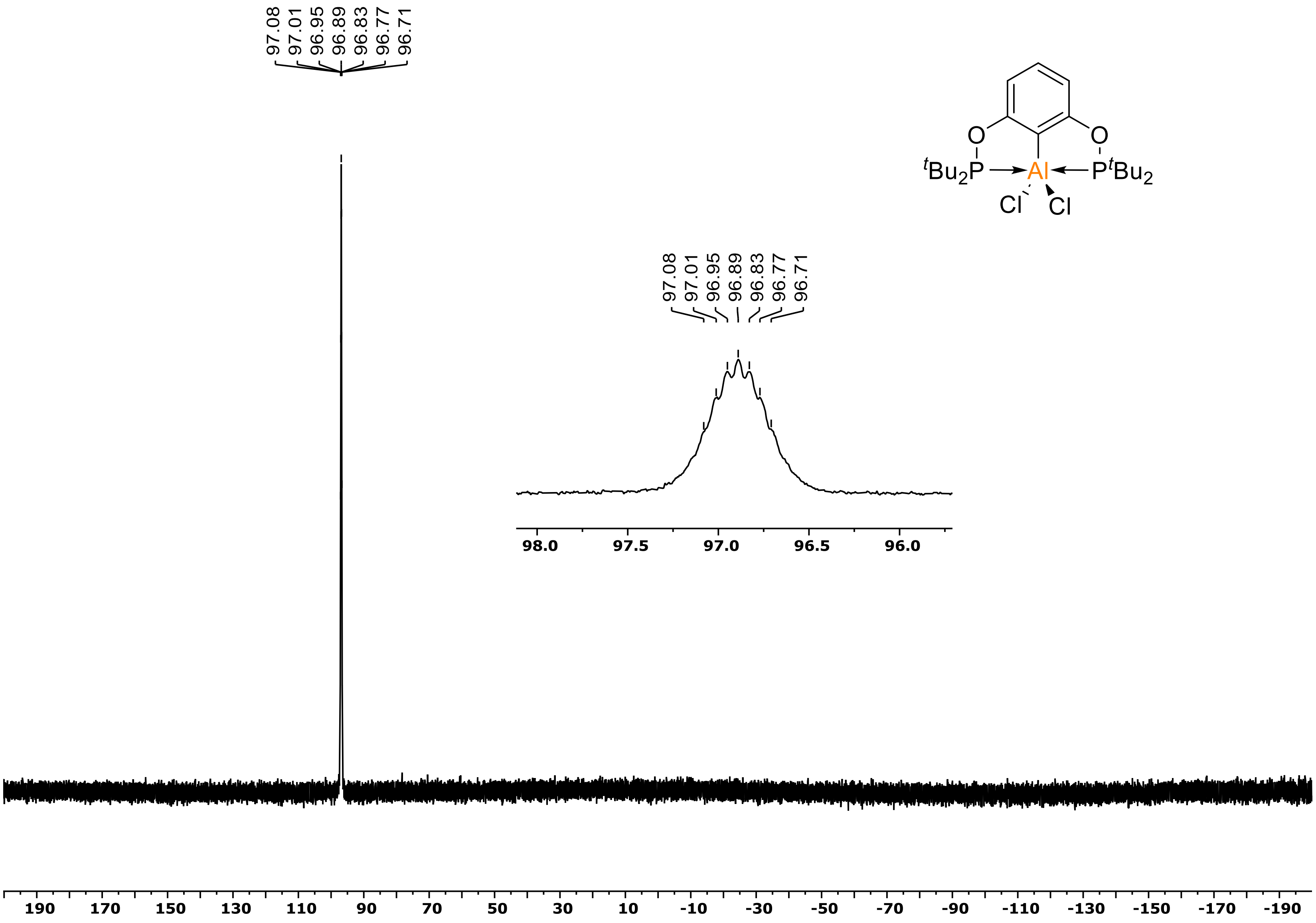

Figure S17: $^{31}P\{^{1}H\}$ NMR spectrum of **2** (162 MHz, C_6D_6, 298 K).

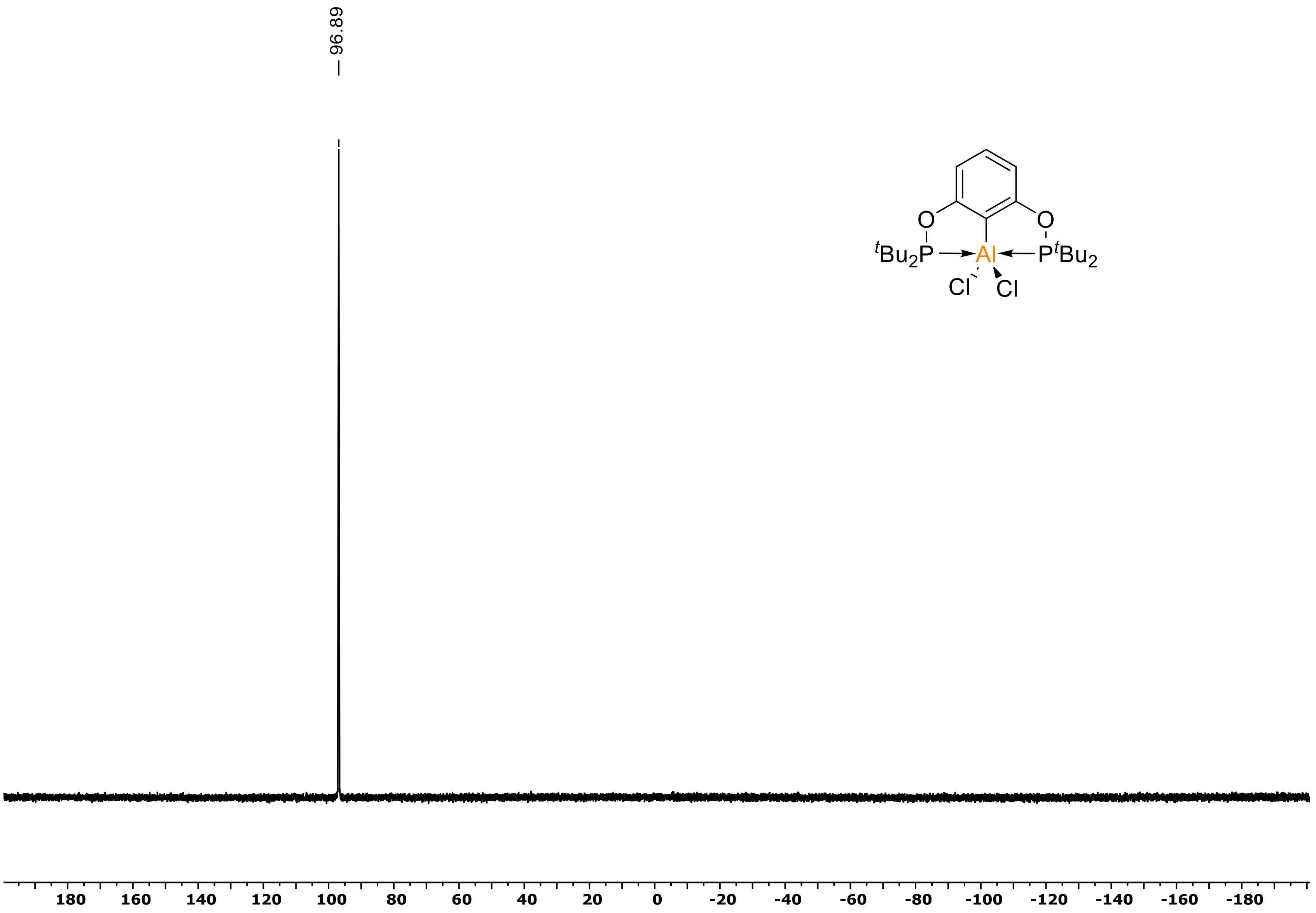

Figure S18: ^{1}H NMR spectrum of **3**/**3'** (400 MHz, Tol-d_8, 213 K).

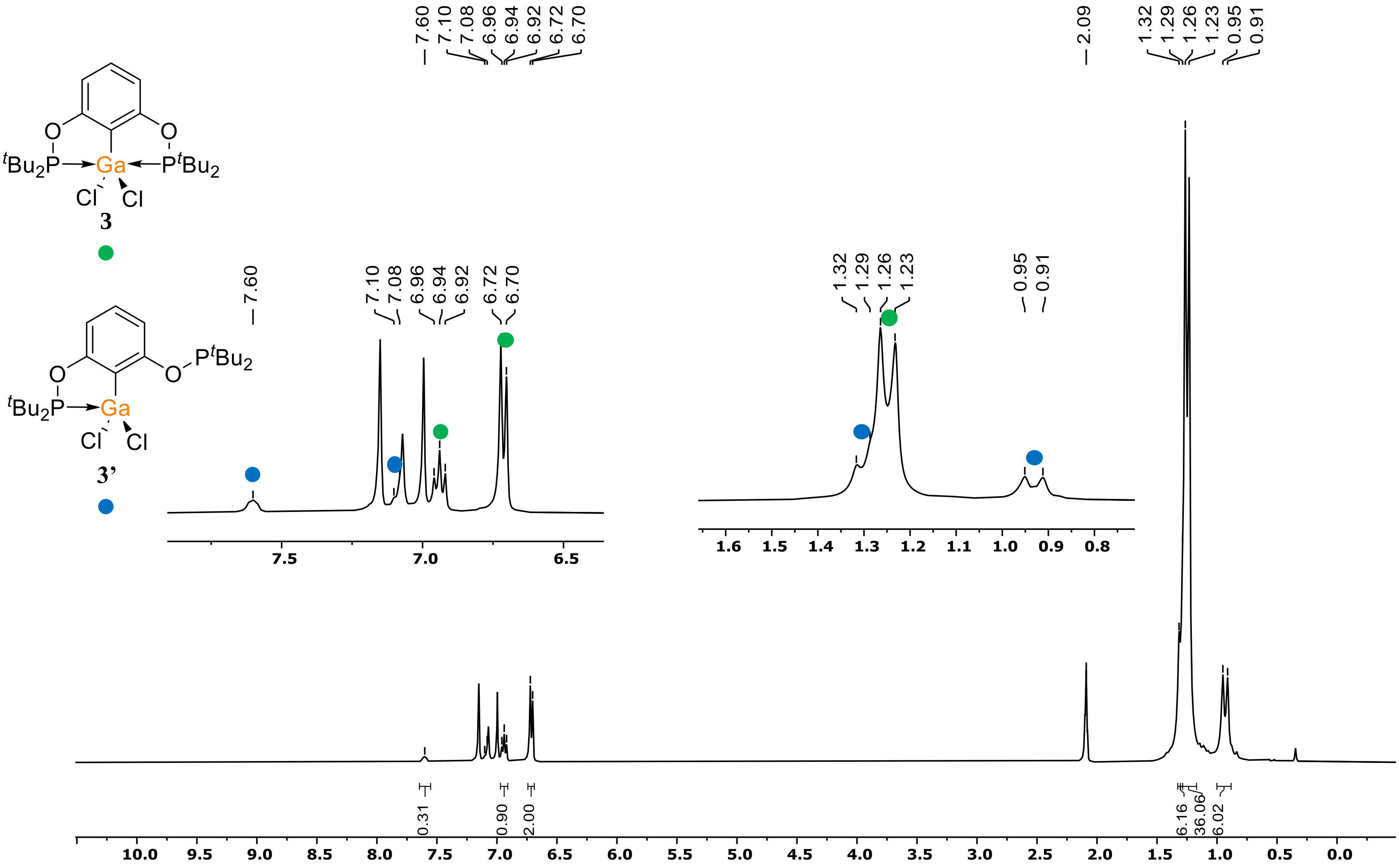

Figure S19: $^{13}C\{^{1}H\}$ NMR spectrum of **3**/**3’** (101 MHz, C_6D_6, 298 K).

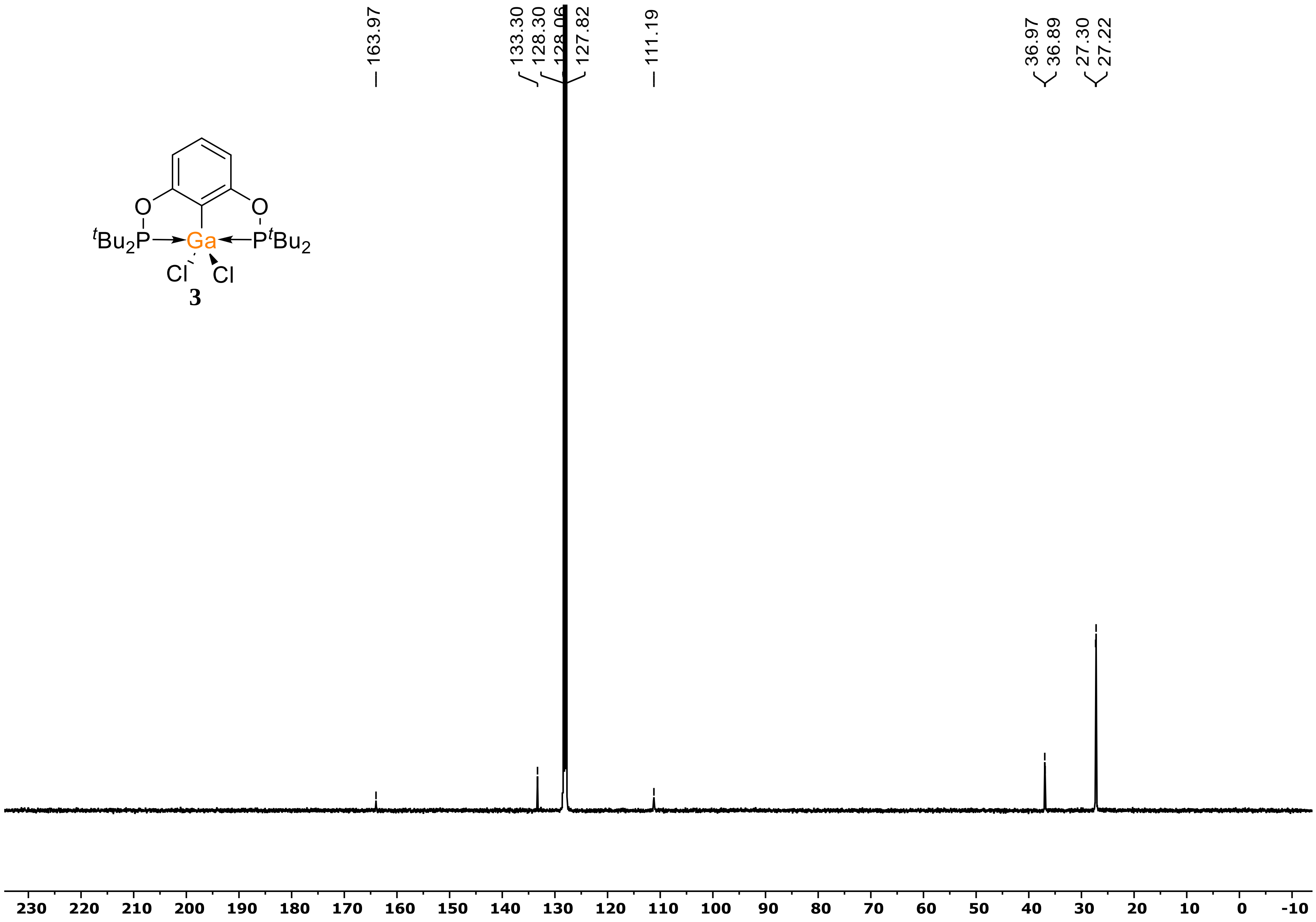

Figure S20: ^{31}P NMR spectrum of **3**/**3'** (162 MHz, C_6D_6, 298 K).

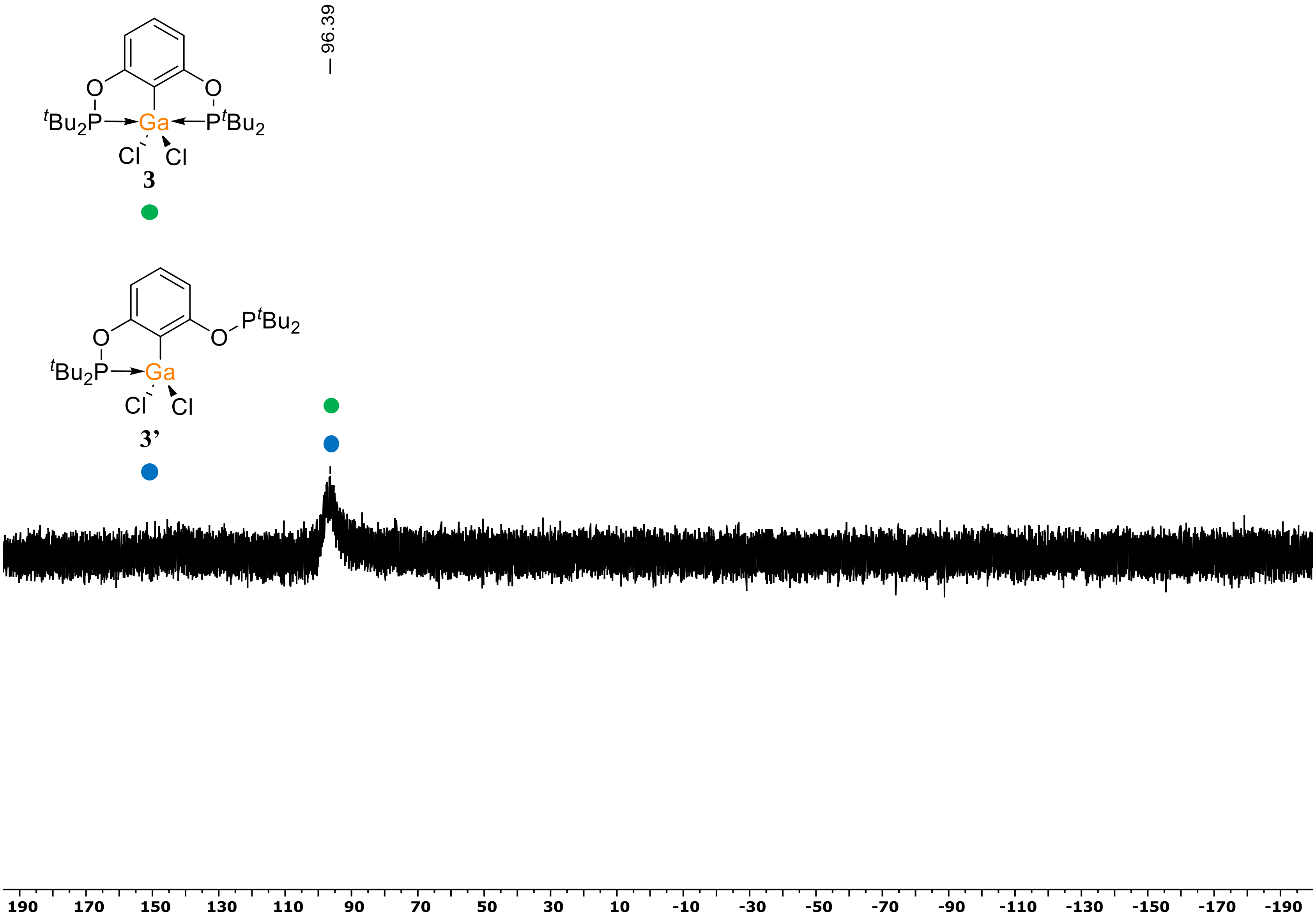

Figure S21: $^{31}P\{^{1}H\}$ inverse gated NMR spectrum of **3/3’** (162 MHz, Tol-d_8, 212 K).

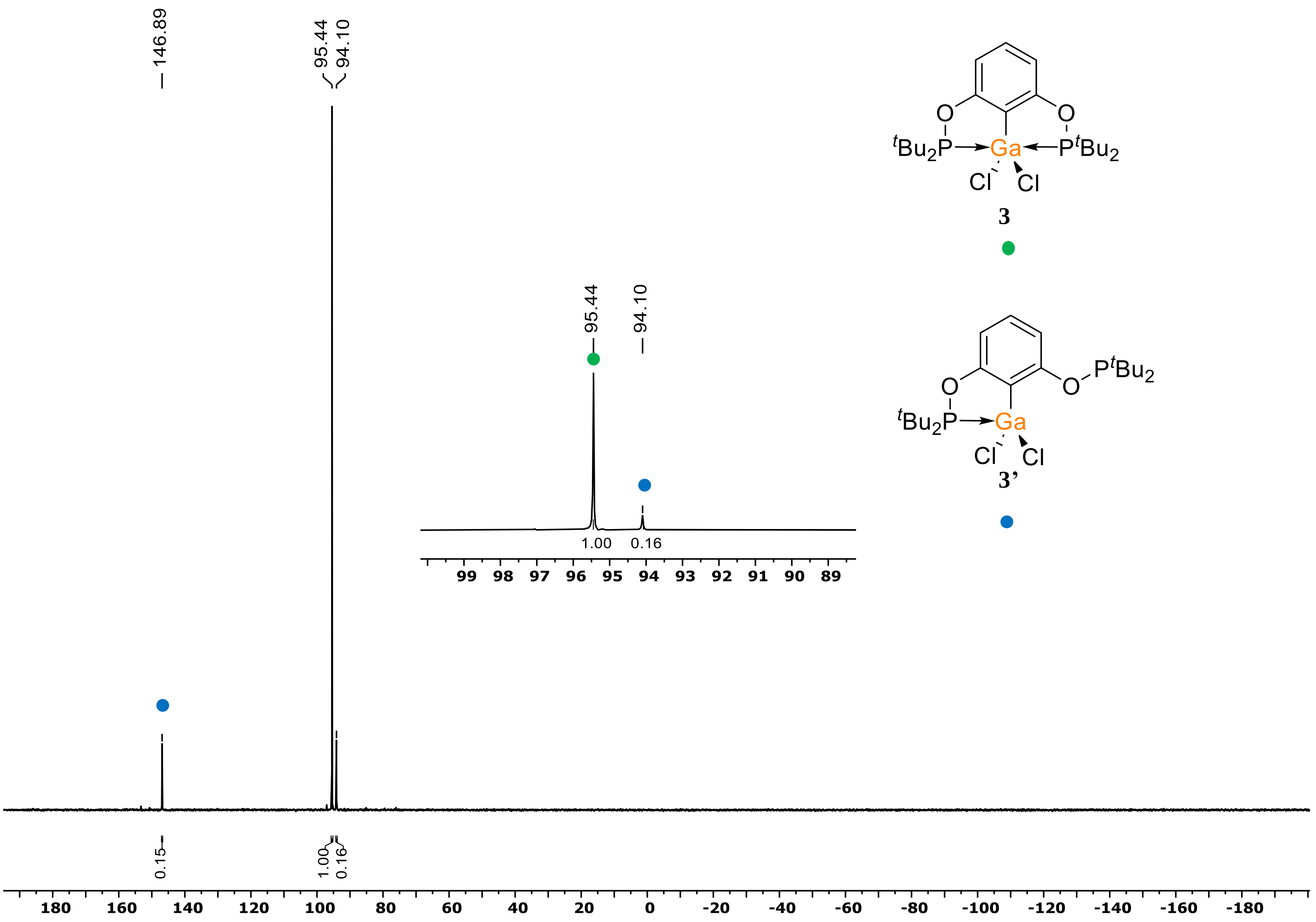

Figure S22: ^{1}H NMR speca of **3**/**3'** (400 MHz, Tol-d_8, 211 K (orange), 223 K (brown), 234 K (olive green), 243 K (forest green), 253 K (teal), 263 K (dark blue), 272 K (purple), 283 K (magenta)).

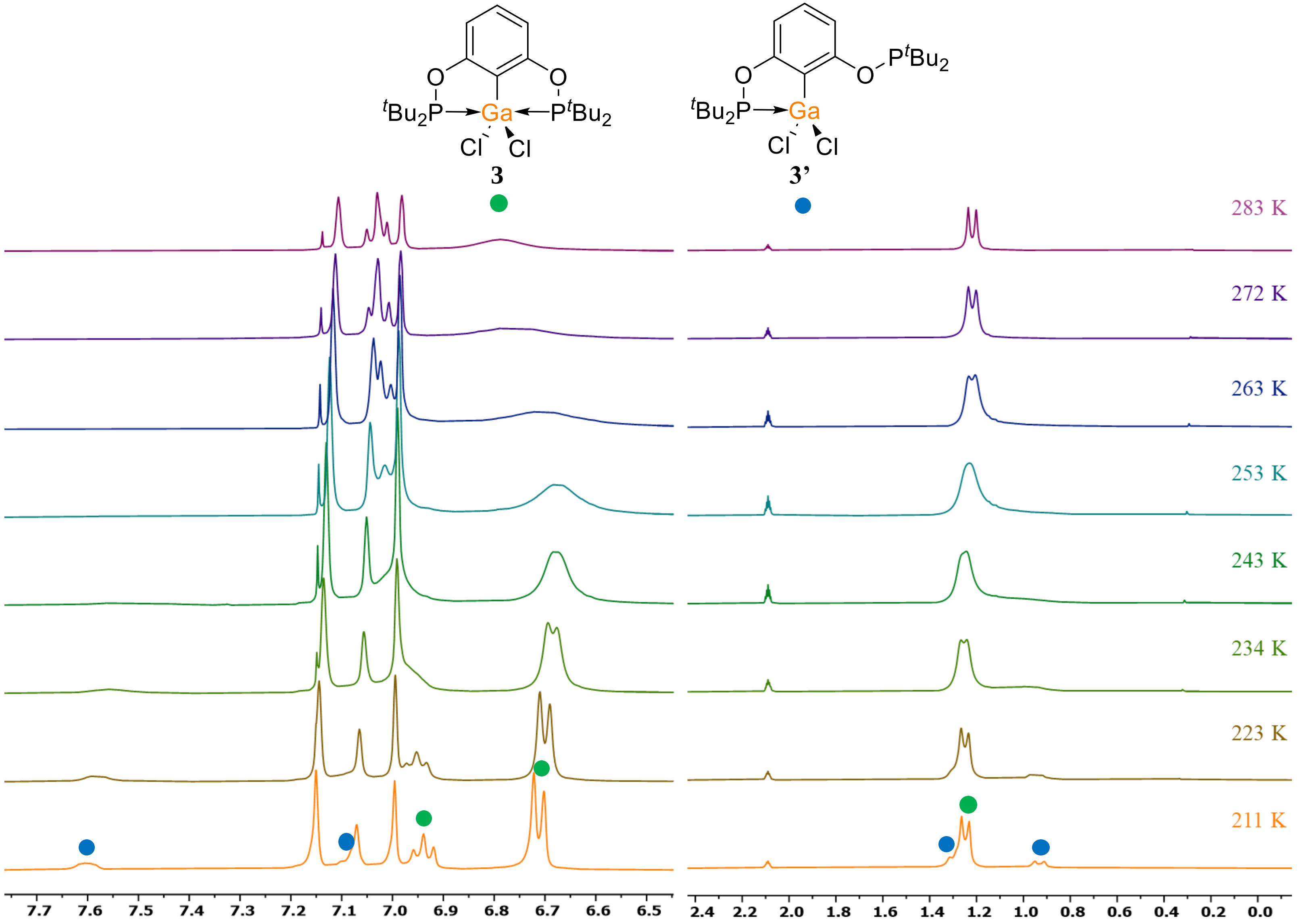

Figure S23: ^{1}H NMR specta of **3**/ **3’** before and after VT experiments (400 MHz, Tol-d_8, 293 K (orange) and 283 K (teal)).

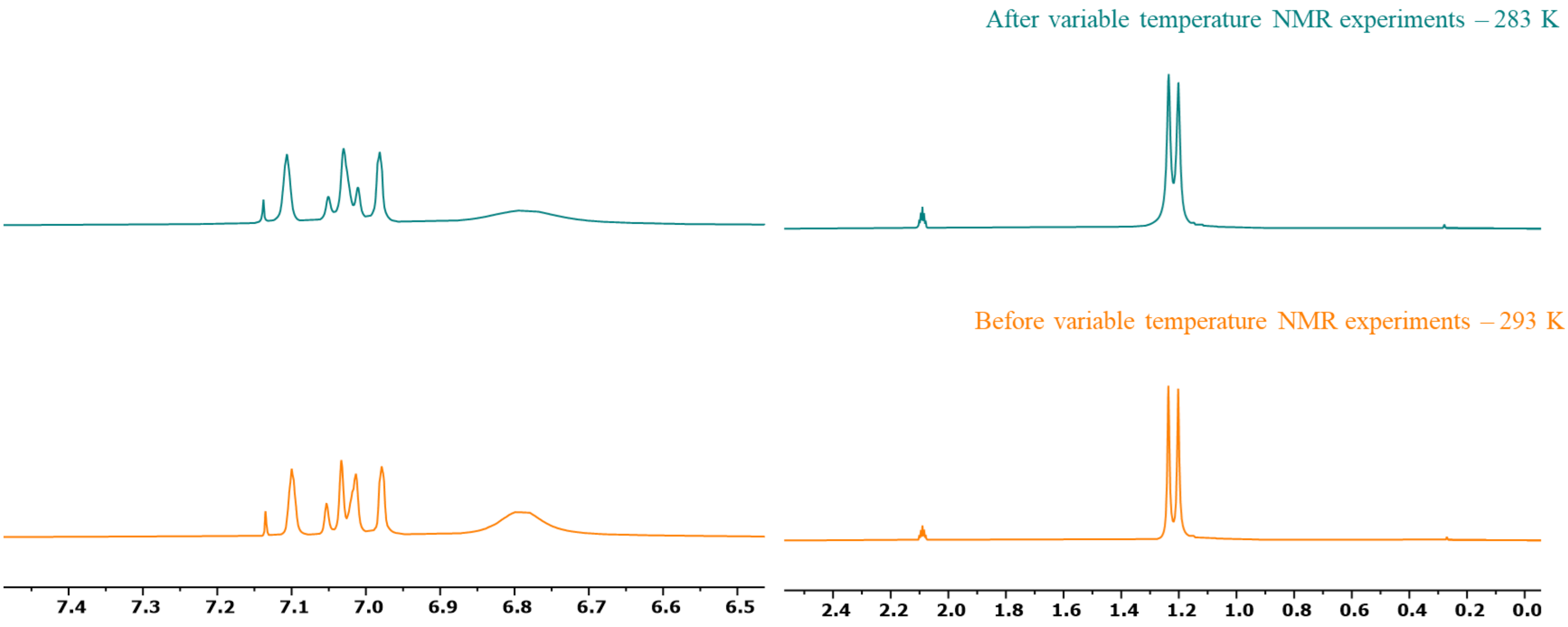

Figure S24: $^{1}H\{^{31}P\}$ NMR spectra of **3**/**3'**(400 MHz, Tol-d_8, 212 K (orange), 294 K (brown), 323 K (olive green)).

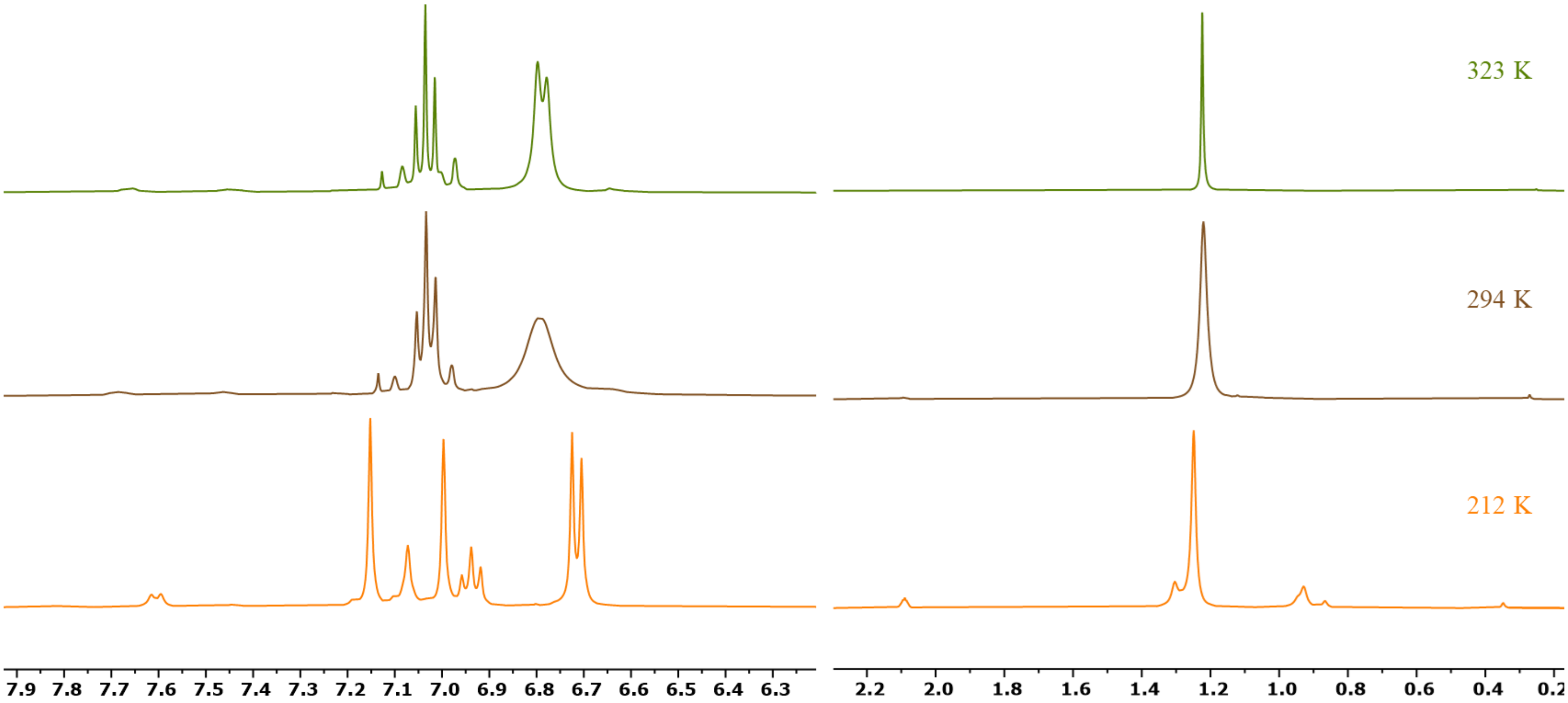

Figure S25: $^{31}P\{^1H\}$ NMR spectra of **3**/ **3'** (162 MHz, Tol-*d*8, 211 K (orange), 223 K (brown), 234 K (olive green), 243 K (forest green), 253 K (teal), 263 K (dark blue), 272 K (purple), 283 K (magenta)).

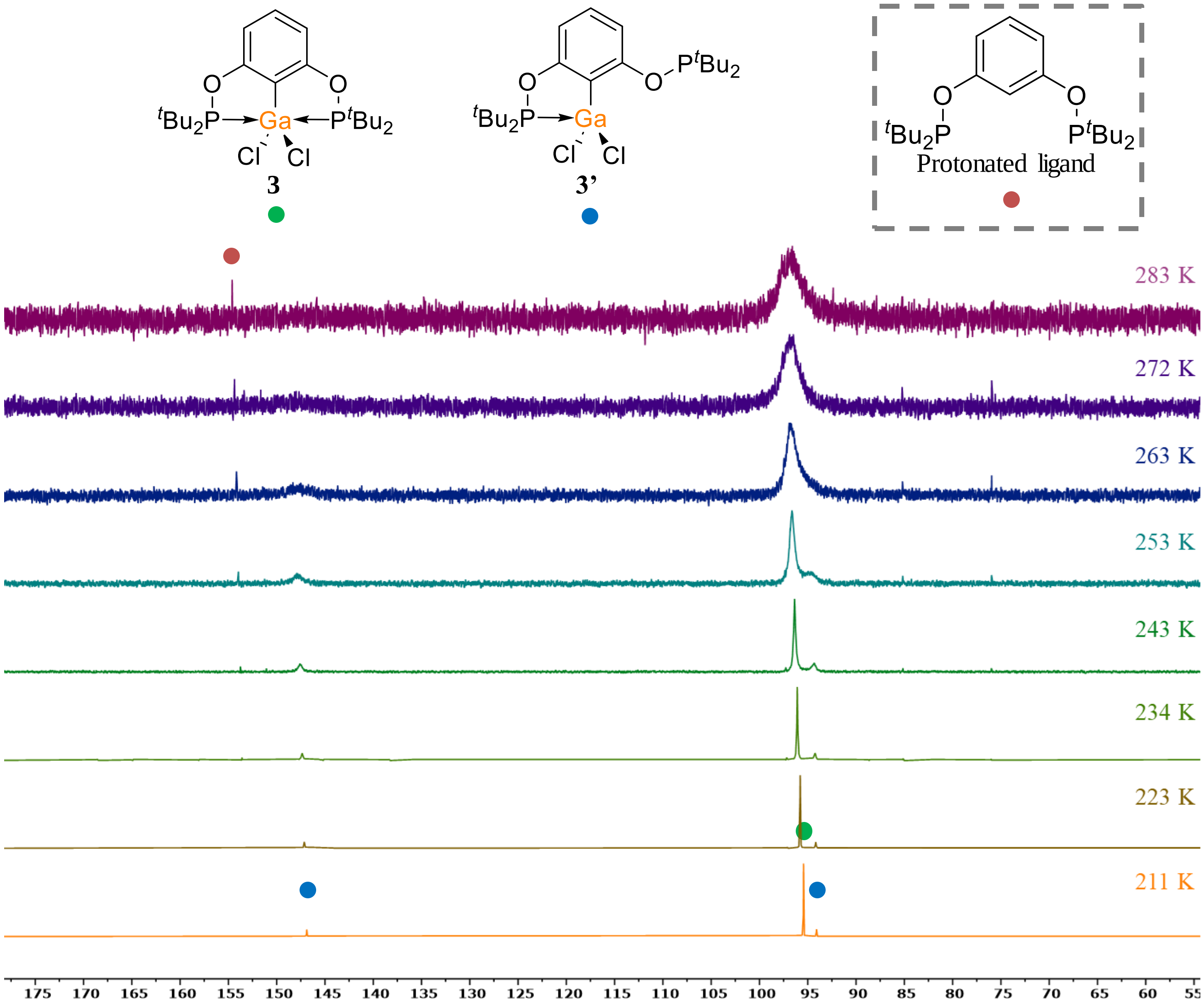

Figure S26: $^{31}P\{^{1}H\}$ NMR spectra of **3/3'** before and after VT experiments (400 MHz, Tol-d_8, 293 K (orange) and 283 K (teal)).

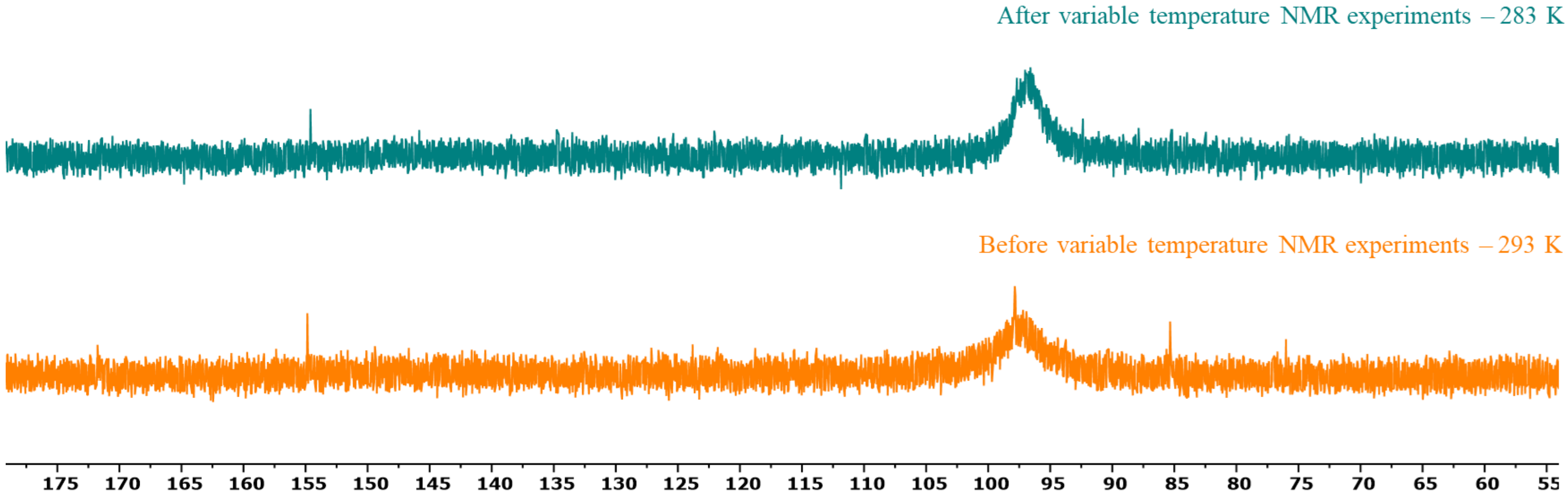

Figure S27: HRMS (APCI) mass spectrum of **3**/**3'**. Peak at 537.1147 corresponds to $[M+H]^{+}$.

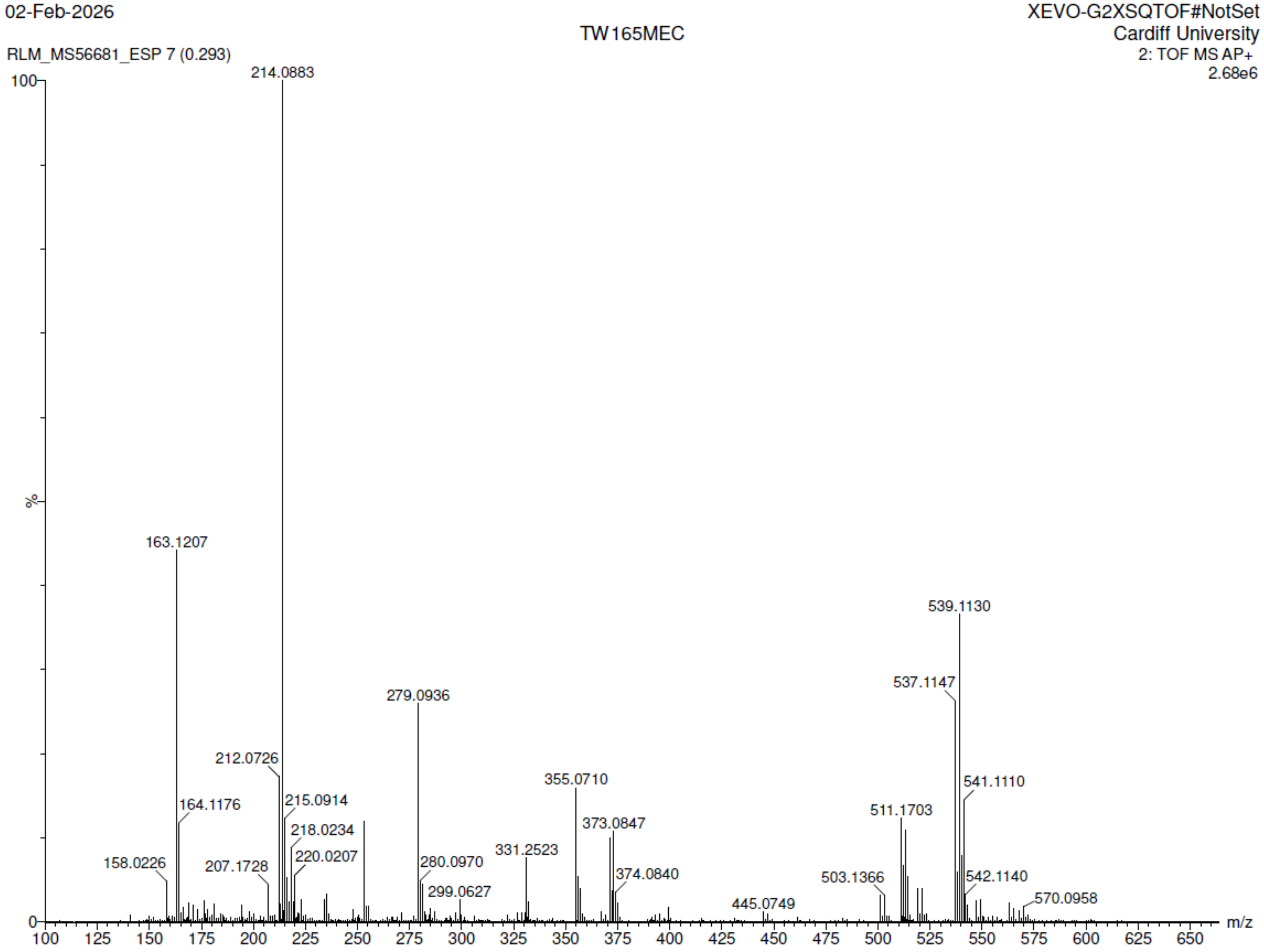

Figure S28: ^{1}H NMR spectrum of **4** (400 MHz, C_6D_6, 298 K).

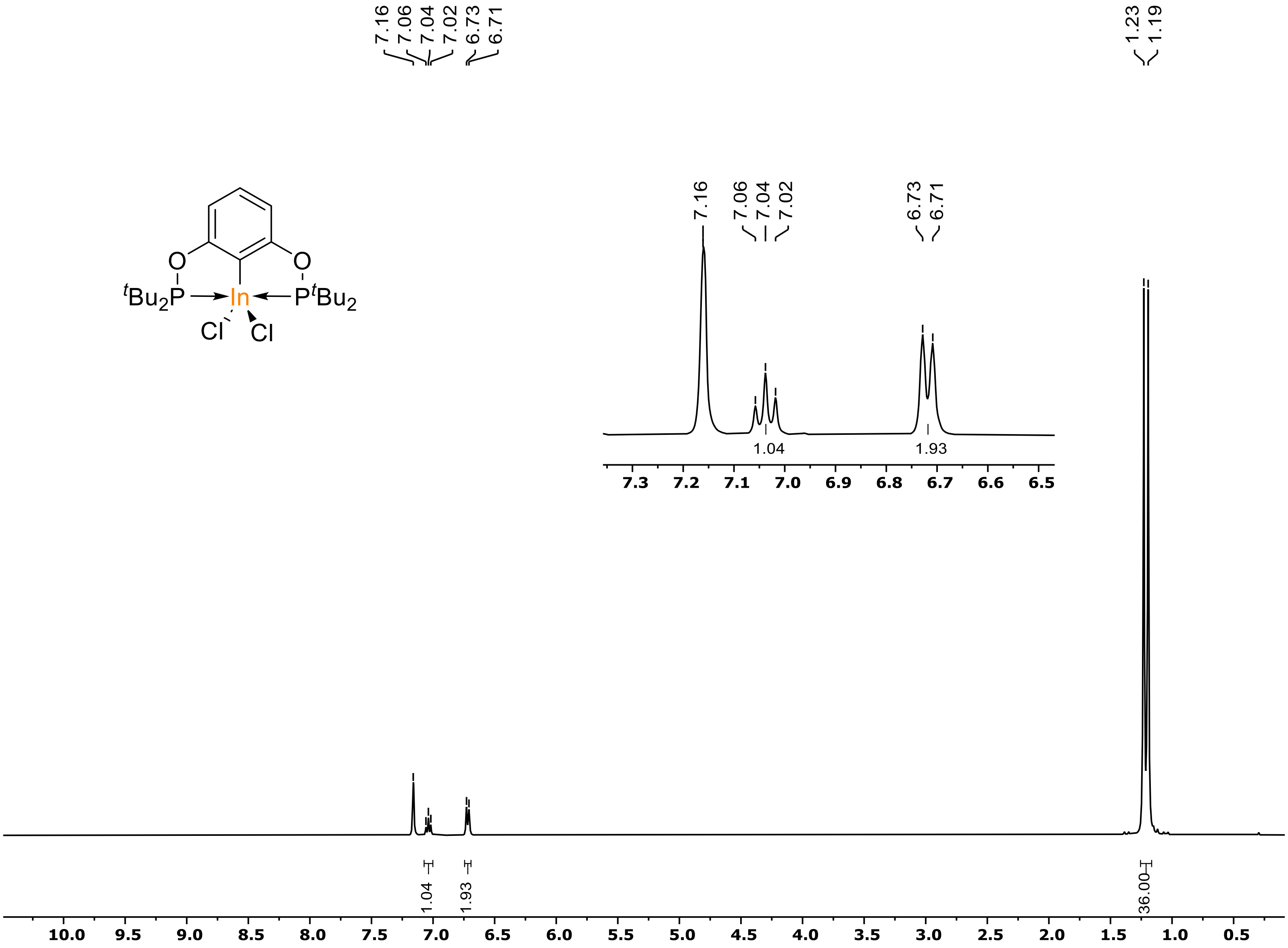

Figure S29: $^{13}C\{^{1}H\}$ NMR spectrum of **4** (101 MHz, C_6D_6, 298 K).

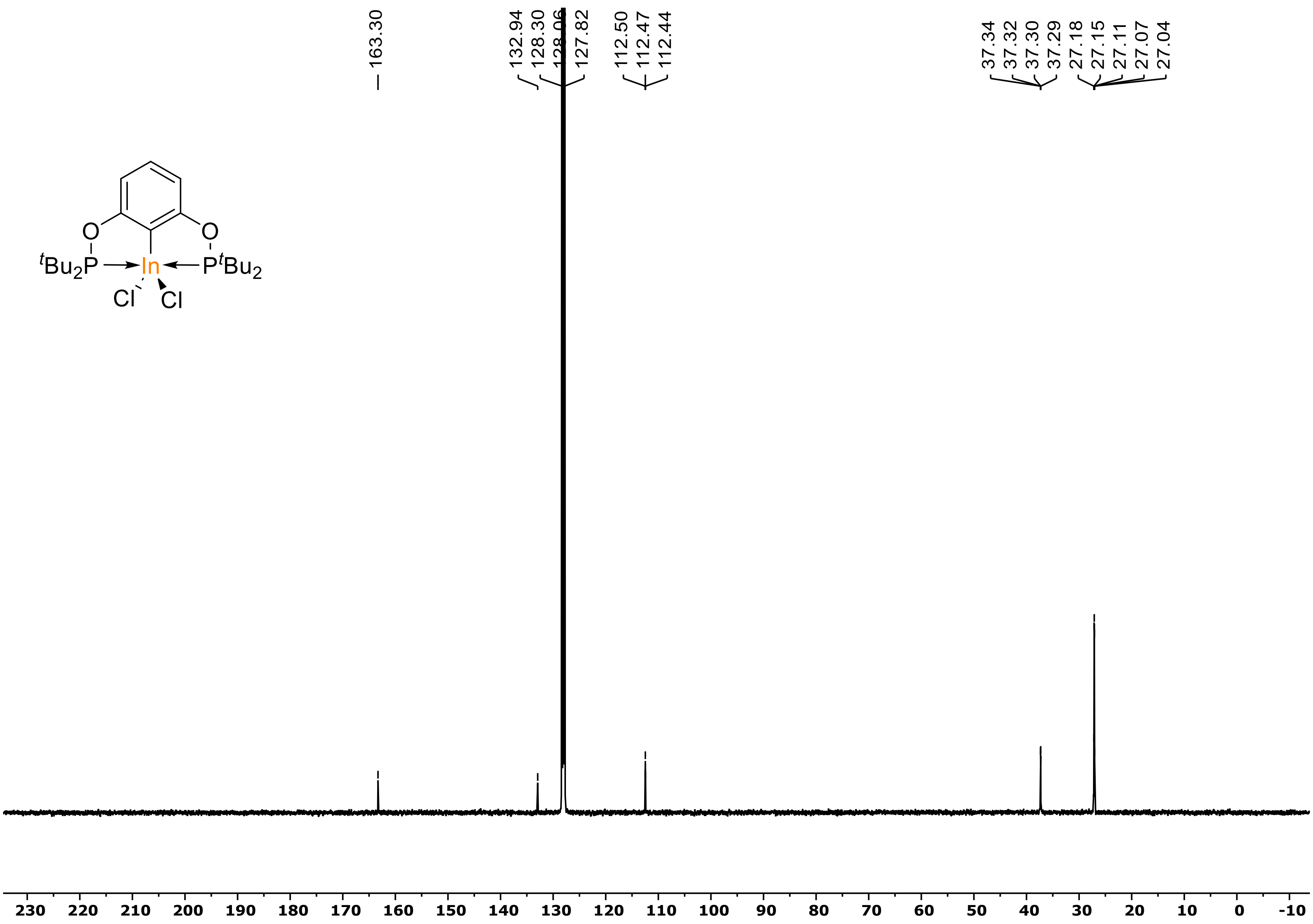

Figure S30: ^{1}H-^{13}C HMBC NMR spectrum of **4** (C_6D_6, 298 K).

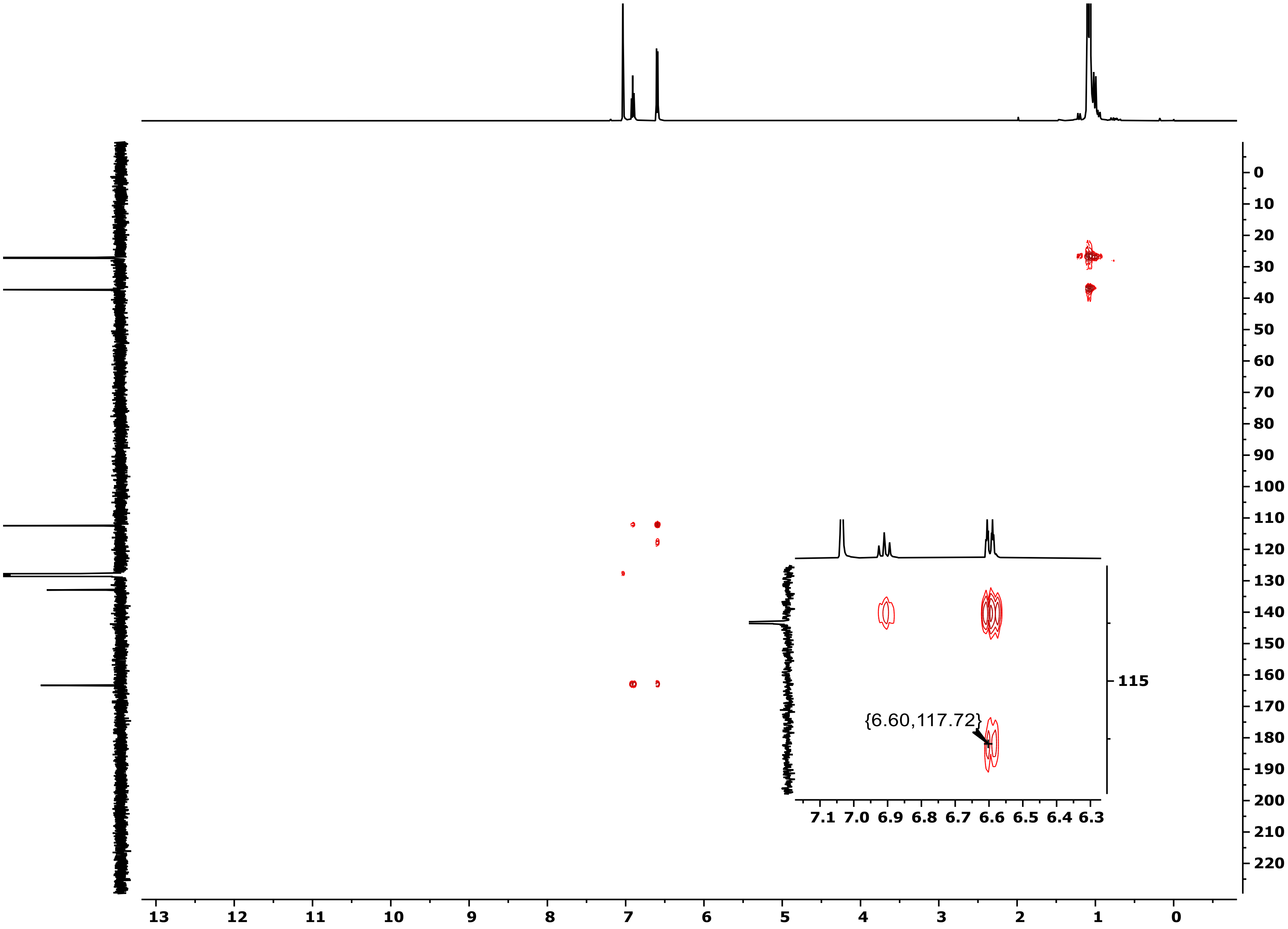

Figure S31: ^{31}P NMR spectrum of **4** (162 MHz, C_6D_6, 298 K).

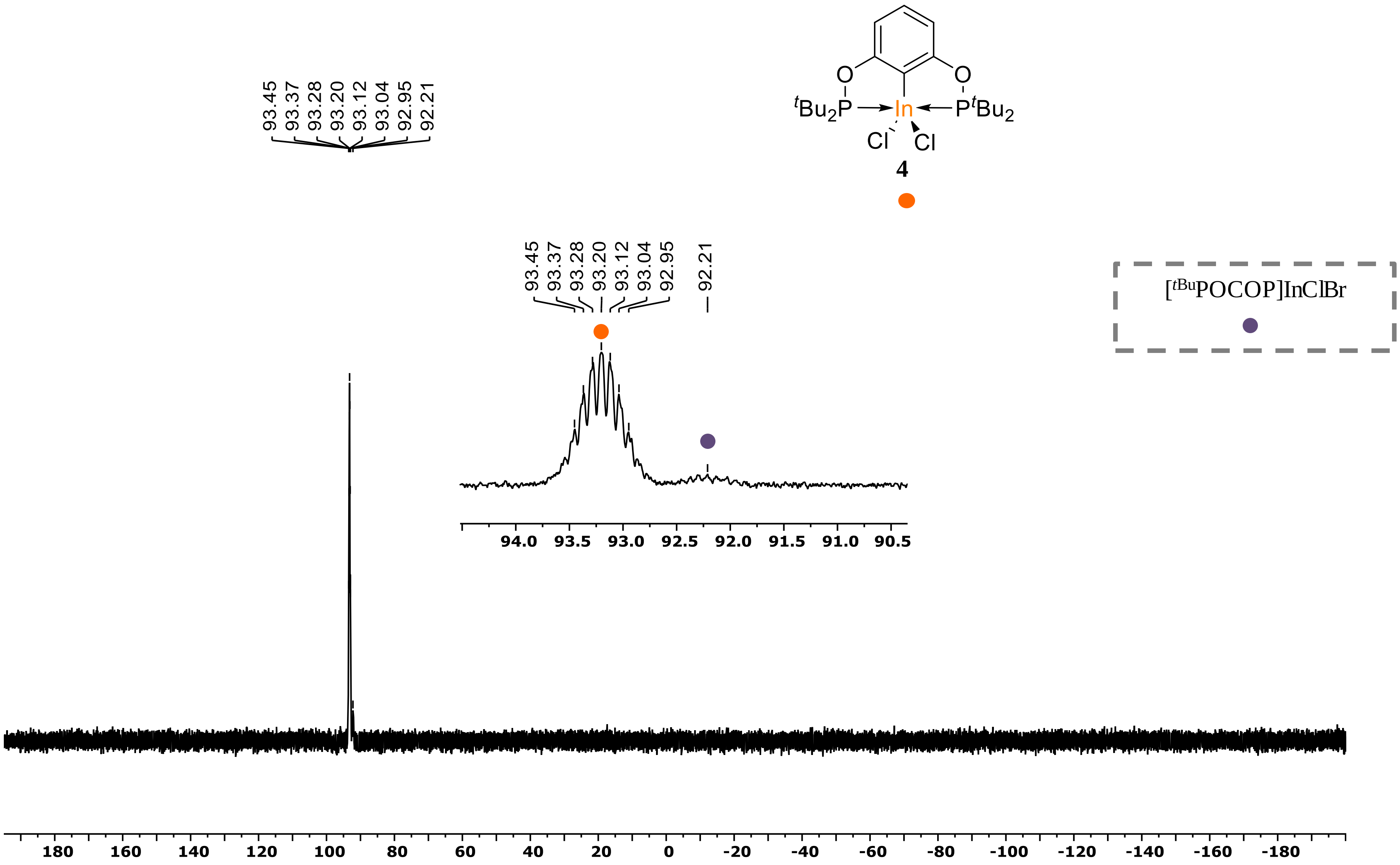

Figure S32: $^{31}P\{^{1}H\}$ NMR spectrum of **4** (162 MHz, C_6D_6, 298 K).

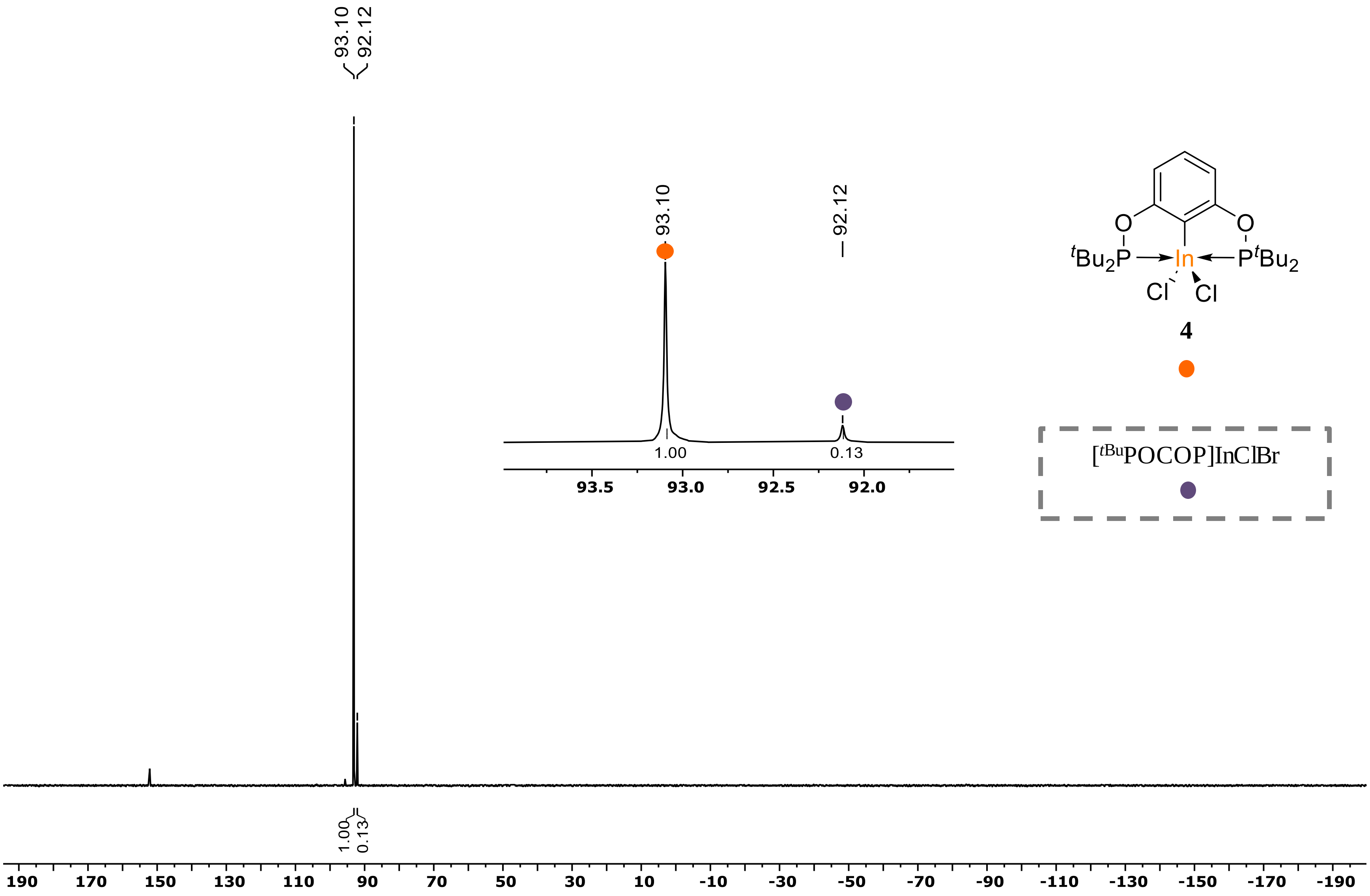

Figure S33: ^{1}H NMR spectrum of **4** (400 MHz, Tol-d_8, 298 K).

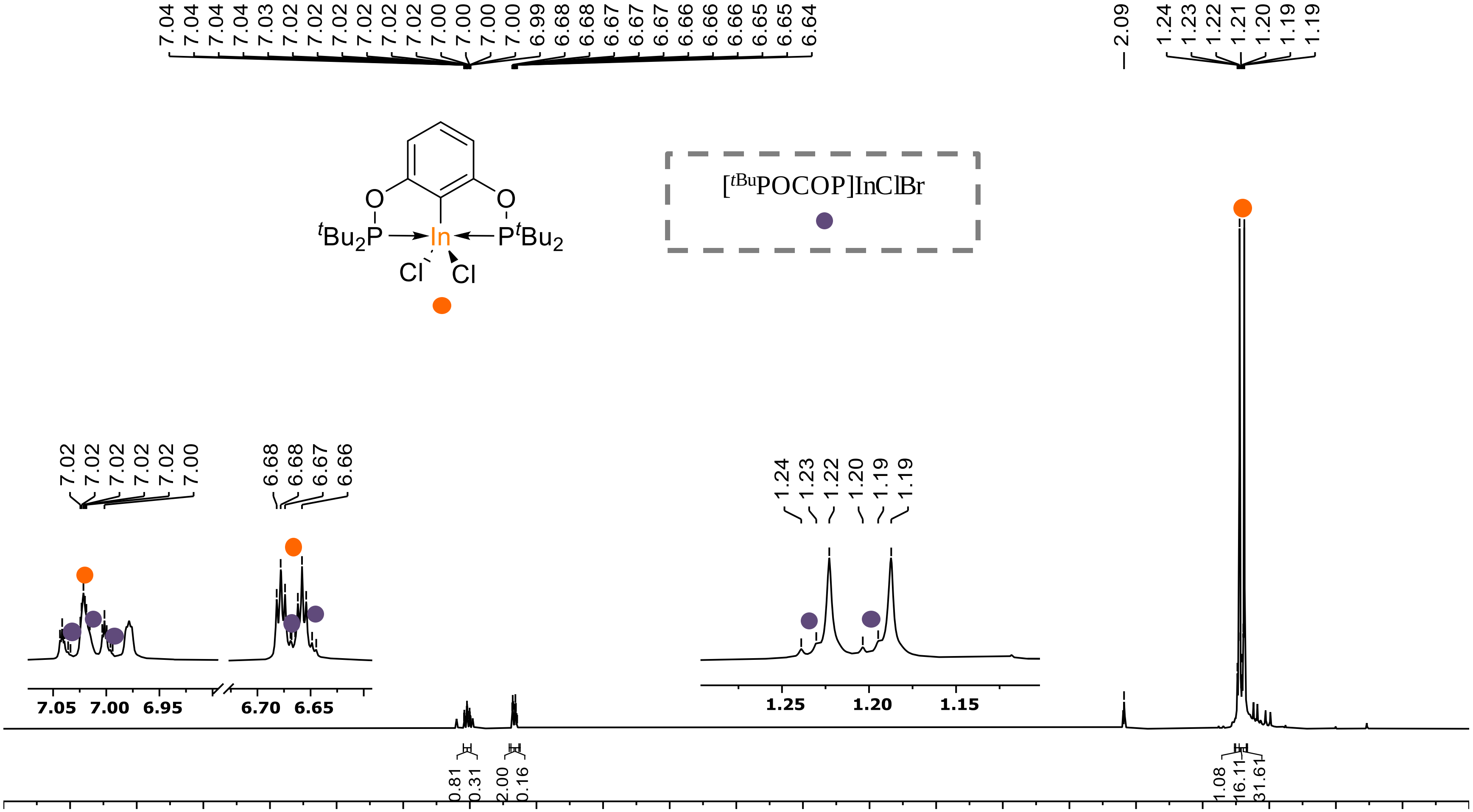

Figure S34: Top, zoom in on the ^{t}Bu signals in the ^{1}H NMR spectrum of **4** and [^{tBu}POCOP]InBrCl (400 MHz, Tol-d_8, 298 K). Bottom, line shape fitting used to determine the ratio of **4** and [^{tBu}POCOP]InBrCl.

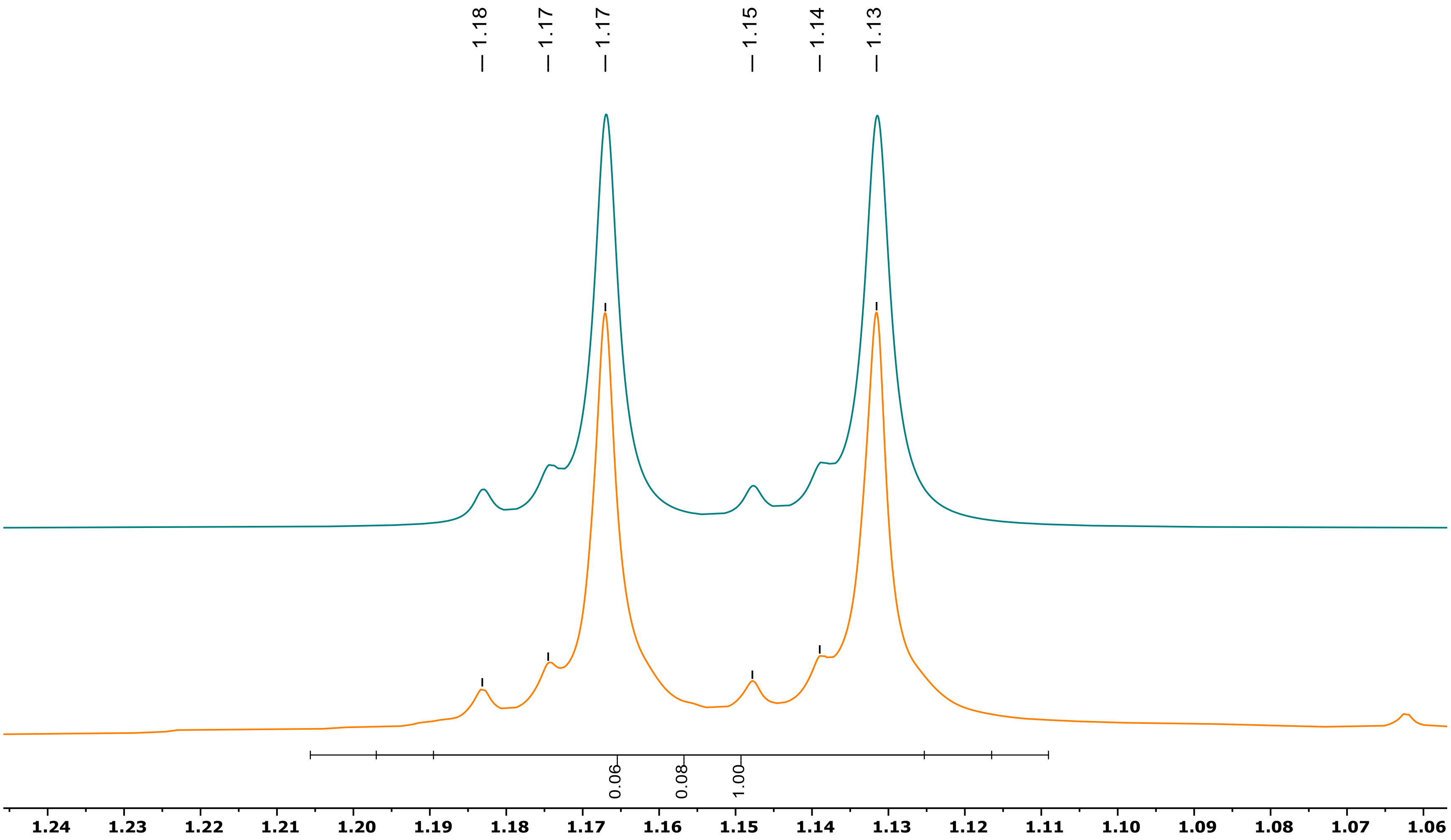

Figure S35: Output from line shape fitting used in figure S34. Black, original spectrum. Green, fit. Blue, peaks used for fit. Red, residuals.

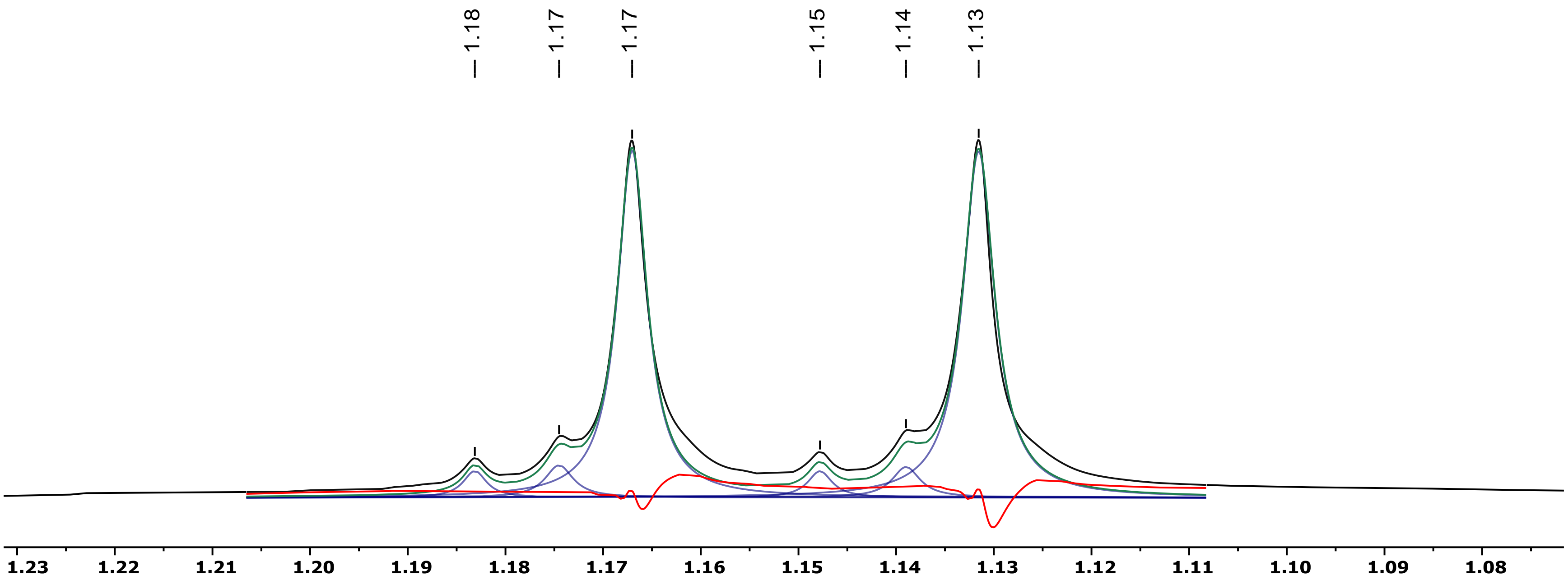

Figure S36: HRMS (ESI) mass spectrum of **4**. Peak at 583.0928 corresponds to $[M+H]^+$.

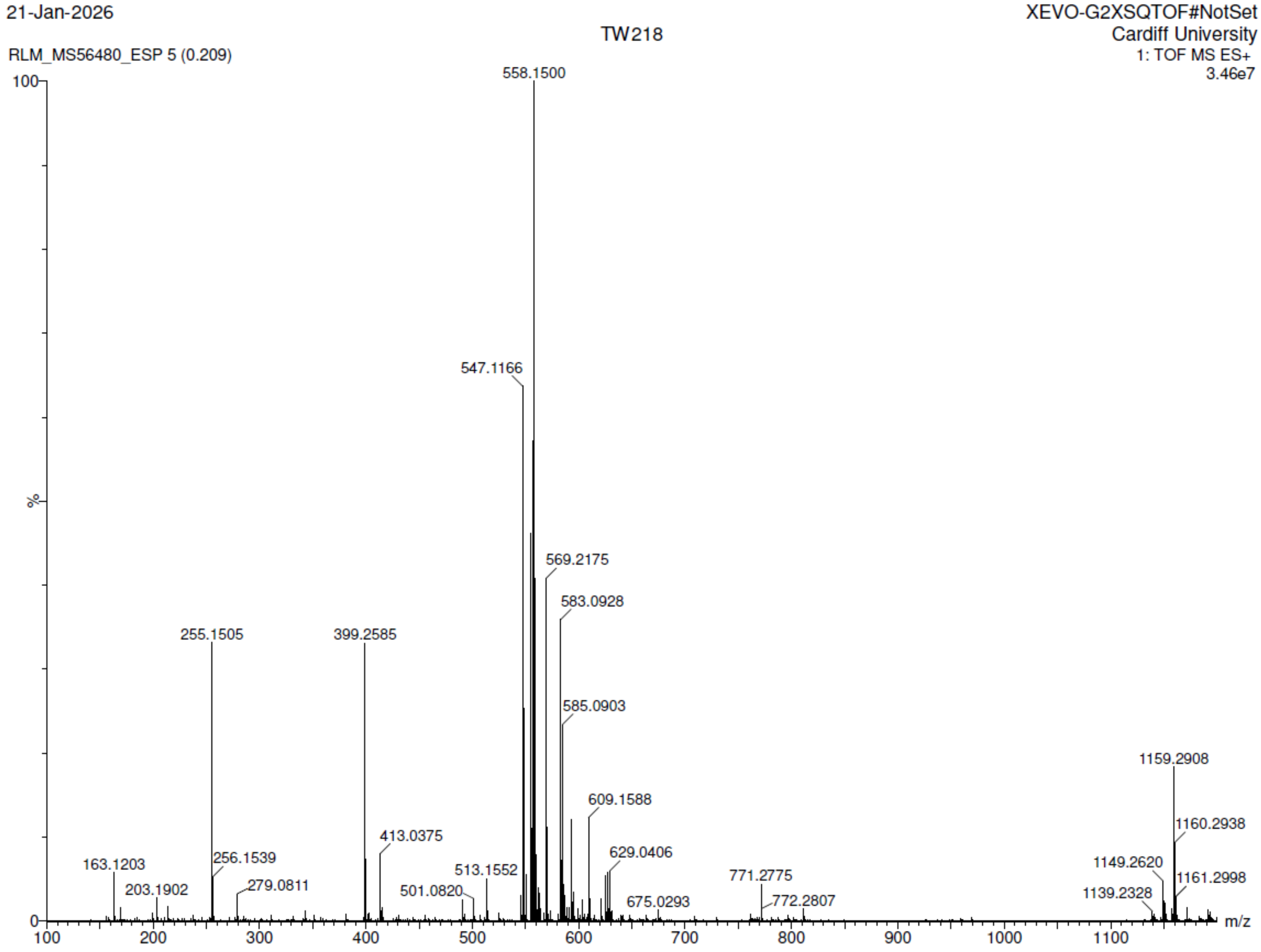

Figure S37: LRMS (ESI) mass spectrum of [^{tBu}POCOP]InBrCl. Peak at 629.04 corresponds to [M+H]$^+$. Top, modelled spectrum. Bottom, close up of the mass spectrum.

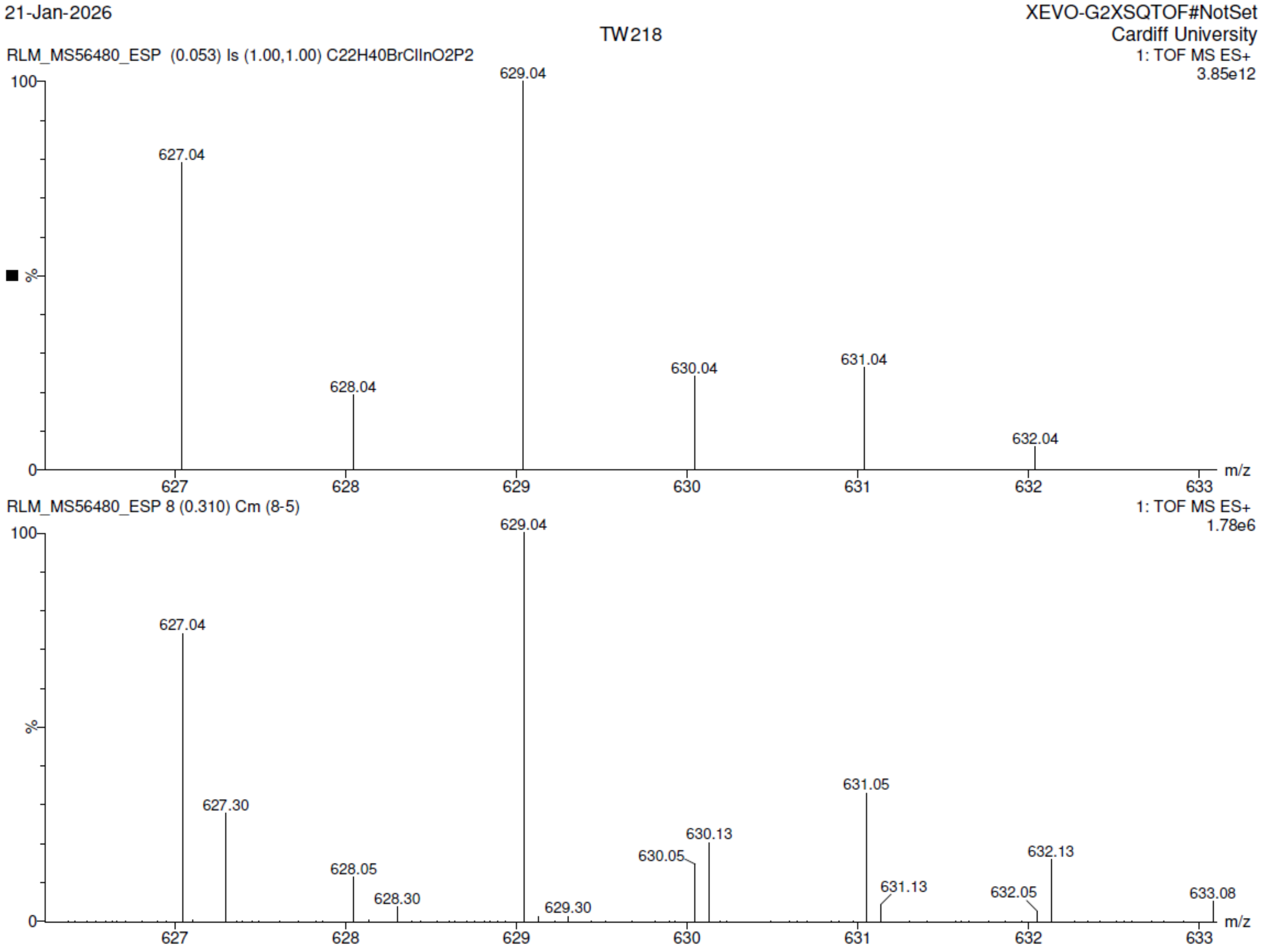

Figure S38: ^{1}H NMR spectrum of **5/5’** and [^{tBu}POCOP]AlMeCl (400 MHz, C_6D_6, 298 K).

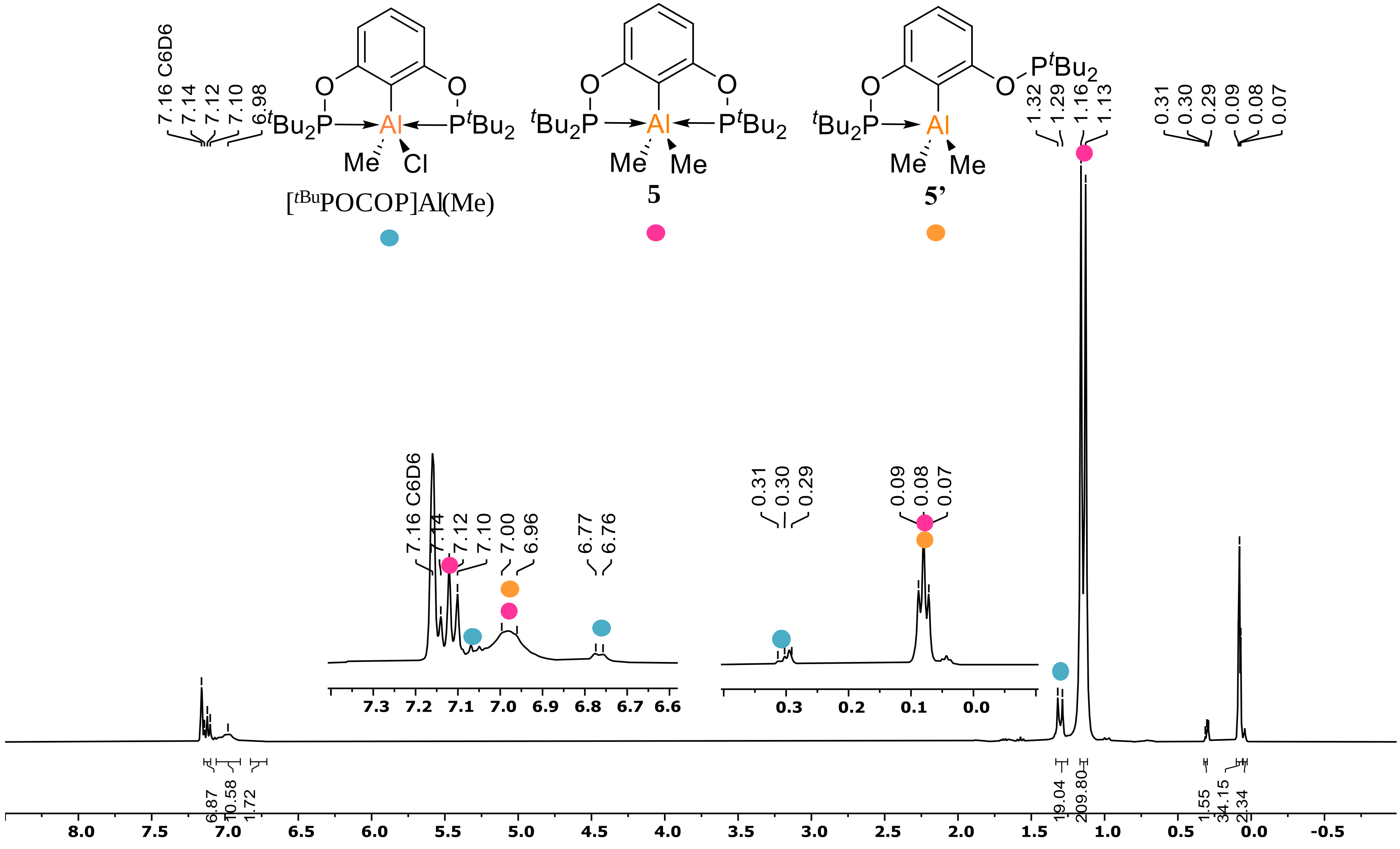

Figure S39: $^{13}C\{^1H\}$ NMR spectrum of **5**/**5'** and [^{tBu}POCOP]AlMeCl (101 MHz, C_6D_6, 298 K).

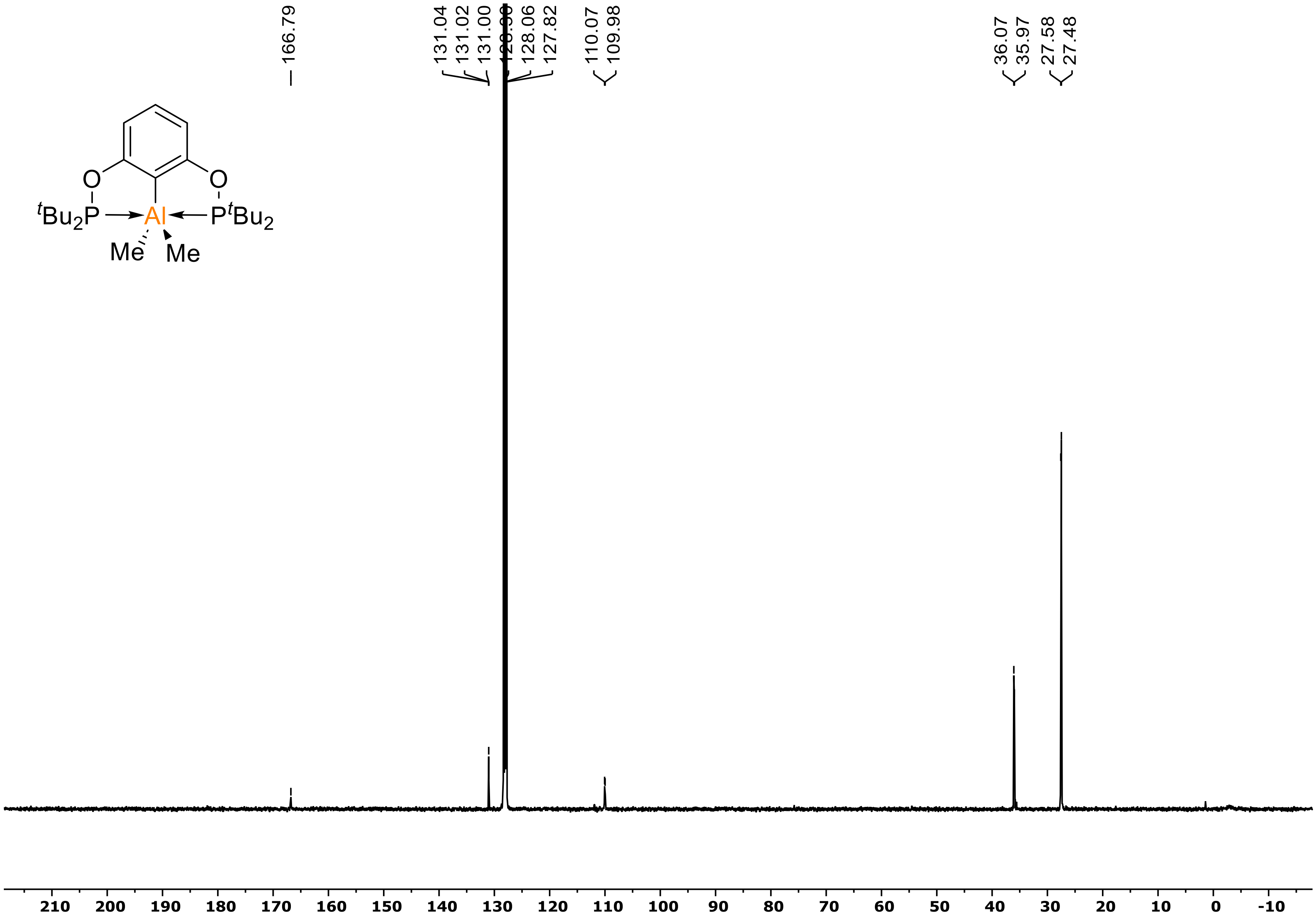

Figure S40: ^{1}H-^{13}C HMBC NMR spectrum of **5**/**5'** and [^{tBu}POCOP]AlMeCl (C_6D_6, 298 K).

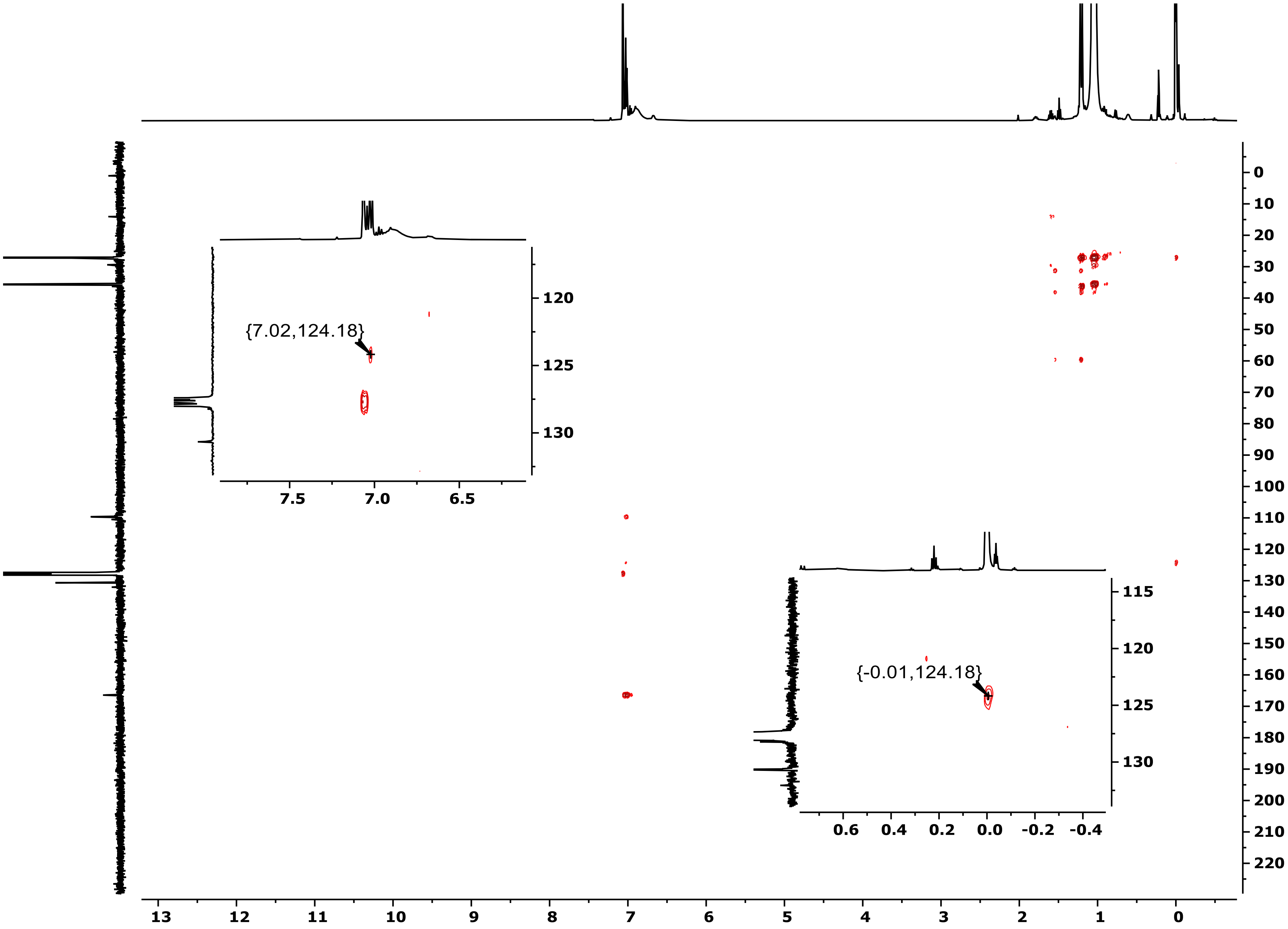

Figure S41: ^{31}P NMR spectrum of **5/5'** and [^{tBu}POCOP]AlMeCl (162 MHz, C_6D_6, 298 K).

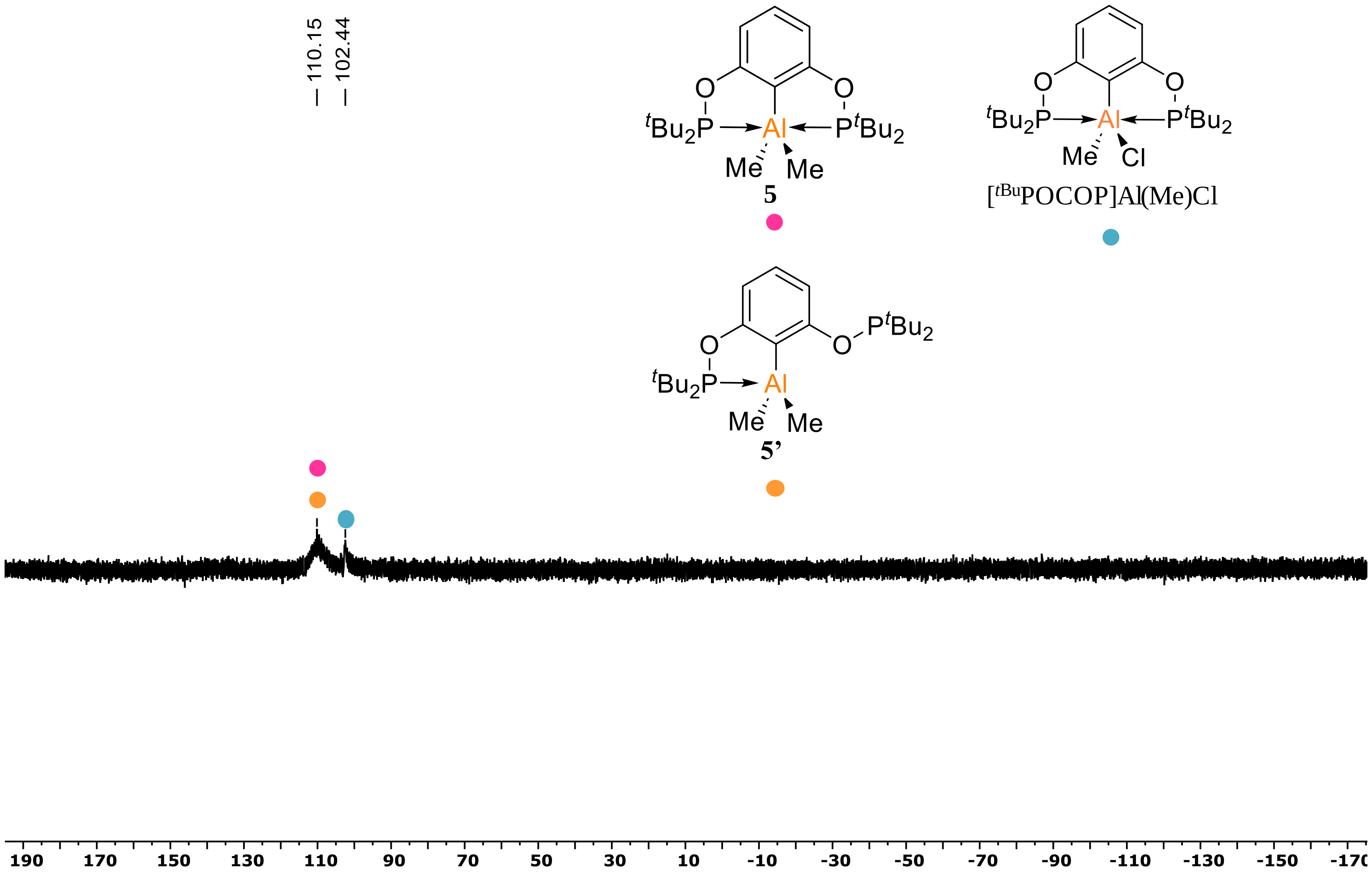

Figure S42: $^{31}P\{^1H\}$ NMR spectrum of **5**/**5'** and [^{tBu}POCOP]AlMeCl (162 MHz, C_6D_6, 298 K).

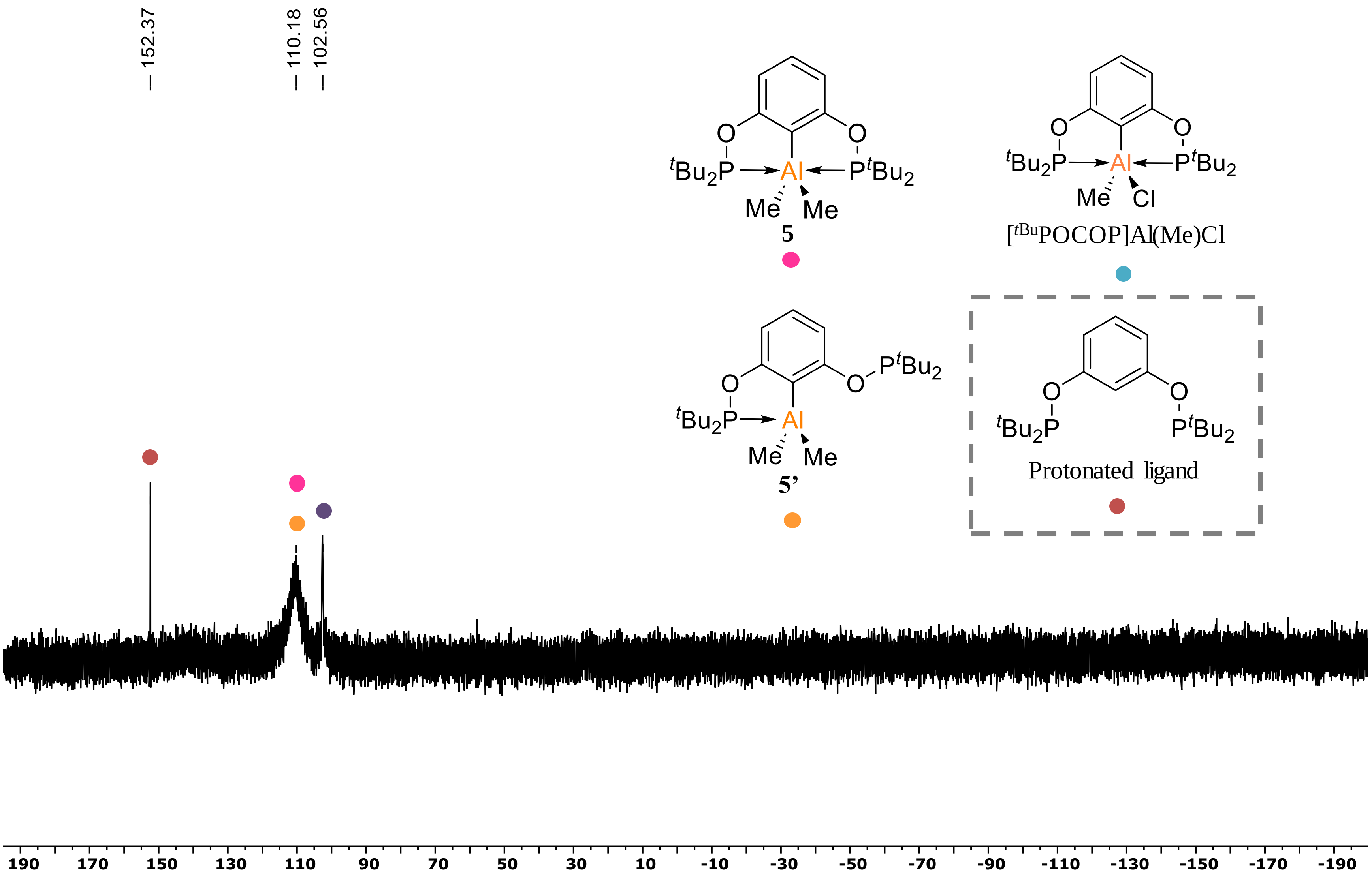

Figure S43: ^{1}H NMR spectra of **5**/**5'** and [^{tBu}POCOP]AlMeCl (162 MHz, Tol-d_8, 211 K (orange), 223 K (brown), 232 K (olive green), 243 K (forest green), 253 K (teal), 263 K (dark blue), 274 K (purple), 284 K (magenta)).

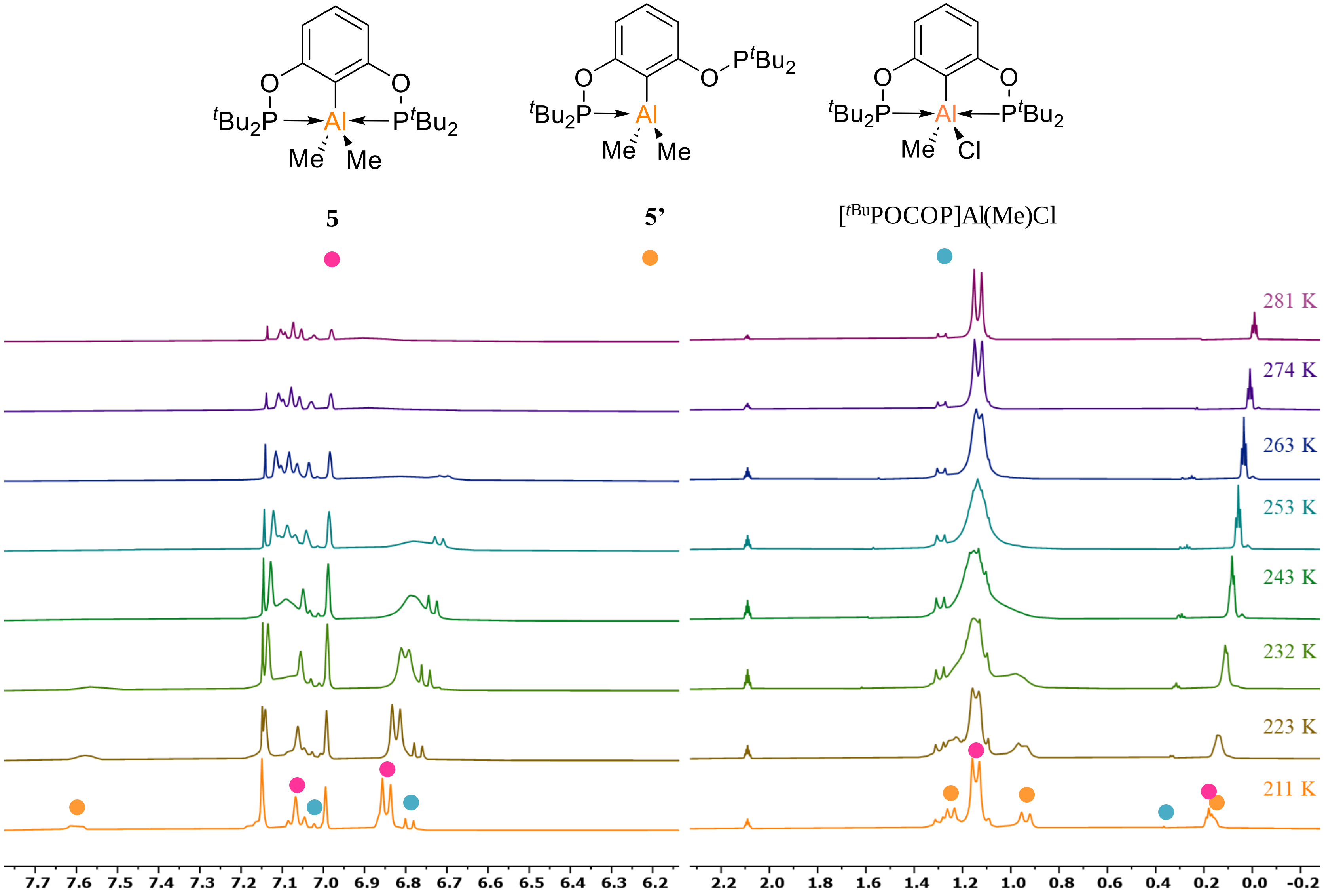

Figure S44: ^{1}H NMR spectra of **5**/**5’** and [^{tBu}POCOP]AlMeCl before and after VT experiments (400 MHz, Tol-d_8, 293 K (orange) and 281 K (teal)).

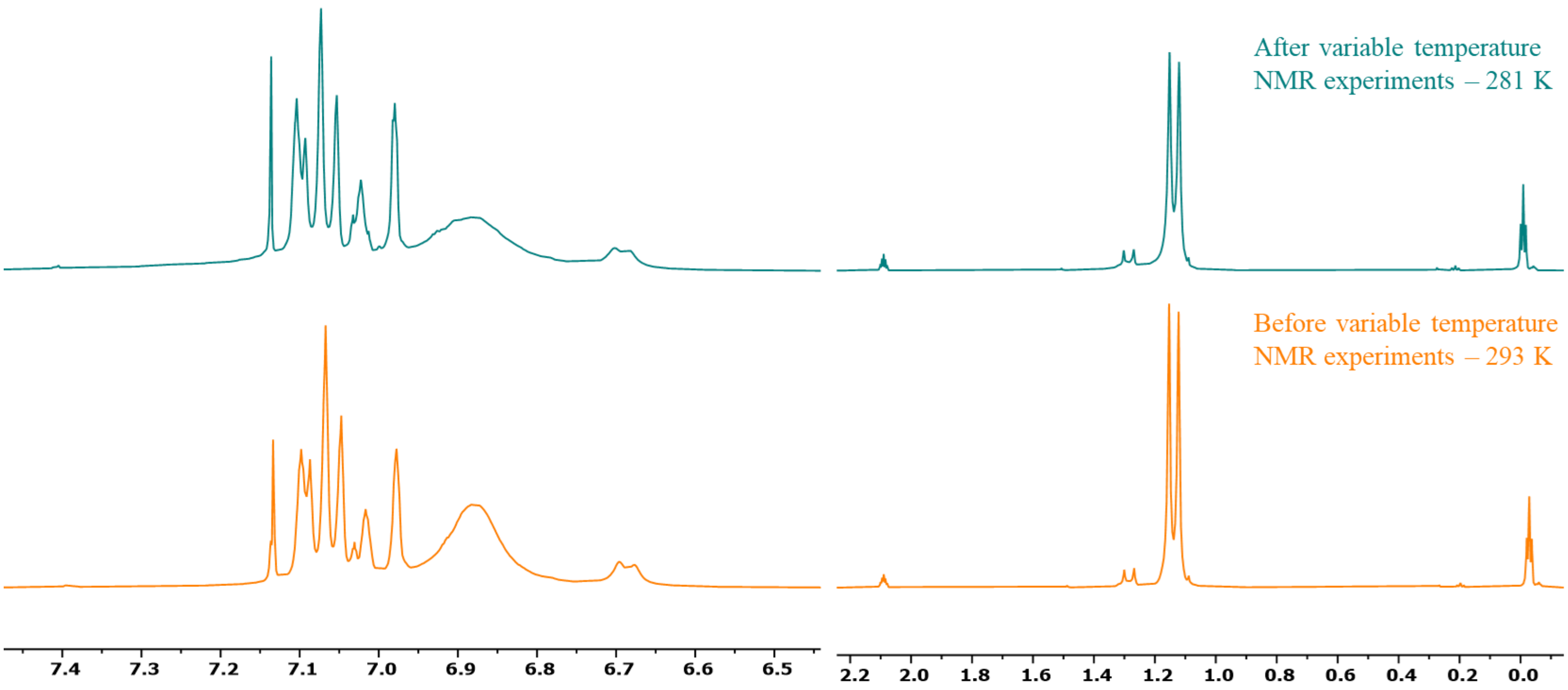

Figure S45: $^{31}P\{^{1}H\}$ NMR spectra of **5**/**5'** and [^{tBu}POCOP]AlMeCl (162 MHz, Tol-d_8, 211 K (orange), 223 K (brown), 232 K (olive green), 243 K (forest green), 253 K (teal), 263 K (dark blue), 274 K (purple), 284 K (magenta)).

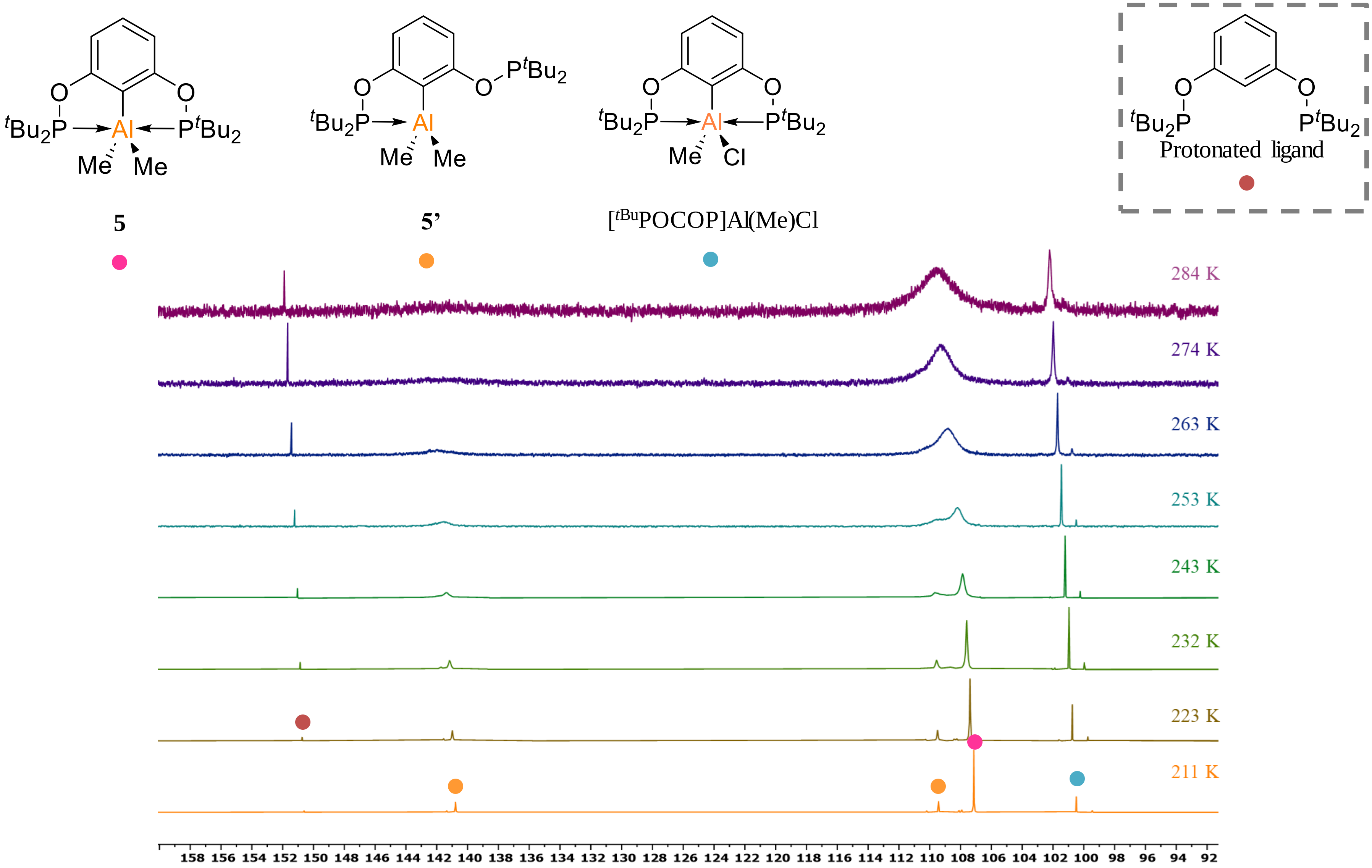

Figure S46: $^{31}P\{^{1}H\}$ NMR spectra of **5**/**5'** and [^{tBu}POCOP]AlMeCl before and after VT experiments. Protonated ligand observed at 152 ppm (162 MHz, Tol-d_8, 293 K (orange) and 284 K (teal)).

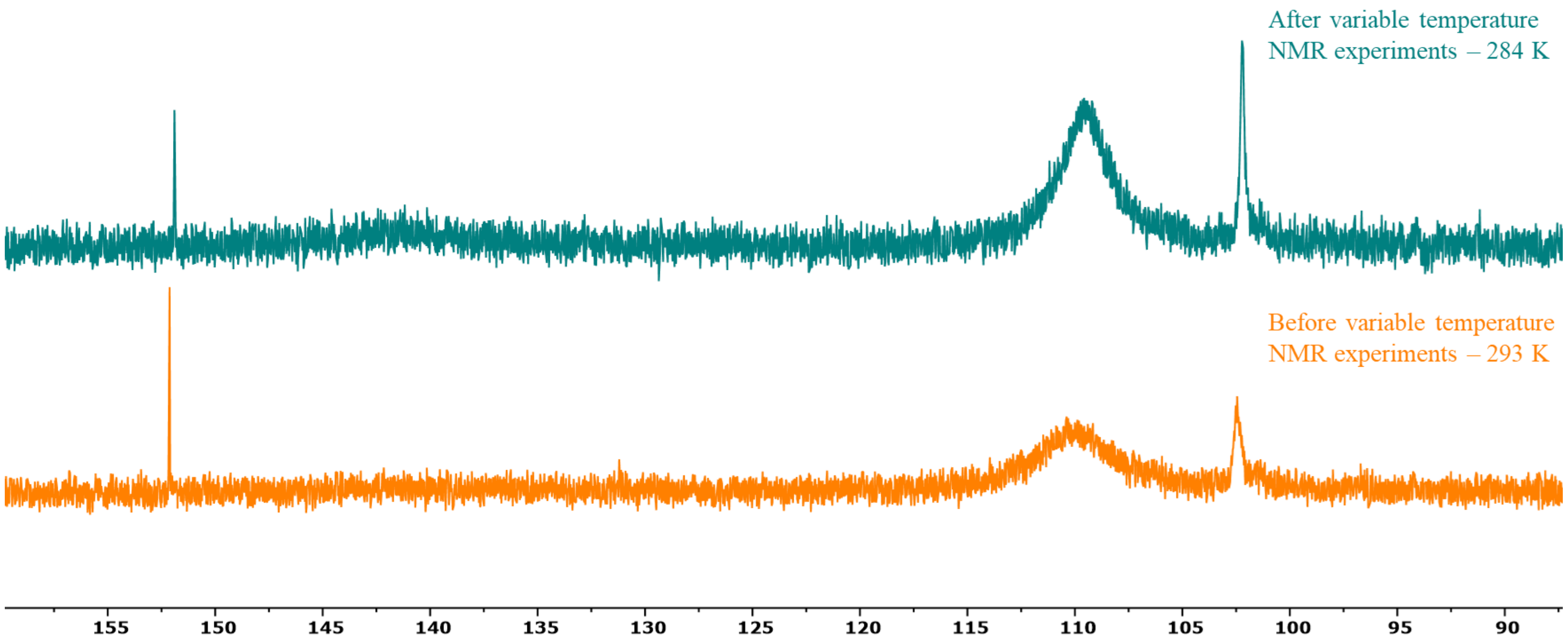

Figure S47: ESI mass spectrum of **5**/**5'**. Peak at 455.2803 corresponds to $[M+H]^+$.

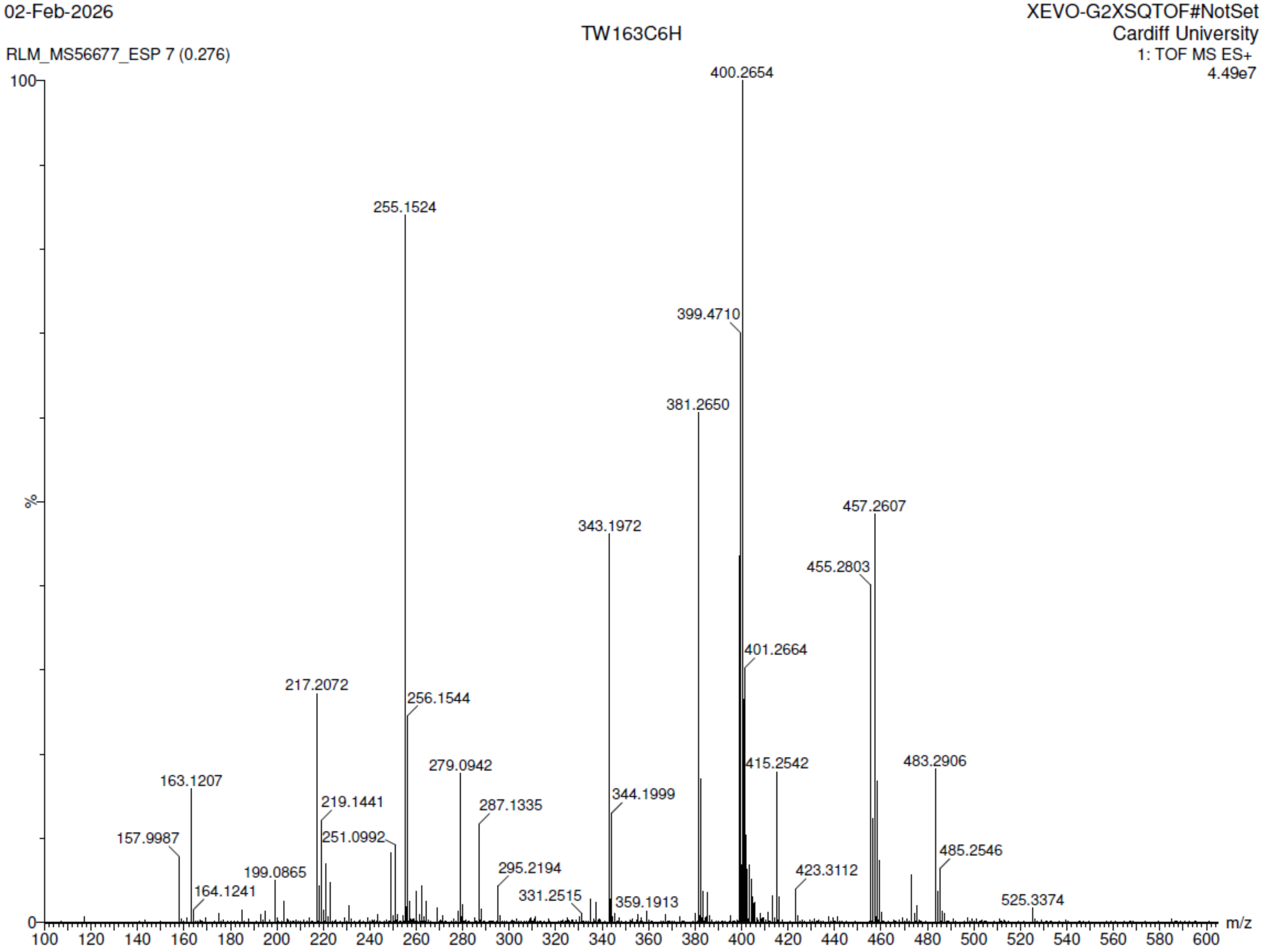

Figure S48: ^{1}H NMR spectrum of **6** (400 MHz, C_6D_6, 298 K).

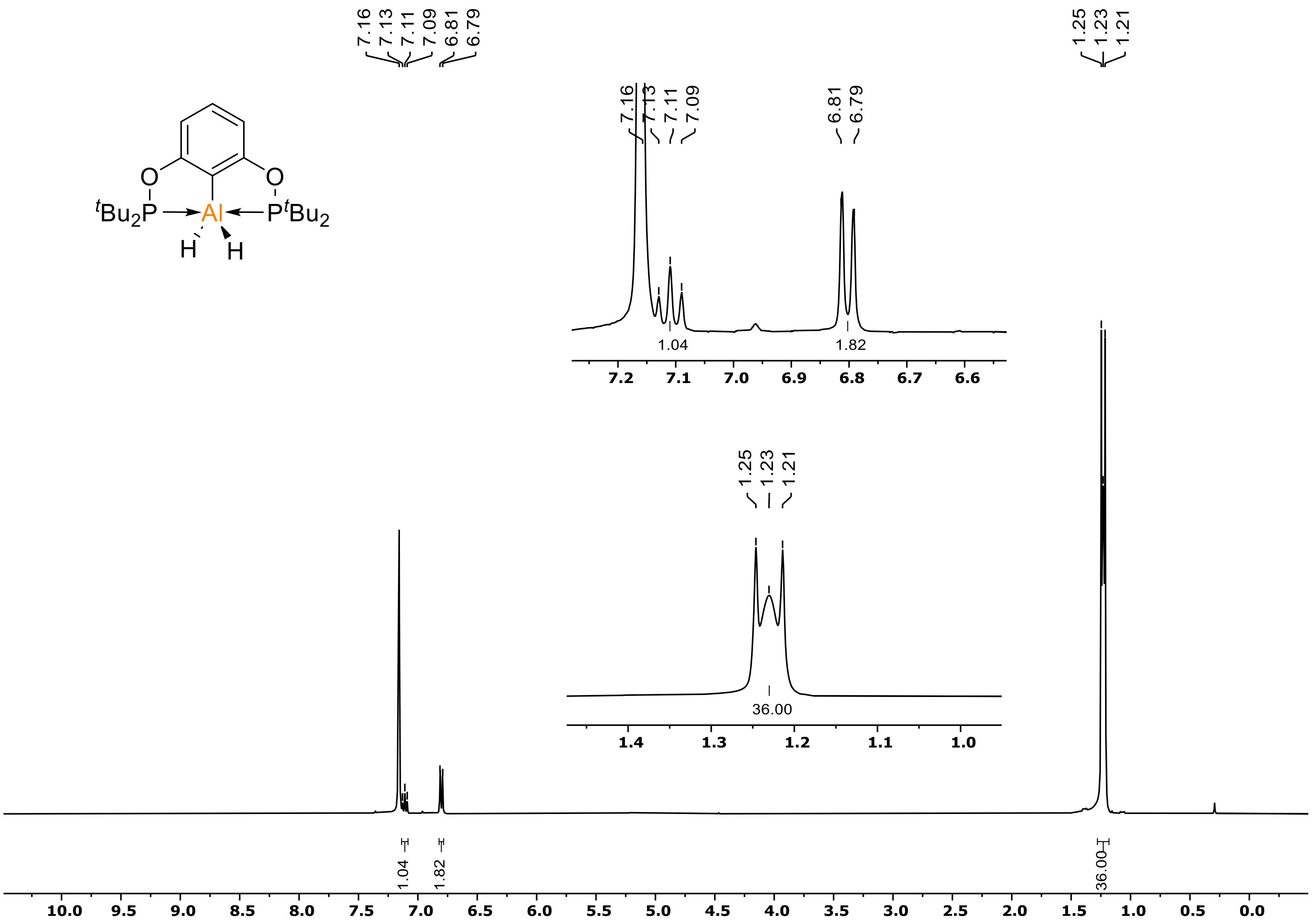

Figure S49: ^{1}H NMR spectrum of **6** (400 MHz, Tol-d_8, 203 K).

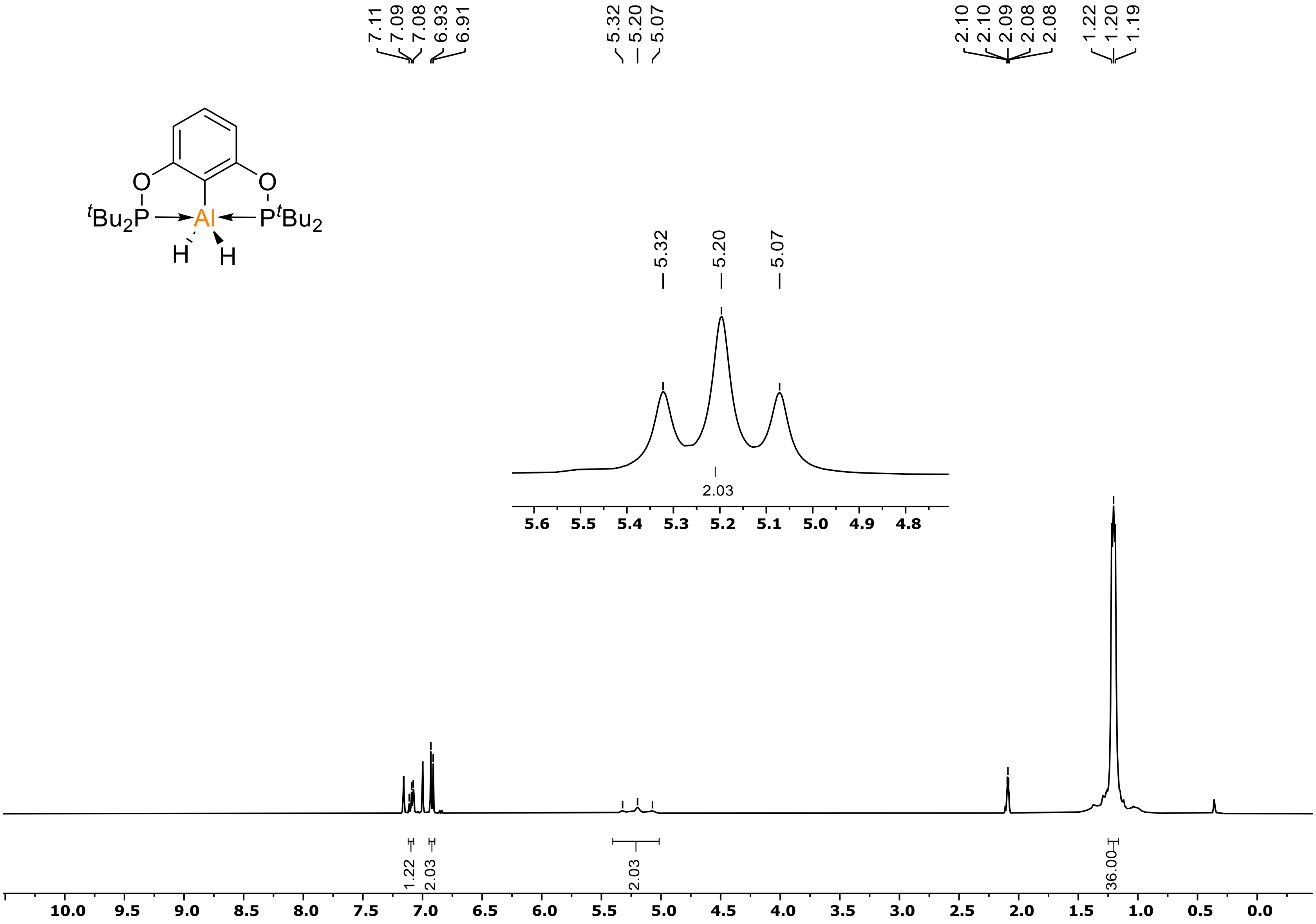

Figure S50: $^{13}C\{^1H\}$ NMR spectrum of **6** (101 MHz, C_6D_6, 298 K).

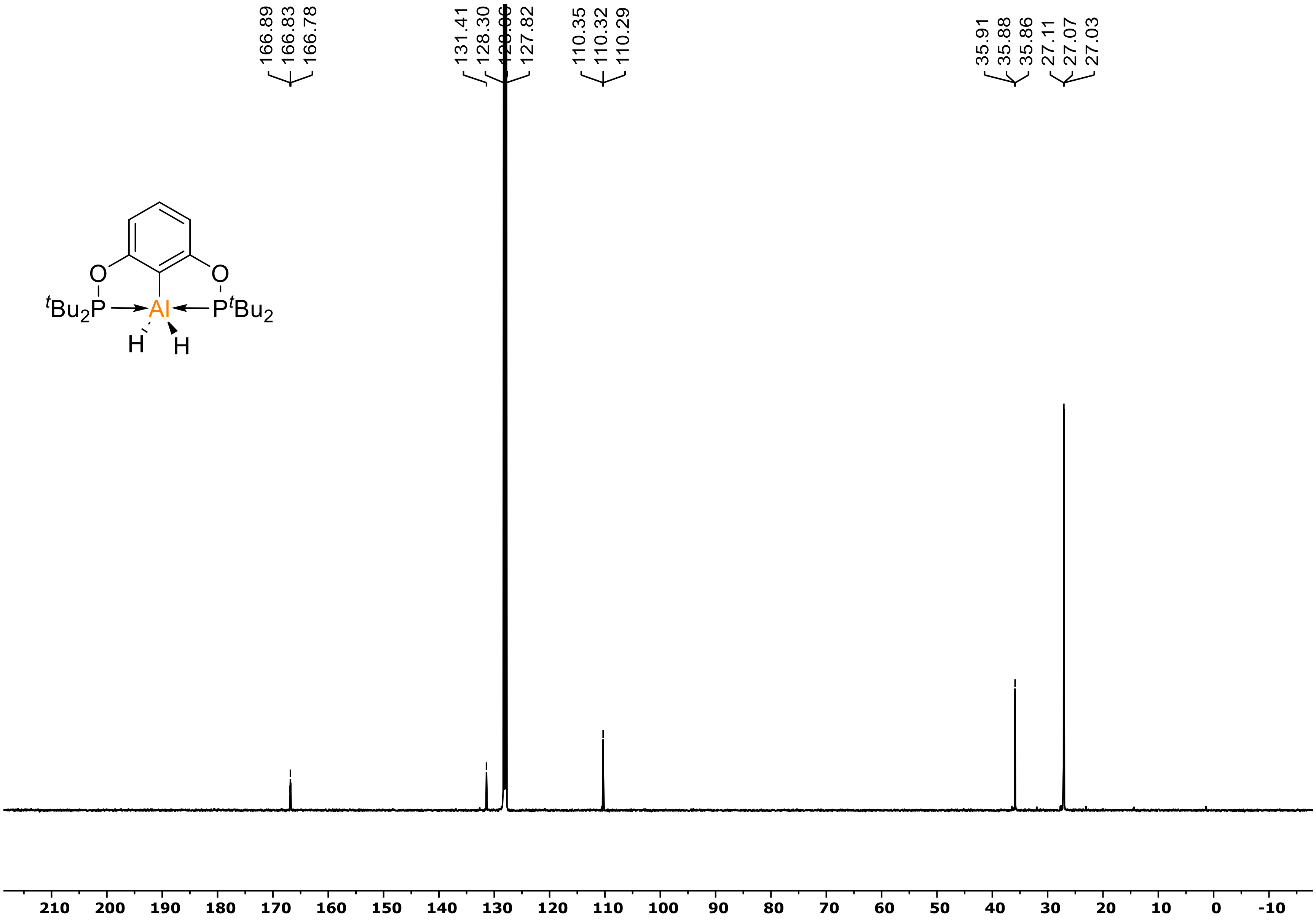

Figure S51: ^{1}H-^{13}C HMBC NMR spectrum of **6** (C_6D_6, 298 K).

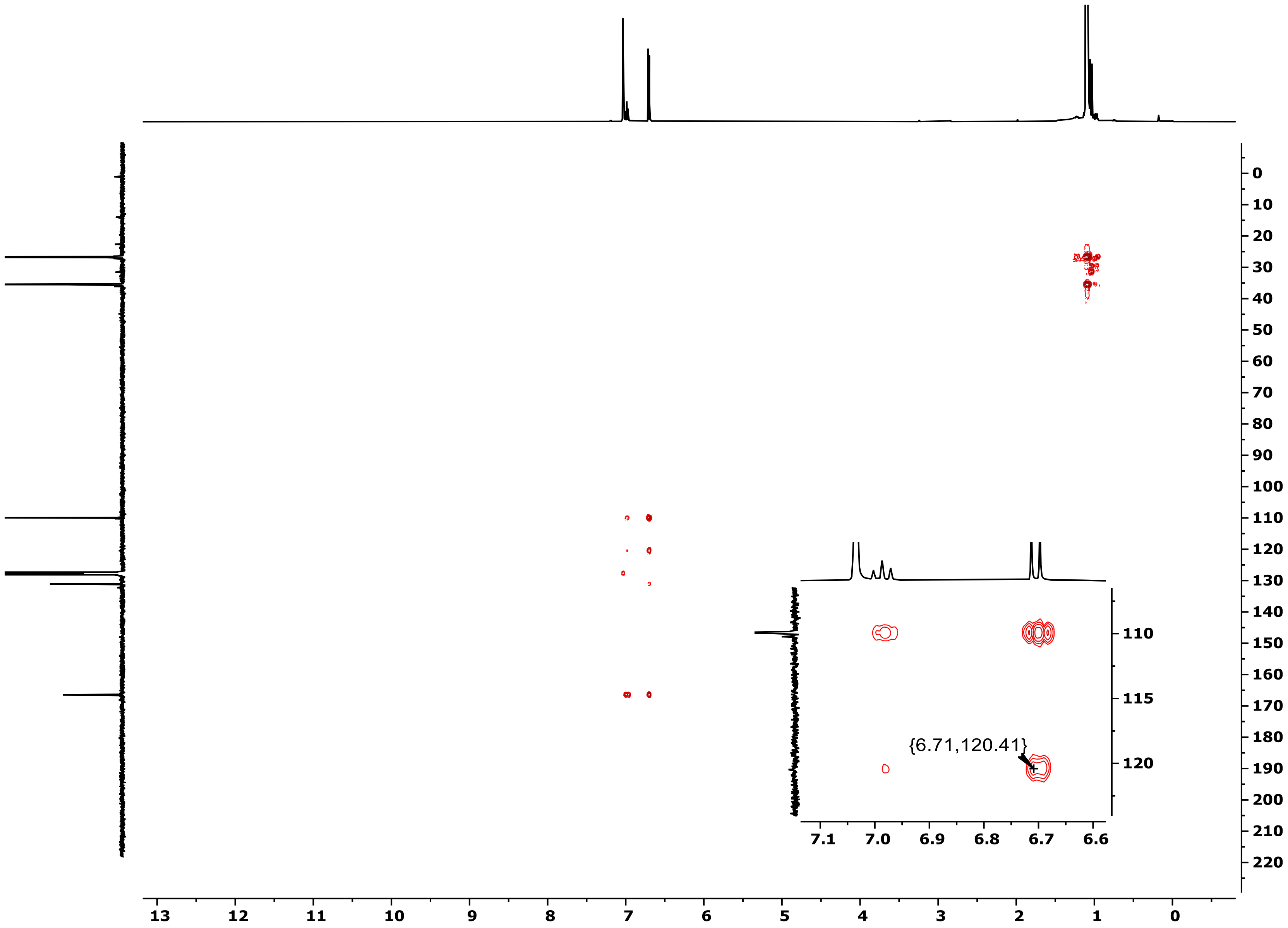

Figure S52: ${}^{31}P$ NMR spectrum of **6** (162 MHz, C_6D_6, 298 K).

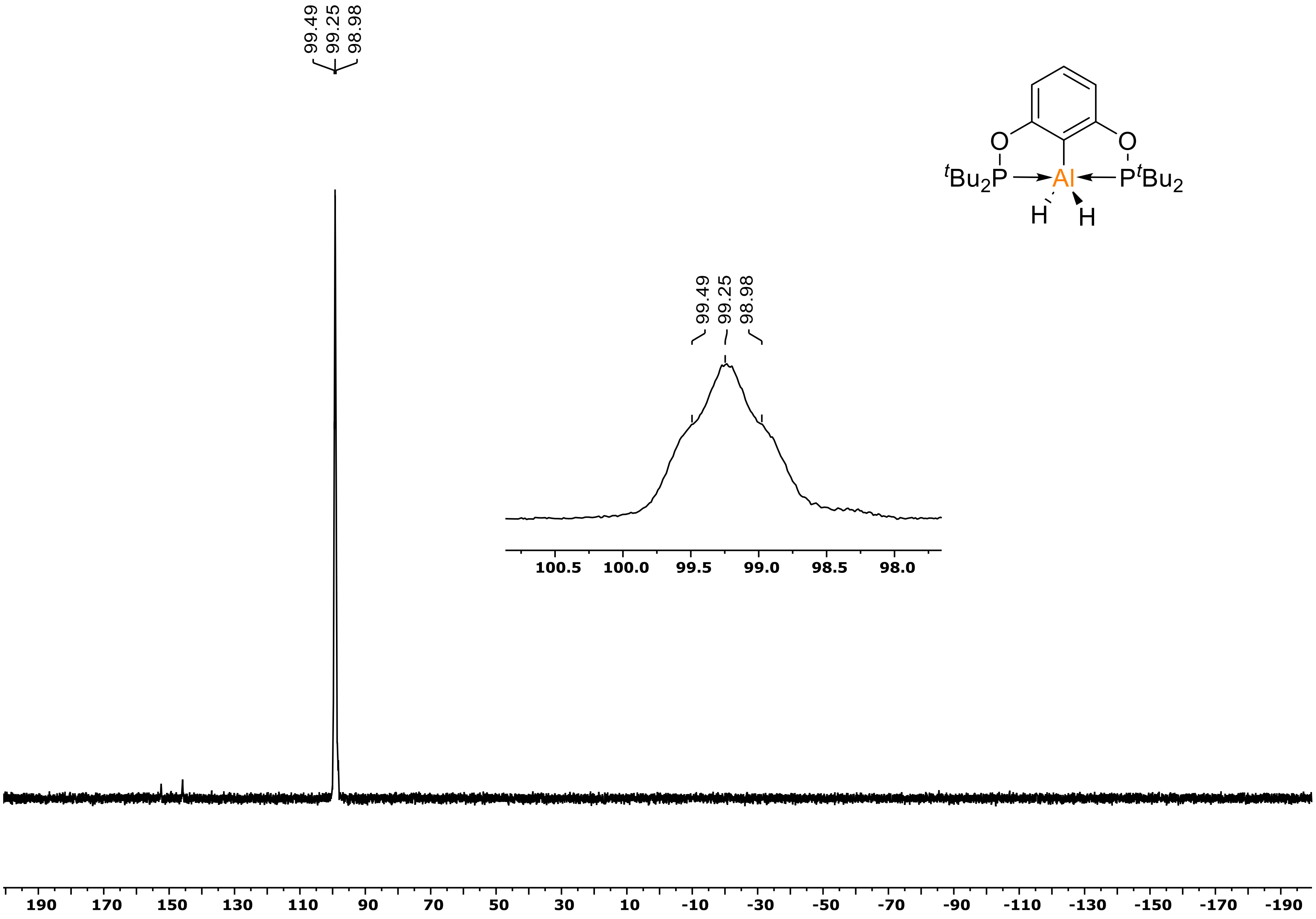

Figure S53: $^{31}P\{^{1}H\}$ NMR spectrum of **6** (162 MHz, C_6D_6, 298 K).

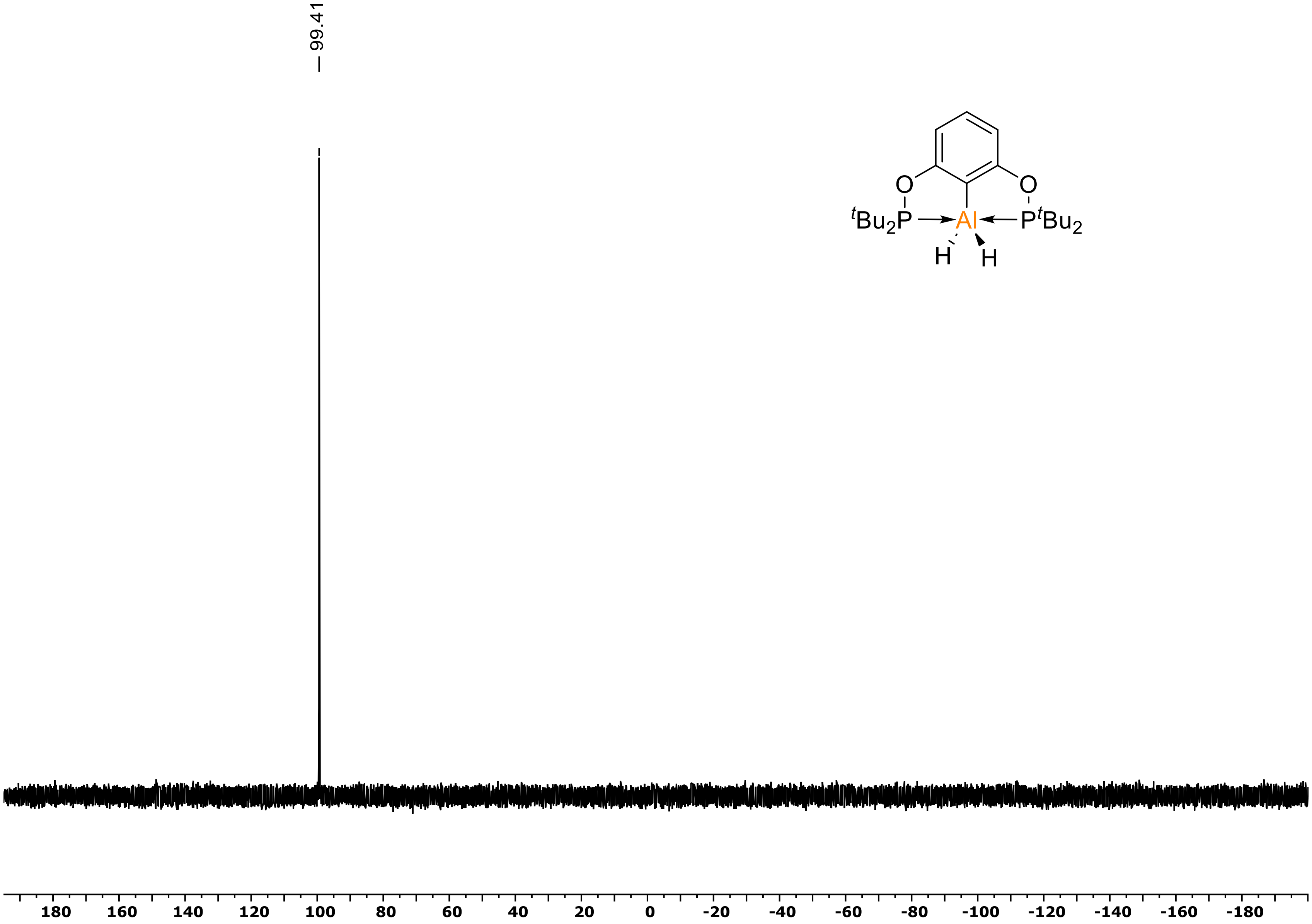

Figure S54: ^{1}H NMR spectrum of a mixture of **2**, **6** and **7** (400 MHz, C_6D_6, 298 K).

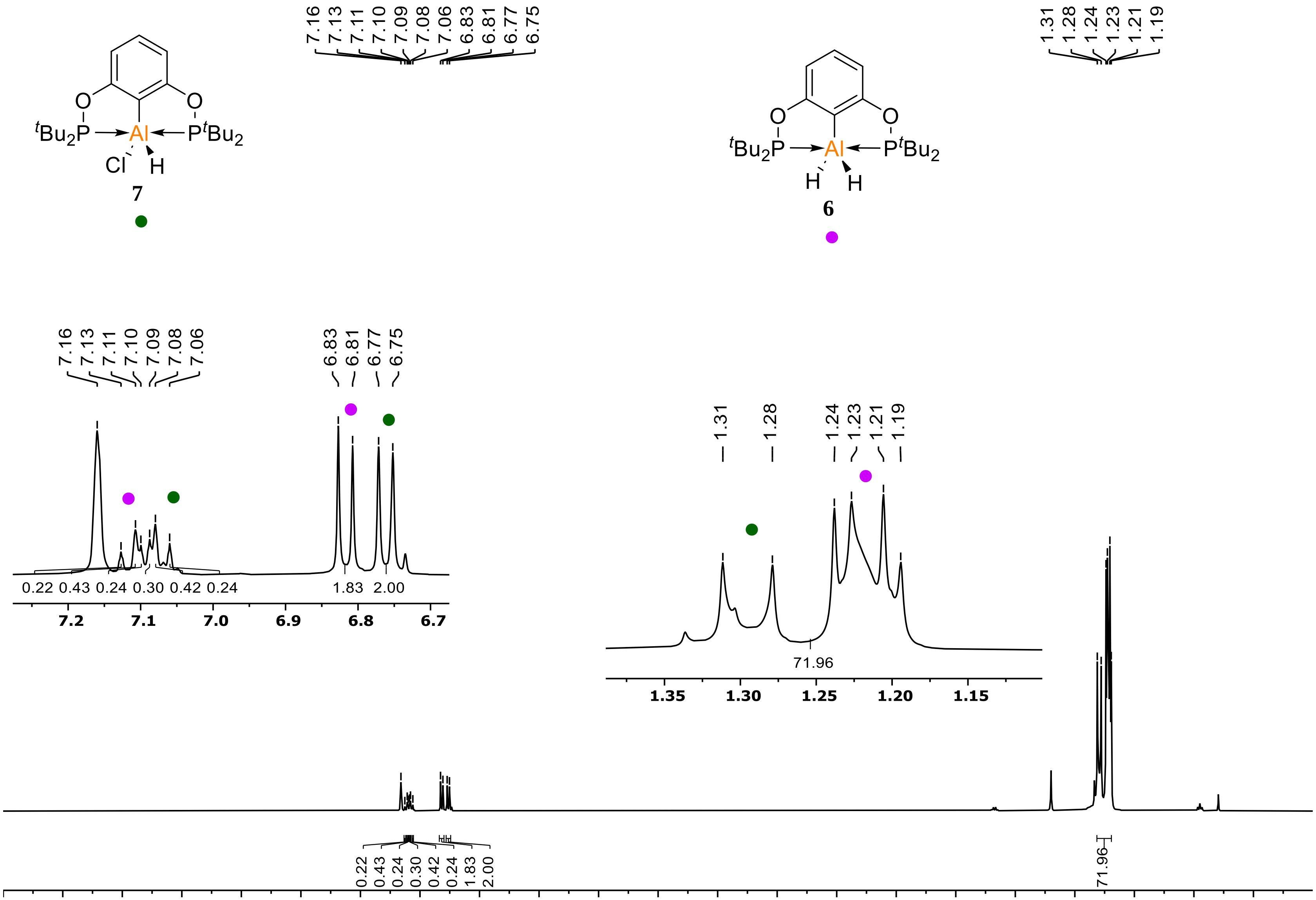

Figure S55: ^{1}H NMR spectra of a mixture of **2**, **6** and **7** (red) & a spectrum of independently synthesized **6** (blue) superimposed (400 MHz, C_6D_6, 298 K).

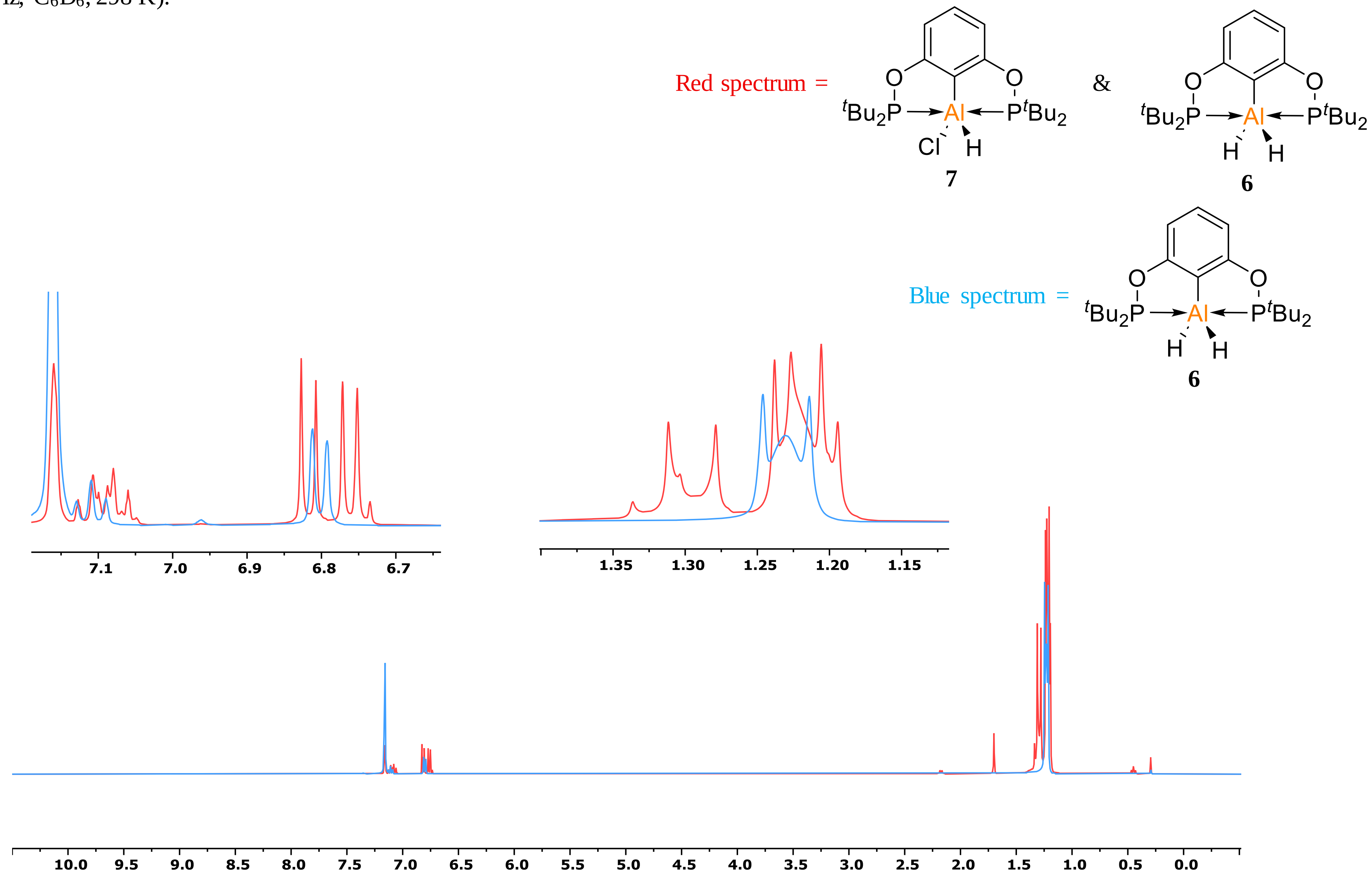

Figure S56: $^{13}C\{^{1}H\}$ NMR spectrum of a mixture of **2**, **6** and **7** (101 MHz, C_6D_6, 298 K).

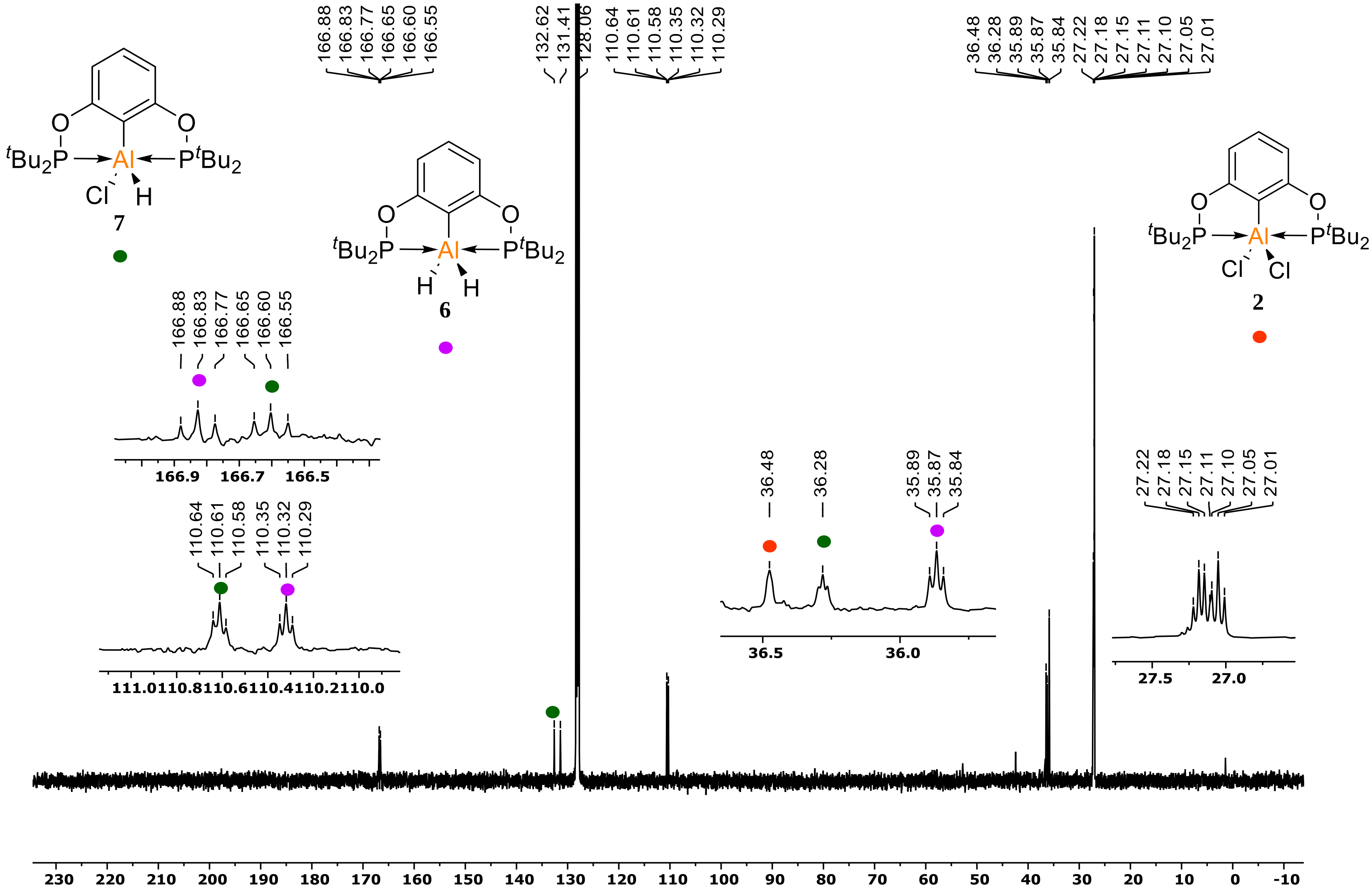

Figure S57: ^{1}H-^{13}C HMBC NMR spectrum of **7** (C_6D_6, 298 K).

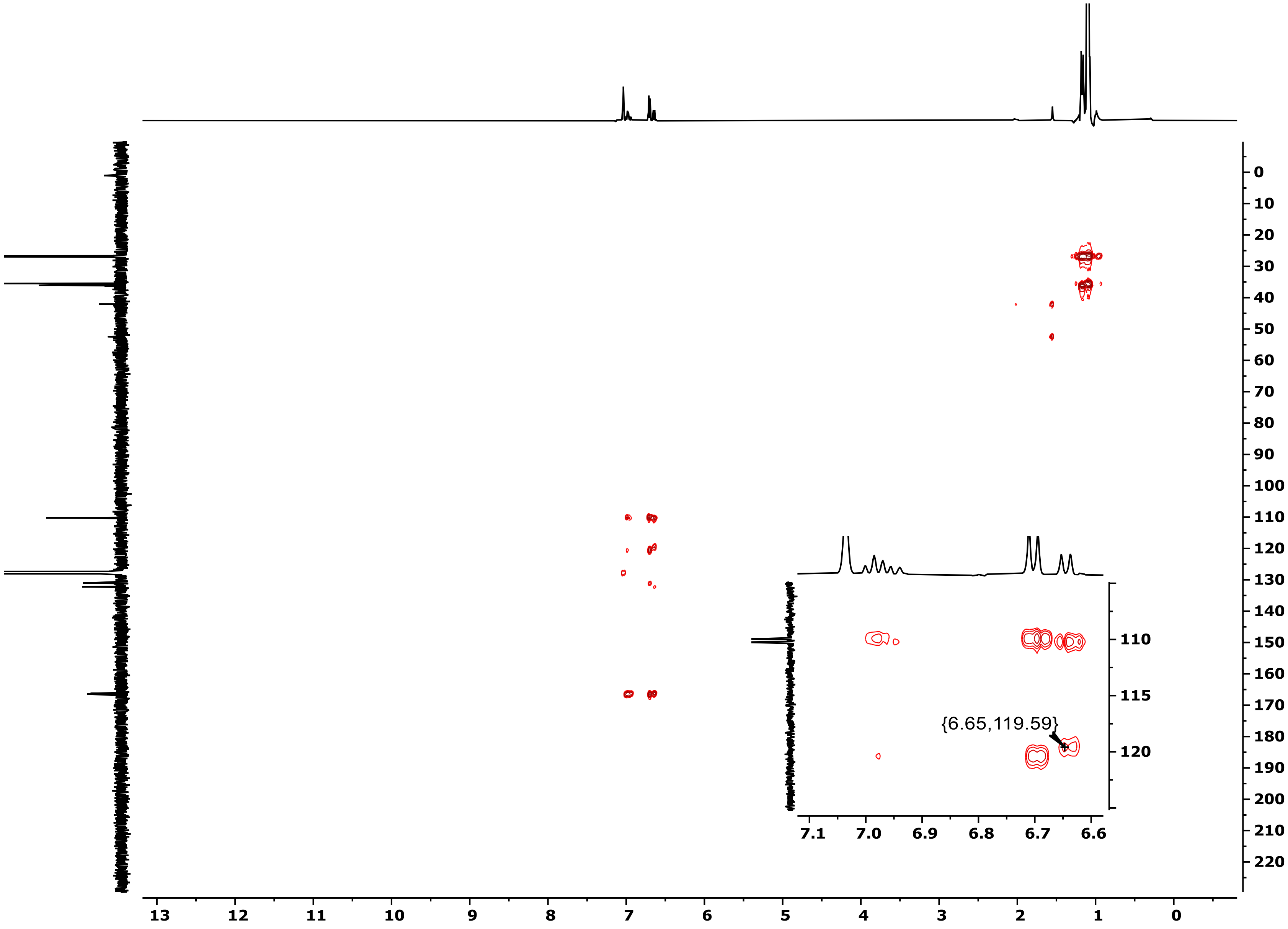

Figure S58: ^{31}P NMR spectrum of a mixture of **2**, **6** and **7** (162 MHz, C_6D_6, 298 K).

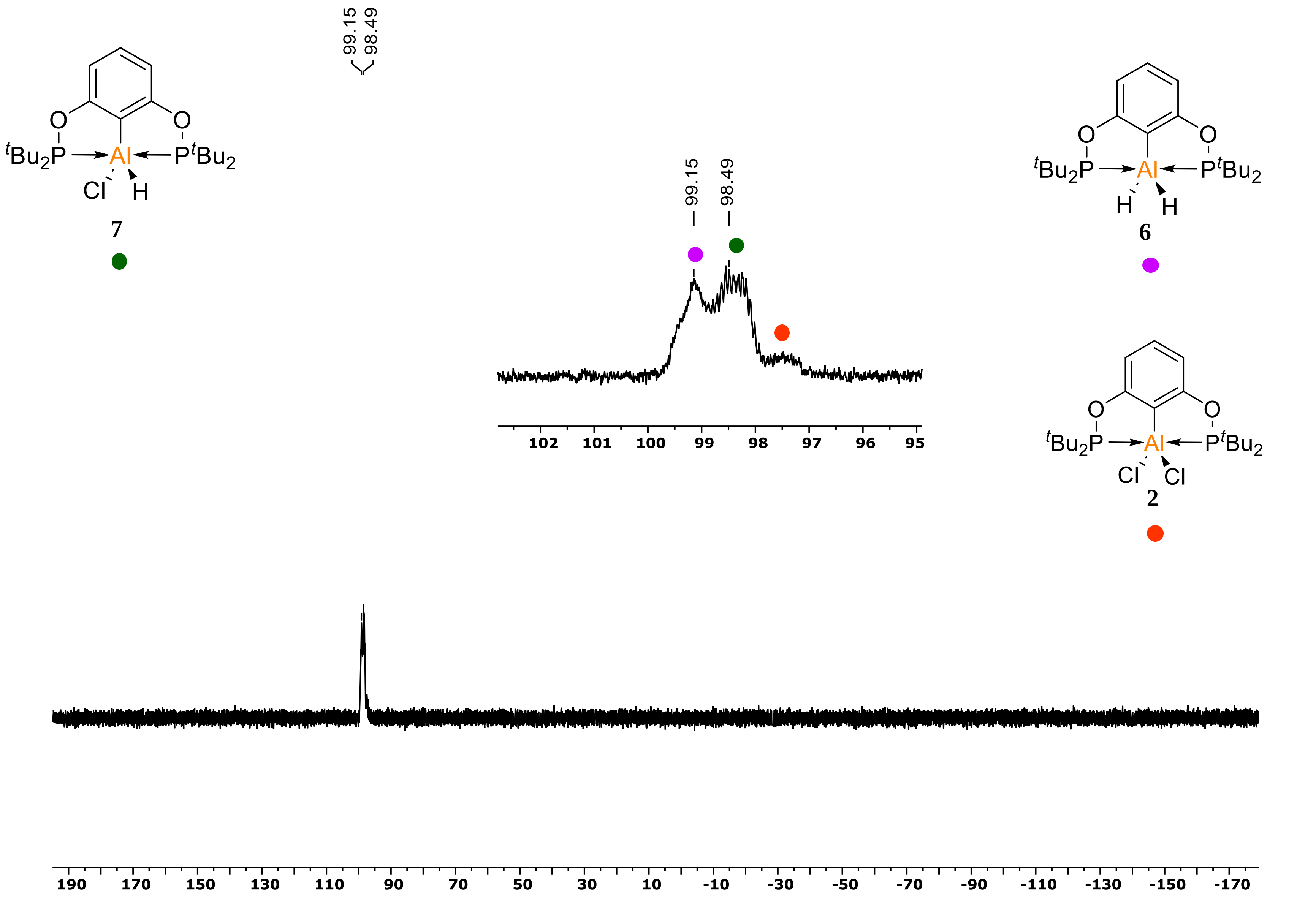

Figure S59: $^{31}P\{^{1}H\}$ NMR spectrum of a mixture of **2**, **6** and **7** (162 MHz, C_6D_6, 298 K).

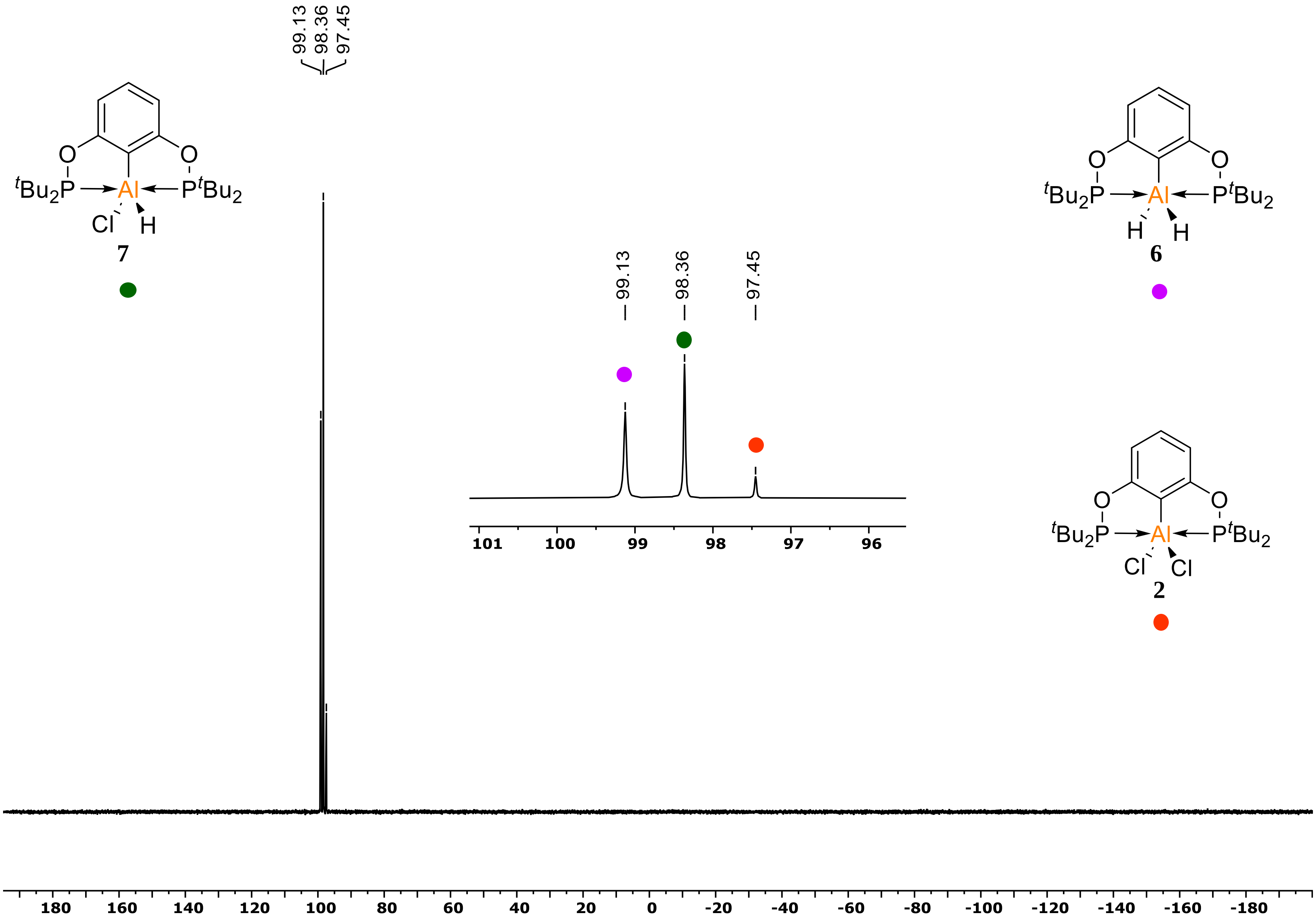

Figure S60: ^{1}H NMR spectrum of **8** (500 MHz, C_6D_6, 298 K).

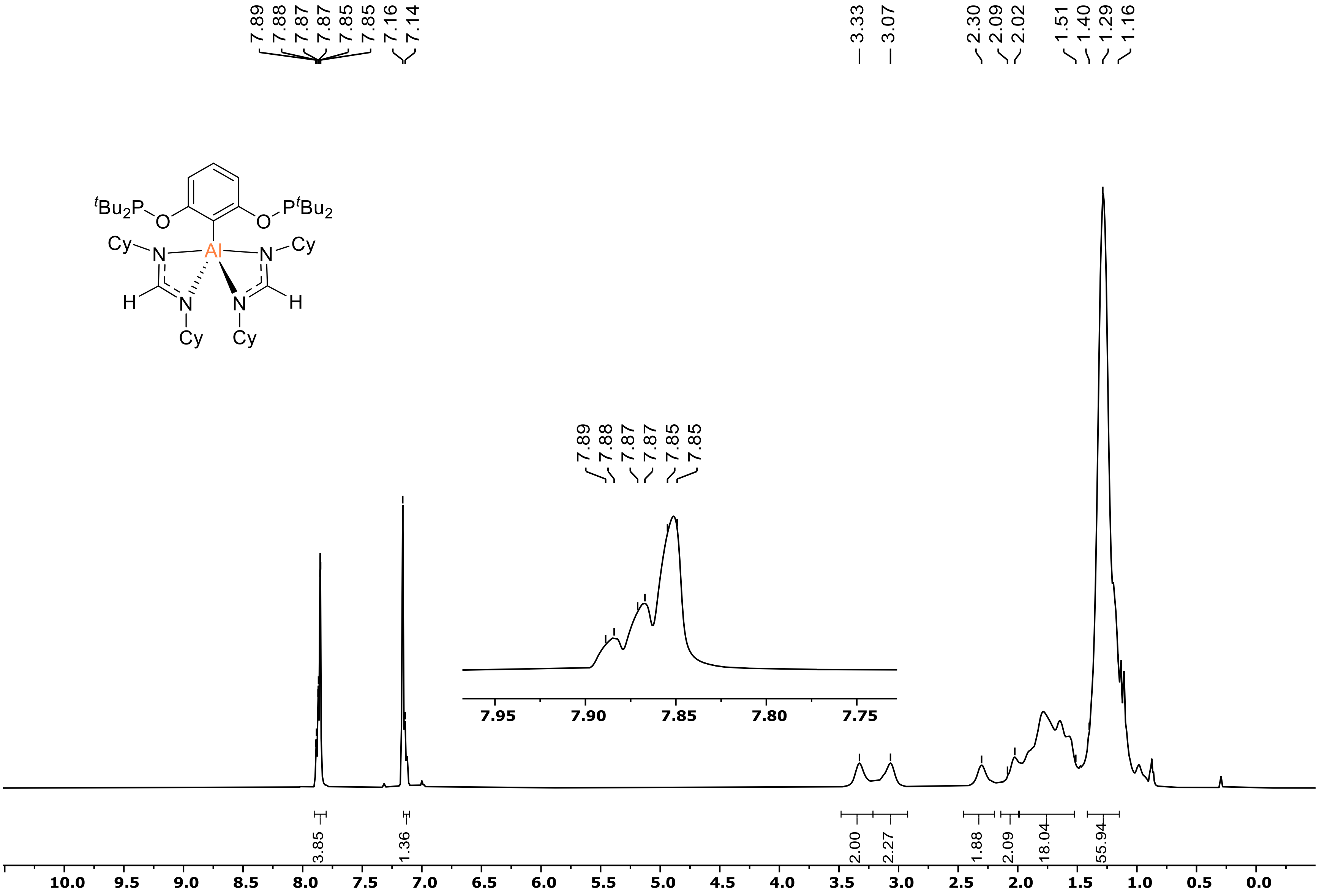

Figure S61: $^{13}C\{^{1}H\}$ NMR spectrum of **8** (126 MHz, C_6D_6, 298 K).

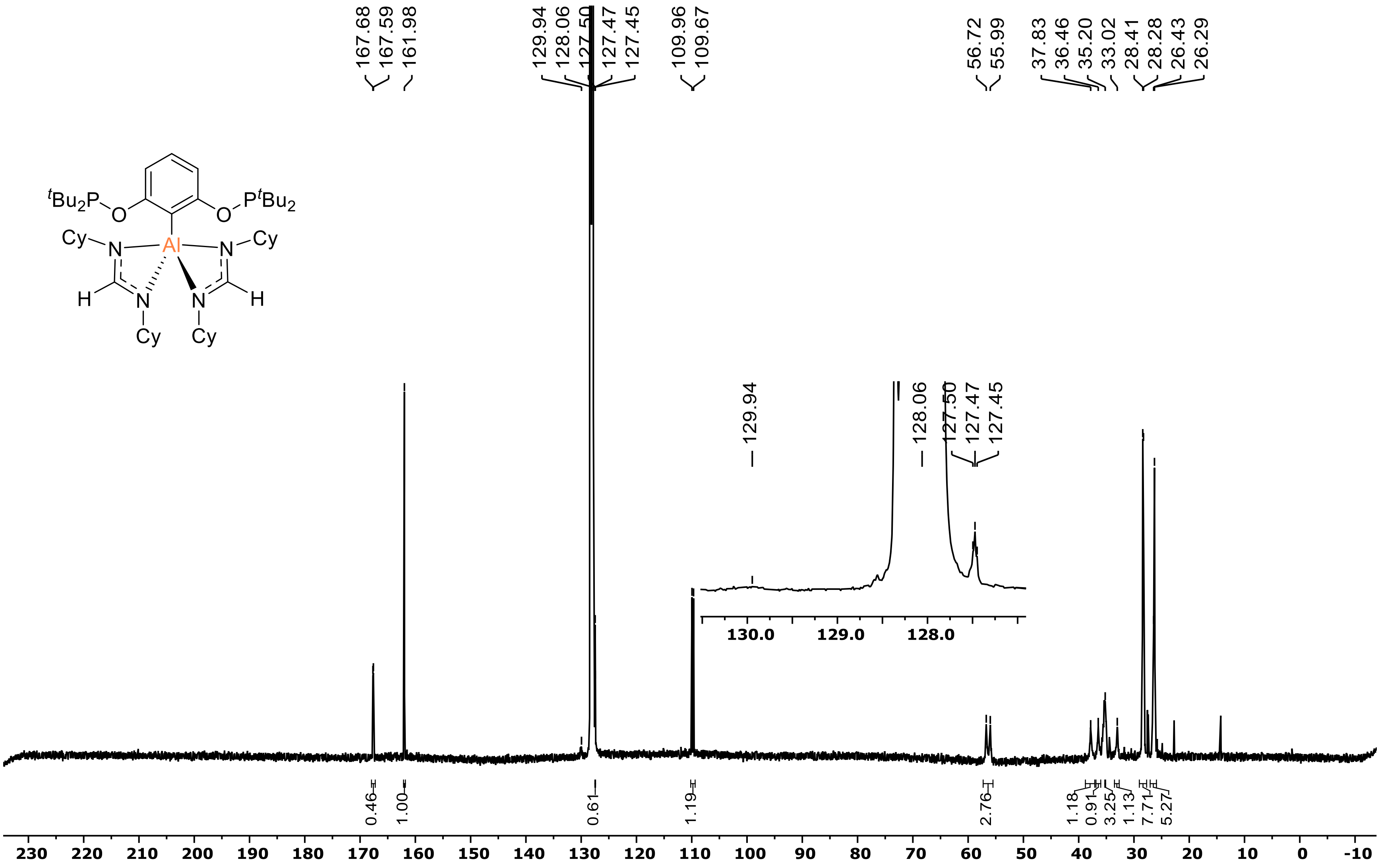

Figure S62: ^{1}H-^{13}C HMBC NMR spectrum of **8** (C_6D_6, 298 K).

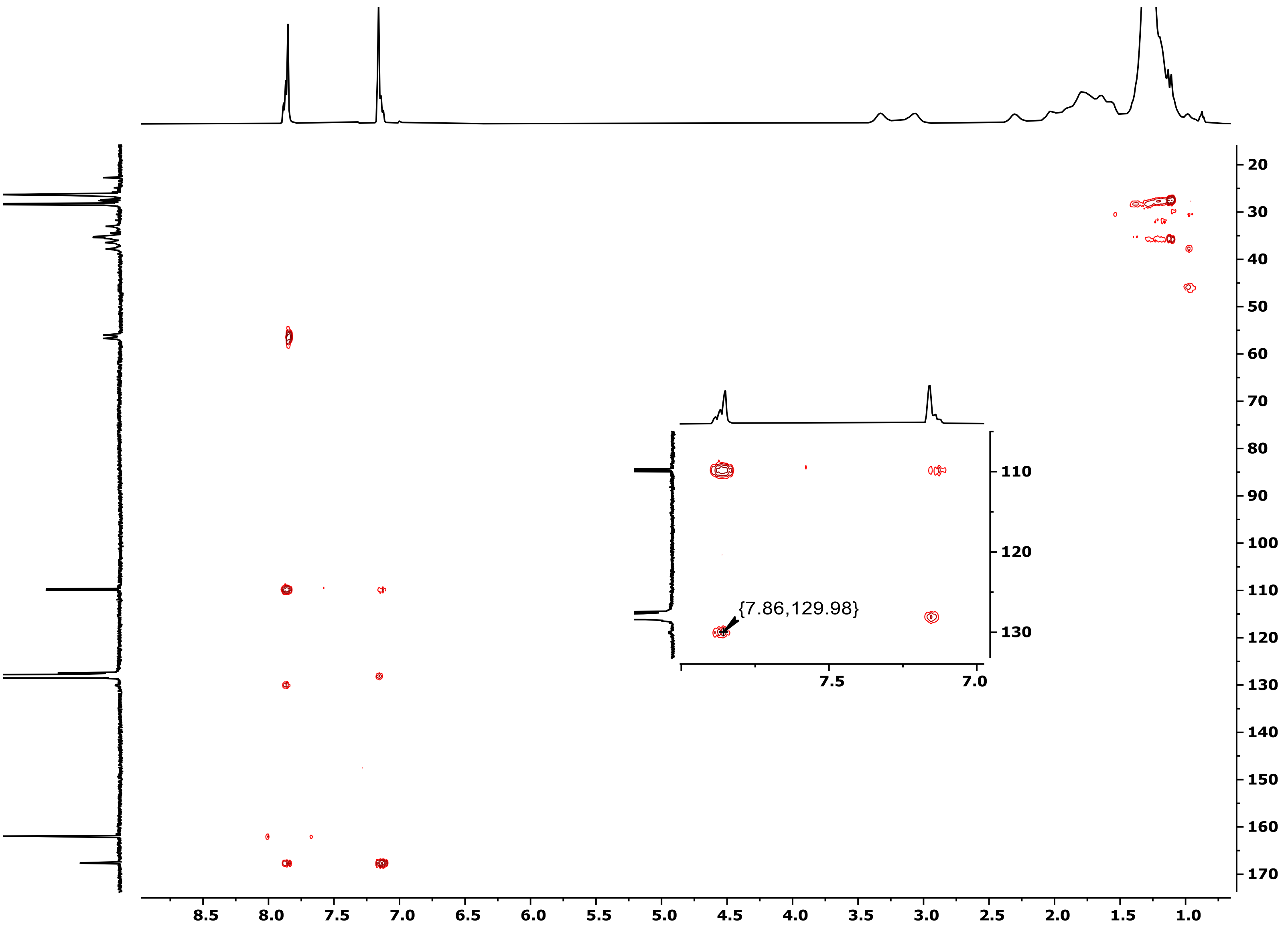

Figure S63: $^{31}P\{^{1}H\}$ NMR spectrum of **8** (202 MHz, C_6D_6, 298 K).

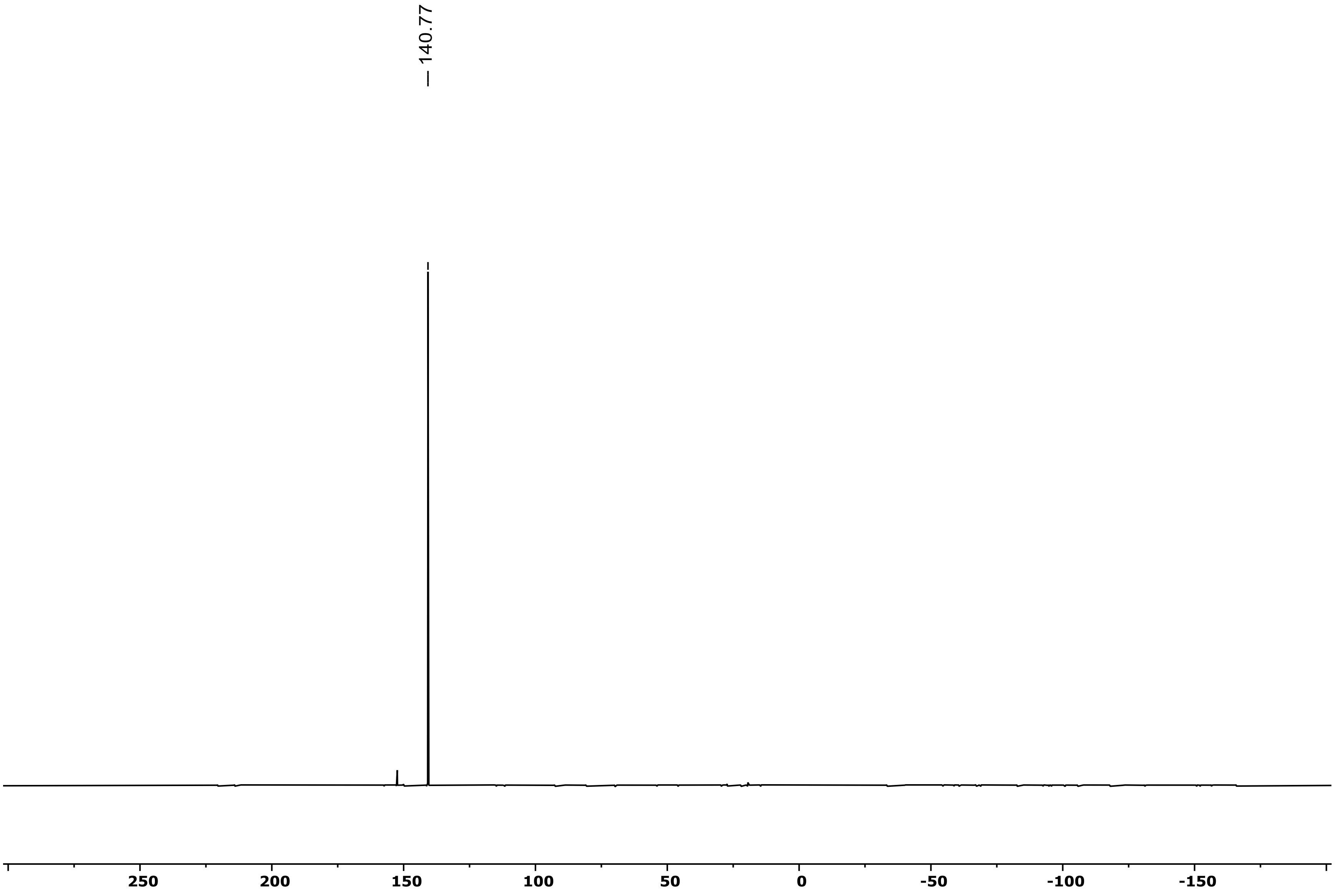

Figure S64: ^{1}H NMR spectrum of the reaction between **6** and 2 equivalents of benzophenone (600 MHz, C_6D_6, 298 K).

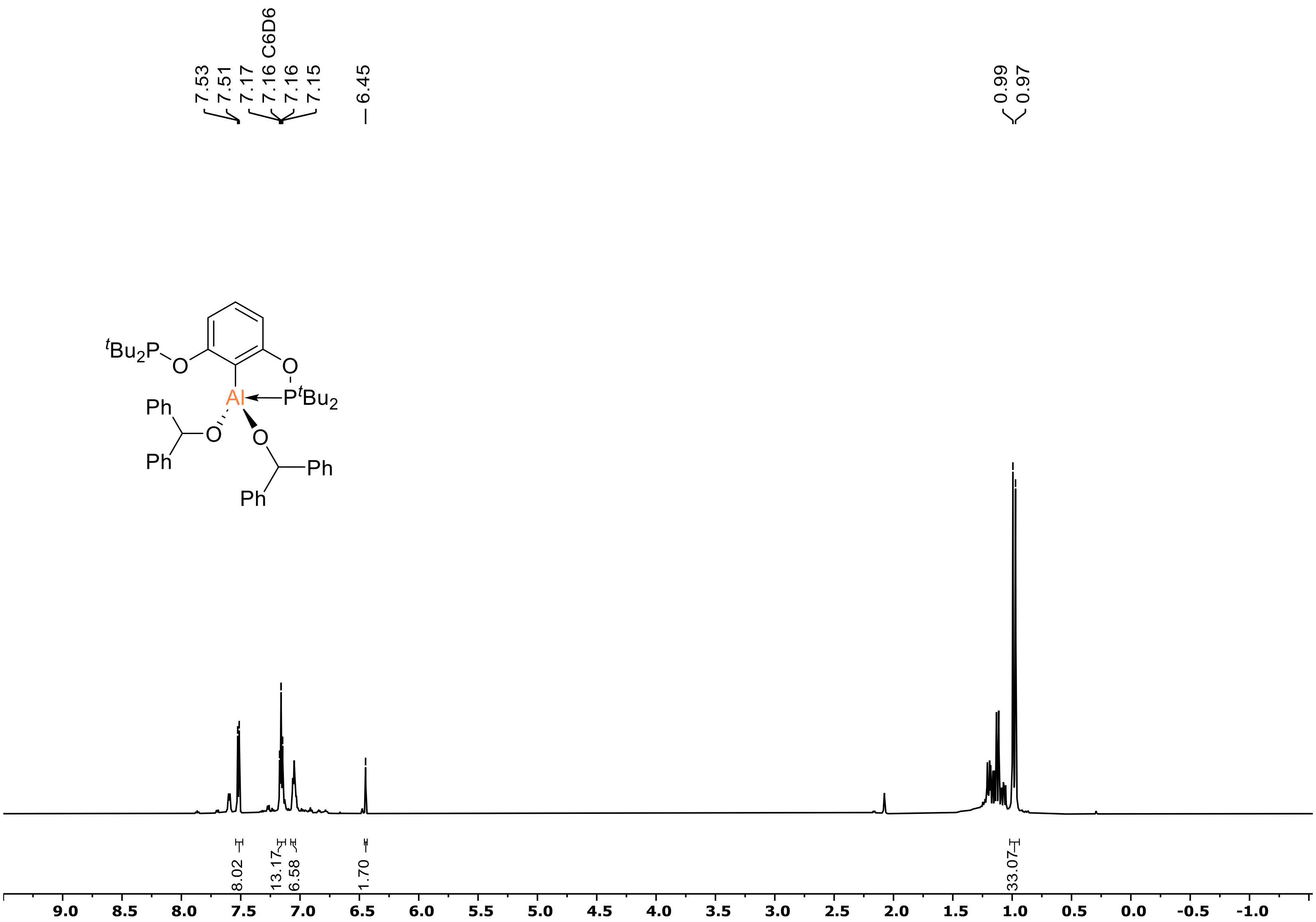

Figure S65: $^{13}C\{^{1}H\}$ NMR spectrum of the reaction between **6** and 2 equivalents of benzophenone (151 MHz, C_6D_6, 298 K).

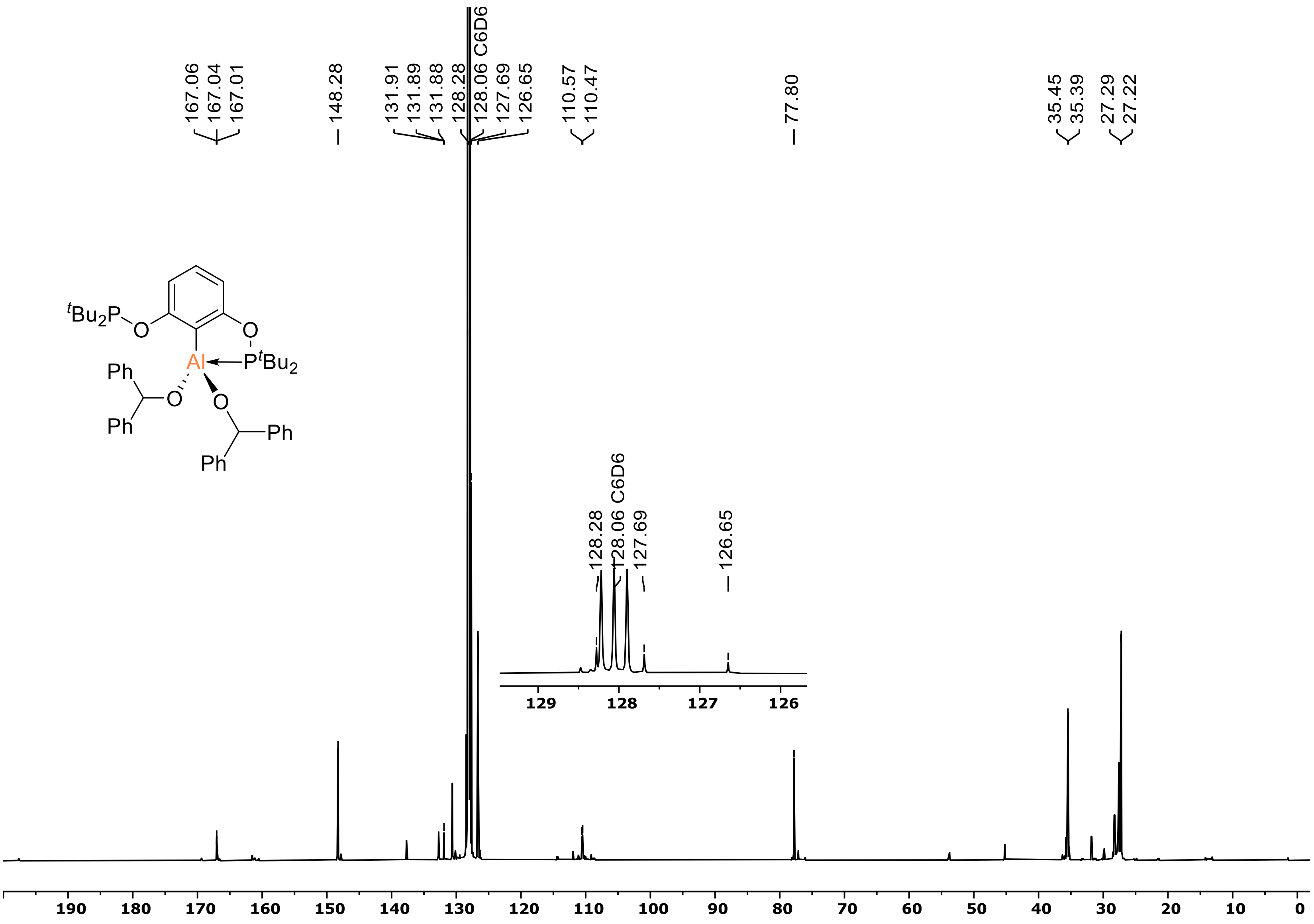

Figure S66: ^{31}P NMR spectrum of the reaction between **6** and 2 equivalents of benzophenone (243 MHz, C_6D_6, 298 K).

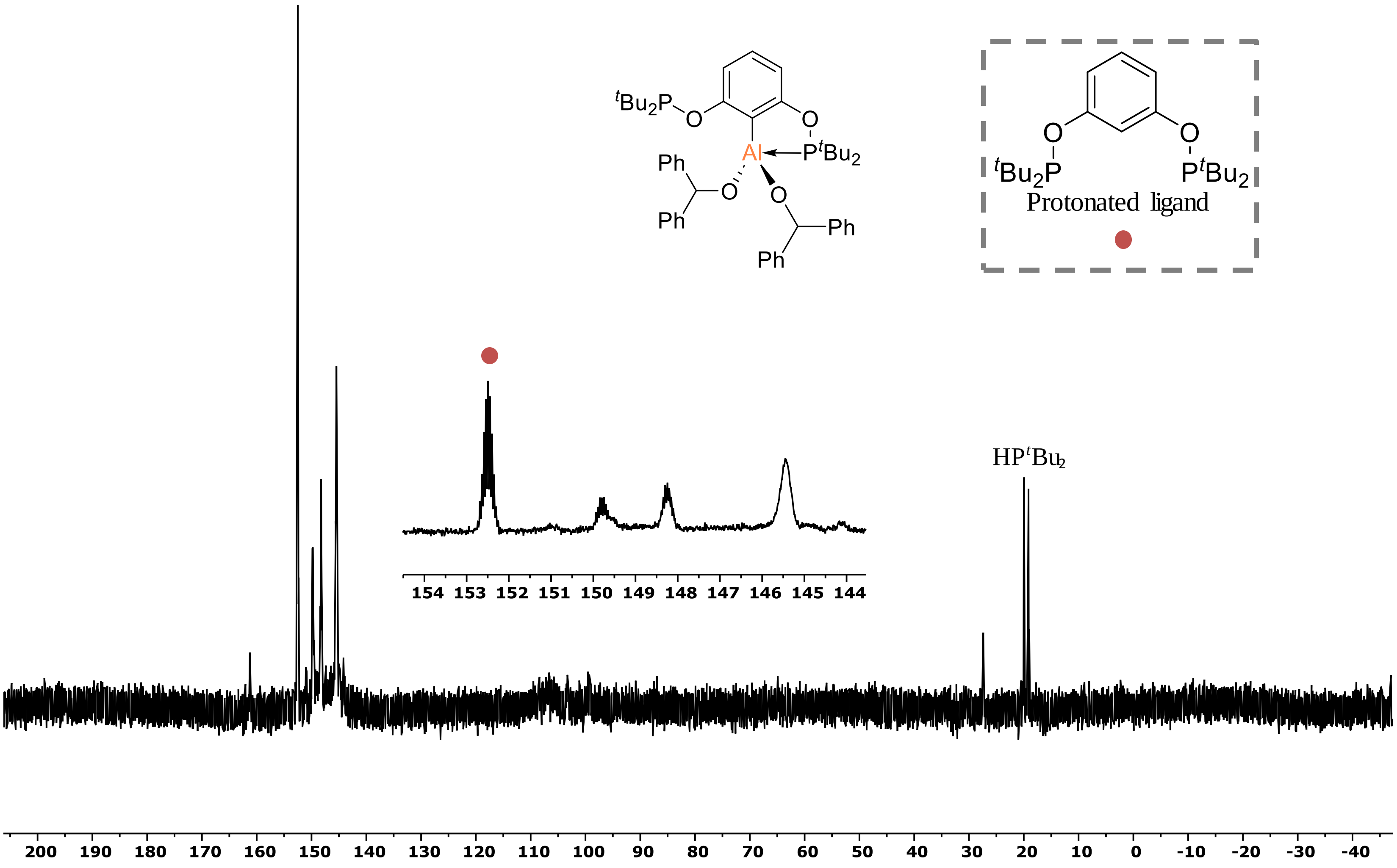

Figure S67: $^{31}P\{^1H\}$ NMR spectrum of the reaction between **6** and 2 equivalents of benzophenone (243 MHz, C_6D_6, 298 K).

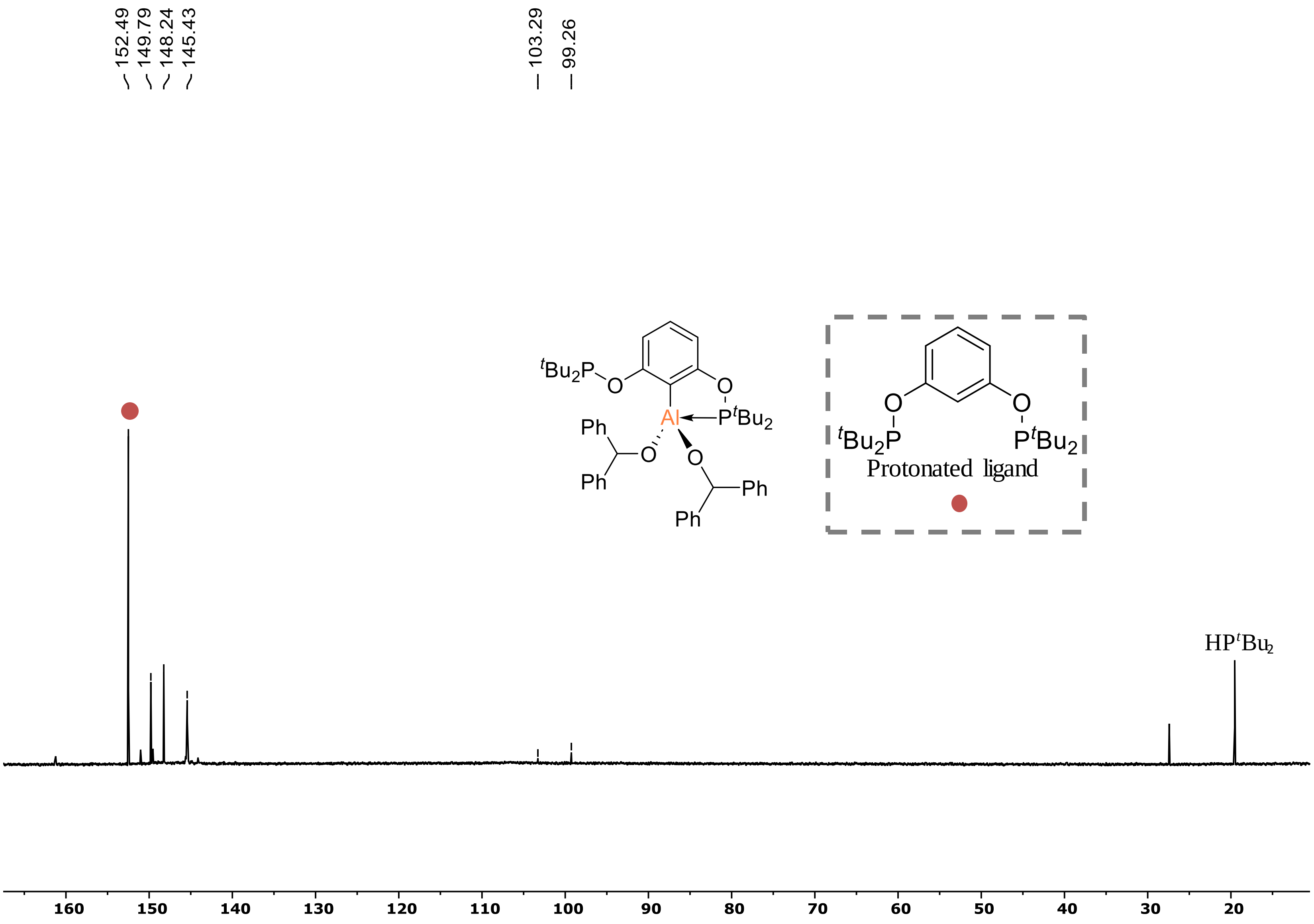

Figure S68: ^{1}H-^{31}P HMBC NMR spectrum of the reaction between **6** and 2 equivalents of benzophenone (C_6D_6, 298 K).

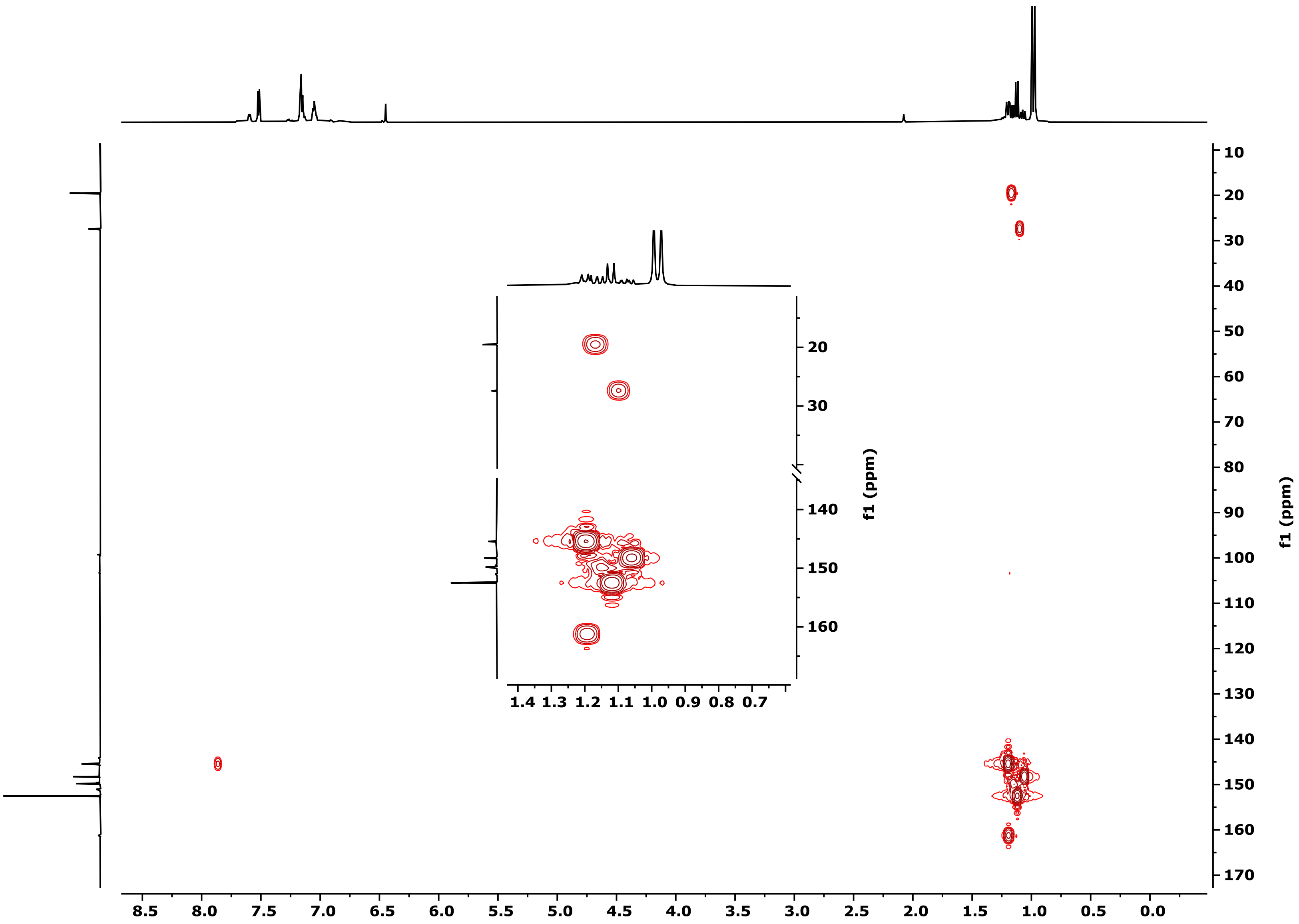

Figure S69: ^{1}H NMR spectrum of the reaction between **6** and CO_2 after 15 minutes (600 MHz, C_6D_6, 298 K).

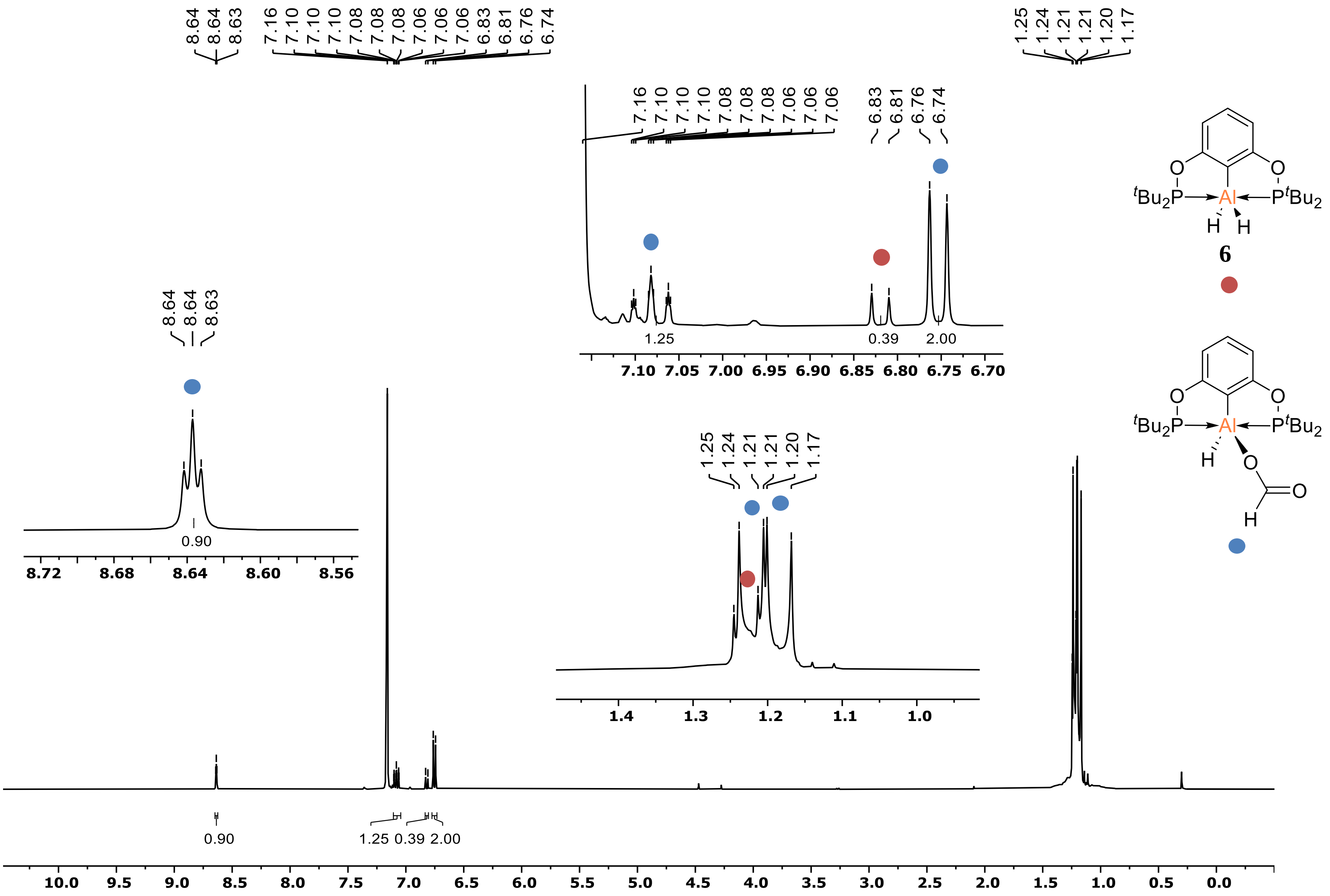

Figure S70: $^{13}C\{^{1}H\}$ NMR spectrum of the reaction between **6** and CO_2 after 15 minutes (151 MHz, C_6D_6, 298 K).

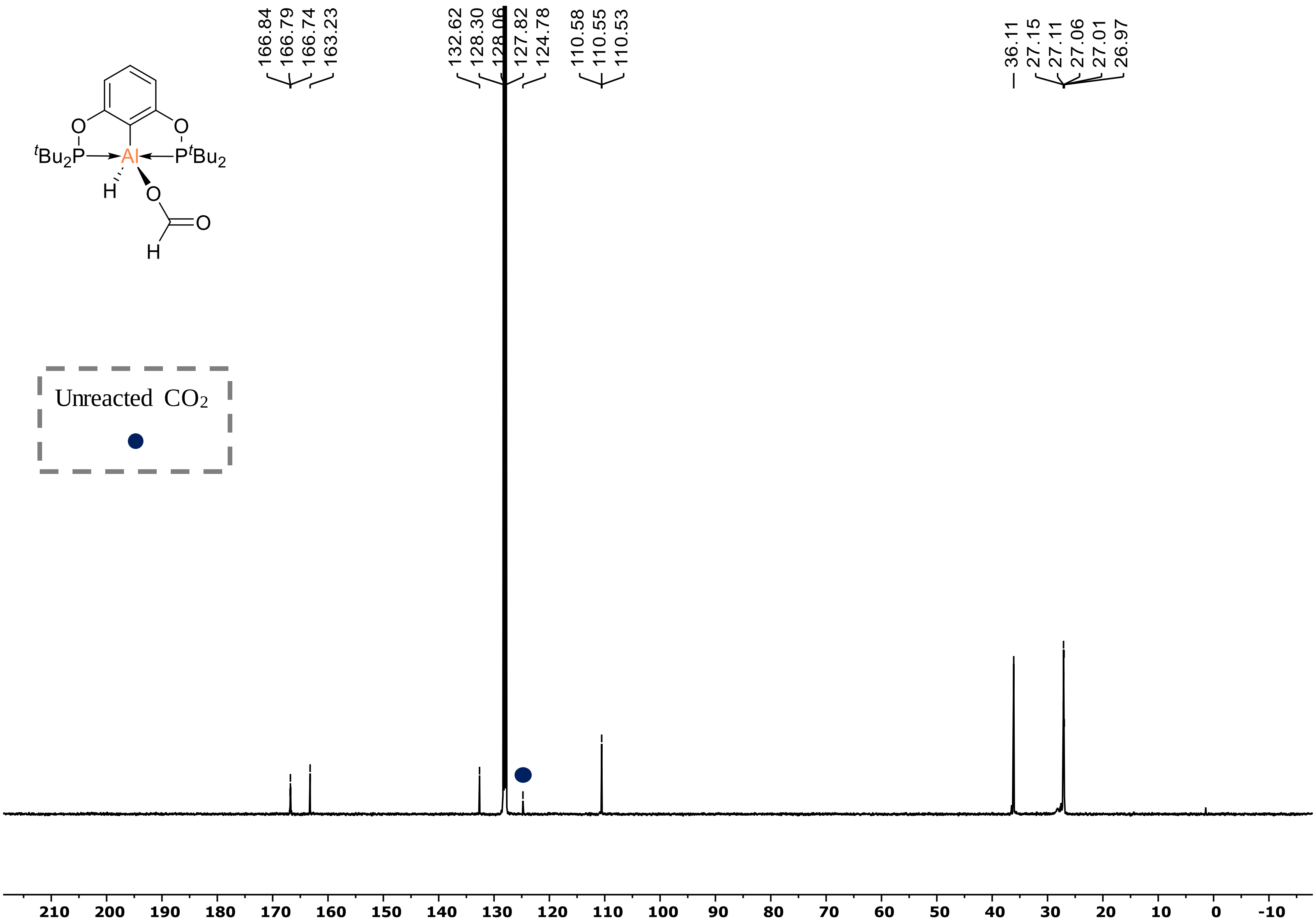

Figure S71: $^{31}P\{^{1}H\}$ NMR spectrum of the reaction between **6** and CO_2 after 15 minutes (243 MHz, C_6D_6, 298 K).

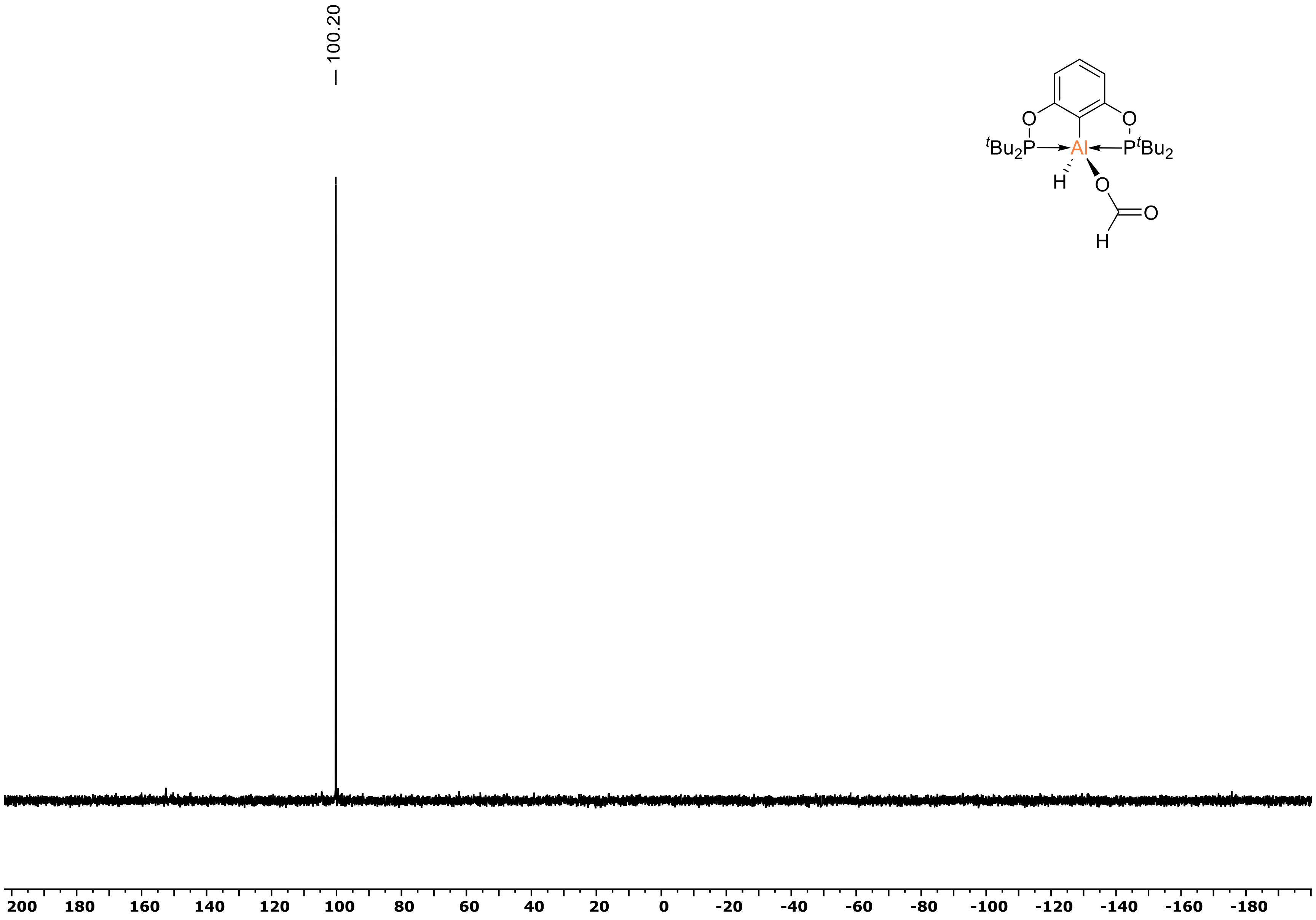

Figure S72: ^1H NMR spectrum of the reaction between 50 mg **6** and CO_2 after 24 hours (600 MHz, C_6D_6, 298 K).

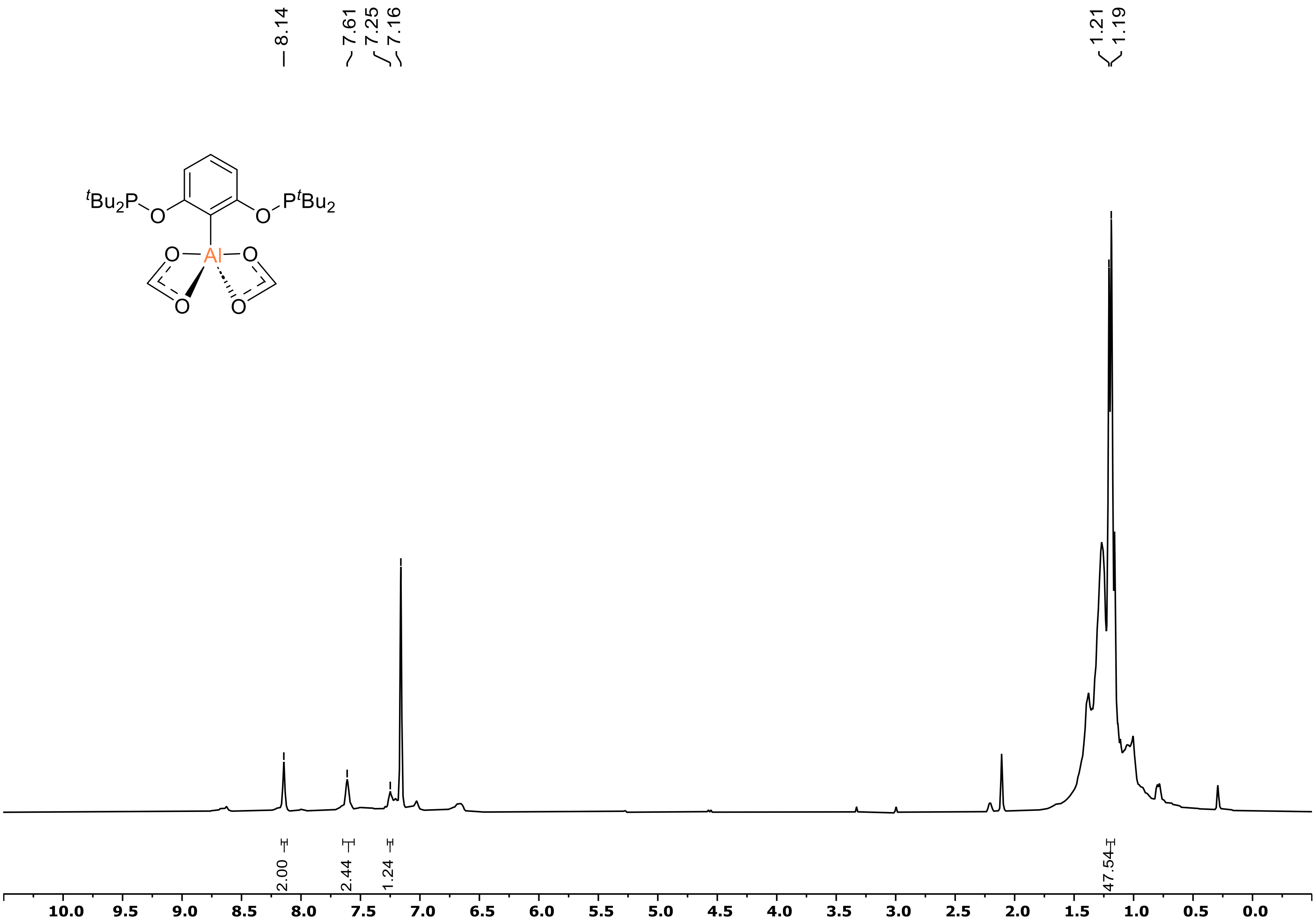

Figure S73: $^{13}C\{^{1}H\}$ NMR spectrum of the reaction between 50 mg **6** and CO_2 after 24 hours (151 MHz, C_6D_6, 298 K).

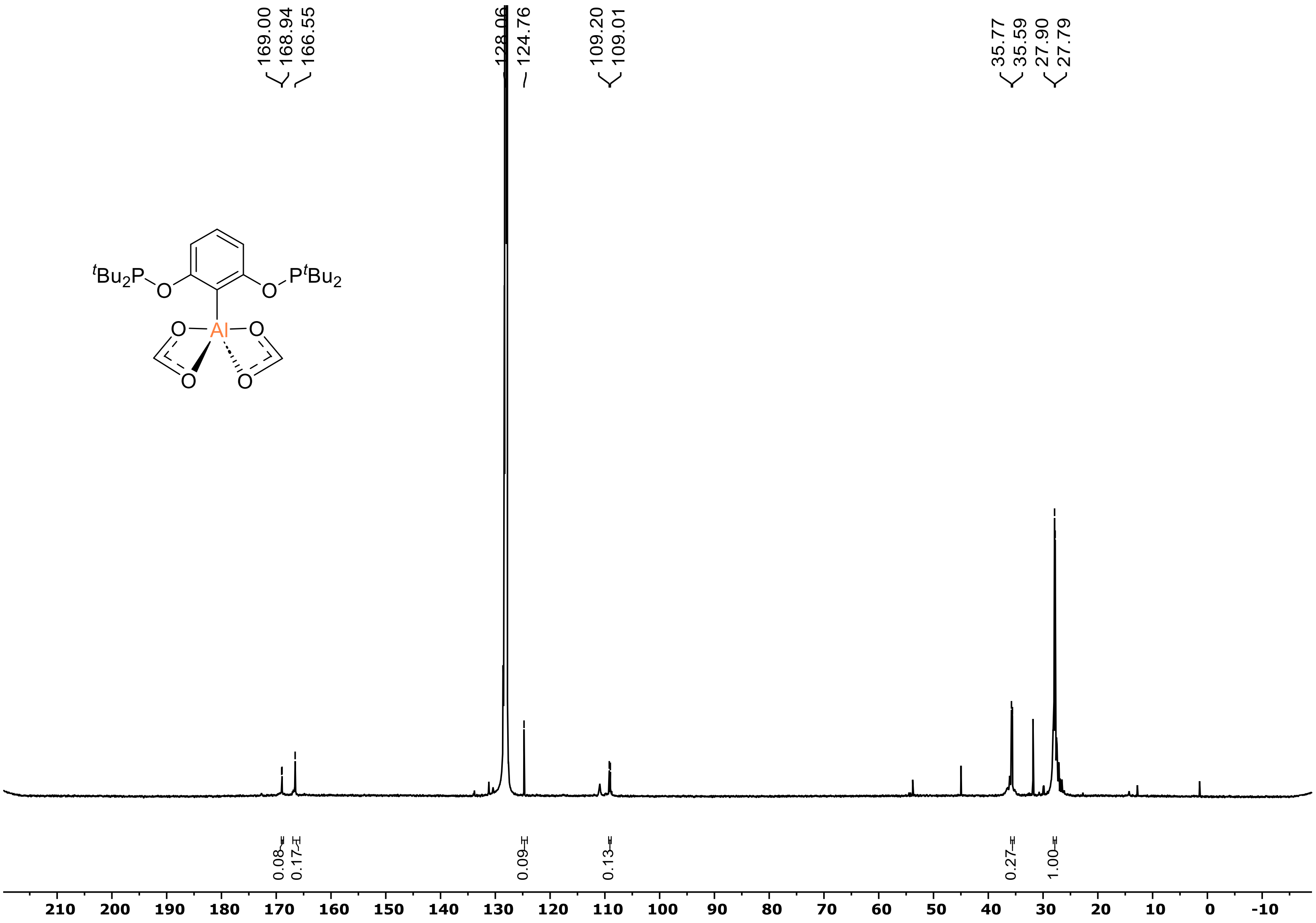

Figure S74: $^{31}P\{^{1}H\}$ NMR spectrum of the reaction between 50 mg **6** and CO_2 after 24 hours (243 MHz, C_6D_6, 298 K).

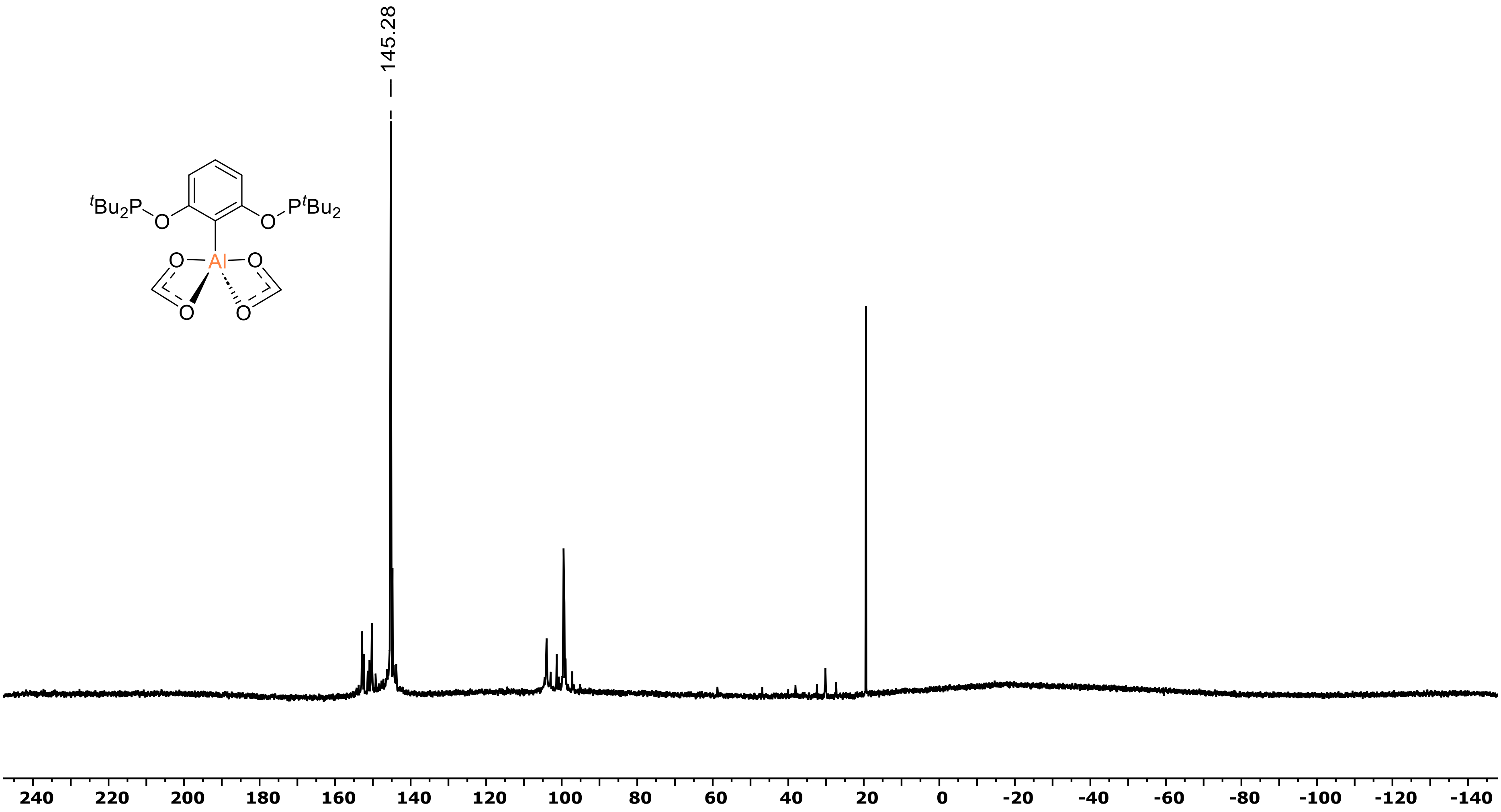

6. NMR spectra from *in situ* reactions of 6

6.1 Reactivity with benzophenone

Figure S75: ^{1}H NMR spectra for the reaction between **6** and benzophenone (1 and 3 equivalents, 400 MHz, C_6D_6, 298 K; 2 equivalents, 600 MHz, C_6D_6, 298 K).

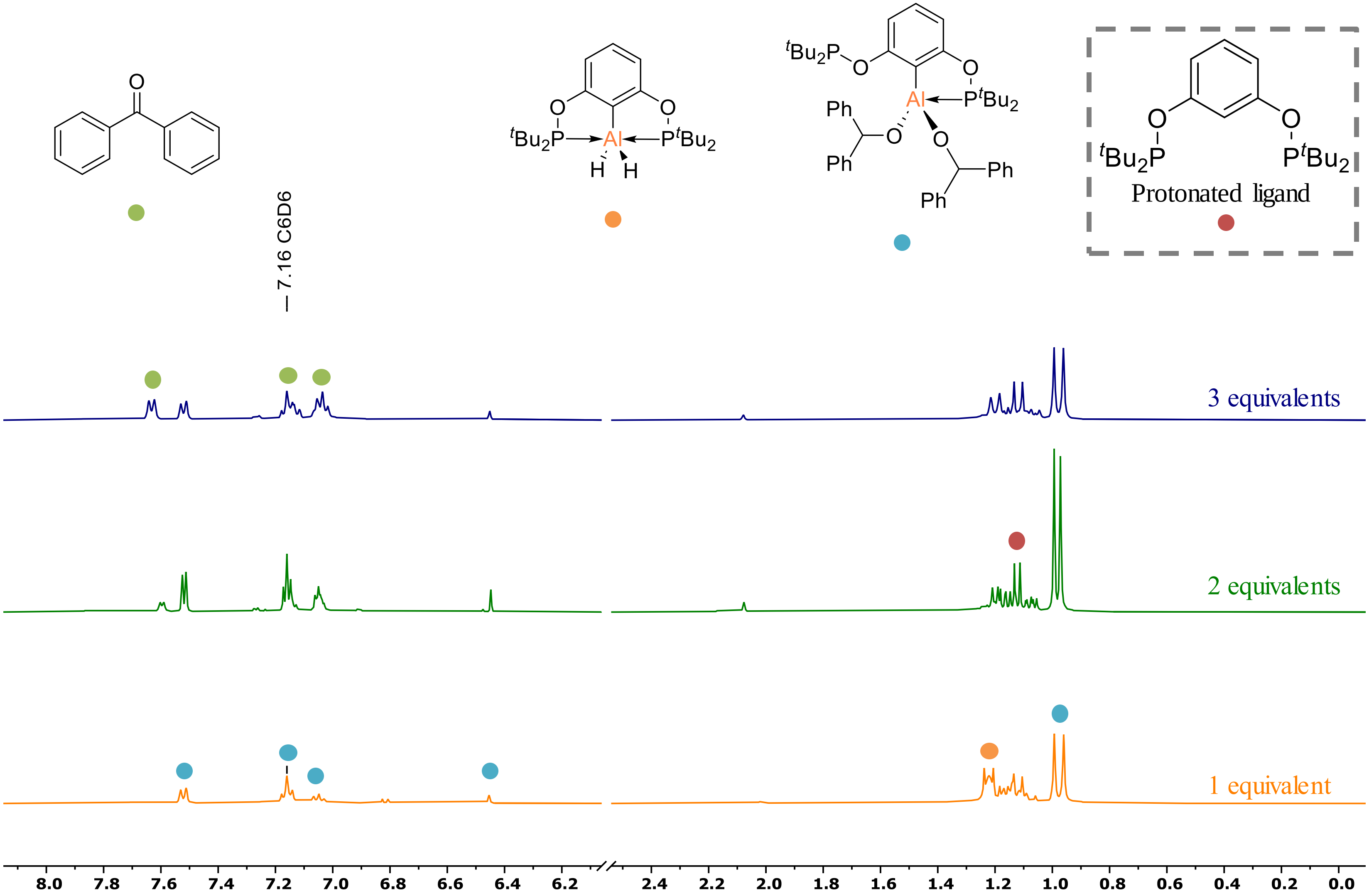

Figure S76: $^{31}P\{^{1}H\}$ NMR spectra of the reaction between **6** and benzophenone (1 and 3 equivalents, 162 MHz, C_6D_6, 298 K; 2 equivalents, 243 MHz, C_6D_6, 298 K). No definitive signals for the diinsertion product can be assigned.

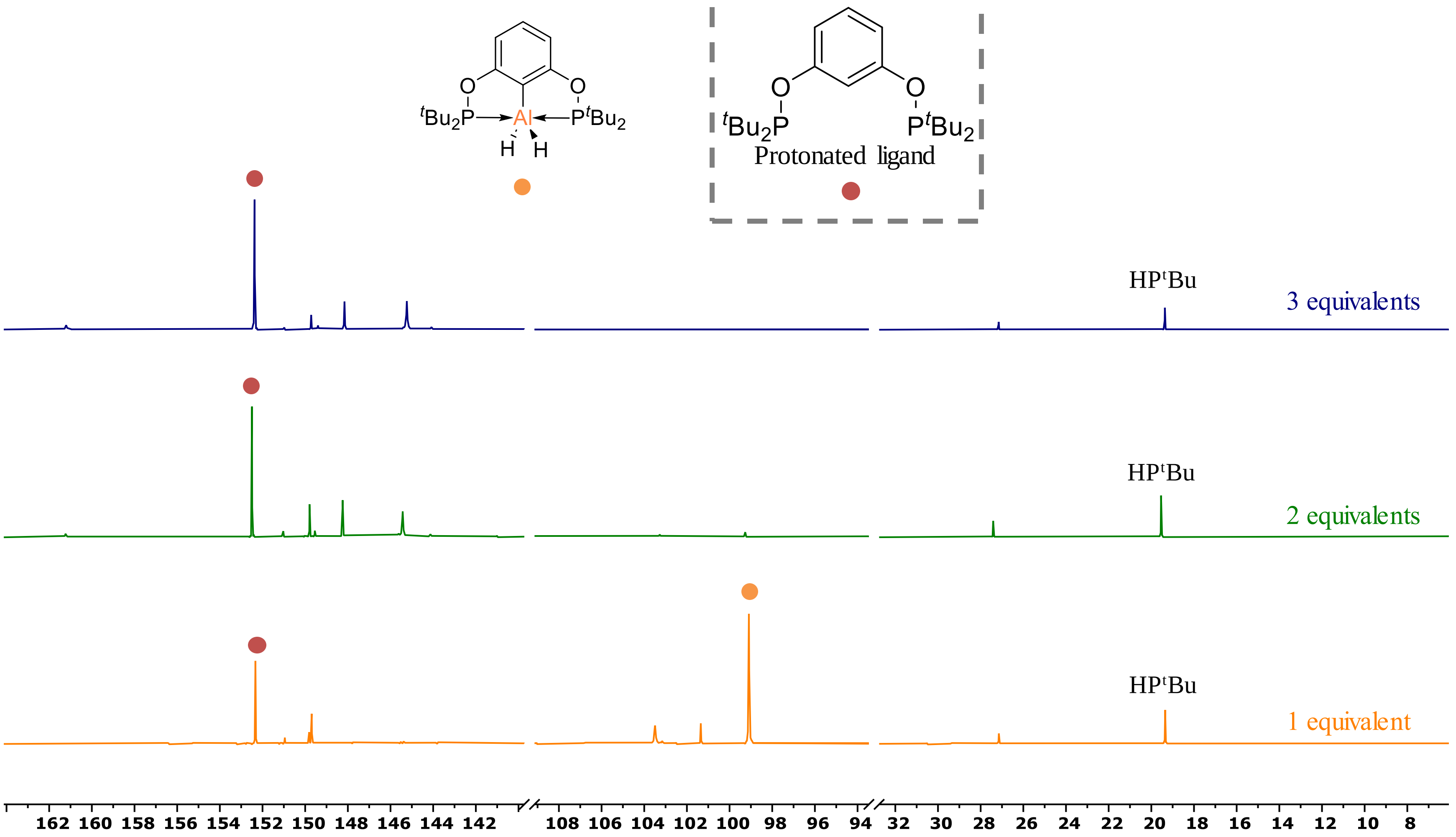

6.2 Reactivity with CO_2

Reactions were carried out to test different conditions which may affect how CO_2 inserts into compound **6**, such as purity of sample used, concentration, vessel size and evacuation method. In Figures S77 and S78, the conditions used are described in the table below.

Table S1 – Details on the material and methods used for *in situ* monitoring of CO_2 reactivity towards **6**.

#	Material used	Amount	Vessel	Evacuation method
1	[^{tBu}POCOP]AlH_2 powder	26 mg	NMR tube	Freeze-pump-thaw
2	[^{tBu}POCOP]AlH_2 powder	50 mg	10 mL flask	Freeze-pump-thaw
3	[^{tBu}POCOP]AlH_2 crystal, trace HP^tBu_2	50 mg	NMR tube	Headspace gas evacuated
4	[^{tBu}POCOP]AlH_2 powder	50 mg	NMR tube	Headspace gas evacuated
5	[^{tBu}POCOP]AlH_2 crystal	5 mg	NMR tube	Freeze-pump-thaw

From these experiments, it was concluded that reacting CO_2 with crystalline as opposed to powdered material resulted in less side products. This illustrates the sensitivity of compound **6**. Too much of an excess of CO_2 (by larger headspace) caused more peaks to arise in the $^{31}P\{^1H\}$ NMR spectra. There suggested to be no real effect depending on which evacuation method was used.

Figure S77: ^{1}H NMR spectra for the reaction between **6** and CO_2. Conditions used can be found in Table S1. (#1, #3 #5, [^{tBu}POCOP]AlH_2, 400 MHz, C_6D_6, 298 K; #2, #4, 600 MHz, C_6D_6, 298 K).

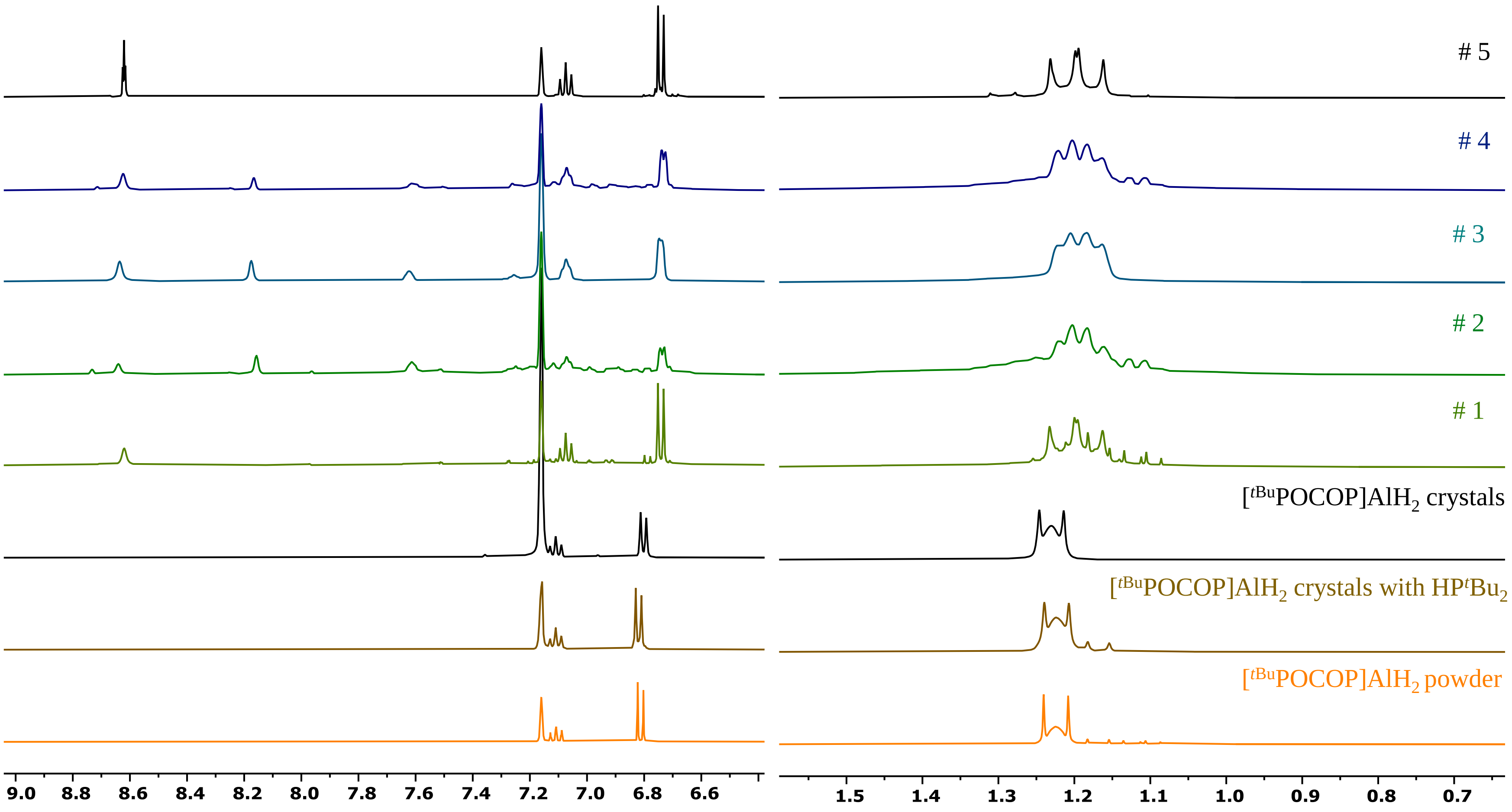

Figure S78: $^{31}P\{^{1}H\}$ NMR spectra for the reaction between **6** and CO_2 Conditions used can be found in Table S1. (#1, #3 #5, [^{tBu}POCOP]AlH_2, 400 MHz, C_6D_6, 298 K; #2, #4, 600 MHz, C_6D_6, 298 K).

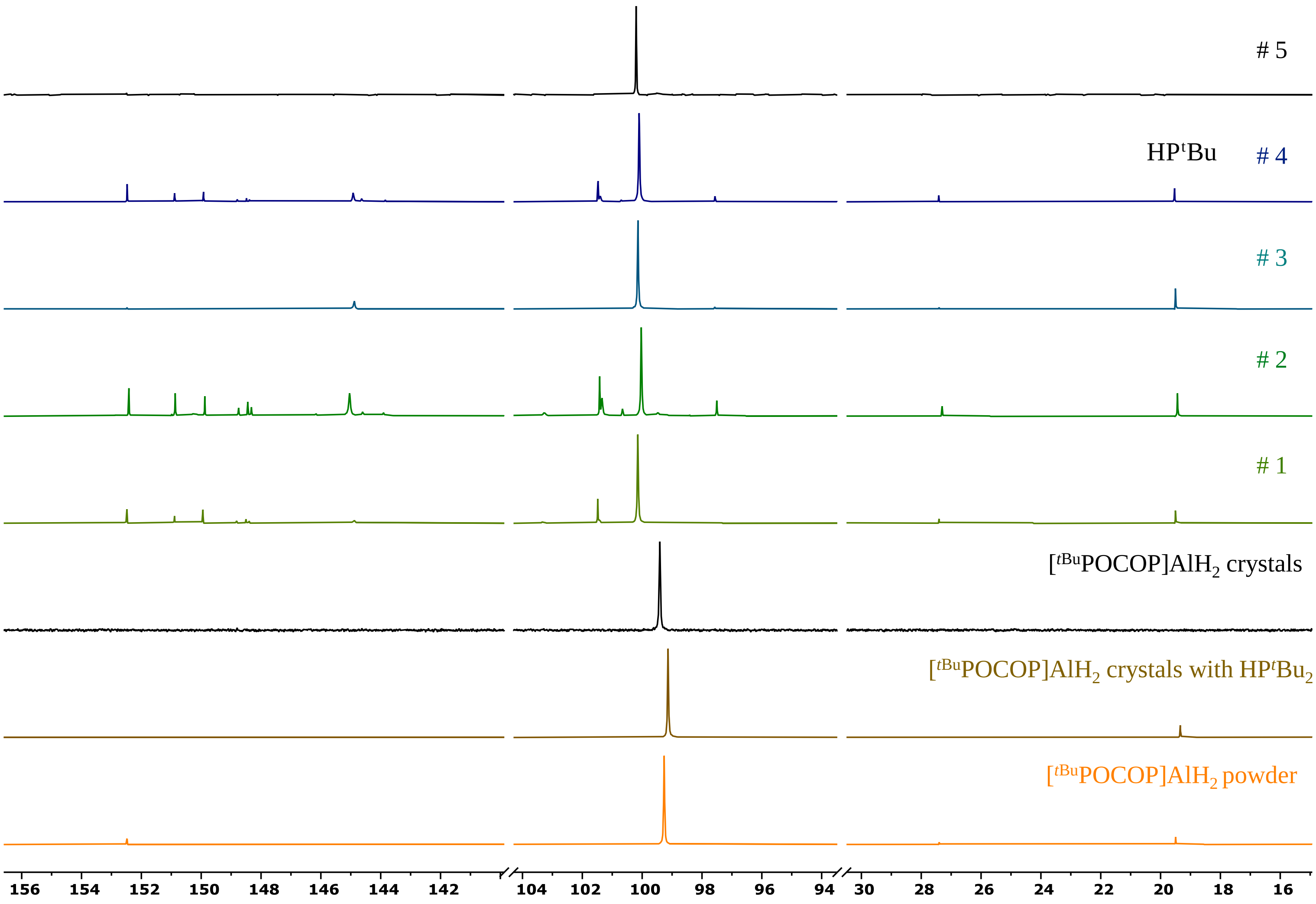

7. Crystallographic data for the structural analysis of compounds 1–8 and [^{tBu}POCOP]Al(OCHPh$_2$)$_2$

Single crystals of **1–7** were grown in an N_2 filled glovebox by slow evaporation from C_6D_6 at –30 °C. Crystals of **8** and **[^{tBu}POCOP]Al(OCHPh$_2$)$_2$** were isolated from a concentrated pentane solution at – 32 °C in a glovebox. For compound **7**, the analysis was carried out by the UK National Crystallography Service. Crystals were picked under polarized optical microscopy in Fomblin®Y and data for compounds **1**, **2**, **3/3'**, **3/3'_2**, **4**, **5, 6** and **8** were recorded on an Agilent SuperNova Dual Atlas four-circle diffractometer using either Mo or Cu Kα radiation and a CCD detector. The data collection temperatures were controlled using Oxford Cryostream systems. Data were collected, integrated and corrected for absorption usingCrysAlisPro.[2] The absorption correction implemented a numerical absorption correction based on Gaussian integration over a multifaceted crystal model. For **[^{tBu}POCOP]Al(OCHPh$_2$)$_2$** data was collected in Experimental Hutch 1 (EH1) on beamline I19 at Diamond Light Source[3] on a Fluid Film Devices 3-circle fixed-chi diffractometer (Dectris PILATUS 2M Detector, wavelength 0.6889 Å). The frames were integrated using XIA26 software[4] and the data were corrected for absorption effects using a AIMLESS[5] an empirical method. Structures were solved within Olex2[6] by dual space iterative methods (SHELXT)[7] and all non-hydrogen atoms refined by full-matrix least-squares on all unique F2 values with anisotropic displacement parameters (SHELXL).[8] All hydrogen atoms, except Al hydrides in complexes **6** and **7**, were located in the electron density map and refined with constrained geometries and riding thermal parameters. For compounds **6** and **7**, details on the refinement of these atoms can be found in the corresponding CIF. The structures have been deposited with the Cambridge Structural Database [CSD accession numbers: 2420397–2420401, 2490290, 2493731,

2494232, 2529859, 2529995]. These can be obtained free of charge from the Cambridge Crystallographic Data Centre via www.ccdc.cam.ac.uk/data_request/cif.

Identification code	1	2	3/3'	3/3'_2	4
CSD No	2493731	2420400	2420401	2490290	2494232
Empirical formula	$C_{22}H_{39}O_2P_2Br$	$C_{22}H_{39}AlO_2P_2Cl_2$	$C_{22}H_{39}GaO_2P_2Cl_2$	$C_{22}H_{39}GaO_2P_2Cl_2$	$C_{22}H_{39}InO_2P_2Cl_2$
Formula weight	477.40	495.38	538.09	538.09	583.22
Temperature/K	200(2)	200(2)	200(2)	200(2)	200(2)
Crystal system	ortho-rhombic	monoclinic	triclinic	monoclinic	monoclinic
Space group	*Pbca*	$P2_1/c$	*P*-1	$P2_1/c$	$P2_1/c$
Habit	Shards	Hexagonal plates	Blocks	Blocks	Blocks
Crystal color	Colorless	Colorless	Colorless	Colorless	Colorless
a/Å	16.0850(5)	15.7276(2)	8.7802(3)	15.7802(6)	15.9665(5)
b/Å	15.9403(5)	10.8387(2)	17.9425(7)	10.8337(4)	10.8325(3)
c/Å	20.1643(5)	15.8735(3)	19.4941(8)	15.7910(6)	15.8642(5)
α/°	90	90	63.551(4)	90	90
β/°	90	98.909(2)	86.087(3)	98.922(4)	99.007(3)
γ/°	90	90	89.556(3)	90	90
Volume/Å^3	5170.1(3)	2673.26(8)	2742.3(2)	2666.93(18)	2709.99(14)
Z	8	4	4	4	4
ρ_{calc}g/cm^3	1.227	1.231	1.303	1.340	1.429
μ/mm^{-1}	1.727	3.751	4.382	1.368	1.203
F(000)	2016	1056	1128	1128	1200
Crystal size/mm^3	0.5 × 0.39 × 0.27	0.30 × 0.15 × 0.15	0.34 × 0.13 × 0.10	0.3 × 0.27 × 0.17	0.48 × 0.46 × 0.33
Diffracto-meter	SuperNova, Dual, Cu at home/near, Atlas	SuperNova, Dual, Cu at home/near, Atlas	SuperNova, Dual, Cu at home/near, Atlas	SuperNova, Dual, Cu at home/near, Atlas	SuperNova, Dual, Cu at home/near, Atlas
Absorption correction	Gaussian	Gaussian	Gaussian	Gaussian	Gaussian
No. of measured independent reflections	6678	5276	10626	6390	6414

**Reflections collected**	44206	19095	21395	13014	14233
$\boldsymbol{R_{int}}$	0.0874	0.0277	0.0302	0.0185	0.0209
**No. of parameters**	256	302	807	414	293
**Goodness-of-fit on F^2**	1.025	1.034	1.043	1.035	1.034
**Final R indexes [I>=2σ (I)]**	R_1 = 0.0515, wR_2 = 0.0976	R_1 = 0.0383, wR_2 = 0.0961	R_1 = 0.0514, wR_2 = 0.1440	R_1 = 0.0372, wR_2 = 0.0872	R_1 = 0.0257, wR_2 = 0.0617
**Final R indexes [all data]**	R_1 = 0.1009, wR_2 = 0.1138	R_1 = 0.0463, wR_2 = 0.1032	R_1 = 0.0605, wR_2 = 0.1533	R_1 = 0.0507, wR_2 = 0.0950	R_1 = 0.0324, wR_2 = 0.0660
**Largest diff. peak/hole / e Å^{-3}**	0.60/–0.66	0.64/–0.44	1.39/–0.85	0.96/–0.58	0.43/–0.96

**Identification code**	**5**	**6**	**7**	**8**	**[^{tBu}POCOP]Al(OCHPh$_2$)$_2$**
**CSD No**	2420398	2420399	2420397	2529859	2529995
**Empirical formula**	$C_{24}H_{45}AlO_2P_2$	$C_{22}H_{41}AlO_2P_2$	$C_{22}H_{40}AlO_2P_2Cl$	$C_{48}H_{85}AlN_4O_2P_2$	$C_{48}H_{61}AlO_4P_2$
**Formula weight**	454.52	426.47	461.69	839.11	790.88
**Temperature/K**	293(2)	200(10)	100.15	130.00(10)	100.00
**Crystal system**	monoclinic	monoclinic	triclinic	Monoclinic	monoclinic
**Space group**	$P2_1/c$	$P2_1/c$	P-1	$P2_1/c$	$P2_1/c$
**Habit**	Blocks	Hexagonal plates	Shards	Blocks	Irregular
**Crystal color**	Colorless	Colorless	Colorless	Colorless	Colorless
**a/Å**	15.9236(6)	16.0946(5)	8.28860(10)	15.3035(2)	18.9527(3)
**b/Å**	10.8943(5)	11.2703(2)	12.4566(2)	11.3566(2)	11.9121(2)
**c/Å**	15.8382(5)	15.5988(4)	13.2146(2)	29.6420(40	20.8873(3)
**α/°**	90	90	99.567(2)	90	90
**β/°**	99.354(4)	114.522(3)	96.5510(10)	102.6510(10)	107.4210(10)
**γ/°**	90	90	102.2980(10)	90	90
**Volume/Å^3**	2711.02(18)	2574.27(13)	1298.61(3)	5026.58(13)	4499.35(12)
**Z**	4	4	2	4	4
**ρ_{calc}g/cm^3**	1.114	1.100	1.136	1.109	1.168
**μ/mm^{-1}**	1.885	1.957	2.901	1.245	0.146
**F(000)**	992.0	928.0	484.1	1840.0	1696.0
**Crystal size/mm^3**	0.52 × 0.27 × 0.12	0.33 × 0.20 × 0.17	0.16 × 0.12 × 0.06	0.75 × 0.55× 0.44	0.12 × 0.055 × 0.04

Diffracto-meter	SuperNova, Dual, Cu at home/near, Atlas	SuperNova, Dual, Cu at home/near, Atlas	ROD, Synergy Custom system, HyPix-Arc 100	SuperNova, Dual, Cu at home/near, Atlas	Diamond Light Source, I19, EH1. Fluid Film Devices 3-circle fixed-chi diffractometer
**Absorption correction**	Gaussian	Gaussian	Multi-scan	Gaussian	Empirical
**No. of measured independent reflections**	5273	4988	4851	10467	13723
**Reflections collected**	11218	9913	24015	27195	76976
**R_{int}**	0.0307	0.0318	0.0239	0.0270	0.0610
**No. of parameters**	276	264	452	686	508
**Goodness-of-fit on F^2**	1.032	1.030	1.120	1.030	1.013
**Final R indexes [I>=2σ (I)]**	R_1 = 0.0488, wR_2 = 0.1305	R_1 = 0.0411, wR_2 = 0.1033	R_1 = 0.0341, wR_2 = 0.0814	R_1 = 0.0460, wR_2 = 0.1175	R_1 = 0.0464, wR_2 = 0.1155
**Final R indexes [all data]**	R_1 = 0.0606, wR_2 = 0.1436	R_1 = 0.0563, wR_2 = 0.1125	R_1 = 0.0354, wR_2 = 0.0820	R_1 = 0.0511, wR_2 = 0.1225	R_1 = 0.0760, wR_2 = 0.1225
**Largest diff. peak/hole / e Å^{-3}**	0.68/–0.41	0.42/–0.29	0.47/–0.35	0.64/-0.44	0.78/-0.35

Table S2:Crystallographic data for all X-ray structures presented in this manuscript.

Complex	*d*(P–E) /Å	*d*(C–E) /Å	∠(P–E–C) /°	∠(P–E–P) /°	*τ*
**2**	2.595(1)	2.005(2)	78.73(6)	154.93(5)	0.37
	2.660(1)		76.93(6)		
**3** "closed"	2.587(5)	1.997(7)	77.1(2)	154.6(2)	0.26
	2.702(6)		79.8(2)		
**3'** "open"	2.441(1)	1.959(3)	84.2(1)	–	–
**4**	2.701(1)	2.035(2)	77.05(7)	152.65(4)	0.38
	2.762(1)		75.93(6)		
**5**	2.5976(7)	2.024(2)	77.38(6)	154.85(3)	0.51
	2.6206(7)		78.00(6)		
**6**	2.572(1)	2.029(2)	77.25(5)	153.60(5)	0.28
	2.570(1)		77.74(5)		

Table S3: Structural parameters for complexes **2**–**7**. E = Al, Ga or In.

Figure S79: Solid state structure of **1**. Anisotropic displacement parameters are depicted at 50% probability level. Br is shown in brown, C is shown in grey, O is shown in red, and P is shown in orange. All H atoms are omitted and ^{t}Bu groups shown as wireframes for clarity.

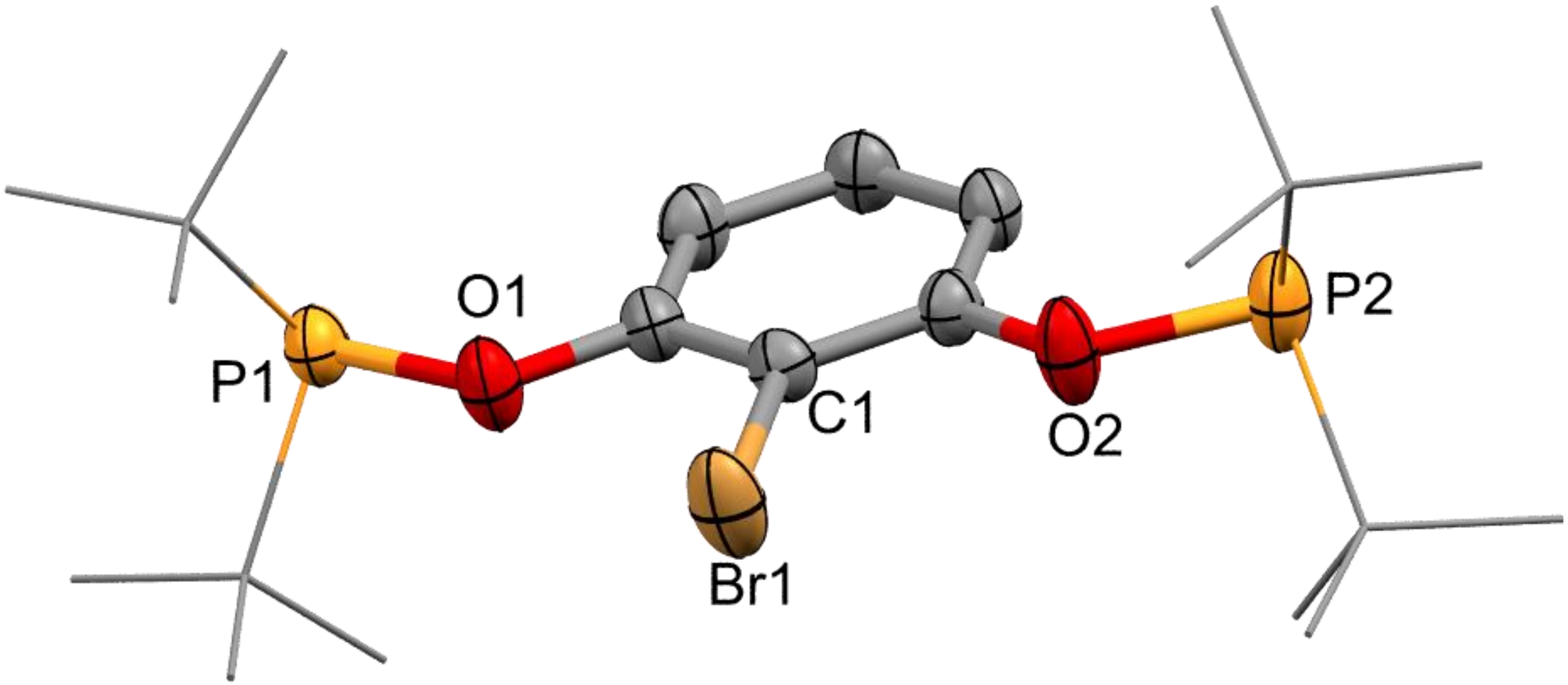

Figure S80: Solid state structure of **2**. Anisotropic displacement parameters are depicted at 50% probability level. Al is shown in pink, C is shown in grey, Cl is shown in green, O is shown in red, and P is shown in orange. All H atoms and disordered component are omitted and ^{t}Bu groups shown as wireframes for clarity.

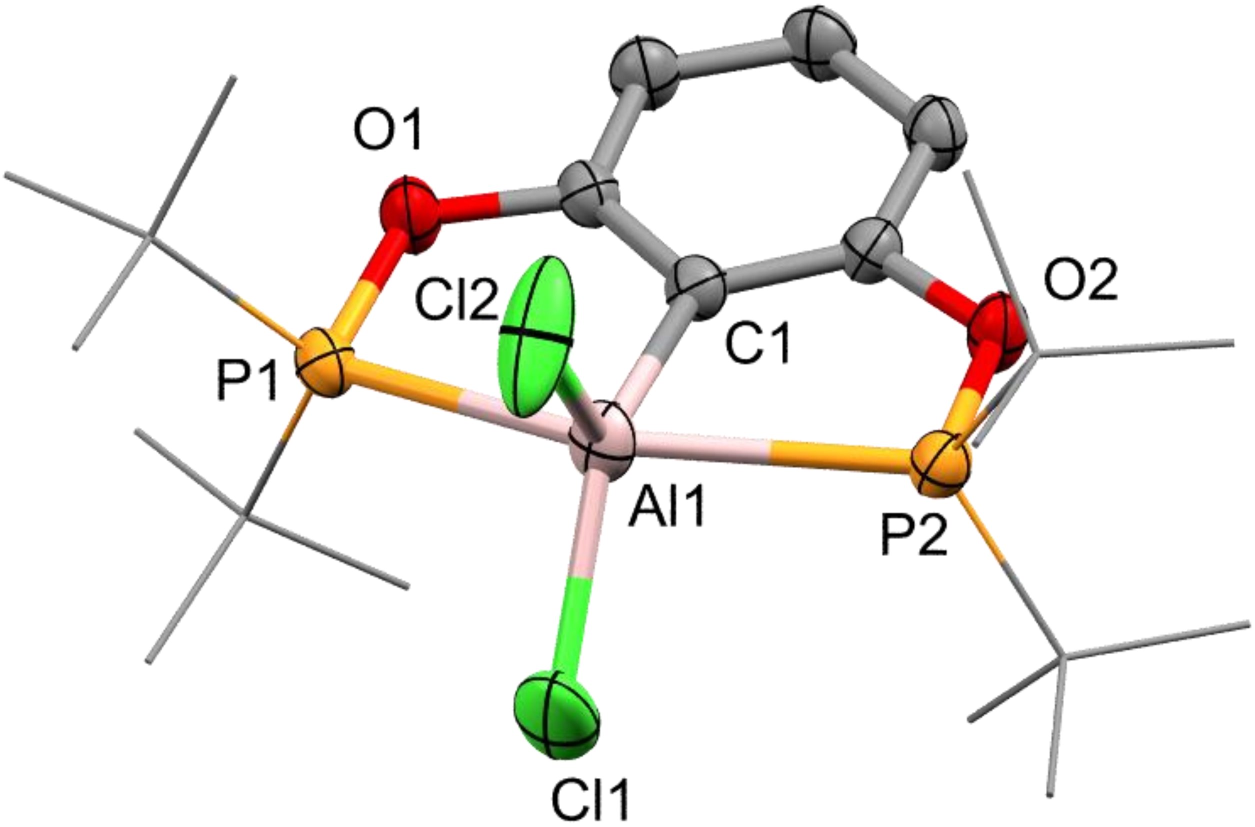

Figure S81: Solid state structure of **3** and **3'** in $P\bar{1}$. Both components from the asymmetric unit are depicted below. Anisotropic displacement parameters are depicted at 50% probability level. C is shown in grey, Cl is shown in green, Ga is shown in pink, O is shown in red, and P is shown in orange. All H atoms and disordered components are omitted and ^{t}Bu groups shown as wireframes for clarity.

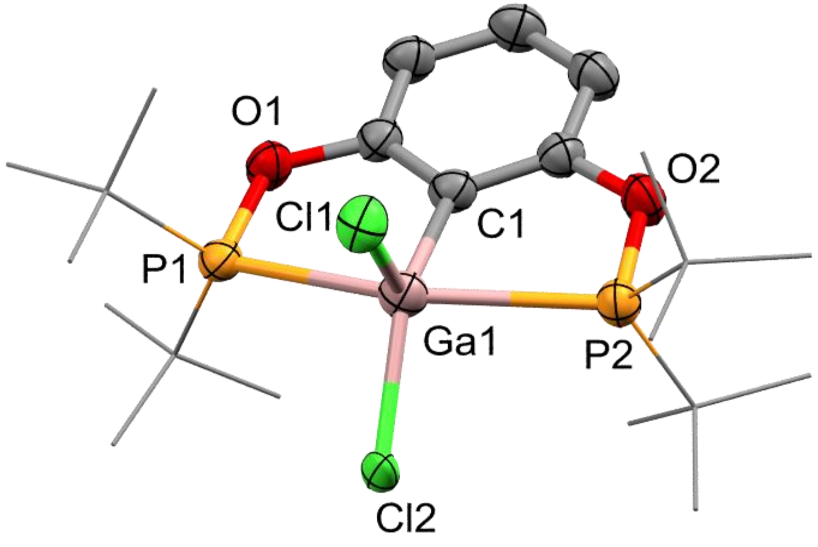

3

~50% occupancy

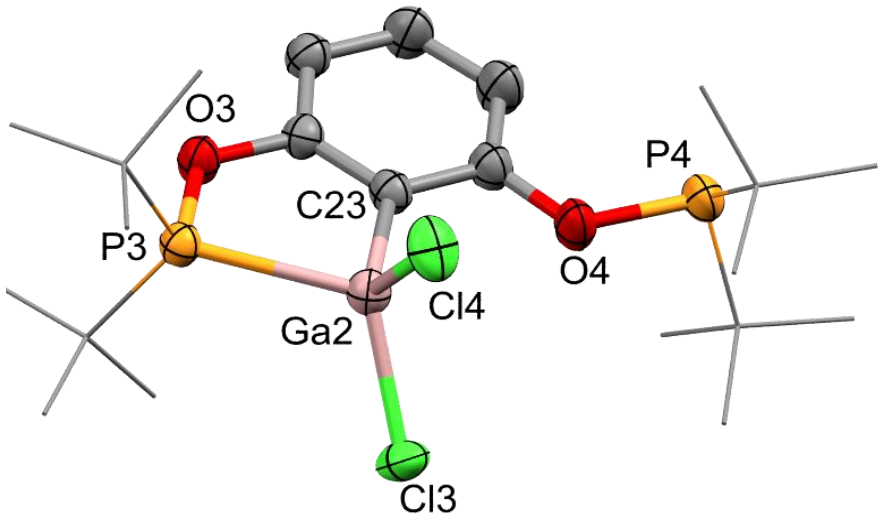

3'

~50% occupancy

Figure S82: Solid state structure of **3** and **3'** in $P2_1/c$ (**3/3'_2**). **3** and **3'** crystallized on the same site within the unit cell in a ratio of 0.95:0.05, respectively. Independent components as well as the full model shown below. Anisotropic displacement parameters are depicted at 50% probability level. C is shown in grey, Cl is shown in green, Ga is shown in pink, O is shown in red, and P is shown in orange. All H atoms and disordered components are omitted and ^{t}Bu groups shown as wireframes for clarity.

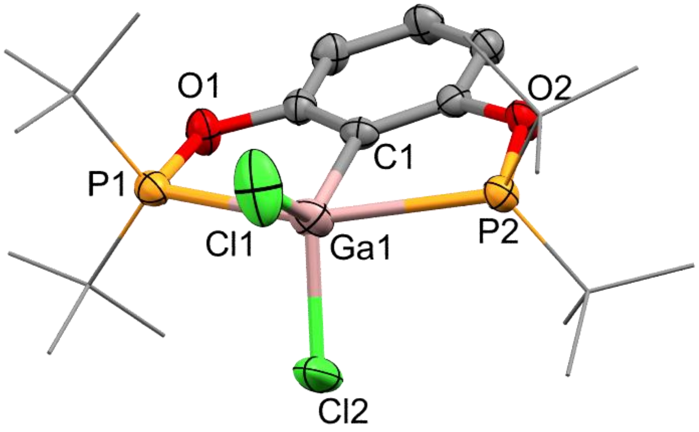

3

~95% occupancy

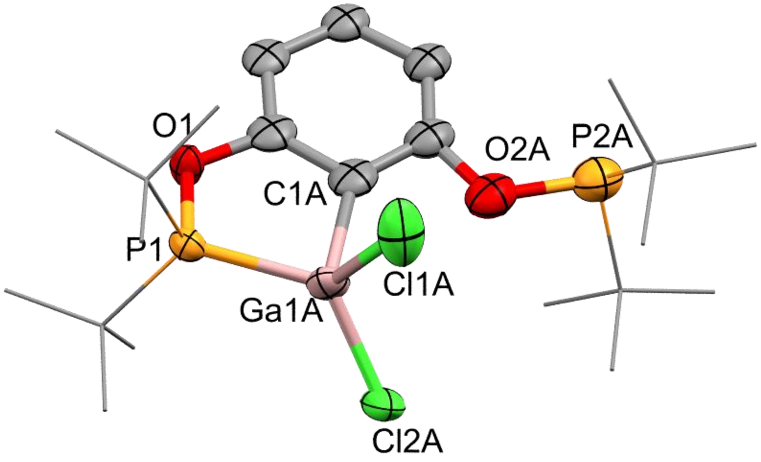

3'

~5% occupancy

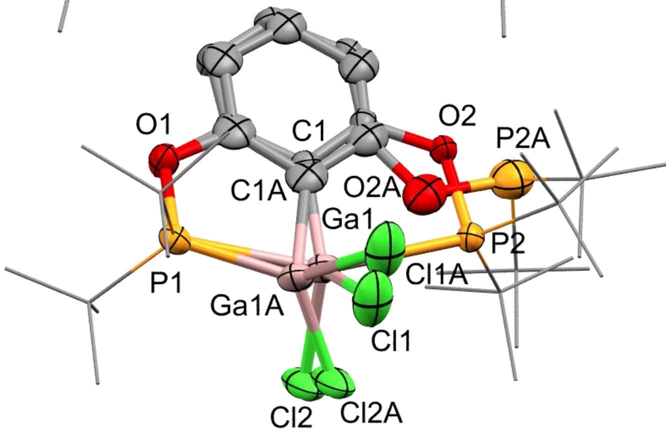

3/3'

Full crystal structure

Figure S83: Solid state structure of **4**. Anisotropic displacement parameters are depicted at 50% probability level. C is shown in grey, Cl is shown in green, In is shown in pink, O is shown in red, and P is shown in orange. All H atoms and disordered components are omitted and ^{t}Bu groups shown as wireframes for clarity.

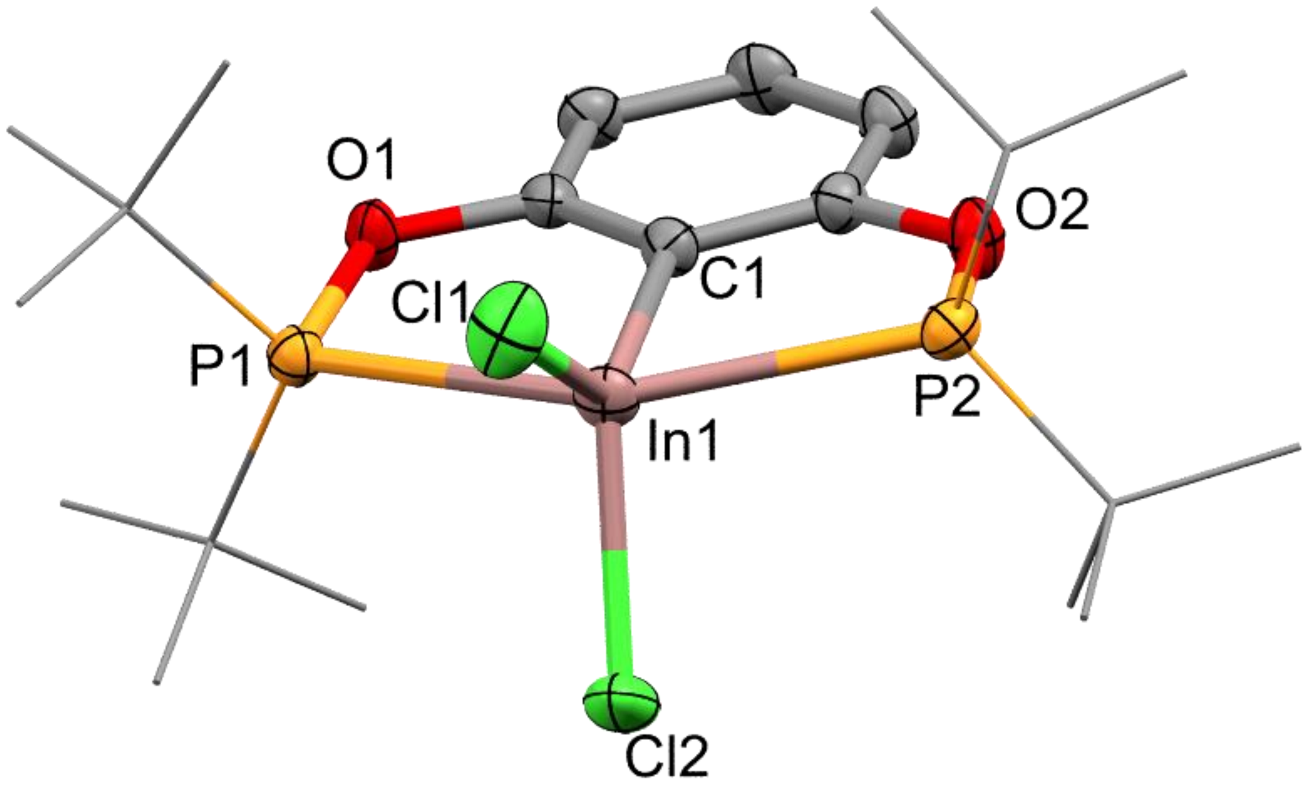

Figure S84: Solid state structure of **5**. Anisotropic displacement parameters are depicted at 50% probability level. Al is shown in pink, C is shown in grey, O is shown in red, and P is shown in orange. All H atoms are omitted and ^{t}Bu groups shown as wireframes for clarity.

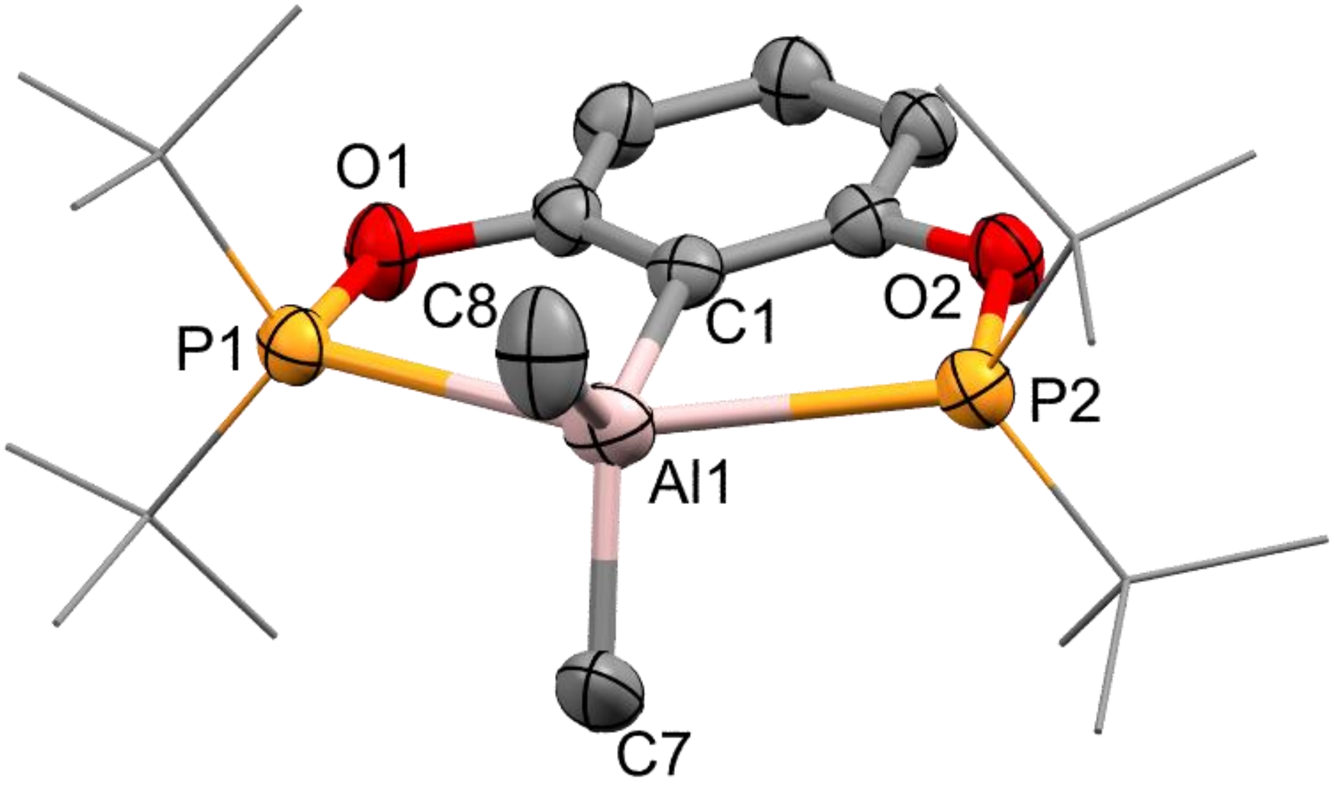

Figure S85: Solid state structure of **6**. Anisotropic displacement parameters are depicted at 50% probability level. Al is shown in pink, C is shown in grey, O is shown in red, and P is shown in orange. All H atoms, except H1 and H2, are omitted and ^{t}Bu groups shown as wireframes for clarity.

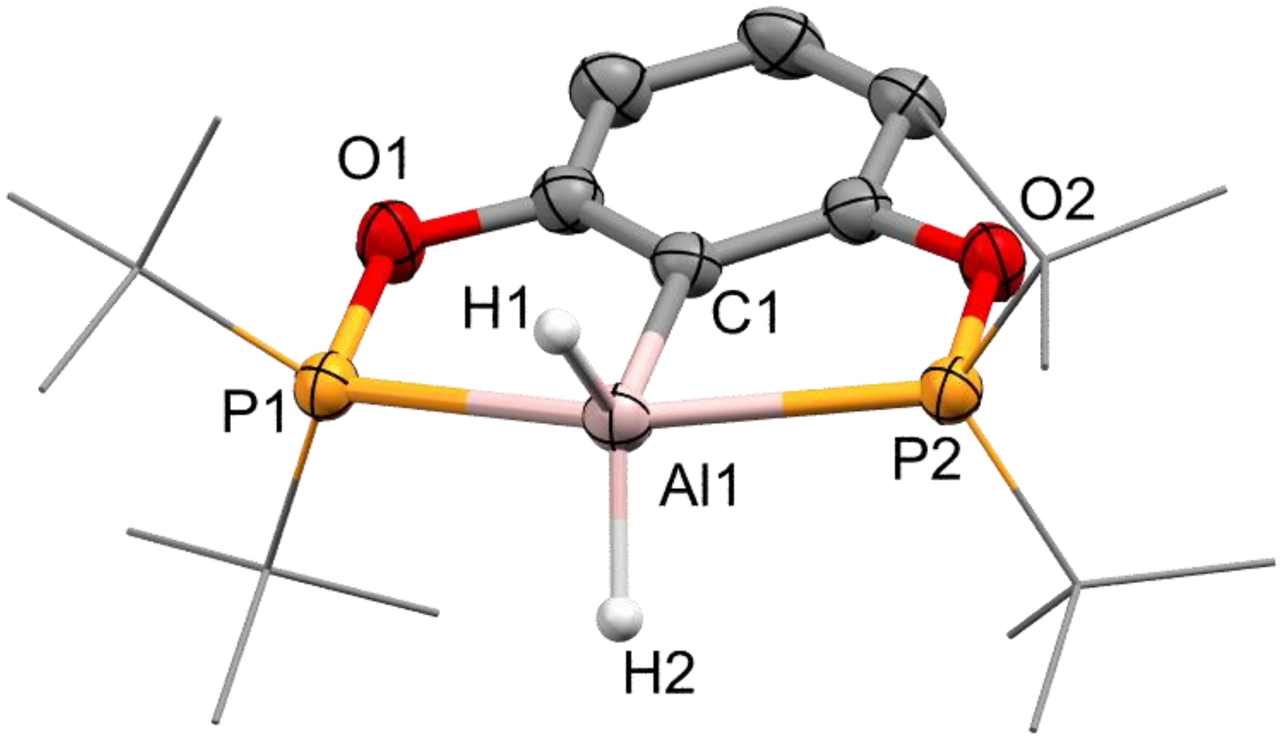

Figure S86: Solid state structure of **7**. Anisotropic displacement parameters are depicted at 50% probability level. Al is shown in pink, C is shown in grey, Cl is shown in green, O is shown in red, and P is shown in orange. All H atoms, except H1, and disordered component are omitted and ^{t}Bu groups shown as wireframes for clarity.

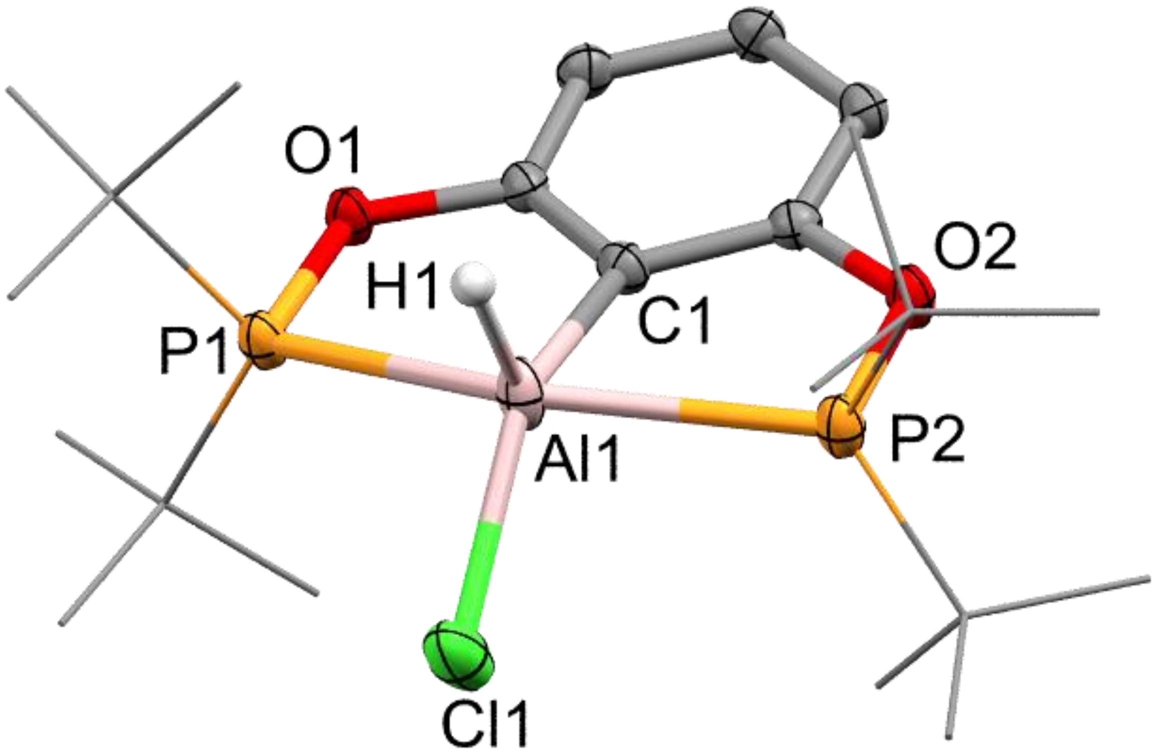

Figure S87: Solid state structure of **8**. Anisotropic displacement parameters are depicted at 50% probability level. Al is shown in pink, C is shown in grey, N is shown in blue, O is shown in red, and P is shown in orange. All H atoms, except H23 and H36, and disordered component are omitted and ^{t}Bu/Cy groups shown as wireframes for clarity.

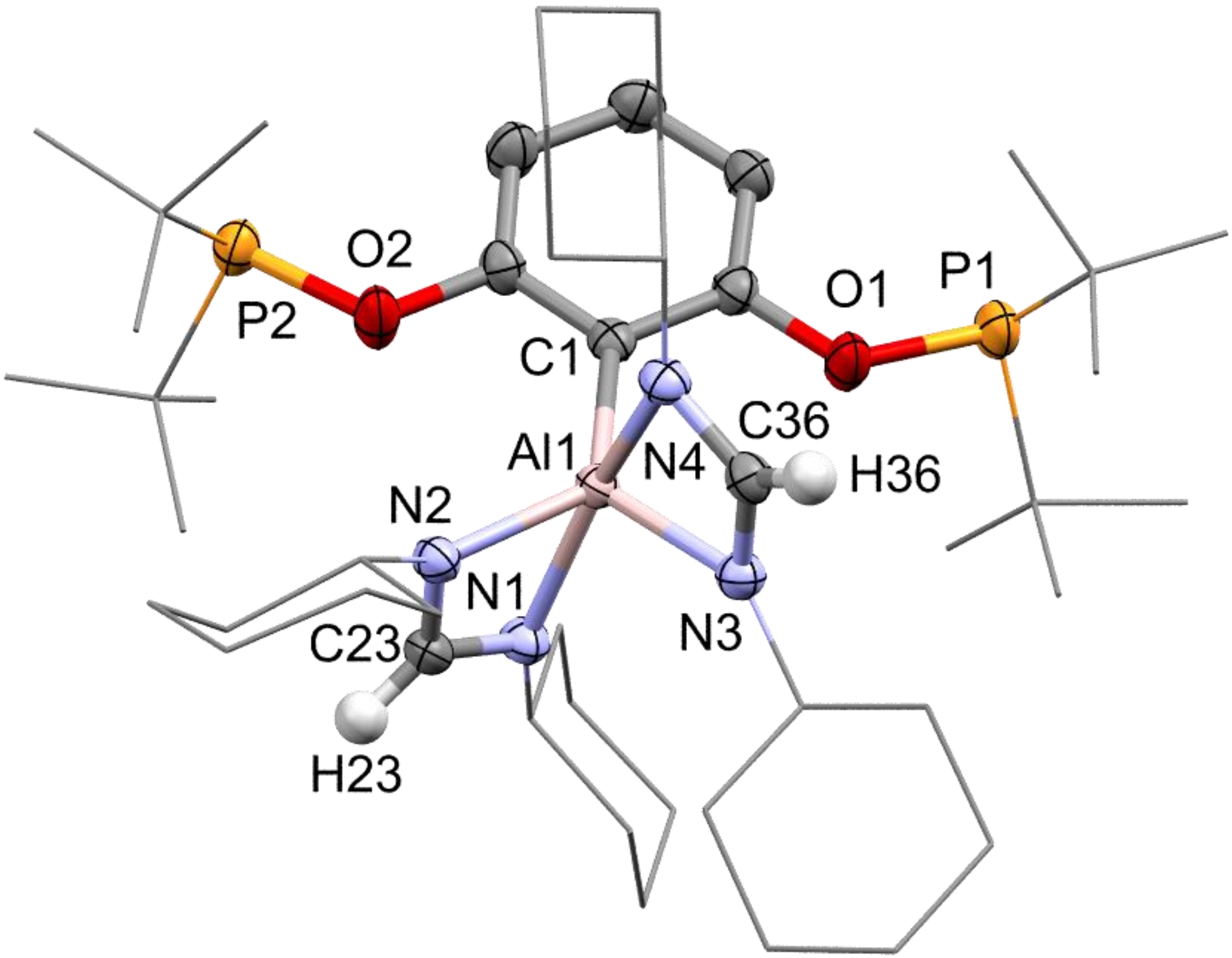

Figure S88: Solid state structure from the reaction of **6** with two equivalents of benzophenone. Anisotropic displacement parameters are depicted at 50% probability level. Al is shown in pink, C is shown in grey, O is shown in red, and P is shown in orange. All H atoms, except H23 and H36, are omitted for clarity.

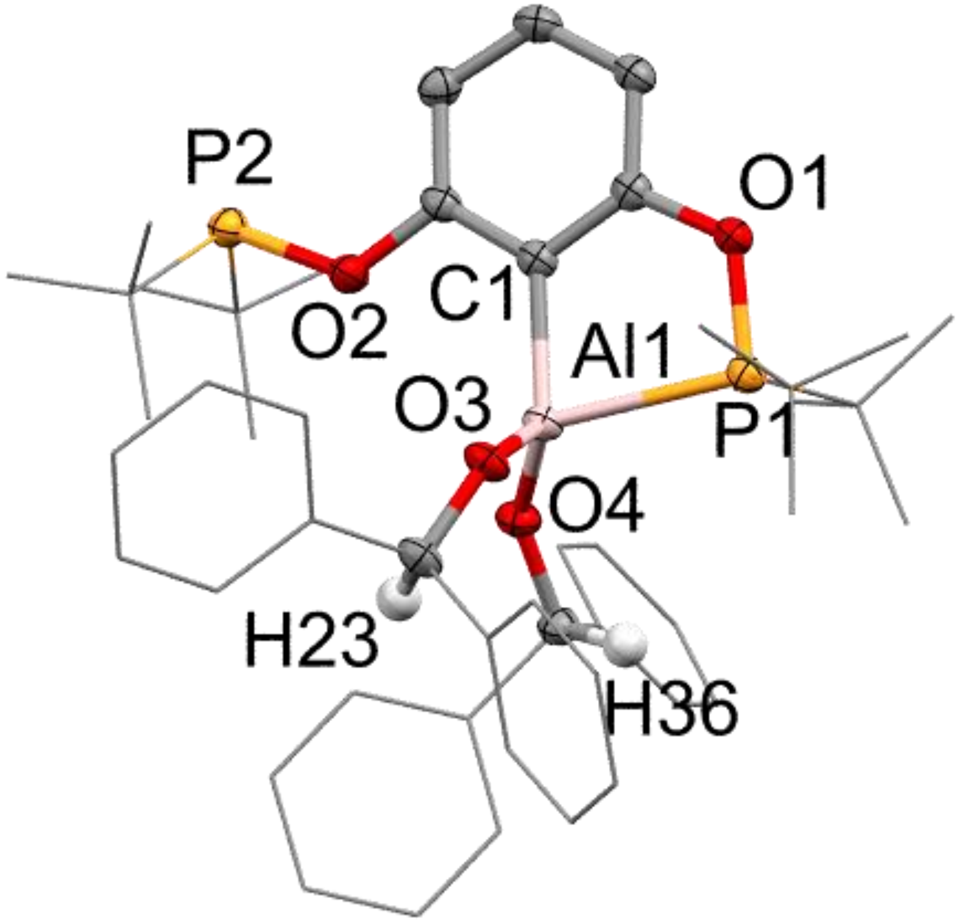

8. Computational methodology

The closed and open structures of the Ga, Al and In POCOP catalysts were taken from the crystal structures obtained from X-ray diffraction. The structure geometries were optimized using density functional theory (DFT), and their relative energies compared. The calculation geometries were processed using the *Atomic Simulation Environment* (ASE),[9] with DFT calculations performed via the *Fritz Haber Institute - ab initio molecular simulations* (FHI-aims)[10] software package. The M06[11] functional was used to approximate the exchange-correlation (XC) energy as it provides accurate dissociation energies for organometallics, with errors reported as 1.6 kcal mol^{-1} for a test set of 30 organometallic complexes.[12] A "*light*" basis, which is equivalent to the split-valence double-zeta with polarization (DZP) basis set, was used from the FHI-aims package (version: *2020*). In addition to being efficient, the "*light*" basis set gives trends among structural analogues due to low error.[10] Relativistic effects were considered using the *Zero-Order Regular Approximation* (ZORA)[13] scalar formalism. The structures were relaxed using the *Broyden–Fletcher–Goldfarb–Shanno* (BFGS)[14,15] optimizer until the forces on each atom (f_{max}) were less than 0.01 eV/Å.

For transition state calculations, 13 linearly interpolated images were generated between the closed and open geometries of both catalysts, *i.e.*, 15 images in total. The images were combined with the *Climbing Image – Nudged Elastic Band* (CI-NEB)[16] method for transition state searches, employing the *Fast Inertial Relaxation Engine* (FIRE)[17] minimizer with $f_{\text{max}} = 0.05$ eV/Å. Initially, the MACE-omol forcefield[18,19] was used to estimate the geometries of the 13 minimum energy pathway (MEP) images to optimize computational resources. The identified MEP geometries were then used as starting points for the transition state search with the M06 functional. The final transition state (TS) structure identified using the M06 functional was confirmed as a true saddle point through a frequency calculation with the same functional, using the finite difference method with a 0.01 Å displacement. The presence of a single imaginary frequency validated the identified TS.

The stability of the complexes were studied using the reaction energy (ΔE_{reax}) and the activation energy (ΔE_{act}) which were calculated as:

$$\Delta E_{\text{reax}} = E_{\text{1P\_open}} - E_{\text{closed}} \quad \text{or} \quad E_{\text{2P\_open}} - E_{\text{1P\_open}}$$

$$\Delta E_{act} = E_{TS1} - E_{closed} \quad \text{or} \quad E_{TS2} - E_{1P\_open}$$

Where E_{1P_open} and E_{2P_open} are the energies of the conformers with one (*1P_open*) and two (*2P_open*) phosphonite arms open, respectively; and E_{TS1} and E_{TS2} are the energies of the transition states between the closed and *1P_open* conformers, and between the *1P_open* and *2P_open* conformers, respectively. The results are tabulated in Table S4.

For the orbital analysis, single-point energy calculations were performed on the optimized structures and the TS using the Gaussian 09 package[20] with the B3LYP exchange-correlation density functional and a 6-31+g(d,p) basis set. The orbitals were visualized using the checkpoint file with an isosurface density of 0.02 e^-/Å^3.

Table S4: Calculated energies (in kcal/mol) for conversion between the open and closed complexes containing Ga, Al or In. Values are derived as explained in the text.

**Metal**	ΔE_{act} ($E_{TS1} - E_{closed}$)	ΔE_{reax} ($E_{1P\_open} - E_{closed}$)	ΔE_{act} ($E_{TS2} - E_{1P\_open}$)	ΔE_{reax} ($E_{2P\_open} - E_{1P\_open}$)
Ga	17.83	14.13	35.48	27.93
Al	19.50	17.20	31.53	23.47
In	22.70	20.62	-	-

The DFT calculation files that underpin the results presented in this article can be found on DOI: 10.6084/m9.figshare.28485857.

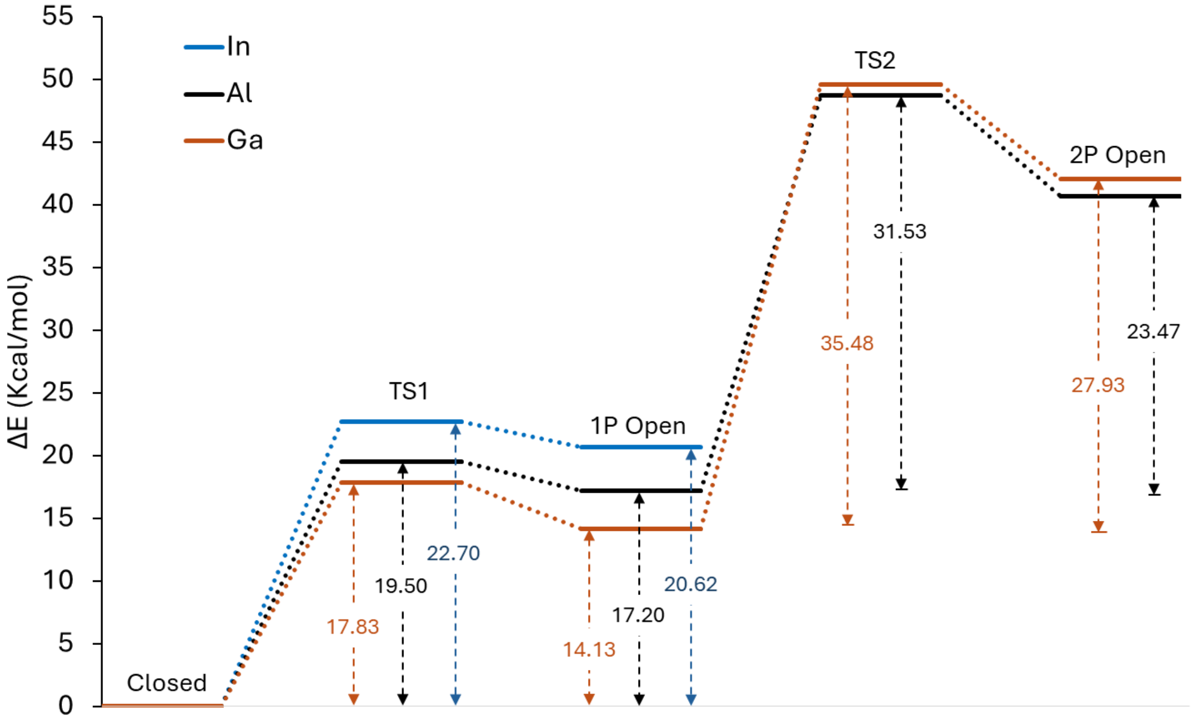

Figure S89: DFT calculated energy profile of In, Al and Ga complexes going from their closed conformer to a conformer with both phosphinite arms open (2P open), via the conformer with a single phosphinite arm open (1P open). Transition states (TS1 and TS2) are identified.

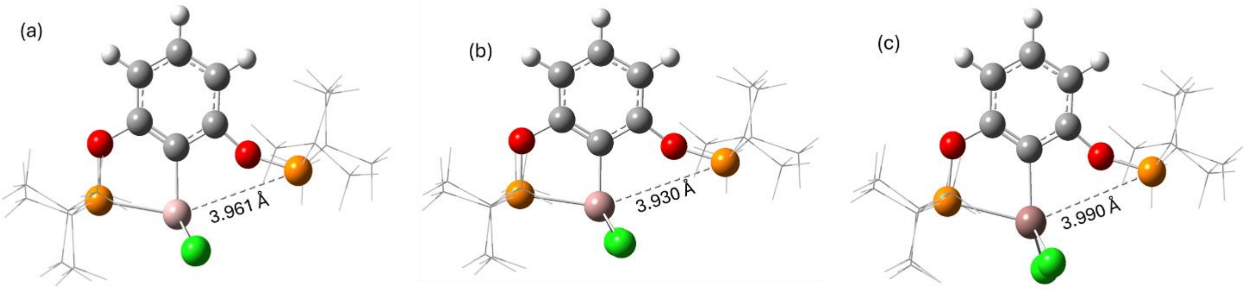

Figure S90: Transition state structures for the interconversion between closed conformers and a conformer with one phosphinite arm open: (a) Al (pink), (b) Ga (pink), and (c) In (brown) complexes. Carbon atoms are shown in grey, oxygen in red, hydrogen in white, phosphorus in yellow, and chlorine in green; the ^{t}Bu groups are shown as wireframe. The distance between the metal center and the detaching phosphorus atom is reported in angstroms.

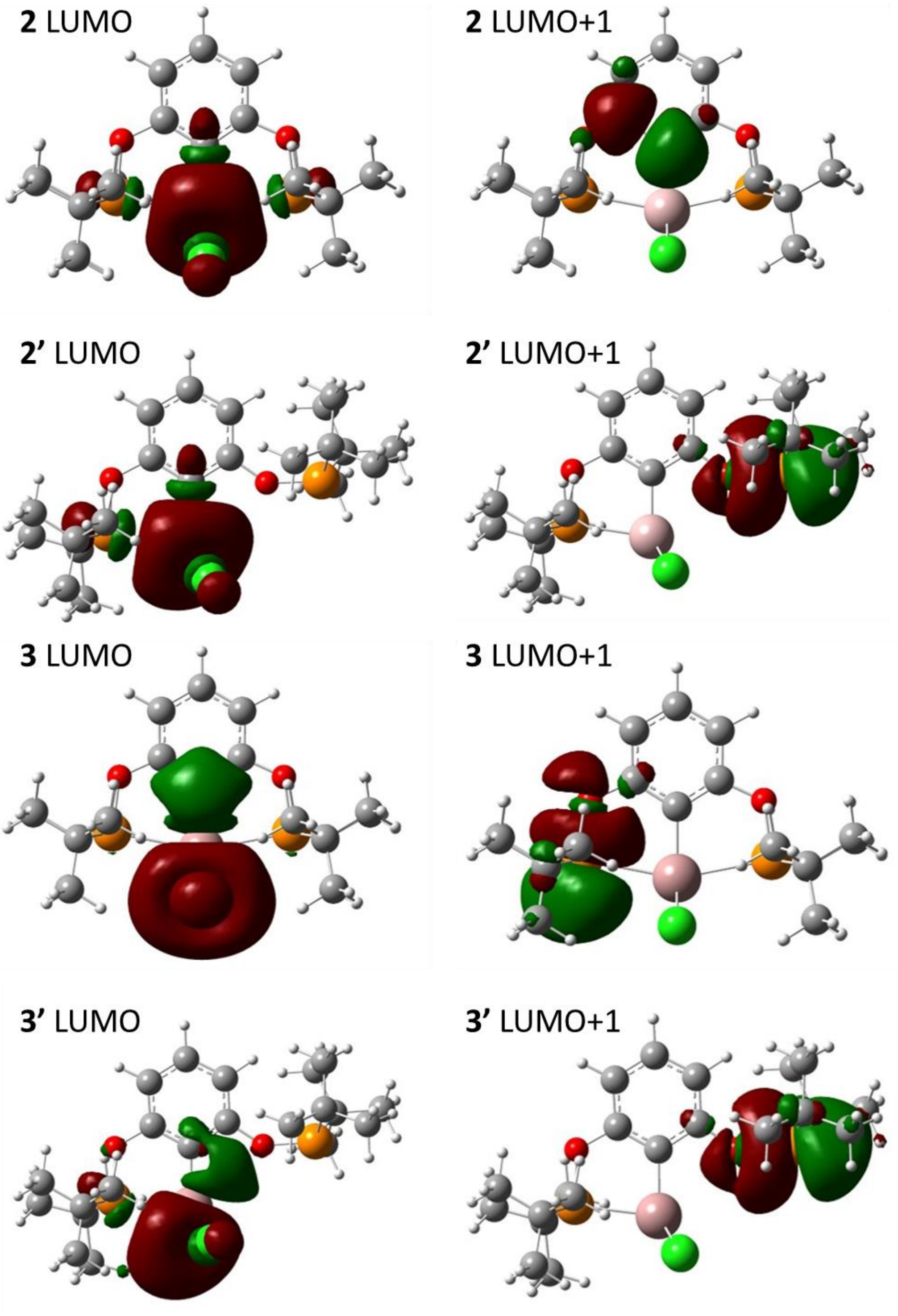

Figure S91: Calculated LUMO and LUMO+1 for the closed (**2**, **3**) and open (**2'**, **3'**) aluminum and gallium complexes. C is shown in grey, O in red, P in orange, Cl in green, and Al in pink. Green represents positive isosurface and red represents negative isosurface.

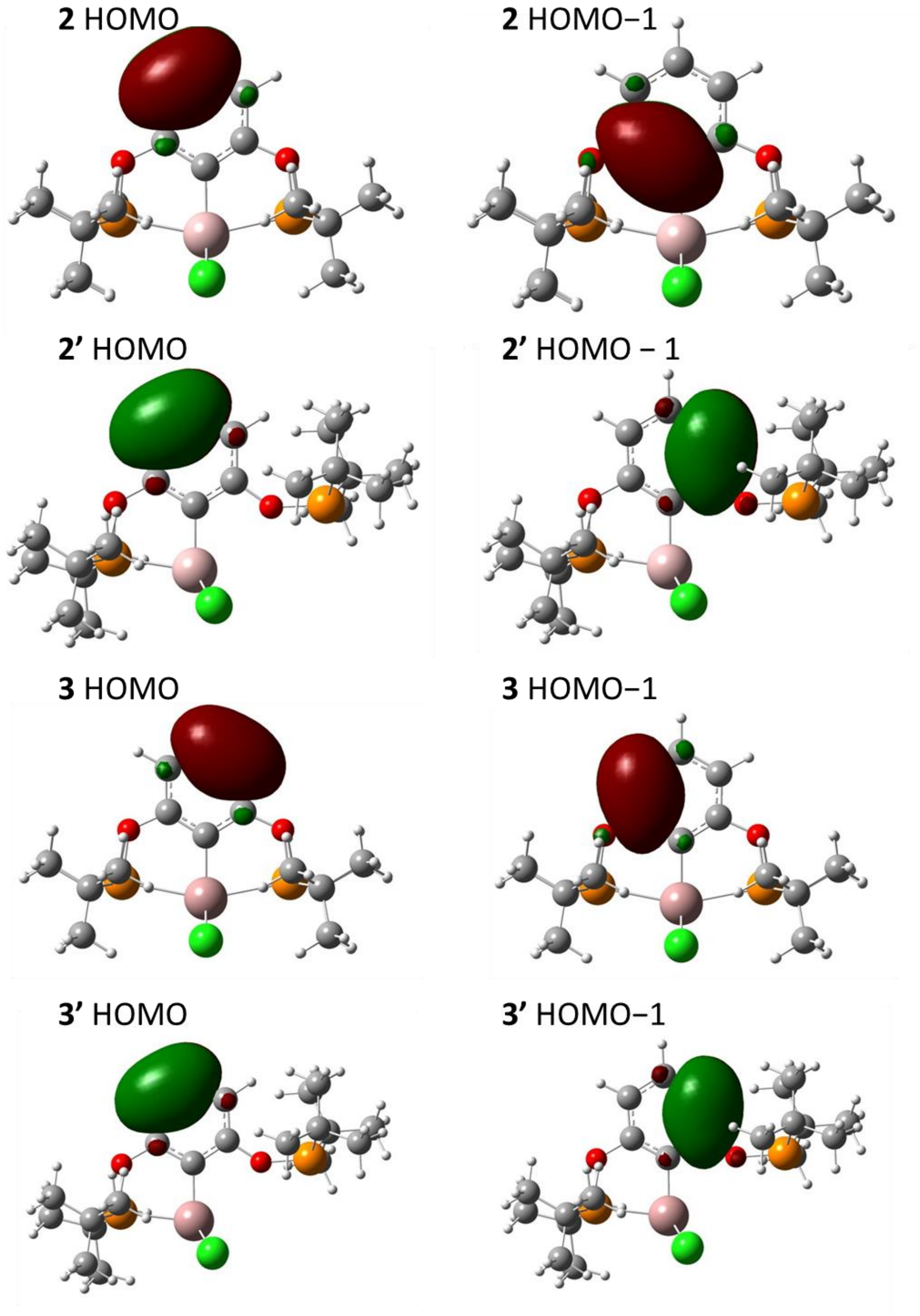

Figure S92: Calculated HOMO and HOMO−1 for the closed (**2**, **3**) and open (**2'**, **3'**) aluminum and gallium complexes. C is shown in grey, O in red, P in orange, Cl in green, and Al in pink. Green represents positive isosurface and red represents negative isosurface.

9. References

(1) Zhang, J.; Huang, W.; Han, K.; Song, G.; Hu, S. Scandium, Titanium and Vanadium Complexes Supported by PCP-Type Pincer Ligands: Synthesis, Structure, and Styrene Polymerization Activity. *Dalton Trans.* **2022**, *51* (32), 12250–12257. https://doi.org/10.1039/D2DT01389D.

(2) Rigaku Oxford Diffraction, (2018), CrysAlisPro Software System, Version 1.171.40.45a, Rigaku Corporation, Oxford, UK.

(3) Allan, D. R.; Nowell, H.; Barnett, S. A.; Warren, M. R.; Wilcox, A.; Christensen, J.; Saunders, L. K.; Peach, A.; Hooper, M. T.; Zaja, L.; Patel, S.; Cahill, L.; Marshall, R.; Trimnell, S.; Foster, A. J.; Bates, T.; Lay, S.; Williams, M. A.; Hathaway, P. V.; Winter, G.; Gerstel, M.; Wooley, R. W. A Novel Dual Air-Bearing Fixed-χ Diffractometer for Small-Molecule Single-Crystal X-Ray Diffraction on Beamline I19 at Diamond Light Source. *Crystals* **2017**, *7* (11), 336. https://doi.org/10.3390/cryst7110336.

(4) Winter, G.; Waterman, D. G.; Parkhurst, J. M.; Brewster, A. S.; Gildea, R. J.; Gerstel, M.; Fuentes-Montero, L.; Vollmar, M.; Michels-Clark, T.; Young, I. D.; Sauter, N. K.; Evans, G. DIALS: Implementation and Evaluation of a New Integration Package. *Acta. Cryst. D* **2018**, *74* (2), 85–97. https://doi.org/10.1107/S2059798317017235.

(5) AIMLESS (CCP4: Supported Program): Journal, 2018, CCP4 7.0.062: AIMLESS, Version 060.067.062: 027/005/018.

(6) Dolomanov, O. V.; Bourhis, L. J.; Gildea, R. J.; Howard, J. a. K.; Puschmann, H. OLEX2: A Complete Structure Solution, Refinement and Analysis Program. *J. Appl. Cryst.* **2009**, *42* (2), 339–341. https://doi.org/10.1107/S0021889808042726.

(7) Sheldrick, G. M. SHELXT – Integrated Space-Group and Crystal-Structure Determination. *Acta. Cryst. A.* **2015**, *71* (1), 3–8. https://doi.org/10.1107/S2053273314026370.

(8) Sheldrick, G. M. Crystal Structure Refinement with SHELXL. *Acta. Cryst. C.* **2015**, *71* (1), 3–8. https://doi.org/10.1107/S2053229614024218.

(9) Hjorth Larsen, A.; Jørgen Mortensen, J.; Blomqvist, J.; Castelli, I. E.; Christensen, R.; Dułak, M.; Friis, J.; Groves, M. N.; Hammer, B.; Hargus, C.; Hermes, E. D.; Jennings, P. C.; Bjerre Jensen, P.; Kermode, J.; Kitchin, J. R.; Leonhard Kolsbjerg, E.; Kubal, J.; Kaasbjerg, K.; Lysgaard, S.; Bergmann Maronsson, J.; Maxson, T.; Olsen, T.; Pastewka, L.; Peterson, A.; Rostgaard, C.; Schiøtz, J.; Schütt, O.; Strange, M.; Thygesen, K. S.; Vegge, T.; Vilhelmsen, L.; Walter, M.; Zeng, Z.; Jacobsen, K. W. The Atomic Simulation Environment—a Python Library for Working with Atoms. *J. Phys. Condens. Matter* **2017**, *29* (27), 273002. https://doi.org/10.1088/1361-648X/aa680e.

(10) Blum, V.; Gehrke, R.; Hanke, F.; Havu, P.; Havu, V.; Ren, X.; Reuter, K.; Scheffler, M. Ab Initio Molecular Simulations with Numeric Atom-Centered Orbitals. *Comput. Phys. Commun.* **2009**, *180* (11), 2175–2196. https://doi.org/10.1016/j.cpc.2009.06.022.

(11) Zhao, Y.; Truhlar, D. G. The M06 Suite of Density Functionals for Main Group Thermochemistry, Thermochemical Kinetics, Noncovalent Interactions, Excited States, and Transition Elements: Two New Functionals and Systematic Testing of Four M06-Class Functionals and 12 Other Functionals. *Theor. Chem. Acc.* **2008**, *120* (1–3), 215–241. https://doi.org/10.1007/s00214-007-0310-x.

(12) Raju, R. K.; Bengali, A. A.; Brothers, E. N. A Unified Set of Experimental Organometallic Data Used to Evaluate Modern Theoretical Methods. *Dalton Trans.* **2016**, *45* (35), 13766–13778. https://doi.org/10.1039/C6DT02763F.

(13) Van Lenthe, E.; Baerends, E. J.; Snijders, J. G. Relativistic Total Energy Using Regular Approximations. *J. Chem. Phys.* **1994**, *101* (11), 9783–9792. https://doi.org/10.1063/1.467943.

(14) Broyden, C. G. The Convergence of a Class of Double-Rank Minimization Algorithms: 2. The New Algorithm. *IMA J. Appl. Math.* **1970**, *6* (3), 222–231. https://doi.org/10.1093/imamat/6.3.222.

(15) Fletcher, R. A New Approach to Variable Metric Algorithms. *Comput. J.* **1970**, *13* (3), 317–322. https://doi.org/10.1093/comjnl/13.3.317.

(16) Henkelman, G.; Uberuaga, B. P.; Jónsson, H. A Climbing Image Nudged Elastic Band Method for Finding Saddle Points and Minimum Energy Paths. *J. Chem. Phys.* **2000**, *113* (22), 9901–9904. https://doi.org/10.1063/1.1329672.

(17) Bitzek, E.; Koskinen, P.; Gähler, F.; Moseler, M.; Gumbsch, P. Structural Relaxation Made Simple. *Phys. Rev. Lett.* **2006**, *97* (17), 170201. https://doi.org/10.1103/PhysRevLett.97.170201.

(18) Batatia, I.; Kovács, D. P.; Simm, G. N. C.; Ortner, C.; Csányi, G. MACE: Higher Order Equivariant Message Passing Neural Networks for Fast and Accurate Force Fields. arXiv 2022. https://doi.org/10.48550/ARXIV.2206.07697.

(19) Levine, D. S.; Shuaibi, M.; Spotte-Smith, E. W. C.; Taylor, M. G.; Hasyim, M. R.; Michel, K.; Batatia, I.; Csányi, G.; Dzamba, M.; Eastman, P.; Frey, N. C.; Fu, X.; Gharakhanyan, V.; Krishnapriyan, A. S.; Rackers, J. A.; Raja, S.; Rizvi, A.; Rosen, A. S.; Ulissi, Z.; Vargas, S.; Zitnick, C. L.; Blau, S. M.; Wood, B. M. The Open Molecules 2025 (OMol25) Dataset, Evaluations, and Models. arXiv 2025. https://doi.org/10.48550/ARXIV.2505.08762.

(20) Frisch, M. J.; Trucks, G. W.; Schlegel, H. B.; Scuseria, G. E.; Robb, M. A.; Cheeseman, J. R.; Scalmani, G.; Barone, V.; Mennucci, B.; Petersson, G. A.; Nakatsuji, H.; Caricato, M.; Li, X.; Hratchian, H. P.; Izmaylov, A. F.; Bloino, J.; Zheng, G.; Sonnenberg, J. L.; Hada, M.; Ehara, M.; Toyota, K.; Fukuda, R.; Hasegawa, J.; Ishida, M.; Nakajima, T.; Honda, Y.; Kitao, O.; Nakai, H.; Vreven, T.; Montgomery, J. A.; Peralta, J. E.; Ogliaro, F.; Bearpark, M.; Heyd, J. J.; Brothers, E.; Kudin, K. N.; Staroverov, V. N.; Kobayashi, R.; Normand, J.; Raghavachari, K.; Rendell, A.; Burant, J. C.; Iyengar, S. S.; Tomasi, J.; Cossi, M.; Rega, N.; Millam, J. M.; Klene, M.; Knox, J. E.; Cross, J. B.; Bakken, V.; Adamo, C.; Jaramillo, J.; Gomperts, R.; Stratmann, R. E.; Yazyev, O.; Austin, A. J.; Cammi, R.; Pomelli, C.; Ochterski, J. W.; Martin, R. L.; Morokuma, K.; Zakrzewski, V. G.; Voth, G. A.; Salvador, P.; Dannenberg, J. J.; Dapprich, S.; Daniels, A. D.; Farkas, O.; Foresman, J. B.; Ortiz, J. V.; Cioslowski, J.; Fox, D. J. GAUSSIAN 09 (Revision D.01) 2013, Gaussian, Inc., Wallingford, CT.
